# Highly stereoselective synthesis of complex molecules *via* coupling of chiral carbamates with chiral boronic acid esters

**DOI:** 10.1039/d6ra03391a

**Published:** 2026-07-04

**Authors:** Max Benjamin Becker, Uli Kazmaier

**Affiliations:** a Organic Chemistry, Saarland University P.O. Box 151150 66041 Saarbrücken Germany u.kazmaier@mx.uni-saarland.de

## Abstract

Matteson homologation not only allows the highly stereoselective synthesis of complex chiral boronic acid esters, but also the formation of chiral secondary alcohols through their oxidation. By converting them into chiral carbamates, they can be coupled with chiral boronic acid esters after deprotonation, whereby tertiary boronic acid esters can be obtained highly stereoselectively (d.r. ≥ 97 : 3). No matched/mismatched situation is observed, *i.e.* arbitrarily configured building blocks can be coupled, which predestines this method for the synthesis of complex molecules, such as natural products.

## Abstract 2

The reaction of chiral secondary carbamates with chiral alkylboronic esters allows the highly stereoselective synthesis of complex tertiary boronic esters.

## Graphical abstract

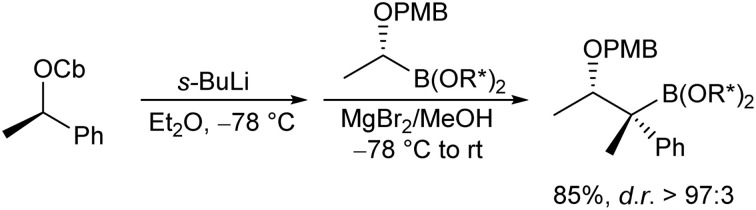

## Introduction

**Fig. 1:**
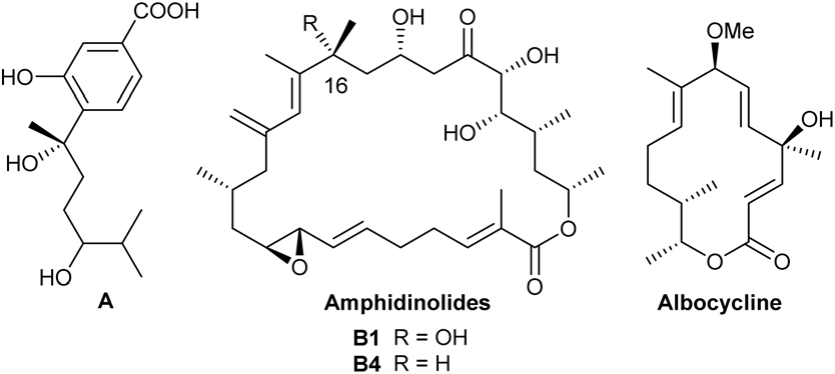
Natural products containing chiral tertiary alcohols.

Natural products play a central role in the development of active ingredients and pharmaceuticals. In the years 1981–2006, for example, about 50% of all newly discovered active substances were compounds derived from natural products,^1^ produced by plants, fungi and microorganisms.^2^ The structures of the isolated compounds are as diverse as their producers (Fig. 1). For example, phenolic bisabolane sesquiterpenoid A was isolated from the Thai mangrove endophytic fungus, *Aspergillus* sp*.* xy02, along with some other derivatives, and shows antibacterial activities against the Gram positive strain *Staphylococcus aureus* ATCC 25923.^3^ The amphidinolides B1 and B4, two representatives of the very large substance class of amphidinolides, were isolated as secondary metabolites from *Amphidinium* spp., a symbiotic marine dinoflagellate separated from inside cells of Okinawan marine flatworms.^4^ The amphidinolides show interesting antimicrobial, antifungal, and anticancer activities.^5^ The two amphidinolides B1 and B4 differ only in position 16, which is hydroxylated to a tertiary alcohol in the case of amphidinolides B1.

And finally, albocycline is a macrolactone obtained from *Streptomyces maizeus*. Albocycline displays antimicrobial properties against *S. aureus* (ATCC 25923) with an activity against resistant *S. aureus* strains which is superior to that of vancomycin.^6^

All these natural substances contain tertiary benzylic or allylic alcohol functionalities as characteristic structural features, which occur much less frequently in nature than the classical secondary alcohols, which result from the polyketide synthesis pathway. While methods for their stereoselective assembly, whether by aldol reactions or allylation/ozonolysis sequences, are well established, stereoselective synthesis of tertiary alcohols still poses a synthetic challenge. Of particular interest are synthetic routes that allow the generation of several derivatives from a common precursor, as in the case of amphidinolides (R=OH, H,…).

**Scheme 1:**
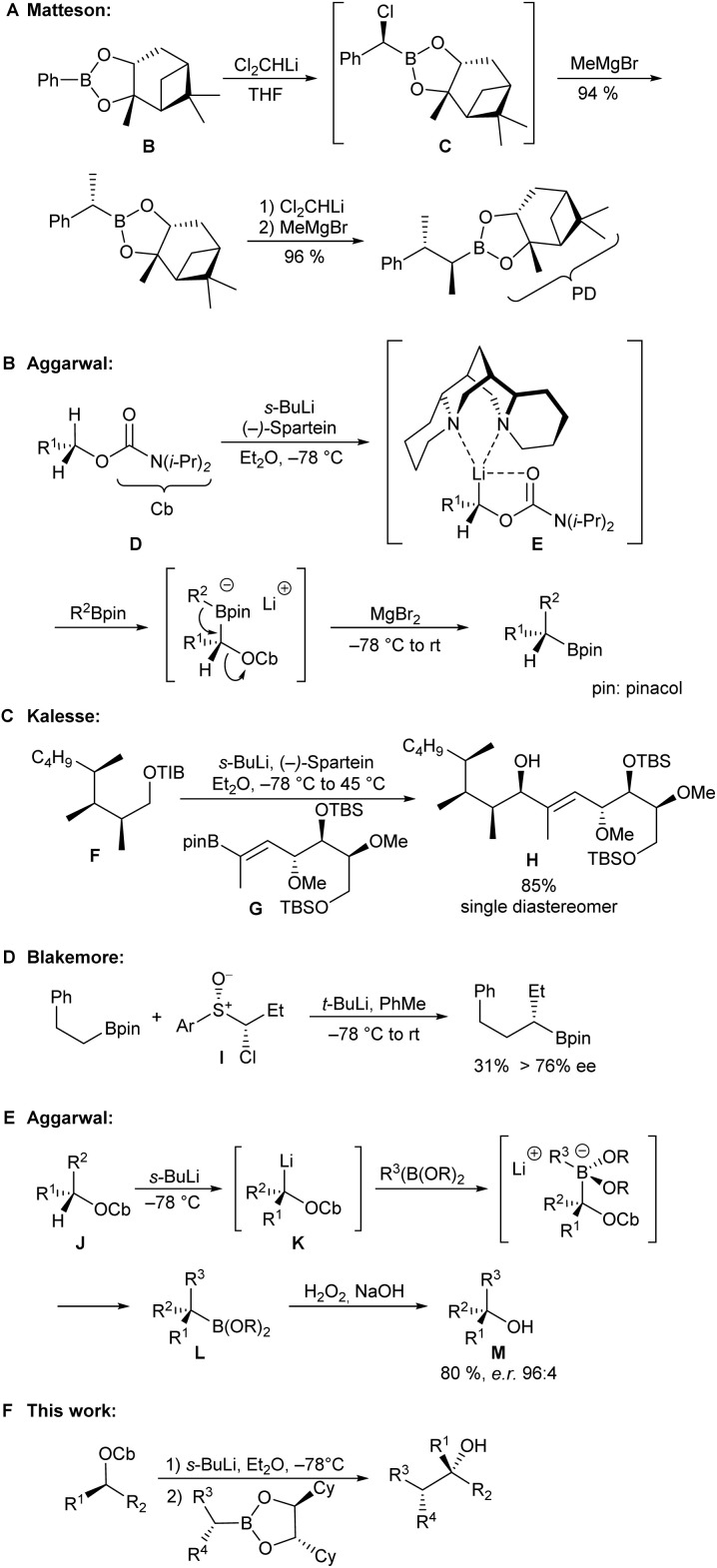
Matteson homologations and related reactions.

In addition to the already mentioned methods for the stereoselective assembly of polyketide structures, Matteson homologation is particularly suitable for the formation of highly substituted and functionalized alkyl chains (Scheme 1A).^7^ Matteson *et al.* 1980 were able to show that chiral boronic acid esters such as B can be highly stereoselective reacted at low temperature with deprotonated dichloromethane to the C1-extended α-chloroboronic acid esters C,^8^ which can be further reacted in a subsequent step with a large number of nucleophiles under S_N_2 conditions. With pinanediol (PD) as a chiral auxiliary, diastereoselectivities of 98.5–99.5% could be achieved under these conditions. By using chiral, C_2_-symmetrical diols, a further increase in diastereoselectivities is possible. Very good results can be achieved, for example, with diisopropylethandiol (DIPED)^9^ and dicyclohexylethandiol (DICHED).^10^

Since the chiral boronic acid ester remains in the molecule during the reaction, it can be used for further homologation steps. Therefore, the Matteson homologation is ideally suited for iterative setup of neighbouring stereocenters with high enantio- and diastereoselectivities.^11^ In addition to Grignard reagents, a variety of other nucleophiles can be used in the substitution step, such as enolates,^12^ alcoholates^13^ and *N*-nucleophiles.^14^ Since the stereochemical course of the reaction is almost exclusively controlled by the chiral auxiliary, excellent diastereoselectivities are usually obtained. As a rule, 1,2-*anti*-configured products are formed under standard conditions.^15^

This stereochemical limitations of the classical Matteson reaction prompted Aggarwal *et al.* to develop a reagent-controlled variant.^16^ based on Hoppe's chiral lithiated carbamates D (Scheme 1B).^17^ In this process, achiral primary carbamates are deprotonated in the presence of a chiral base, usually sparteine, and then reacted with achiral boronic acid pinacol esters. The stereoselectivity in this case is determined by the configuration of the lithiated carbamate E.^16^ This method also allows a continuous extension of a C-chain, whereby the configuration of each newly introduced stereogenic centre can be controlled individually, depending on the configuration of the chiral base.

The disadvantage of this procedure, however, is that stoichiometric amounts of chiral auxiliary base are required for each homologation step, which is not unproblematic, especially in the case of sparteine due to poor availability. Nevertheless, this method was used in the synthesis of a number of natural products.^18^ Recently, Bojaryn and Hirschhäuser reported on the conversion of sparteine-complexed lithium carbenoids E with chiral α-bromoboronic acid esters. Depending on the choice of the chiral auxiliary, diastereomeric products could be obtained, which were used in the syntheses of protected glycols.^19^

The fact that the use of chiral auxiliary bases can be dispensed with under certain circumstances is shown by work by Kalesse *et al.*^21^ (Scheme 1C). During the synthesis of chondrochlorene, the chiral 2,4,6-triisopropylbenzoyl ester (TIP) F should be coupled with the vinylogous boronic acid pinacol ester G.^20^ Both (+)- and (−)-sparteine yielded only moderate yields, while in the absence of the ligand a yield of 85% could be obtained, whereby only one diastereomer of H was formed.^21^ Interestingly, the *N*,*N*-diisopropylcarbamate (Cb) provided the opposite diastereomer.^22^

Blakemore *et al.* were the first to describe a sparteine-free “lithiation–borylation” reaction based on the generation of chiral lithium reagents from chiral α-sulfinyl chlorides (I) (Scheme 1D).^23^ However, these are not easy to produce and are not particularly stable. Also, the yields and enantioselectivities are often only moderate, which is why this method has not really prevailed so far.^24^

In principle, the use of sparteine can also be surrendered if carbamates of secondary chiral alcohols J are deprotonated under suitable conditions. Based on the work of Hoppe *et al.*^25^ Aggarwal *et al.* were able to show that benzylic^26^ and allylic carbamates^27^ are particularly suitable for deprotonation, whereby the configuration at the chiral centre was largely preserved (Scheme 1E). The lithiated species K could then be reacted with boronic acid esters under retention to form the corresponding tertiary boronic acid esters L. Their oxidation lead to the chiral tertiary alcohols M, whereby a high degree of chirality transfer was observed. If the chiral carbenoids K are reacted with boranes, the corresponding enantiomeric tertiary alcohols can be obtained.^28^

Recently, Hirschhäuser *et al.* reported on the implementation of Beak's chiral lithiated benzoates with chiral boronic acid esters, which provides the diastereomeric products in addition to the classic Matteson products, depending on the chiral lithium carbenoid used.^29^ These were obtained by transmetalation from the enantiomerically pure tin analogues,^30^ which in turn were obtained using the sparteine method. Depending on the substrate used, both the yields and the diastereoselectivities vary relatively strongly, even if diastereoselectivities of 99 : 1 were achieved in individual cases.

It should not go unmentioned that catalytic, enantioselective variants of Matteson homologation were also developed, but early work with chiral Lewis acids yielded only moderate enantioselectivities and required high catalyst loadings.^31^ Jacobsen *et al.* achieved significantly better results with a lithium isothiourea–borate complex.^32^ With this organocatalyst, various boronic acid pinacol esters could be homologated with good yield and enantioselectivity. Recently, Dong *et al.* reported other variants of the Matteson reaction that allow the introduction of *O*- and *N*-functionalities.^33^

## Results and discussion

Our research group has been working on the further expansion of the classical Matteson homologation^34^ and its application in natural product syntheses for a couple of years.^35^ For example, we were recently able to report on a protocol that allows the synthesis of epimeric and tertiary boronic acid esters, which are normally not easily accessible *via* Matteson reaction.^36^ Since complex secondary alcohols, and thus also carbamates, can be generated easily *via* Matteson homologation, we wanted to see whether these carbamates could also be reacted with more complex boronic acid esters, which may also be obtained *via* Matteson reaction (Scheme 1). This should allow the stereoselective synthesis of complex tertiary alcohols *via* coupling of arbitrarily substituted fragments. To the best of our knowledge, reactions of this kind have not been described in the literature or applied in further syntheses so far.

**Fig. 2:**
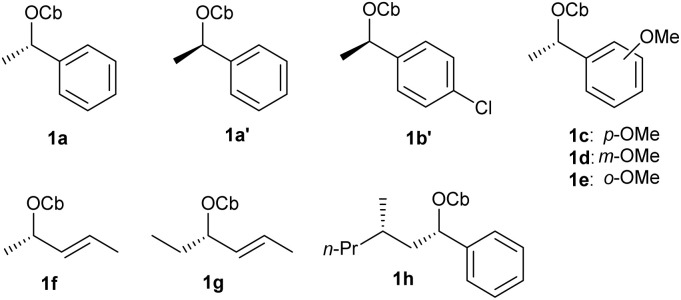
Chiral carbamates 1.

For our investigations, we started with the synthesis of various chiral carbamates 1.^27^ Either commercially available chiral alcohols were used, or secondary alcohols were synthesized according to literature and, if necessary, subjected to an enzymatic kinetic resolution using novozyme.^37^Fig. 2 summarizes the derivatives synthesized.

**Scheme 2:**
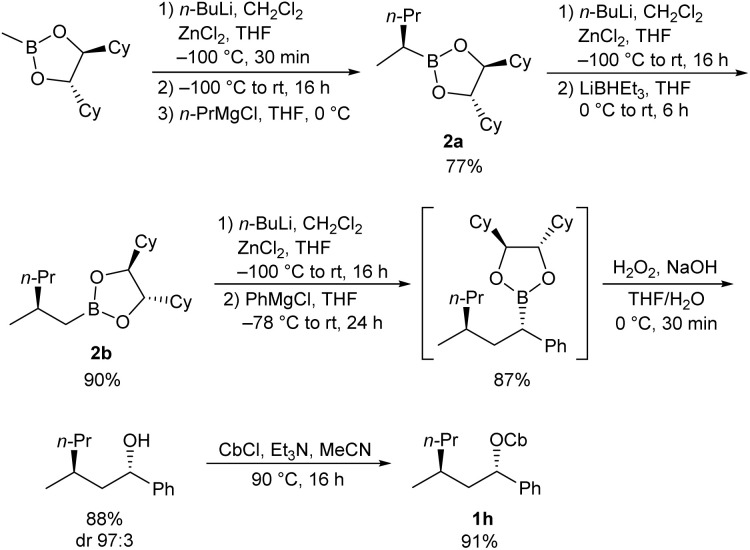
Stereoselective synthesis of carbamate 1i and boronic acid esters 2a and 2b.

In addition to benzylic carbamates (1a–1e) and some of their enantiomers (1x′), allyl carbamates^27^ (1f and 1g) were also synthesized to see whether tertiary allyl alcohols, such as in amphidinolides or albocyclines, could also be obtained. Carbamate 1h was finally synthesized to find out whether somewhat more complex carbamates could also be used in the reaction. 1h was obtained *via* Matteson homologation, together with the chiral boronic acid esters 2a and 2b. Their synthesis is outlined in Scheme 2.

**Fig. 3:**
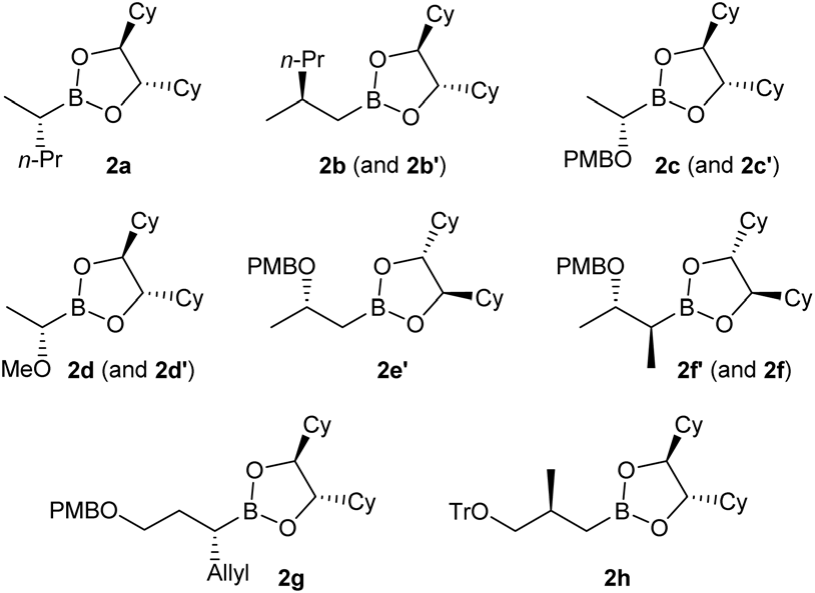
DICHED boronic acid esters 2 and 2′ (enantiomers).

The other chiral boron esters 2 synthesized for the investigations (Fig. 3) were either known in the literature or were obtained analogously to 2a/b according to standard methods.

**Scheme 3:**
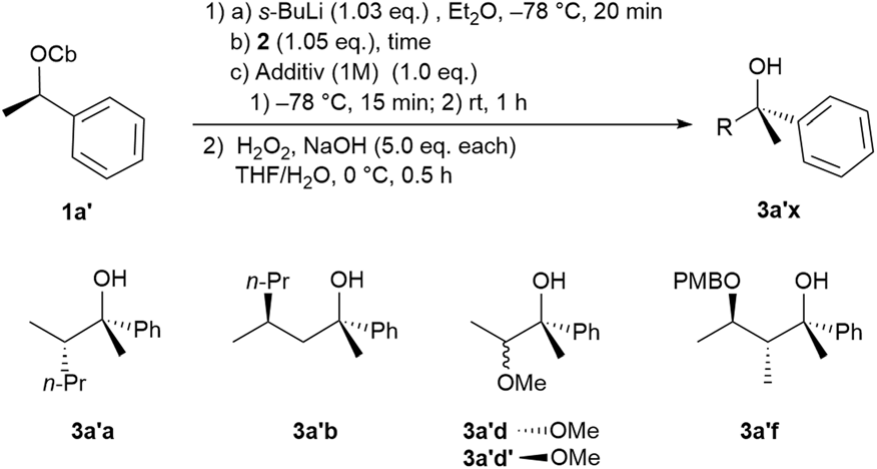
Coupling of chiral deprotonated carbamate 1a′ with different chiral boronic acid esters 2.

In order to find optimal conditions for the conversion of carbamate 1 with the DICHED boronic acid esters 2, various test reactions with carbamate 1a′ were first carried out (Scheme 3 and Table 1). Starting with α-alkylboronic acid ester 2a, the desired tertiary alcohol 3a′a could be obtained in moderate yield and diastereoselectivity according to a literature protocol (Table 1, entry 1).^28a^ The intermediately formed boronic acid ester was briefly purified by flash chromatography, not isolated, but directly subjected to the oxidation conditions. This workup protocol was used in all further reactions.

**Table 1:** Coupling of chiral carbamate 1a′ with different chiral boronic acid esters 2

Entry	2	Solvent	Additive	Time	Product	Yield	dr^c^
1^a^	2a	Et_2_O	—	30 min	3a′a	63%	8 : 2
2^a^	2a	THF	ZnCl_2_	30 min	3a′a	31%	7 : 3
3^a^	2a	Et_2_O	MgBr_2_/MeOH	30 min	3a′a	64%	>97 : 3
4^b^	2a	Et_2_O	MgBr_2_/MeOH	30 min	3a′a	81%	>97 : 3
5^b^	2a	Et_2_O	MgBr_2_/MeOH	14 h	3a′a	87%	>97 : 3
6^a^	2b	Et_2_O	—	30 min	3a′b	53%	74 : 26
7^a^	2b	Et_2_O	MgBr_2_/MeOH	30 min	3a′b	64%	>97 : 3
8^a^	2f	Et_2_O	—	30 min	3a′f	25%	42 : 58
9^a^	2f	Et_2_O	MgBr_2_/MeOH	180 min	3a′f	62%	>97 : 3
10^a^	2d	Et_2_O	—	60 min	3a′d	65%	97 : 3
11^b^	2d′	Et_2_O	—	60 min	3a′d′	69%	>97 : 3
12^b^	2d′	Et_2_O	MgBr_2_/MeOH	60 min	3a′d′	72%	>97 : 3

a Batch size: 300–400 µmol.

b Batch size: 1.00 mmol.

c Determined *via*^1^H NMR.

Attempts to achieve better results by changing the solvent failed. In THF, for example, both the yield and the selectivity were significantly worse, and the addition of a Lewis acid to accelerate the 1,2-rearrangement, as is often successful in Matteson homologation, did not bring any improvement (entry 2). On the other hand, the addition of MgBr_2_ in MeOH provided the tertiary alcohol with an excellent diastereomer ratio (entry 3). In this case, the addition of MgBr_2_/MeOH probably reduces the reversibility of the boronate complex formation in favour of the 1,2-rearrangement. MgBr_2_ serves to accelerate the re-arrangement, while MeOH protonates the carbamate anion released and thus withdraws it from equilibrium.^28^

Interestingly, by changing the batch size from 300 µmol to 1.00 mmol, the yield could be significantly improved (entries 4 and 5). Similar observations on the influence of reaction scaling have been made in previous Matteson homologations of DICHED-boronic acid esters. The significant influence of MgBr_2_ in MeOH on yield and diastereoselectivity could also be observed with other boronic acid esters, such as the β-substituted ester 2b (entries 6 and 7) and the enantiomeric boronic ester 2f (entries 8 and 9). Obviously, there is no matched/mismatched situation here, because both yield and selectivity were completely comparable to the other experiments. Also, the alkoxy substituent in β-position of 2f does not seem to have a significant effect on the outcome of the reaction, in contrast to the reaction of boronic ester 2d, with an alkoxy group in α-position (entry 10). Here, comparable yields and selectivity's were already obtained without the addition of MgBr_2_/MeOH, as well as with the enantiomeric boron ester 2d′ (entry 11). The addition of MgBr_2_/MeOH had no effect on the result in this case (entry 12). Attempts to increase the yield by increasing the amount of boronic acid ester (1.5 equiv.) did not result in any further significant improvement.

**Scheme 4:**
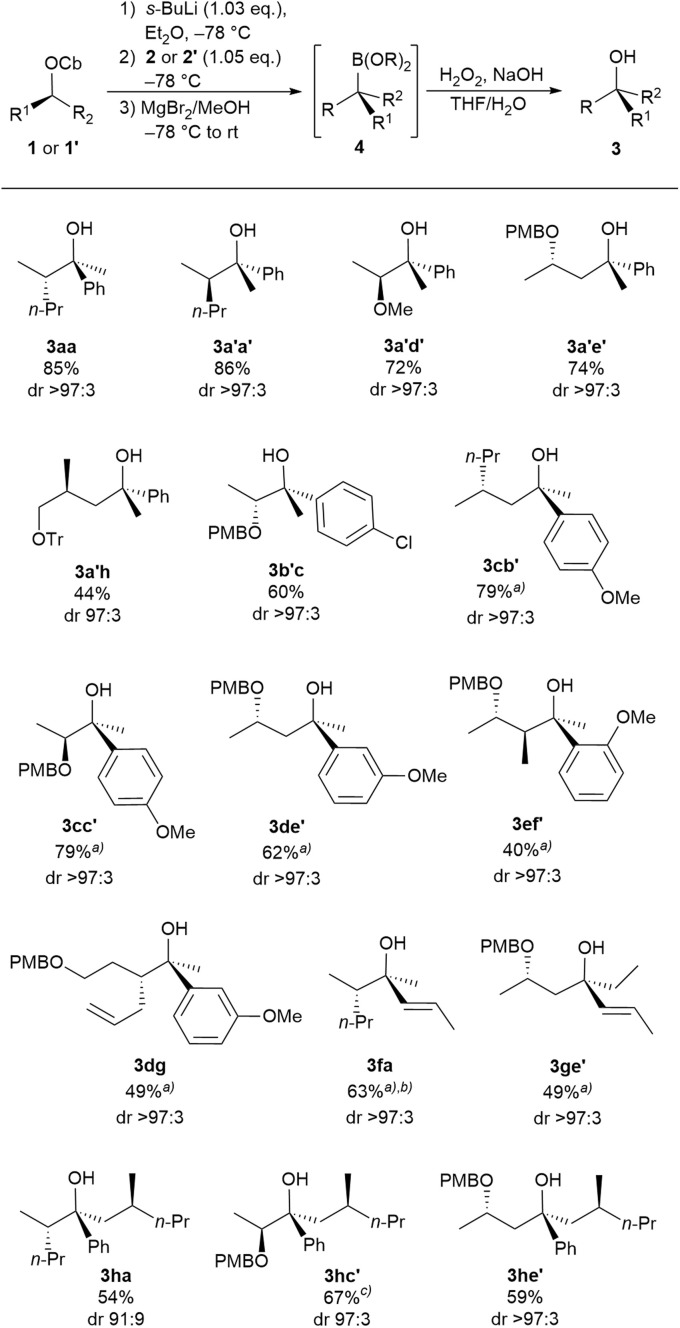
Synthesis of chiral tertiary alcohols 3 from boronic acid esters 2 (a) deprotonation in the presence of 1.1 eq. TMEDA; (b) use of 1.2 equiv. 2g; (c) use of 1.4 equiv. 2c′.

**Fig. 4:**
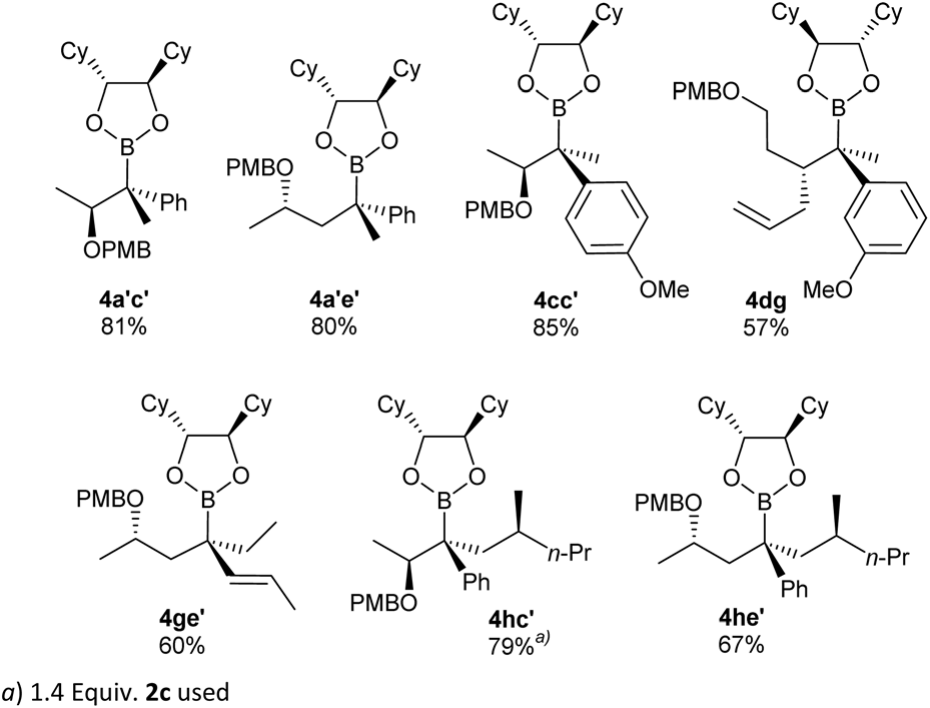
Isolated tertiary boronic acid esters 4. (a) 1.4 Equiv. 2c used.

Under the optimized reaction conditions (entries 4 and 5), we performed the homologations of the different boronic acid esters 2 (Fig. 2). In some cases, the newly formed tertiary boronic acid esters 4 could not be separated chromatographically from the starting boronic acid esters 2, so that in all cases the briefly purified boronic ester was oxidized to the corresponding alcohol 3, which could be purified without any problems (Scheme 4). The yields given in Scheme 4 therefore refer to two steps. The boronic acid esters 4, which could be purified by chromatography and purely isolated, are summarized in Fig. 4.

The synthesis of the stereoisomeric alcohols 3aa and 3a′a′ was used to re-examine whether the chiral stereogenic centres in the carbamate and the boronic acid ester mutually influence each other and whether a matched/mismatched situation occurs, but this was not observed here either. Both the yields and diastereoselectivities were approximately identical to the results obtained for 3a′a (Table 1, entry 4). Electron-rich benzyl carbamates deliver slightly better yields than electron-poor systems (3a′d′, 3b′c and 3cc′) under comparable conditions.

Apart from a few exceptions, such as 3ha, almost exclusively the formation of a single stereoisomer was observed, as could be clearly demonstrated *via* NMR and HPLC. The tertiary alcohols (3ha, 3hc′ and 3he′) derived from carbamate 1h demonstrate that this method is also suitable for linking more complex building blocks. The yields under standard conditions were somewhat lower (50–60%), but by using a slight excess of boronic acid ester 2c′ (1.4 equiv), the yield of both alcohol 3hc′ and boronic acid ester 4hc′ could be significantly increased in these cases.

**Scheme 5:**
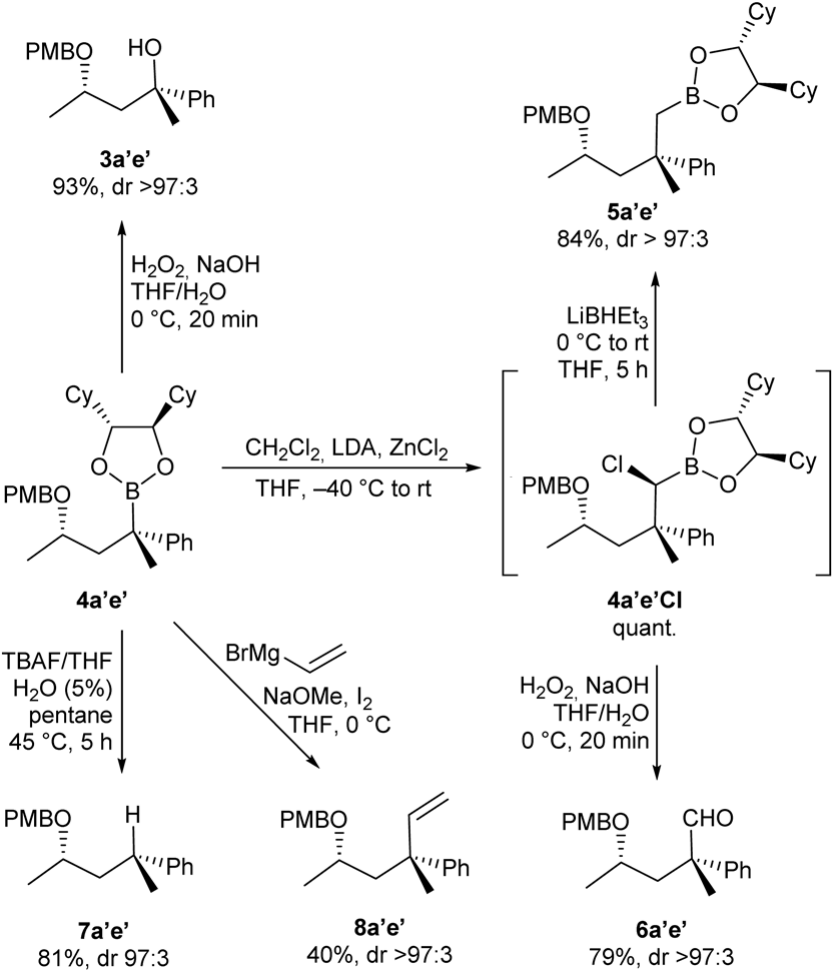
Further modifications of tertiary boronic acid esters.

Further conversions were to be tested based on the isolated boronic acid esters in order to determine the synthetic potential of the tertiary DICHED-boronic acid esters. As an example, boronic acid ester 4a′e′ was subjected to different reaction conditions (Scheme 5). First, a classic Matteson homologation was carried out. ^1^H and ^13^C-NMR confirmed the stereoselective and quantitative conversion to the desired α-chloroboronic acid ester 4a′e′Cl. Its reaction with superhydride yielded the corresponding boronic acid ester 5a′e′, extended by a CH_2_ group, in good yield. Reaction under oxidative conditions yielded the corresponding aldehyde 6a′e′. Identical conditions yielded the already described alcohol 3a′e′ from boronic acid ester 4a′e′, while stereoselective protodeboration^38^ with 1M TBAF solution in pentane made the corresponding deoxygenated compound 7a′e′ accessible. In addition, the terminal alkene 8a′e′ could be obtained from 4a′e′*via* Zweifel olefination.^39^ These examples show that a variety of different substitution/hydroxylation patterns can be generated from complex tertiary boronic acid esters, including quaternary stereogenic centres, which is why this protocol should be excellently suited for the synthesis of natural products and their derivatives.

## Conclusions

In summary, Matteson homologation not only allows the highly stereoselective synthesis of complex chiral boronic acid esters, but also the formation of chiral secondary alcohols through their oxidation. By converting these alcohols into chiral carbamates, they can be coupled with chiral boronic acid esters after deprotonation, whereby tertiary boronic acid esters can be obtained in a highly stereoselective fashion (d.r. ≥ 97 : 3). No matched/mismatched situation is observed, and therefore arbitrarily configured building blocks can be coupled. The tertiary boronic acid esters can be converted into a range of other functionalities, such as alcohols, aldehydes or alkenes, which predestines this method for the synthesis of complex molecules. Applications for this protocol in natural product syntheses are currently under investigation.

## Conflicts of interest

There are no conflicts to declare.

## Supplementary Material

RA-OLF-D6RA03391A-s001

## Data Availability

Supplementary information (SI): copies of ^1^H and ^13^C NMR spectra and experimental details. See DOI: https://doi.org/10.1039/d6ra03391a.
