# Supplementary material for: Highly stereoselective synthesis of complex molecules via coupling of chiral carbamates with chiral boronic acid esters
Source: RSC Adv. 2026 Jul 4;16(35):36769–76. doi: 10.1039/d6ra03391a (PMC13331684; doi:10.1039/d6ra03391a)
Supplement: RA-016-D6RA03391A-s001 [file RA-016-D6RA03391A-s001.pdf]

Supplementary Information (SI) for RSC Advances.
This journal is © The Royal Society of Chemistry 2026

Highly Stereoselective Synthesis of Complex Molecules via Coupling of Chiral Carbamates with chiral Boronicacid Esters

Max Becker and Uli Kazmaier

Supporting Information

Materials and methods S2
General procedures S3
- Synthesis of benzylic and allylic carbamates **1** S5
- Synthesis of secondary boronic esters **2** S13
- Synthesis of tertiary alcohols **3** S19
- Synthesis and isolation of tertiary boronic ester **4** S31
- Further transformations of **4a’e’** S34

NMR-spectra S37
References S89

Materials and methods

All air- and moisture-sensitive reactions were performed in dried glassware (>100 °C) under a nitrogen atmosphere. Tetrahydrofuran (THF) was dried by distillation over sodium/benzophenone, diisopropylamine (DIPA) and *N,N,N',N'*-tetramethylethane-1,2-diamine (TMEDA) were distilled over CaH_2 and stored under a nitrogen atmosphere before use. All other anhydrous solvents: acetonitrile (ACN), dimethyl sulfoxide (DMSO), dichloromethane (DCM), diethyl ether (Et_2O) were purchased from *Acros Organics*. The solvents: *n*-pentane, diethyl ether, and ethyl acetate were distilled prior to use. $ZnCl_2$ and $MgBr_2$ was flame dried in vacuo at 0.5–1.0 mbar prior to use. Reactions at low temperature (< -100 °C) were stirred using elliptical high magnetic force stirring bars (rare earth) purchased from Avantor. Reactions at and under -100 °C were cooled using an ethanol/$CO_{2(s)}$ bath, otherwise an acetone/$CO_{2(s)}$ bath was employed. For longer reaction times at low temperatures a *Thermo Scientific EK90* immersion cooler was used. Room temperature refers to temperatures between 21–24 °C.

Reactions were monitored by NMR or analytical TLC, which was performed on precoated silica gel plates (*Machery-Nagel*, Polygram Sil G/UV_{254}) and visualized using UV light (254 nm) or a cerium(IV)/ ammonium molybdate solution. The crude products were purified by column chromatography on silica gel (*Machery-Nagel 60* or *60M*, 0.04–0.063 or 0.063–0.2 mm). In some cases, the purification was carried out by reversed-phase flash chromatography using the *Büchi Reveleris Prep Chromatography* system with *Büchi FlashPure Select C18* (30 µm spherical) columns.

^{1}H- and ^{13}C-NMR spectra were recorded on a Bruker Advance II 400 MHz spectrometer (^{1}H: 400 MHz and ^{13}C: 100 MHz) or a Bruker 500 MHz spectrometer (^{1}H: 500 MHz and ^{13}C: 125 MHz). $CDCl_3$ was mixed with the internal standard TMS and used as NMR-solvent. The spectra were analyzed with the *ACD/NMR* Software Processor *Academic Edition* (Version: 12.0) or with the *MestreLab* Mnova NMR Software and the chemical shifts δ are reported in ppm relative to the internal solvent signal ($CDCl_3$: 7.26 ppm, 77.0 ppm) or TMS (0.00 ppm). Data are reported as follows: chemical shift, multiplicity (m = multiplet, s = singlet, br. s = broad singlet, d = doublet, t = triplet, q = quartet, quin = quintet, sext = sextet, etc.). The assignment of the peaks was carried out using (^{1}H,^{1}H)-COSY, (^{1}H,^{13}C)-HSQC and (^{1}H,^{13}C)-HMBC spectra. The α-carbon atoms of the boronic esters could often not be observed in the ^{13}C-NMR spectra.

HPLC was performed with the system *LaChrom D-7000* from *Merck Hitachi* using the chiral column *Chiracel OD-H* (4.6 x 250 mm, particle size 5 µm). The signals were detected with a diode array detector (*LaChrom L-7455*) and the analysis was carried out with the MultiHSM-manager from *Merck Hitachi*. The column was kept at 20 °C using a *Knauer Advanced Scientific Instruments* column oven. A GC-2010 System from Shimadzu (AOC-20i autoinjector, FID-detector) with a CP-Chirasil-Dex CB (Varian, 25 m x 0.25 mm, 0.25 µm internal diameter) with N_2 as carrier gas was used for GC-FID analysis.

High resolution mass spectra were recorded with a Finnigan MAT 95 spectrometer (quadrupole) using the CI technique (CI). Further HRMS were performed using an ESI-Orbitrap Q exactive mass spectrometer, equipped with a heated ESI source and a quadrupole-orbitrap coupled mass detector and an Ultimate3000 HPLC from Thermo Finnigan (ESI-Orbitrap).

Optical rotations were measured on a *A. Krüss Optronic GmbH P8000-T* polarimeter at 20 °C. The measured values are given in 10^{-1} deg cm^2 g^{-1}. The radiation source used was a sodium vapor lamp (λ = 589 nm).

Melting points were determined with a melting point apparatus MEL-TEMP II by Laboratory Devices and are uncorrected.

General procedures

GP1: Synthesis of carbamates

The chiral alcohol (1.0 eq.) was dissolved in ACN (1.0 ml/mmol), diisopropylcarbamoyl chloride (1.25 eq.) and Et_3N (1.3 eq.) was added at room temperature. The reaction mixture was stirred at 90 °C over night (14–16 h). After confirming complete conversion (TLC) the reaction was poured in a separatory funny filled with H_2O and Et_2O. The aqueous phase was extracted with Et_2O (3x) and The combined organic extracts were washed with H_2O, then sat. NaCl and dried over $MgSO_4$. After evaporating the solvent, the crude mixture was purified by flash chromatography, and the product was dried in vacuo.

GP2: Matteson-homologation

For the preparation of carbenoid-solution: In a flame dried Schlenk-flask DCM (2.5 eq.) was dissolved in THF (0.87 ml/mmol) and cooled to -100 – -110 °C (Ethanol/$CO_{2(s)}$). Then a mixture of *n*-BuLi (1.05 eq., 2.5 M in *n*-hexane) and THF (0.64 ml/mmol) was cooled to -78 °C and added dropwise (via transfer cannula for reaction scales > 5.00 mmol). The reaction was stirred for 30 min at -100 – -110 °C.

Homologation: A precooled (-78 °C) solution of boronic ester (1.0 eq.) in THF (1.1 ml/mmol) was added dropwise (transfer cannula usable). After complete addition, the reaction was stirred 30 min at - 100 °C – - 110 °C before dried $ZnCl_2$ (1.05–3.0 eq.) in THF (0.8 ml/mmol $ZnCl_2$) was added. The mixture was stirred 30 min at the aforementioned temperature and then slowly warmed up to room temperature over 4–16 h (*sidenote*: most α-chloro boronic esters will be fully formed after warming up over 3–4 h and stirring 0.5–1 h at room temperature. Longer times resulted out of convenience).

GP3: Workup of α-Cl-boronic esters and boronic esters

The reaction mixture was transferred into a separatory funnel filled with *n*-pentane and sat. NH_4Cl-solution. The phases were separated, and the aqueous phase extracted with *n*-pentane (3x). The combined organic extracts were dried over MgSO4 and the solvent removed under reduced pressure.

GP4: Oxidation of boronic esters

The boronic ester (1.0 eq.) was dissolved in THF (10 ml/mmol), H_2O_2 (5–10 eq., 33% in H_2O) and NaOH (5–10 eq., 0.5 M in H_2O) was added and the mixture was stirred at 0 °C–50 °C for 0.5–2.5 h. Upon completion (monitored by TLC) the reaction mixture was transferred into a separatory funnel containing sat. NaCl and Et_2O. The aqueous phase was extracted with Et_2O (3x). The organic phase was dried over $MgSO_4$ and the solvent evaporated (*sidenote*: for better separation and/or isolation of the chiral auxiliar, 1.1–1.5 eq methylboronic acid can be added while drying over $MgSO_4$ and stirred for 15 min at room temperature).

GP5: Synthesis of tertiary DICHED-boronic esters[1]

In a flame-dried Schlenk-tube the carbamate **1x** (1.0 eq.) was dissolved in Et_2O (4.0 ml/mmol), (1.1 eq. TMEDA added in indicated cases) and under continuous stirring (750–800 U/min) *s*-BuLi (1.03–1.05 eq.) was added dropwise at -78 °C. After 10 min–3.5 h the boronic ester (1.05–1.4 eq.) was added dropwise as a solution in Et_2O (1.0 ml/mmol) at -78 °C. The mixture was stirred at this temperature for 0.5–14 h before $MgBr_2$ (1.0 eq.) in MeOH (1.0 ml/mmol $MgBr_2$) was added. After 15 min at -78 °C the reaction was warmed up to room temperature and stirred for 1–16 h (monitored by ^{1}H-NMR). The reaction mixture was worked up according to **GP3**.

GP5.1: Direct oxidation of the tertiary boronic ester to the tertiary alcohol

The mixture of boronic esters were dissolved in THF (10 ml/mmol), cooled to 0 °C and reacted with H_2O_2 (5.0–10 eq. in relation to carbamate, 33% in H_2O) and NaOH (5.0–10 eq., in relation to carbamate, 0.5 M in H_2O) at 0 °C–50 °C and stirred until complete conversion (monitored by TLC). The reaction mixture was transferred into a separatory funnel filled with Et_2O and sat. NaCl-solution. The phases were separated, and the aqueous phase extracted trice with Et_2O. The combined organic extracts were dried over MgSO4 and the solvent removed under reduced pressure. (*sidenote*: for better separation and/or isolation of the chiral auxiliar, 1.1–1.5 eq methylboronic acid can be added while drying over $MgSO_4$ and stirred for 15 min at room temperature).

Synthesis of benzylic and allylic carbamates 1

(*S*)-1-Phenylethyldiisopropylcarbamate (1a)

According to **GP1** 696 mg (5.70 mmol, 1.0 eq.) commercially available (*S*)-1-phenylethan-1-ol were reacted with 1.17 g (5.30 mmol, 1.25 eq.) *N,N*-diisopropylcarbamoyl chloride and 1.03 ml (7.41 mmol, 1.3 eq., ρ = 0.73 g/ml). The obtained residue was purified by flash chromatography (SiO_2, 60M, *n*-pentane/EtOAc 95:5). **Yield**: 1.32 g (5.30 mmol, 93%); yellowish oil; $\mathbf{R_f}$ = 0.12 (*n*-pentane/EtOAc 95:5); $[\boldsymbol{\alpha}]_{\boldsymbol{D}}^{\mathbf{20}}$ = -6.7 ($CHCl_3$, c = 1.00); Lit.:[1] $[\alpha]_D^{22}$ = -7.0 ($CHCl_3$, c = 1.00); **^{1}H-NMR** (400 MHz, $CDCl_3$): δ = 1.16–1.26 (br. s, 12 H), 1.55 (d, *J* = 6.6 Hz, 3 H), 3.70–4.16 (br. s, 2 H), 5.78 (q, *J* = 6.6 Hz, 1 H), 7.24–7.29 (m, 1 H, 1-H), 7.45–7.48 (m, 4 H); **^{13}C-NMR** (100 MHz, $CDCl_3$): δ = 20.8–21.5 (br. s), 22.9, 72.7, 126.0, 127.4, 128.4, 142.8, 155.1; **HRMS** (CI) calcd. for $C_{15}H_{24}NO_2$ $[M+H]^+$: 250.1802, found: 250.1804; analytical data are in accordance with literature[1].

(*R*)-1-Phenylethyldiisopropylcarbamate (1a')

According to **GP1** 2.00 g (16.4 mmol, 1.0 eq.) commercially available (*R*)-1-phenylethan-1-ol were reacted with 3.35 g (20.5 mmol, 1.25 eq.) *N,N*-diisopropylcarbamoyl chloride and 2.97 ml (7.41 mmol, 1.3 eq., ρ = 0.73 g/ml) Et_3N. After workup the obtained residue was purified by flash chromatography (SiO_2, *60M*, *n*-pentane/EtOAc 95:5). **Yield**: 3.65 g (14.6 mmol, 89%); yellowish oil; $\mathbf{R_f}$ = 0.12 (*n*-pentane/EtOAc 95:5); $[\boldsymbol{\alpha}]_{\boldsymbol{D}}^{\mathbf{20}}$ = +6.5 ($CHCl_3$, c = 1.00); Lit.:[2] $[\alpha]_D^{22}$ = +7.0 ($CHCl_3$, c = 1.00); spectroscopic data identical to enantiomer **1a**.

Chiral GC measurements of (*S*)-1-phenylethan-1-ol and (*R*)-1-phenylethan-1-ol used for the synthesis of **1a** and **1a'**.

Method for shown measurements: *Chirasil-Dex CB*, 60 °C [5 min]–220 °C [5 min], 5 °C/min

Racemic mixture: Phenylethan-1-ol

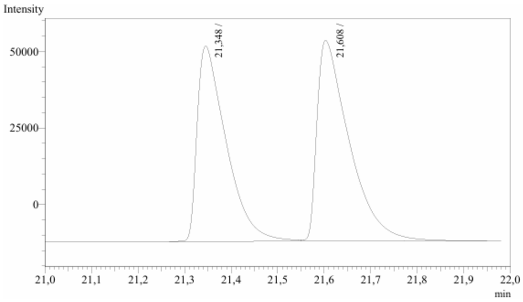

Peak#	Ret.Time	Area	Height	Conc.	Unit	Mark	ID#	Cmpd Name
1	21,348	275780	56499	46,660				
2	21,608	315257	63544	53,340		V		
Total		591037	120043					

(*S*)-1-phenylethan-1-ol for the synthesis of **1a**:

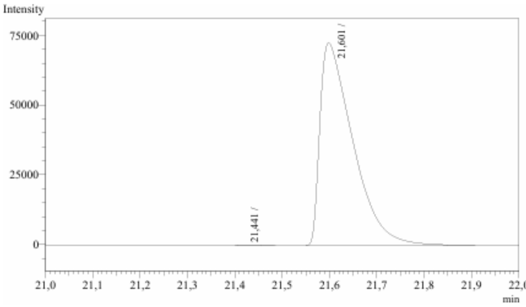

Peak#	Ret.Time	Area	Height	Conc.	Unit	Mark	ID#	Cmpd Name
1	21,441	727	279	0,206				
2	21,601	352983	68203	99,794				
Total		353710	68482					

(*R*)-1-phenylethan-1-ol for the synthesis of **1a'**:

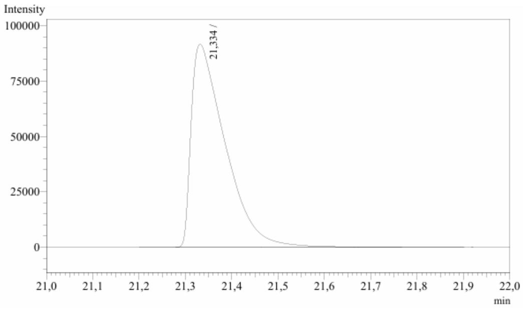

Peak#	Ret.Time	Area	Height	Conc.	Unit	Mark	ID#	Cmpd Name
1	21,334	467083	86225	100,000				
Total		467083	86225					

(*R*)-1-(4-Chlorophenyl)ethyldiisopropylcarbamat (1b’)

According to **GP1** 529 mg (3.38 mmol, 1.0 eq.) commercially available (*R*)-1-(4-chlorphenyl)ethan-1-ol were reacted with 636 mg (3.89 mmol, 1.15 eq.) *N,N*-diisopropylcarbamoyl chloride and 565 µL (4.05 mmol, 1.2 eq., ρ = 0.73 g/ml) Et_3N. After workup, the obtained residue was purified by flash chromatography (SiO_2, *60M*, *n*-pentane/EtOAc 9:1). **Yield**: 737 mg (2.91 mmol, 86%); white solid; $\mathbf{R_f}$ = 0.16 (*n*-pentane/EtOAc 9:1); $[\alpha]_D^{20}$ = +2.5 ($CHCl_3$, c = 1.00); **melting point**: 55–56 °C; ^{1}**H-NMR** (500 MHz, $CDCl_3$): δ = 1.16–1.26 (br. s, 12 H), 1.53 (d, *J* = 6.6 Hz, 3 H), 3.73–4.10 (br. s, 2 H), 5.80 (q, *J* = 6.6 Hz, 1 H), 7.28–7.32 (m, 4 H); ^{13}**C-NMR** (100 MHz, $CDCl_3$): δ = 20.6–21.6 (br. s), 22.7, 45.2–46.5 (br. s), 72.0, 127.4, 128.6, 133.1, 141.4, 154.9; **HRMS** (CI) calcd. for $C_{15}H_{23}ClNO_2$ $[M+H]^+$: 284.1412, found: 284.1425.

(*S*)-1-(4-Methoxyphenyl)ethyldiisopropylcarbamate (1c)

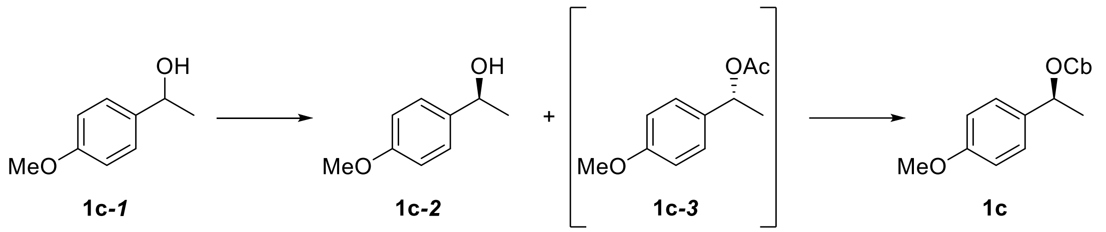

1-(4-Methoxyphenyl)ethan-1-ol **(1c-*1*)**

3.00 g (20.0 mmol, 1.0 eq.) 1-(4-methoxyphenyl)ethan-1-one were dissolved in MeOH (0.6 M) and reacted with 1.13 g (30.0 mmol, 1.5 eq.) $NaBH_4$. After confirming complete conversion (TLC) the reaction was worked up according to **GP3** using EtOAc instead of *n*-pentane. The obtained residue was purified by flash chromatography (SiO_2, *60M*, *n*-pentane/EtOAc 9:1). **Yield**: 2.89 g (16.1 mmol, 94 %); colorless liquid; $\mathbf{R_f}$ = 0.06 (*n*-pentane/EtOAc 9:1); ^{1}**H-NMR** (400 MHz, $CDCl_3$): δ = 1.48 (d, *J* = 6.5 Hz, 3 H), 3.80 (s, 3 H), 4.86 (q, *J* = 6.5 Hz, 1 H), 6.87–6.90 (m, 2 H), 7.29–7.32 (m, 2 H); ^{13}**C-NMR** (100 MHz, $CDCl_3$): δ = 25.0, 55.3, 70.0, 113.8, 126.6, 138.0, 159.0; **HRMS** (CI) calcd. for $C_9H_{13}O_2$ $[M+H]^+$: 153.0910, found: 153.0885.

(*S*)-1-(4-methoxyphenyl)ethan-1-ol (**1c-*2***) and (*R*)-1-(4-methoxyphenyl)ethyl acetate (**1c-*3***)

2.15 g (14.1 mmol, 1.0 eq.) 1-(4-Methoxyphenyl)ethan-1-ol (**1c-*1***) were dissolved in 32 ml vinyl acetate and reacted with novozyme 435 (15 mg/mmol) for 4 h. After reaching 51% conversion (monitored by ^{1}H-NMR) the reaction was stopped by filtering off the enzyme. After workup the obtained residue was purified by flash chromatography (SiO_2, *60M*, *n*-pentane/EtOAc 9:1).
Yield (**1c-*2***): 1.04 g (6.83 mmol, 48 %, *er* >99:1); colorless liquid; $\mathbf{R_f}$ = 0.06 (*n*-pentane/EtOAc; 9:1); $[\alpha]_D^{20}$ = -50.6 [$CHCl_3$, c = 1.00]; Lit.:[1] $[\alpha]_D^{20}$ = -50.0 ($CHCl_3$, c = 1.00); spectroscopic data identical to 1-(4-Methoxyphenyl)ethan-1-ol.

Yield (**1c-*3***): 1.14 g (7.14 mmol, 50 %), colorless liquid, $\mathbf{R_f}$ = 0.28 (*n*-pentane/EtOAc 9:1), $[\alpha]_D^{20}$ = +130.6 [$CHCl_3$, c = 1.00]; ^{1}**H-NMR** (400 MHz, $CDCl_3$): δ = 1.52 (d, *J* = 6.6 Hz, 3 H), 2.05 (s, 3 H), 3.80 (s, 3 H), 5.85 (q, *J* = 6.6 Hz, 1 H), 6.86–6.90 (m, 2 H), 7.28–7.31 (m, 2 H);^{13}**C-NMR** (100 MHz, $CDCl_3$): δ = 21.4, 21.9, 55.3, 72.0, 113.8, 127.6, 133.7, 170.4, 159.2; **HRMS** (CI) calcd. for $C_9H_{11}O$ $[M+H-H_3CCOOH]^+$: 135.0804, found: 135.0800.

Chiral HPLC measurement of 1-(4-Methoxyphenyl)ethan-1-ol (**1c-*1***) and (*S*)-1-(4-Methoxy-phenyl)ethan-1-ol (**1c-*2***)

Method: *OD-H*, Hex:iPrOH, 99:1, 1 ml/min

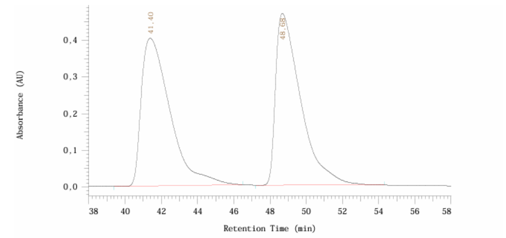

Peak Quantitation: AREA
Calculation Method: AREA%
Peak Quantitation: AREA

No.	RT	Area	Area %
1	41.40	22252729	49.755
2	48.68	22471664	50.245
		44724393	100.000

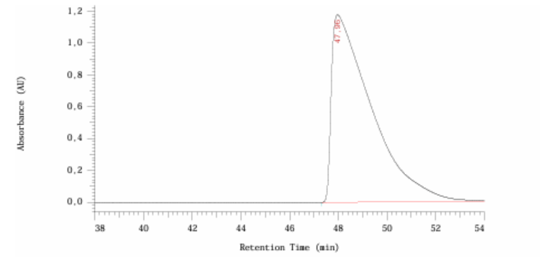

Peak Quantitation: AREA
Calculation Method: AREA%
Peak Quantitation: AREA

No.	RT	Area	Area %
1	47.96	67145580	100.000
		67145580	100.000

(*S*)-1-(4-Methoxyphenyl)ethyldiisopropylcarbamate (1c)

According to **GP1** 673 mg (4.42 mmol, 1.0 eq.) (*S*)-1-(4-methoxyphenyl)ethan-1-ol (**1c-*2***) were reacted with 905 mg (5.53 mmol, 1.25 eq.) *N,N*-diisopropylcarbamoyl chloride and 801 µL (5.75 mmol, 1.3 eq.) Et_3N. The obtained residue was purified by flash chromatography (SiO_2, *60M*, *n*-pentane/EtOAc 9:1). **Yield**: 947 mg (3.39 mmol, 77%); colorless liquid; $\mathbf{R_f}$ = 0.11 (*n*-pentane/EtOAc 95:5); $[\alpha]_D^{20}$ = –11.7 [$CHCl_3$, c = 1.00]; Lit.:[1] $[\alpha]_D^{21}$ = -14.3 (CH_2Cl_2, c = 1.12), **^{1}H-NMR** (400 MHz, $CDCl_3$): δ = 1.17–1.20 (m, 12 H), 1.54 (d, *J* = 6.6 Hz, 3 H), 3.75–4.12 (br. s, 2 H), 3.80 (s, 3 H), 5.80 (q, *J* = 6.6 Hz, 1 H), 6.86–6.89 (m, 2 H), 7.29–7.31 (m, 2 H); **^{13}C-NMR** (100 MHz, $CDCl_3$): δ = 20.6–21.4 (br. s), 22.7, 45.2–46.1 (br. s) , 55.2, 72.3,113.7, 127.4, 134.9, 155.2, 158.9; **HRMS** (CI) calcd. for $C_{16}H_{26}NO_3$ $[M+H]^+$: 280.1907, found: 280.1896; analytical data are in accordance with literature[1]

(*S*)-1-(3-Methoxyphenyl)ethyl diisopropylcarbamate (1d)

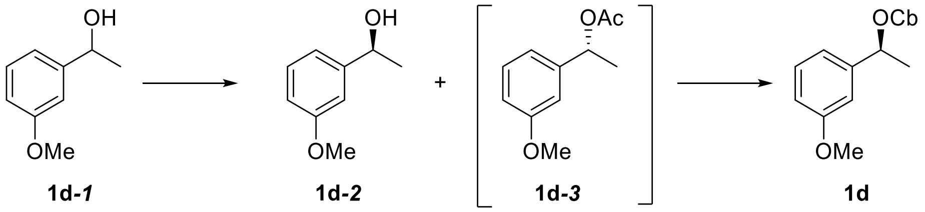

(*S*)-1-(3-Methoxyphenyl)ethan-1-ol (**1d-*2***) and (*R*)-1-(3-Methoxyphenyl)ethyl acetate (**1d-*3***)

2.01 g (13.2 mmol, 1.0 eq.) commercially available 1-(3-methoxyphenyl)ethan-1-ol (**1d-*1***) were dissolved in 32 mL vinyl acetate and reacted with novozyme 435 (15 mg/mmol) for 25 h. After reaching 51% conversion (monitored by ^{1}H-NMR) the reaction was stopped by filtering off the enzyme. After workup the obtained residue was purified by flash chromatography (SiO_2, *60M*, *n*-pentane/EtOAc 95:5). **Yield** (**1d-*2***): 955 mg (6.27 mmol, 48 %, *er* >99:1); colorless liquid; $\mathbf{R_f}$ = 0.16 (*n*-pentane/EtOAc; 85:15); $[\alpha]_D^{20}$ = -44.5 [$CHCl_3$, c = 1.00]; **^{1}H-NMR** (400 MHz, $CDCl_3$): δ = 1.49 (d, *J* = 6.4 Hz, 3 H), 3.82 (s, 3 H), 4.88 (q, *J* = 6.4 Hz, 1 H), 6.80–6.83 (m, 1 H), 6.94–6.96 (m, 2 H, 5-H), 7.25–7.29 (m, 1 H); **^{13}C-NMR** (100 MHz, $CDCl_3$): δ = 25.1, 55.2, 70.4, 110.9, 112.9, 117.7, 129.5, 147.6, 159.8; **HRMS** (CI) calcd. for $C_9H_{11}O$ $[M+H-H_2O]^+$: 135.0804, found: 135.0802.

Yield (**1d-*3***): 1.33 g (6.86 mmol, 52 %), colorless liquid, $\mathbf{R_f}$ = 0.41 (*n*-pentane/EtOAc 85:15), $[\alpha]_D^{20}$ = +82.5 [$CHCl_3$, c = 1.00]; **^{1}H-NMR** (400 MHz, $CDCl_3$): δ = 1.52 (d, *J* = 6.6 Hz, 3 H), 2.08 (s, 3 H), 3.85 (s, 3 H), 5.58

(q, J = 6.6 Hz, 1 H), 6.82–6.83 (m, 1 H), 6.89–6.90 (m, 1 H), 6.93– 6.94 (m, 1 H), 7.27 (t, J = 7.9 Hz, 1 H); **^{13}C-NMR** (100 MHz, $CDCl_3$): δ = 21.4, 22.2, 55.2, 72.2, 111.9, 113.0, 118.3, 129.5, 143.3, 159.6, 170.3; **HRMS** (CI) calcd. for $C_{11}H_{16}O_3$ $[M+H]^+$: 195.1016, found: 195.1012.

Chiral GC measurement of 1-(3-Methoxyphenyl)ethan-1-ol (**1d-*1***) and (*S*)-1-(3-Methoxyphenyl)ethan-1-ol (**1d-*21***)
Method: *Chirasil-Dex CB*, 60 °C [5 min] – 220 °C [5 min], 5 °C/min

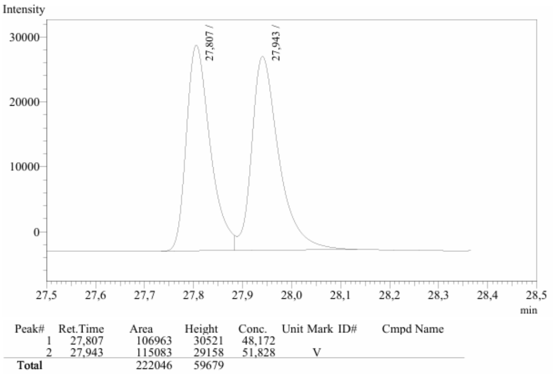

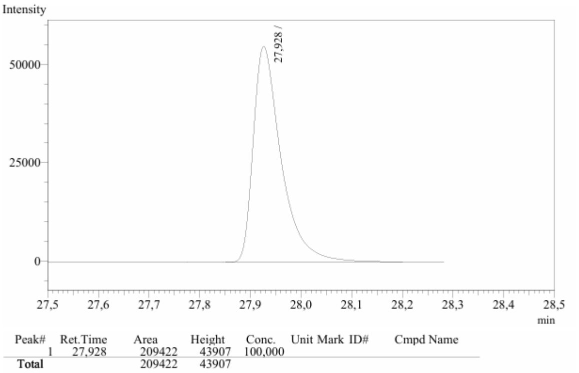

(*S*)-1-(3-Methoxyphenyl)ethyl diisopropylcarbamate (**1d**)

According to **GP1** 927 mg (6.09 mmol, 1.0 eq.) (*S*)-1-(3-Methoxyphenyl)ethan-1-ol (**1d-*2***) were reacted with 1.25 g (7.62 mmol, 1.25 eq.) *N,N*-diisopropylcarbamoyl chloride and 1.10 ml (7.92 mmol, 1.3 eq.) Et_3N. After workup the obtained residue was purified by flash chromatography (SiO_2, *60M*, *n*-pentane/EtOAc 95:5). **Yield**: 1.45 g (5.19 mmol, 85%); colorless liquid; **R_f** = 0.11 (*n*-pentane/EtOAc 95:5); **$[\alpha]_D^{20}$** = -2.8 [$CHCl_3$, c = 1.00]; **^{1}H-NMR** (400 MHz, $CDCl_3$): δ = 1.21–1.23 (br. s, 12 H), 1.54 (d, J = 6.6 Hz, 3 H), 3.80–4.10 (br. s, 2 H), 3.80 (s, 3 H), 5.81 (q, J = 6.6 Hz, 1 H), 6.79–6.82 (m, 1 H), 6.90–6.91 (m, 1 H), 6.93–6.96 (m, 1 H), 7.26 (t, J = 7.9 Hz, 1 H); **^{13}C-NMR** (100 MHz, $CDCl_3$): δ = 20.6–21.0 (br. s), 22.9, 45.2–46.7 (br. s), 55.1 , 72.6, 111.6, 112.8, 118.3, 129.4, 144.6, 155.0, 159.6; **HRMS** (CI) calcd. for $C_{16}H_{25}NO_3$ $[M]^+$: 279.1834, found: 279.1823.

(*S*)-1-(2-Methoxyphenyl)ethyldiisopropylcarbamate (1e)[3]

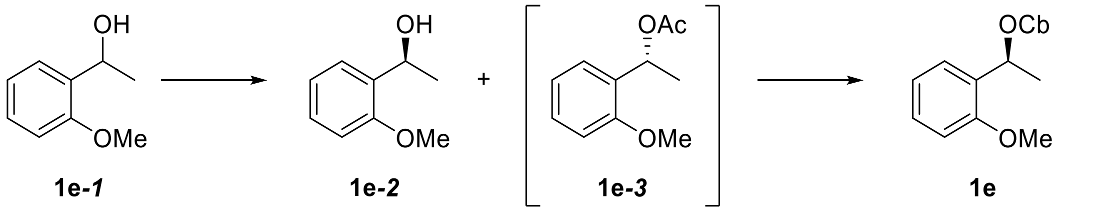

1e-*1* **1e-*2*** **1e-*3*** **1e**

1-(2-Methoxyphenyl)ethan-1-ol (**1e-*1***)

4.31 g (28.7 mmol, 1.0 eq.) 1-(2-Methoxyphenyl)ethan-1-one were dissolved in MeOH (0.6 M) and reacted with 1.63 g (43.0 mmol, 1.5 eq.) $NaBH_4$ at 0 °C. After 1.5 h at 0 °C and full conversion (TLC) the reaction was worked up according to **GP3** using EtOAc instead of *n*-pentane. The solvent was evaporated under reduced pressure and the crude residue was purified by flash chromatography (SiO_2, *60M*, *n*-pentane/EtOAc 8:2). **Yield**: 4.19 g (27.5 mmol, 96 %); colorless liquid; **R_f** = 0.27 (*n*-pentane/EtOAc 8:2); **^{1}H-NMR** (400 MHz, $CDCl_3$): δ = 1.51 (d, J = 6.5 Hz, 3 H), 3.87 (s, 3 H), 5.09 (q, J = 6.5 Hz, 1 H), 6.89 (dd, J = 1.1, 8.0 Hz, 1 H), 6.96 (dt, J = 1.1, 7.6 Hz, 1 H), 7.25 (dt, J = 1.5 Hz, 8.0 Hz, 1 H),

7.34 (dd, 1.8, 7.6 Hz, 1 H); **^{13}C-NMR** (100 MHz, $CDCl_3$): δ = 22.8 , 55.2, 66.6, 110.4, 120.8, 126.1, 128.3, 133.4, 156.6. **HRMS** (CI) calcd. for $C_9H_{12}O_2$ $[M]^+$: 152.0837, found: 152.0837.

(1-(2-Methoxyphenyl)ethan-1-ol (**1e-*2***) and (*R*)-1-(2-Methoxyphenyl)ethyl acetate (**1e-*3***)[3]

2.00 g (13.1 mmol, 1.0 eq.) 1-(2-ethoxyphenyl)ethan-1-ol (**1e-*1***) were dissolved in 26 mL vinyl acetate and reacted with novozyme 435 (15mg/mmol) for 48 h. After reaching 50% conversion (monitored by ^{1}H-NMR) the reaction was stopped by filtering off the enzyme. After workup the obtained residue was purified by flash chromatography (SiO_2, *60M*, *n*-pentane/EtOAc 95:5).

Yield (**1e-*2***): 957 mg (6.29 mmol, 48 %, *er* 97:3); colorless liquid; **R$_f$** = 0.3 (*n*-pentane/EtOAc; 8:2); $[\alpha]_D^{20}$ = -25.0 [$CHCl_3$, c = 1.00].

Yield (**1e-*3***): 1.20 g (6.18 mmol, 47 %), colorless liquid, **R$_f$** = 0.41 (*n*-pentane/EtOAc 8:2), $[\alpha]_D^{20}$ = +67.3 [$CHCl_3$, c = 1.00]; **^{1}H-NMR** (400 MHz, $CDCl_3$): δ = 1.48 (d, *J* = 6.6 Hz, 3 H), 2.09 (s, 3 H), 3.84 (s, 3 H), 6.24 (q, *J* = 6.6 Hz, 1 H), 6.87 (dd, *J* = 0.8, 8.4 Hz, 1 H), 6.96 (dt, *J* = 0.8, 7.5 Hz, 1 H), 7.24–7.27 (m, 1 H), 7.36 (dd, *J* = 1.9, 7.6 Hz, 1 H); **^{13}C-NMR** (100 MHz, $CDCl_3$): δ = 21.1, 21.4, 55.4, 67.2, 110.5, 120.5, 125.8, 128.6), 130.4, 155.9, 170.2; **HRMS** (CI) calcd. for $C_9H_{11}O$ $[M-CH_3COOH+H]^+$: 135.0804, found: 135.0801.

Chiral GC measurement of racemate and (*S*)-1-(2-Methoxyphenyl)ethan-1-ol (**1e-*2***)
Method: *Chirasil-Dex CB*, 60 °C [5 min] – 220°C [5 min], 5°C/min

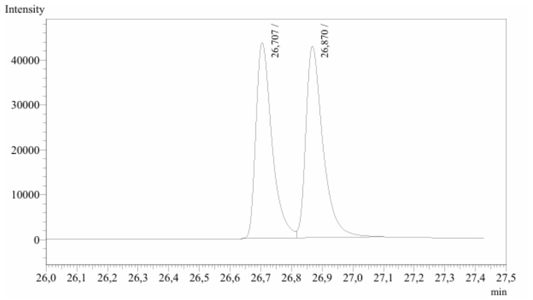

Peak#	Ret.Time	Area	Height	Conc.	Unit	Mark	ID#	Cmpd Name
1	26,707	157967	41856	48,980				
2	26,870	164544	39919	51,020		V		
Total		322511	81775					

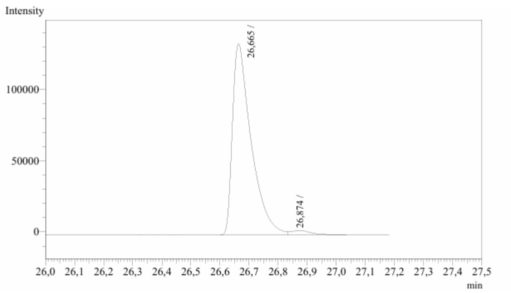

Peak#	Ret.Time	Area	Height	Conc.	Unit	Mark	ID#	Cmpd Name
1	26,665	599667	121197	97,667				
2	26,874	14324	3027	2,333		V		
Total		613991	124224					

(*S*)-1-(2-Methoxyphenyl)ethyldiisopropylcarbamate (1e)[3]

According to **GP1** 937 mg (6.15 mmol, 1.0 eq.) (*S*)-1-(2-Methoxyphenyl)ethan-1-ol were reacted with 1.26 g (7.69 mmol, 1.25 eq.) *N,N*-diisopropylcarbamoyl chloride and 1.12 ml (8.00 mmol, 1.3 eq.) Et_3N. After workup the obtained residue was purified by flash chromatography (SiO_2, *60M*, *n*-pentane/EtOAc 95:5). **Yield**: 1.20 g (4.30 mmol, 70%); colorless liquid; **R$_f$** = 0.11 (*n*-pentane/EtOAc 95:5); $[\alpha]_D^{20}$ = +22.2 [$CHCl_3$, c = 1.00]; Lit.[3] $[\alpha]_D^{20}$ = +19.8 (c 6, CH_2Cl_2); **^{1}H-NMR** (400 MHz, $CDCl_3$): δ = 1.21–1.24 (br. s, 12 H), 1.54 (d, *J* = 6.5 Hz, 3 H), 3.75–4.10 (br. s, 2 H), 3.83 (s, 3 H), 6.91 (q, *J* = 6.5 Hz, 1 H), 6.86 (dd, *J* = 1.0, 8.0 Hz, 1 H), 6.95 (dt, *J* = 1.0, 7.6 Hz, 1 H), 7.23 (dt, *J* = 1.8, 8.0 Hz, 1 H), 7.35 (dd, *J* = 1.8, 7.6 Hz, 1 H); **^{13}C-NMR** (100 MHz, $CDCl_3$): δ = 20.8–21.2 (br. s), 21.8, 44.1–46.4 (br. s), 55.3, 67.8, 110.4, 120.4, 125.9, 128.1, 131.7, 155.0, 155.9; **HRMS** (CI) calcd. for $C_{16}H_{26}NO_3$ $[M+H]^+$: 280.1907, found: 280.1915; analytical data are in accordance with literature[3].

(*S*,*E*)-Pent-3-en-2-yl diisopropylcarbamat (1f)[4]

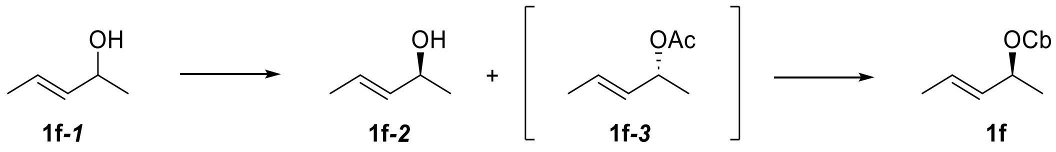

(*S*,*E*)-Pent-3-en-2-ol (**1f-*2***) and (*R*,*E*)-Pent-3-en-2-yl acetate (**1f-*3***)[4]

3.00 g (34.8 mmol, 1.0 eq.) commercially available (*E*)-Pent-3-en-2-ol (**1f-*1***) (with <5% *Z*-Isomer) were dissolved in 30 ml *n*-pentane. 16.1 ml (15.0 mmol, 5.0 eq., ρ = 0.93 g/ml) vinyl acetate and 348 mg Novozym 435 were added and reacted for 3.5 h. After reaching 56% conversion (monitored by ^{1}H-NMR) the reaction was stopped by filtering off the enzyme. The solvent was carefully evaporated by rotary evaporator using a small Vigreux column (8 cm) instead of the foam brake. The residue was purified by flash chromatography (SiO_2, *60M*, *n*-pentane/Et_2O 9:1). *Products are strongly volatile!* Separation by distillation instead of column chromatography failed.

Yield (**1f-*1***): 3.50 g (17.8 mmol, 51%, *er* >99:1, purity 65%, impurity: *n*-pentane); colorless liquid; **R$_f$** = 0.11 (*n*-pentane/Et_2O 8:2); the alcohol was used in the following reaction without further purification; **^{1}H-NMR** (400 MHz, $CDCl_3$): δ = 1.25 (d, *J* = 6.4 Hz, 3 H), 1.69 (dd, *J* = 1.6 Hz, 6.4 Hz 3 H), 4.26 (p, *J* = 6.4 Hz, 1 H), 5.44 (qdd, *J* = 1.5, 6.7, 15.3 Hz, 1 H), 5.66 (dq, *J* = 6.4, 15.3 Hz, 1 H); **^{13}C-NMR** (100 MHz, $CDCl_3$): δ = 17.6, 23.3, 70.5, 127.0, 133.0.

Yield (**1f-*2***): 1.61 g (14.0 mmol, 40%, purity: 75%, impurity: *n*-pentane), colorless liquid, **R$_f$** = 0.46 (*n*-pentane/Et_2O 8:2). **^{1}H-NMR** (400 MHz, $CDCl_3$): δ = 1.29 (d, 3 H), 1.69 (dd, 3 H), 4.26 (p, 1 H), 5.44 (qdd, 1 H), 5.66 (dqd, 1 H). **^{13}C-NMR** (100 MHz, $CDCl_3$): δ = 17.6, 20.3, 23.3, 73.3, 128.9, 130.7, 170.4.

Chiral GC measurement of (*E*)-Pent-3-en-2-ol (**1f-*1***) and (*S*,*E*)-Pent-3-en-2-ol (**1f-*2***.
Method: *Chirasil-Dex CB*, 60 °C [5 min] – 220 °C [5 min], 5 °C/min

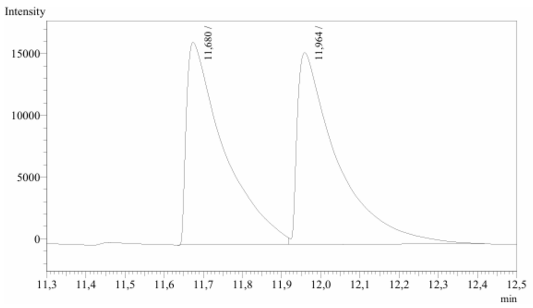

Peak#	Ret.Time	Area	Height	Conc.	Unit	Mark	ID#	Cmpd Name
1	11,680	109317	14791	49,771				
2	11,964	110322	14171	50,229		V		
Total		219639	28962					

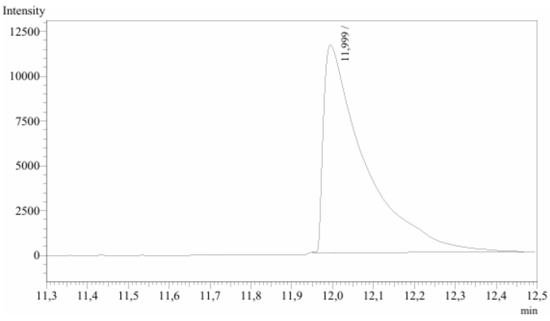

Peak#	Ret.Time	Area	Height	Conc.	Unit	Mark	ID#	Cmpd Name
1	11,999	81292	10721	100,000				
Total		81292	10721					

(*S*,*E*)-Pent-3-en-2-yl diisopropylcarbamat (1f)[4]

1.54 g (11.9 mmol, 1.0 eq., purity 65%, impurity: *n*-pentane) (*S*,*E*)-Pent-3-en-2-ol (**1f-*2***) were dissolved in 17.9 ml THF, 3.02 g NaH (17.9 mmol, 1.5 eq., 60% in mineral oil) were added and the reaction mixture stirred for 30 min at room temperature. Afterwards 58.0 mg (477 µmol, 4.0 mol%) DMAP and 2.54 g (15.5 mmol, 1.3 eq.) *N,N*-diisopropylcarbamoyl chloride were added and the reaction mixture stirred for 17 h under reflux. After completion (monitored by TLC) the reaction worked up analogous to **GP1**. The residue was purified by flash chromatography (SiO_2, *60M*, *n*-pentane/Et_2O 9:1). Yield: 2.05 g (9.13 mmol, 77%); colorless liquid; R$_f$ = 0.2 (*n*-pentane/Et_2O 9:1); $[\alpha]_D^{20}$ = +14.5 [$CHCl_3$, c = 1.00]; Lit.[4] $[\alpha]_D^{22}$ = +11.6 ($CHCl_3$, c = 1.2); **^{1}H-NMR** (400 MHz, $CDCl_3$): δ = 1.20 (d, *J* = 6.8 Hz, 12 H), 1.30 (d, *J* = 6.5 Hz, 3 H), 1.69 (d, *J* = 6.5 Hz, 3 H), 3.74–4.10 (br. s, 2 H), 5.26 (quin, *J* = 6.5 Hz, 1 H), 5.52 (ddd, *J* = 1.5, 6.5, 15.3 Hz, 1 H), 5.52 (qd, *J* = 6.5, 15.3 Hz, 1 H); **^{13}C-NMR** (100 MHz, $CDCl_3$): δ = 17.7, 20.8, 21.0 (br. s), 45.2 (br. s), 71.1, 126.6, 131.8, 155.3; **HRMS** (ESI-Orbitrap) calcd. for $C_{12}H_{24}NO_2$ $[M+H]^+$: 214.1802, found 214.1792; analytical data are in accordance with literature[4].

(*S*,*E*)-Hex-4-en-3-yl diisopropylcarbamate (1g)[4]

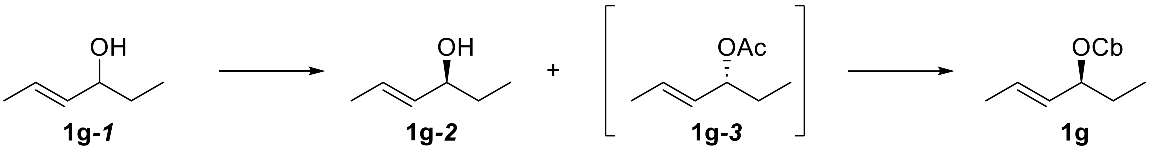

(*E*)-Hex-4-en-3-ol (**1g-*1***)

2.45 g (35.0 mmol, 1.0 eq.) *(E)*-But-2-enal were dissolved in 58.3 ml Et_2O and dropwise reacted with 12.3 ml (36.8 mmol, 1.05 eq. 3.0 M in Et_2O) EtMgBr at 0 °C and stirred for 2 h at this temperature. After completion (monitored by TLC) the reaction was quenched with H_2O and diluted with additional 20 ml Et_2O. After separation the aqueous phase was extracted with Et_2O (2x 25 ml) and the organic phase was dried over $MgSO_4$. Afterwards the solvent was evaporated by fractional distillation (Vigreux-column, oil-bath temperature: 45–50 °C). Yield: 3.91 g (18.0 mmol, 51%, purity: 46%, impurity: *n*-pentane). The residue was used in the following reaction without any further purification. **^{1}H-NMR** (400 MHz, $CDCl_3$): δ = 0.90 (t, *J* = 7.4 Hz, 3 H), 1.53 (dqd, *J* = 6.8, 7.4, 13.6 Hz, 3 H), 1.70 (dd, *J* = 1.6, 6.4 Hz, 3 H), 3.96 (q, *J* = 6.8 Hz, 1 H), 5.47 (qdd, *J* = 1.6, 6.8, 15.2 Hz, 1 H), 5.65 (dqd, *J* = 0.9, 6.4, 15.2 Hz, 1 H); **^{13}C-NMR** (100 MHz, $CDCl_3$): δ = 9.75, 17.7, 30.1, 74.5, 127.0, 134.0.

(*S*,*E*)-Hex-4-en-3-ol (**1g-*2***) and (*R*,*E*)-Hex-4-en-3-yl acetate (**1g-*3***)[4]

2.00 g (9.19 mmol, 1.0 eq. purity 46%, impurity: *n*-pentane) (*E*)-Hex-4-en-3-ol were dissolved in 8.5 ml *n*-pentane. 931 ml (10.1 mmol, 1.1 eq., ρ = 0.93 g/ml) vinyl acetate and 91.9 mg novozyme 435 were added and reacted for 3.5 h. The reaction was stopped after reaching 62% conversion (monitored by ^{1}H-NMR) by filtering off the enzyme. The solvent was carefully evaporated by rotary evaporator using a small Vigreux column (8 cm) instead of the foam brake. The residue was purified by flash chromatography (SiO_2, *60M*, *n*-pentane/Et_2O 9:1). *Products are strongly volatile!*

Yield (**1g-*2***): 1.11 g (3.55 mmol, 39%, *er* >99:1, purity 32%, impurity: *n*-pentane); colorless liquid; **R_f** = 0.14 (*n*-pentane/Et_2O 9:1); the alcohol was used in the following reaction without further purification.

Yield (**1g-*3***): 1.09 g (4.22 mmol, 46%, purity: 55%, impurity: *n*-pentane), colorless liquid, **R_f** = 0.49 (*n*-pentane/Et_2O 9:1; **^{1}H-NMR** (500 MHz, $CDCl_3$): δ = 0.88 (t, *J* = 7.4 Hz, 3 H), 1.57 (dqd, *J* = 6.5, 7.4, 13.9 Hz, 2 H), 1.64 (dqd, *J* = 6.7, 7.5, 13.9 Hz, 2 H), 1.70 (dd, *J* = 1.7, 6.5 Hz, 3 H), 2.04 (s, 3 H), 5.11 (q, *J* = 6.9 Hz, 1 H), 5.40 (qdd, *J* = 1.7, 7.5, 15.3 Hz, 1 H), 5.72 (dqd, *J* = 1.0, 6.5, 15.3 Hz, 1 H); **^{13}C-NMR** (125 MHz, $CDCl_3$): δ = 9.55, 14.1, 17.7, 34.1, 76.2, 129.3, 129.4, 170.5.

Chiral GC measurement of (*E*)-Hex-4-en-3-ol (**1g-*1***) and (*S*,*E*)-Hex-4-en-3-ol (**1g-*2***).
Method: *Chirasil-Dex CB*, 60 °C [5 min] – 220 °C [5 min], 5 °C/min

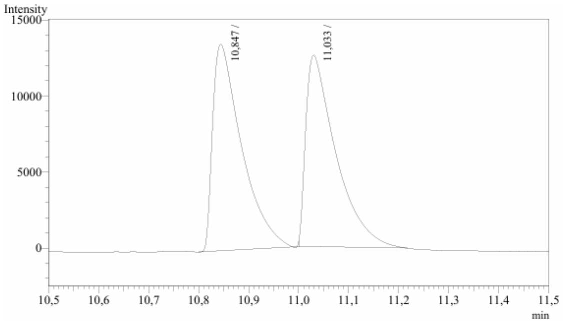

Peak#	Ret.Time	Area	Height	Conc.	Unit	Mark	ID#	Cmpd Name
1	10,847	54617	11718	50,986				
2	11,033	52505	11275	49,014				
Total		107122	22993					

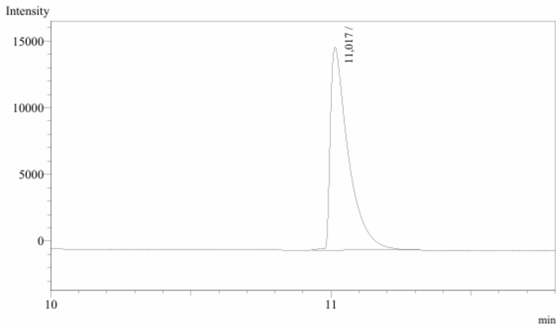

Peak#	Ret.Time	Area	Height	Conc.	Unit	Mark	ID#	Cmpd Name
1	11,017	67760	13843	100,000				
Total		67760	13843					

(S,E)-Hex-4-en-3-yl diisopropylcarbamate (1g)

1.10 g (3.54 mmol, 1.0 eq., purity 32%, impurity: *n*-pentane) (S,E)-Pent-3-en-2-ol (**1g-*2***) were dissolved in 5.31 ml THF, 537 mg NaH (5.31 mmol, 1.5 eq., 60% in mineral oil) were added and the reaction mixture stirred for 30 min at room temperature. Afterwards 17.3 mg (142 µmol, 4.0 mol%) DMAP and 753.1 mg (4.60 mmol, 1.3 eq.) *N,N*-diisopropylcarbamoyl chloride were added and the reaction mixture stirred for 16 h under reflux. After completion (monitored by TLC) the reaction worked up analogous to **GP1**. The residue was purified by flash chromatography (SiO_2, *60M*, *n*-pentane/Et_2O 9:1). 715 mg (3.14 mmol, 89%); colorless liquid; R_f = 0.27 (*n*-pentane/Et_2O 9:1); $[\alpha]_D^{20}$ = +24.6 [$CHCl_3$, c = 1.00]; **^{1}H-NMR** (400 MHz, $CDCl_3$): δ = 0.90 (t, *J* = 7.4 Hz, 3 H), 1.21 (d, *J* = 6.8 Hz, 12 H), 1.54–1.68 (m, 2 H), 1.70 (dd, *J* = 1.7, 6.5 Hz, 3 H), 3.75–4.10 (br. s, 2 H), 5.08 (q, *J* = 6.7 Hz, 1 H), 5.43 (qdd, *J* = 1.7, 7.1, 15.5 Hz, 1 H), 5.69 (qd, *J* = 6.5, 15.5 Hz, 1 H); **^{13}C-NMR** (100 MHz, $CDCl_3$): δ = 9.68, 17.8, 21.9 (br. s), 28.0, 45.7 (br. s), 76.3 , 127.8, 130.4, 155.5; **HRMS** (ESI-Orbitrap) calcd. for $C_{13}H_{26}NO_2$ $[M+H]^+$:228.1958, found 228.1951; analytical data are in accordance with literature[4].

(1S,3S)-3-Methyl-1-phenylhexyl diisopropylcarbamate (1h)

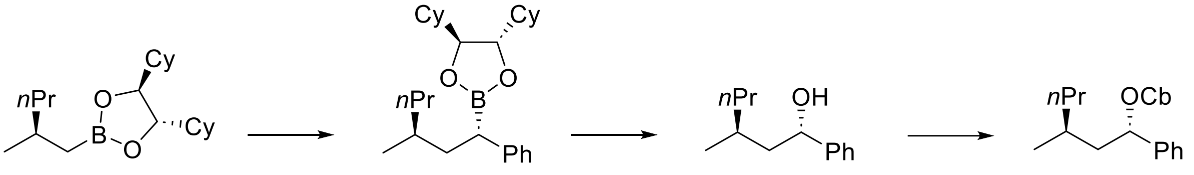

2b **1h-*1*** **1h-*2*** **1h**

(4S,5S)-4,5-Dicyclohexyl-2-((1S,3S)-3-methyl-1-phenylhexyl)-1,3,2-dioxaborolane (**1h-*1***)

According to **GP2** 1.68 g (5.24 mmol, 1.0 eq.) **2b** were reacted with 843 µL (13.1 mmol, 2.5 eq., ρ = 1.32 g/ml) DCM, 2.20 ml (5.50 mmol, 1.05 eq., 2.5 M in *n*-hexane) *n*-BuLi and 750 mg (5.50 mmol, 1.05 eq.) $ZnCl_2$. After 16 h and confirming full conversion the reaction mixture was cooled to - 78 °C and 3.14 ml (9.43 mmol, 1.8 eq., 3.0 M in THF) phenylmagnesium bromide was added dropwise. The reaction was slowly warmed up to room temperature and stirred for 24 h. After confirming full conversion (^{1}H-NMR) and workup according to **GP3**, the crude product was purified by flash chromatography (SiO_2, *60*, *n*-pentane/Et_2O 99:1). **Yield**: 1.88 g (4.58 mmol, 87%), colorless oil; **R_f** = 0.15 (*n*-pentane /Et_2O 99:1); $[\alpha]_D^{20}$ = -9.0 [$CHCl_3$, c = 1.00]; **^{1}H-NMR** (400 MHz, $CDCl_3$): δ = 0.80–0.89 (m, 2 H), 0.81 (t, *J* = 6.8 Hz, 3 H), 0.85 (d, *J* = 6.4 Hz, 3 H), 0.92–1.04 (m, 2 H), 1.05–1.19 (m, 7 H), 1.20–1.33 (m, 7 H), 1.48–1.52 (m, 2 H), 1.57–1.81 (m, 9 H), 2.49 (dd, *J* = 6.8 Hz, 9.5 Hz, 1 H), 3.80–3.82 (m, 2 H), 7.09–7.13 (m, 1 H), 7.19–7.23 (m, 4 H); **^{13}C-NMR** (100 MHz, $CDCl_3$): δ = 14.3, 19.1, 20.0, 25.9, 26.0, 26.4, 27.3, 28.2, 29.2*, 31.0, 39.1, 39.7, 42.9, 83.4, 125.0, 128.1, 128.4, 143.5;*determined with HMBC/HSQC; **HRMS (CI)** calcd. for $C_{27}H_{43}BO_2$ $[M]^+$: 410.3356, found: 410.3378.

(1S,3S)-3-Methyl-1-phenylhexan-1-ol (**1h-*2***)

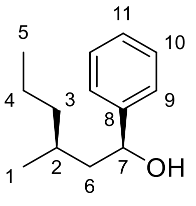

According to **GP4** 1.74 g (4.24 mmol, 1.0 eq.) of the freshly prepared boronic acid ester **1h-*1*** were oxidized with 1.99 ml (21.2 mmol, 5.0 eq. 33% in H_2O) H_2O_2 and 848 mg (21.2 mmol, 5.0 eq.) NaOH at

0 °C for 30 min. After workup the crude product was purified by flash chromatography (SiO_2, *60M*, *n*-pentane/Et_2O 9:1). **Yield**: 713 mg (3.71 mmol, 88%, *dr* 97:3); colorless oil, **R_f** = 0.1 (*n*-pentane/Et_2O 9:1); $[\alpha]_D^{20}$ = -36.1 [$CHCl_3$, c = 1.00];

^{1}H-NMR (400 MHz, $CDCl_3$): δ = 0.85 (t, $^3J_{5,4}$ = 7.1 Hz, 3 H, 5-H), 0.93 (t, $^3J_{1,2}$ = 6.6 Hz, 3 H, 1-H), 1.07–1.15 (m, 1 H, 3-H_a), 1.20–1.40 (m, 3 H, 3-H_b, 4-H), 1.42–1.50 (m, 1 H, 2-H), 1.67 (t, $^3J_{6,7}$ = $^3J_{6,2}$ = 7.0 Hz, 2 H, 6-H), 1.75 (br.s, 1 H, OH), 4.76 (t, $^3J_{7,6}$ = 6.6 Hz, 1 H, 7-H), 7.25–7.30 (m, 1 H, 11-H), 7.32–7.37 (m, 4 H, 9-H, 10-H); **^{13}C-NMR** (100 MHz, $CDCl_3$): δ = 14.3 (C-5), 19.8 (C-4), 20.1 (C-1), 29.4 (C-2), 39.0 (C-3), 46.5 (C-6), 72.9 (C-7), 126.0 (C-9), 127.5 (C-11), 128.5 (C-10), 145.0 (C-8); **HRMS** (CI) calcd. for $C_{13}H_{20}O$ $[M]^+$: 192.1514, found: 192.1504

(1*S*,3*S*)-3-Methyl-1-phenylhexyl diisopropylcarbamate (1h)

According to **GP1** 645 mg (3.36 mmol, 1.0 eq.) secondary alcohol **1h-*2*** were dissolved in ACN and reacted with 687 mg (4.20 mmol, 1.25 eq.) *N,N*-diisopropylcarbamoyl chloride and 608 µL (4.36 mmol, 1.3 eq., ρ = 0.73 g/ml) Et_3N. After workup the obtained residue was purified by flash chromatography (SiO_2, *60M*, *n*-pentane/Et_2O 95:5). **Yield**: 974 mg (3.05 mmol, 91%); colorless oil which solidifies at -20 °C; **R_f** = 0.24 (*n*-pentane/Et_2O 95:5); $[\alpha]_D^{20}$ = -2.0 [$CHCl_3$, c = 1.00]; **^{1}H-NMR** (500 MHz, $CDCl_3$): δ = 0.82 (t, *J* = 7.3 Hz, 3 H), 0.93 (d, *J* = 6.6 Hz, 3 H), 1.07–1.35 (m, 17 H), 1.75 (ddd, *J* = 6.1, 7.4, 13.5 Hz, 1 H), 1.75 (td, 7.4, 13.5 Hz, 1 H), 3.80–4.05 (br. s, 2 H), 5.68 (t, *J* = 6.9 Hz), 5.78 (t, *J* = 7.4 Hz, 1 H), 7.24–7.27 (m, 1 H), 7.31–7.36 (m, 4 H); **^{13}C-NMR** (125 MHz, $CDCl_3$): δ = 14.2, 19.8, 19.8, 20.8–21.6 (br. s), 29.1, 39.1, 44.0, 45.3–46.1 (br. s), 75.2, 126.7, 127.4, 128.3, 141.9, 155.1; **HRMS** (CI) calcd. for $C_{22}H_{34}NO_2$ $[M+H]^+$: 320.2584, found: 320.2578.

Synthesis of secondary boronic esters 2

(4*S*,5*S*)-4,5-Dicyclohexyl-2-((*S*)-pentan-2-yl)-1,3,2-dioxaborolane (2a)

According to **GP2** 2.40 g (9.58 mmol, 1.0 eq.) (4*S*,5*S*)-4,5-dicyclohexyl-2-methyl-1,3,2-dioxaborolan[5] were reacted with 1.54 ml (24.0 mmol, 2.5 eq., ρ = 1.32 g/ml) DCM, 4.02 ml (10.1 mmol, 1.05 eq., 2.5 M in *n*-hexane) *n*-BuLi and 1.44 g (10.1 mmol, 1.1 eq.) $ZnCl_2$. After 16 h and confirming full conversion the reaction mixture was cooled to 0 °C and 11.5 ml (22.0 mmol, 2.4 eq., 2.0 M in Et_2O) *n*-propyl magnesium chloride were added dropwise. After confirming full conversion (^{1}H-NMR) and workup according to **GP3**, the crude product was purified by flash chromatography (SiO_2, *60*, *n*-pentane/Et_2O 99:1). **Yield**: 2.26 g (7.38 mmol, 77%), colorless oil; **R_f** = 0.3 (*n*-pentane /Et_2O 99:1); $[\alpha]_D^{20}$ -56.6 [$CHCl_3$, c = 1.00]; **^{1}H-NMR** (400 MHz, $CDCl_3$): δ = 0.88 (t, *J* = 7.1 Hz, 3 H), 0.90–0.99 (m, 2 H), 0.98 (d, *J* = 7.1 Hz, 3 H), 1.02–1.13(m, 3 H), 1.13–1.25 (m, 6 H), 1.25–1.38 (m, 5 H), 1.41–1.48 (m, 1 H), 1.57–1.60 (m, 2 H), 1.67–1.69 (m, 2 H), 1.75–1.78 (m, 6 H), 3.82–3.84 (m, 2 H); **^{13}C-NMR** (100 MHz, $CDCl_3$): δ = 14.3, 15.7, 21.9, 25.9, 26.0, 26.5, 27.4, 28.3, 35.6, 43.1, 83.1; **HRMS (CI)** calcd. for $C_{19}H_{35}BO_2$ $[M]^+$: 307.2803, found: 307.2840.

(4*S*,5*S*)-4,5-Dicyclohexyl-2-((*R*)-2-methylpentyl)-1,3,2-dioxaborolane (2b)

According to **GP2** 1.94 g (6.33 mmol, 1.0 eq.) **2a** were reacted with 1.02 ml (15.8 mmol, 2.5 eq., ρ = 1.32 g/ml) DCM, 2.66 ml (6.65 mmol, 1.05 eq., 2.5 M in *n*-hexane) *n*-BuLi and 906 mg (6.65 mmol,

1.05 eq.) $ZnCl_2$. After 16 h and confirming full conversion the reaction mixture was cooled to 0 °C and 7.60 ml (7.60 mmol, 1.2 eq., 1.0 M in THF) Super-hydride® were added dropwise. The reaction was warmed up to room temperature over 6 h. After confirming full conversion (^{1}H-NMR) and workup according to **GP3**, the crude product was purified by flash chromatography (SiO_2, *60*, *n*-pentane/Et_2O 99:1). **Yield**: 1.84 g (5.70 mmol, 90%), colorless oil; **R$_f$** = 0.21 (*n*-pentane /Et_2O 99:1); $[\alpha]_D^{20}$ -56.2 [$CHCl_3$, c = 1.00]; **^{1}H-NMR** (400 MHz, $CDCl_3$): δ = 0.70 (dd, *J* = 7.7, 15.9 Hz, 1 H), 0.81–0.85 (m, 1 H), 0.87 (t, *J* = 7.0 Hz, 3 H), 0.91 (d, *J* = 6.6 Hz, 3 H), 0.94–1.00 (m, 2 H), 1.02–1.10 (m, 2 H), 1.15–1.36 (m, 12 H), 1.57–1.61 (m, 2 H), 1.65–1.70 (m, 3 H), 1.74–1.80 (m, 6 H), 3.81–3.84 (m, 2 H); **^{13}C-NMR** (100 MHz, $CDCl_3$): δ = 14.3, 20.5, 22.4, 25.9, 26.0, 26.5, 27.5, 28.4, 29.2, 41.8, 43.1, 83.2; **HRMS (CI)** calcd. for $C_{20}H_{37}BO_2$ $[M]^+$: 320.2887, found: 320.2919.

(4*R*,5*R*)-4,5-dicyclohexyl-2-((*S*)-2-methylpentyl)-1,3,2-dioxaborolane (**2b'**) was synthesized analogous to **2b**. Optical rotation: $[\alpha]_D^{20}$ = +55.8 [$CHCl_3$, c = 1.00].

(4*S*,5S)-4,5-Dicyclohexyl-2-((*S*)-1-((4-methoxybenzyl)oxy)ethyl)-1,3,2-dioxaborolane (2c)

Preparation of the nucleophile: 830 mg (20.8 mmol, 1.3 eq., 60% dispersion in mineral oil) NaH were added to 6.94 ml THF and 25.0 ml DMSO. To the resulting suspension 2.81 ml (22.4 mmol, 1.4 eq., ρ = 1.10 g/ml) 4-methoxyphenyl methanol were added at room temperature and stirred for 5 h.
According to **GP2** 4.00 g (16.0 mmol, 1.0 eq.) (4*S*,5*S*)-4,5-Dicyclohexyl-2-methyl-1,3,2-dioxaborolan[5] was reacted with 2.57 ml (40.0 mmol, 2.5 eq., ρ = 1.32 g/ml) DCM, 6.72 ml (16.8 mmol, 1.05 eq., 2.5 M in *n*-hexane) *n*-BuLi und 2.29 g (16.8 mmol, 1.05 eq.) $ZnCl_2$. After 16 h and confirming full conversion the reaction mixture was cooled to 0 °C and the nucleophile was added dropwise. The reaction mixture was warmed up to room temperature over 17 h and after confirming full conversion (^{1}H-NMR) worked up according to **GP3**. The crude product was purified by flash chromatography (SiO_2, *60*, *n*-pentan/Et_2O 95:5 → 9:1). **Yield**: 4.65 g (11.6 mmol, 73%), colorless oil; **R$_f$** = 0.21 (*n*-pentane /Et_2O 9:1); $[\alpha]_D^{20}$ = -24.3 [$CHCl_3$, c = 1.00]; **^{1}H-NMR** (400 MHz, $CDCl_3$): δ = 0.89–1.01 (m, 2 H), 1.01–1.11 (m, 2 H), 1.13–1.27 (m, 6 H), 1.31 (d, *J* = 7.6 Hz, 3 H), 1.32–1.39 (m, 2 H), 1.56–1.67 (m, 2 H), 1.66–1.70 (m, 2 H), 1.74–1.79 (m, 6 H), 3.43 (q, *J* = 7.6 Hz, 1 H), 3.80 (s, 3 H), 3.90–3.93 (m, 2 H), 4.47 (d, *J* = 11.5 Hz, 1 H), 4.51 (d, *J* = 11.5 Hz, 1 H), 6.84–6.88 (m, 2 H), 7.28–7.30 (m, 2 H). **^{13}C-NMR** (100 MHz, $CDCl_3$): δ = 16.9, 25.9, 26.0, 26.4, 27.3, 28.1, 42.9, 55.2, 71.2, 83.6, 113.6, 129.4, 131.2, 159.0; **HRMS** (CI): fast decomposition at room temperature.

(4*R*,5*R*)-4,5-Dicyclohexyl-2-((*R*)-1-((4-methoxybenzyl)oxy)ethyl)-1,3,2-dioxaborolane (**2c'**) synthesis analogous to synthesis of (**2c**);[5] $[\alpha]_D^{20}$ = +23.3 [$CHCl_3$, c = 1.00]; spectroscopic data identical to enantiomer **2c**; analytical data are in accordance with literature[5]

(4*S*,5*S*)-4,5-Dicyclohexyl-2-((*S*)-1-methoxyethyl)-1,3,2-dioxaborolane (2d)

Preparation of the nucleophile: 592 µL (14.6 mmol, 1.48 eq., ρ = 0.792 g/ml) MeOH were dissolved in 21.9 ml THF and reacted dropwise with 5.52 ml (13.8 mmol, 1.4 eq., 2.5 M in *n*-hexane) *n*-BuLi at -20 °C. The mixture was then stirred for 30 min at room temperature.
According to **GP2** 2.48 g (9.91 mmol, 1.0 eq.) (4*S*,5*S*)-4,5-Dicyclohexyl-2-methyl-1,3,2-dioxaborolan[5] were reacted with 1.59 ml (24.8 mmol, 2.5 eq., ρ = 1.32 g/ml) DCM, 4.16 ml (10.4 mmol, 1.05 eq., 2.5 M in *n*-hexane) *n*-BuLi and 1.49 g (10.9 mmol, 1.1 eq.) $ZnCl_2$. After 16 h and confirming full conversion the reaction mixture was cooled to 0 °C and the nucleophile was added dropwise. The reaction mixture was stirred for 8 h and after confirming full conversion (^{1}H-NMR) the reaction was worked up according to **GP3**. The crude product was purified by flash chromatography (SiO_2, *60*, *n*-

pentane/Et_2O 85:15). **Yield**: 2.36 g (8.03 mmol, 81%), colorless oil; **R$_f$** = 0.36 (*n*-pentane /Et_2O 8:2); **$[\alpha]_D^{20}$** = - 48.2 [$CHCl_3$, c = 1.00]; **^{1}H-NMR** (400 MHz, $CDCl_3$): δ = 0.91–1.02 (m, 2 H), 1.05–1.13 (m, 2 H), 1.15–1.27 (m, 6 H), 1.29 (d, *J* = 7.5 Hz, 3 H), 1.33–1.40 (m, 2 H), 1.58–1.61 (m, 2 H), 1.66–1.69 (m, 2 H), 1.75–1.78 (m, 6 H), 3.29 (q, *J* = 7.5 Hz, 3 H), 3.35 (s, 3 H), 3.91–3.94 (m, 2 H); **^{13}C-NMR** (100 MHz, $CDCl_3$): δ = 15.9, 25.9, 26.0, 26.4, 27.3, 28.1, 42.8, 57.6, 83.7; **HRMS** (CI) calcd. for $C_{17}H_{32}BO_3$ $[M+H]^+$: 295.2439, found: 295.2437.

The enantiomer (4*R*,5*R*)-4,5-Dicyclohexyl-2-((*R*)-1-methoxyethyl)-1,3,2-dioxaborolane (**2d'**) was prepared accordingly. **$[\alpha]_D^{20}$** = +51.3 [$CHCl_3$, c = 1.00]; spectroscopic data identical to enantiomer **2d**.

(4*R*,5*R*)-4,5-Dicyclohexyl-2-((*S*)-2-((4-methoxybenzyl)oxy)propyl)-1,3,2-dioxaborolane (2e')

According to **GP2** 2.54 g (6.35 mmol, 1.0 eq.) **2c'** were reacted with 1.59 ml (24.8 mmol, 2.5 eq., ρ = 1.32 g/ml) DCM, 4.16 ml (10.4 mmol, 1.05 eq., 2.5 M in *n*-hexane) *n*-BuLi and 1.49 g (10.9 mmol, 1.1 eq.) $ZnCl_2$. After 16 h upon confirming full conversion (^{1}H-NMR) the reaction was worked up according to **GP3**. The crude α-chloro boronic ester was dried in vacuo and directly used in the following reactions. **Yield**: 2.85 g (6.35 mmol, 100%); colorless, cloudy oil.
1.45 g (3.10 mmol, 1.0 eq.) crude α-chloro boronic ester and 444 mg (3.25 mmol, 1.05 eq.) $ZnCl_2$ were dissolved in 11.5 ml THF and reacted dropwise with 3.72 ml (3.72 mmol, 1.2 eq., 1.0 M in THF) Super-hydride® at 0 °C. The mixture was warmed up to room temperature over 16 h. Upon completion (^{1}H-NMR), workup of the reaction followed according **GP3**. The crude product was purified by flash chromatography (SiO_2, *60*, *n*-pentane /Et_2O 9:1). **Yield**: 904 mg (2.18 mmol, 70%); colorless oil; **R$_f$** = 0.21 (n-pentane/Et_2O 9:1); **$[\alpha]_D^{20}$** = +55.6 [$CHCl_3$, c = 1.00]; **^{1}H-NMR** (400 MHz, $CDCl_3$): δ = 0.93–0.99 (m, 2 H), 0.99–1.12 (m, 4 H), 1.14–1.36 (m, 6 H), 1.25 (d, *J* = 6.0 Hz, 3 H), 1.28–1.35 (m, 2 H), 1.57–1.60 (m, 2 H), 1.65–1.68 (m, 2 H), 1.73–1.78 (m, 6 H), 3.76–3.81 (m, 1 H), 3.79 (s, 3 H), 3.83–3.84 (m, 2 H), 4.44 (s, 2 H), 6.85 (d, *J* = 8.6 Hz, 1 H), 7.26 (d, *J* = 8.6 Hz, 1 H); **^{13}C-NMR** (100 MHz, $CDCl_3$): δ = 22.1, 25.9, 26.0, 26.4, 27.4, 28.3, 43.0, 55.3, 69.7, 72.5, 83.3, 113.6, 129.0, 131.4, 158.9; **HRMS** (CI) calcd. for $C_{25}H_{39}BO_4$ $[M]^+$: 414.2941, found: 414.2961

(4*R*,5*R*)-4,5-Dicyclohexyl-2-((2*S*,3*S*)-3-((4-methoxybenzyl)oxy)butan-2-yl)-1,3,2-dioxaborolane (2f')

According to **GP2** 2.54 g (6.35 mmol, 1.0 eq.) **2c'** were reacted with 1.59 ml (24.8 mmol, 2.5 eq., ρ = 1.32 g/ml) DCM, 4.16 ml (10.4 mmol, 1.05 eq., 2.5 M in *n*-hexane) *n*-BuLi and 1.49 g (10.9 mmol, 1.1 eq.) $ZnCl_2$. After 16 h upon confirming full conversion (^{1}H-NMR) the reaction was worked up according to **GP3**. The crude α-chloroboronic ester was dried in vacuo and directly used in the following reactions. Yield: 2.85 g (6.35 mmol, 100%); colorless, cloudy oil.
1.40 g (2.99 mmol, 1.0 eq.) crude α-chloro boronic ester and 837 mg (6.14 mmol, 2.05 eq.) $ZnCl_2$ were dissolved in 11.1 ml THF and reacted dropwise with 3.72 ml (9.29 mmol, 3.1 eq., 3.0 M in THF) methyl magnesium chloride at 0 °C. The mixture was warmed up to room temperature over 20 h. Upon completion (^{1}H-NMR), workup of the reaction followed according **GP3**. The crude product was purified by flash chromatography (SiO_2, *60*, *n*-pentane /Et_2O 95:5). **Yield**: 904 mg (2.18 mmol, 70%); colorless oil; **R$_f$** = 0.14 (*n*-pentane/Et_2O 95:5); **$[\alpha]_D^{20}$** = +37.4 [$CHCl_3$, c = 1.00]; **^{1}H-NMR** (400 MHz, $CDCl_3$): δ = 0.89–0.99 (m, 2 H), 0.99 (d, *J* = 7.5 Hz, 3 H), 1.00–1.10 (m, 2 H), 1.15–1.23 (m, 6 H), 1.17 (d, *J* = 6.1 Hz, 3 H), 1.27–1.34 (m, 2 H), 1.50 (dq, *J* = 6.1, 7.5 Hz, 1 H), 1.57–1.60 (m, 2 H), 1.64–1.67 (m, 2 H), 1.72–1.78 (m, 6 H), 3.67 (quin, *J* = 6.1 Hz, 1 H), 3.79 (s, 3 H), 3.81–3.84 (m, 2 H), 4.41 (d, *J* = 11.4 Hz, 1 H), 4.45 (d, *J* =

11.2 Hz, 1 H), 6.85 (d, J = 8.6 Hz, 1 H), 7.26 (d, J = 8.6 Hz, 1 H); **^{13}C-NMR** (100 MHz, $CDCl_3$): δ = 10.4, 18.0, 25.9, 26.0, 26.4, 27.5, 28.3, 43.0, 55.3, 69.7, 76.9, 83.2, 113.6, 128.8, 131.5, 158.8; **HRMS** (CI) calcd. for $C_{26}H_{41}BO_4$ $[M]^+$: 428.3098, found: 428.3085.

Synthesis of (4S,5S)-4,5-dicyclohexyl-2-((2R,3R)-3-((4-methoxybenzyl)oxy)butan-2-yl)-1,3,2-dioxaborolane (**2f**) was performed analogous without isolation of the α-chloro boronic ester. $[\alpha]_D^{20}$ = -35.7 [$CHCl_3$, c = 1.00]; spectroscopic data identical to enantiomer **2f'**.

(4*S*,5*S*)-4,5-Dicyclohexyl-2-((*R*)-1-((4-methoxybenzyl)oxy)hex-5-en-3-yl)-1,3,2-dioxaborolane (2g)

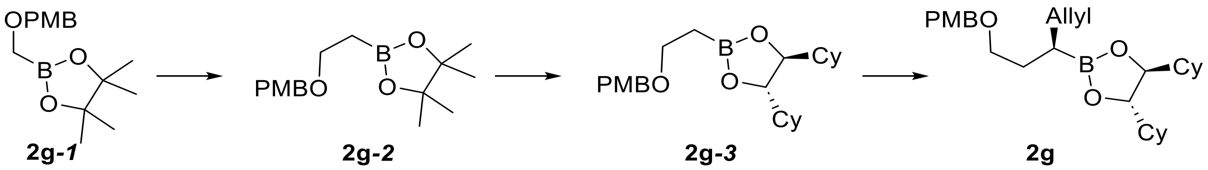

2-(((4-Methoxybenzyl)oxy)methyl)-4,4,5,5-tetramethyl-1,3,2-dioxaborolane (**2g-*1***)

Preparation of the nucleophile: 470 mg (11.8 mmol, 1.3 eq., 60% dispersion in mineral oil) NaH were added to 6.56 ml THF and 23.6 ml DMSO. To the resulting suspension 1.35 ml (10.9 mmol, 1.2 eq., ρ = 1.11 g/ml) (4-Methoxyphenyl)methanol was added at room temperature and stirred for 16 h.
2.00 g (9.05 mmol, 1.0 eq.) commercially available 2-(Bromomethyl)-4,4,5,5-tetramethyl-1,3,2-dioxaborolane were dissolved in 12.0 ml THF and cooled to 0 °C before the nucleophile was added dropwise. The reaction mixture was warmed up to room temperature over 24 h. After complete conversion (monitored by TLC) the reaction was transferred to a separating funnel containing sat. NH_4Cl-solution. The aqueous phase was extracted with *n*-pentane (3x) and the organic phase was dried over $MgSO_4$. The solvent was evaporated using a rotary evaporator and the crude product was purified by flash chromatography (SiO_2, *60*, *n*-pentane /Et_2O 7:3) and due to its inferior stability directly used in the following reaction. **Yield**: 1.61 g (5.10 mmol, 56%, purity: 86%, impurity *n*-pentane); colorless liquid; **R_f** = 0.10 (*n*-pentane /Et_2O 7:3); **^{1}H-NMR** (500 MHz, $CDCl_3$): δ = 1.27 (s, 12 H), 3.24 (s, 2 H), 3.80 (s, 3 H), 4.45 (s, 2 H), 6.87 (d, J = 8.7 Hz, 2 H), 7.26 (d, J = 8.7 Hz, 2 H). **^{13}C-NMR** and **HRMS** (CI) could not be measured due to instability of the compound.

2-(2-((4-Methoxybenzyl)oxy)ethyl)-4,4,5,5-tetramethyl-1,3,2-dioxaborolane (**2g-*2***)

Preparation of the LDA-solution: 946 µl (6.64 mmol, 1.35 eq., ρ = 0.71 g/ml) DIPA were dissolved in 990 µL THF and 2.46 ml (6.14 mmol, 1.25 eq., 2.5 M in *n*-hexane) *n*-BuLi was added dropwise at – 40 °C under continuous stirring. After 10 min the reaction was warmed to room temperature and stirred for 20 min prior to use.

1.59 g (4.92 mmol, 1.0 eq., purity: 86%) **2g-*1*** and 949 µl (14.8 mmol, 3.0 eq., ρ = 1.32 g/ml) DCM were dissolved in 6.89 ml THF and the LDA-solution was added dropwise at -40 °C under continuous stirring. After complete addition the reaction was stirred for 10 min at -40 °C before a solution of 2.04 g (15.0 mmol, 3.05 eq.) $ZnCl_2$ in 10.5 mL THF was added. The reaction was slowly warmed to room temperature (2-3 h) and stirred for 2 h at this temperature. Upon confirming complete conversion (^{1}H-NMR) the reaction was worked up according to **GP3**. The crude product was dissolved in 12.3 ml THF. Then 5.90 ml (5.90 mmol, 1.2 eq., 1.0 M in THF) Super-hydride® were added dropwise at 0 °C. The

reaction mixture was stirred additional 2 h at 0 °C before confirming complete conversion and workup (**GP3**). The obtained crude product was purified by flash chromatography (SiO_2, *60*, *n*-pentane/Et_2O 8:2). **Yield**: 900 mg (3.08 mmol, 63%); colorless runny oil; $\mathbf{R_f}$ = 0.14 (*n*-pentane/Et_2O 8:2); **^{1}H-NMR** (400 MHz, $CDCl_3$): δ = 1.19–1.24 (m, 2 H), 1.24 (s, 12 H), 3.60 (t, *J* = 7.9 Hz, 2 H), 3.80 (s, 3 H), 4.43 (s, 2 H), 6.86 (d, *J* = 8.6 Hz, 2 H), 7.26 (d, *J* = 8.6 Hz, 2 H). **^{13}C-NMR** (100 MHz, $CDCl_3$): δ = 24.8, 55.3, 66.7, 72.2, 83.1, 113.7, 129.2, 130.9, 159.0; **HRMS** (ESI-Orbitrap) calcd. for $C_{16}H_{26}BO_4$ $[M+H]^+$: 293.1919, found: 293.1919.

(4S,5S)-4,5-Dicyclohexyl-2-(2-((4-methoxybenzyl)oxy)ethyl)-1,3,2-dioxaborolane (**2g-*3***)

871 mg (2.98 mmol, 1.0 eq.) **2g-*2*** were dissolved in 14.5 ml THF. At room temperature 810 mg (3.58 mmol, 1.2 eq.) (1*S*,2*S*)-1,2-Dicyclohexylethane-1,2-diol and 376 µl H_2O were added. The reaction was stirred for 5 d at room temperature before complete conversion could be detected (TLC). The reaction was then worked up according to **GP3** but using Et_2O instead of *n*-pentane. The crude product was purified by flash chromatography (SiO_2, *60*, *n*-pentane/Et_2O 95:5 → 9:1). **Yield**: 962 mg (2.40 mmol, 81%); faintly yellow oil; $\mathbf{R_f}$ = 0.39 (*n*-pentane/Et_2O 8:2); $[\alpha]_D^{20}$ = -33.3 [$CHCl_3$, c = 1.00]; **^{1}H-NMR** (400 MHz, $CDCl_3$): δ = 0.90–1.00 (m, 2 H), 1.01–1.12 (m, 2 H), 1.13–1.26 (m, 8 H), 1.29–1.34 (m, 2 H), 1.56–1.60 (m, 2 H), 1.66–1.68 (m, 2 H), 1.74–1.79 (m, 6 H), 3.61 (t, *J* = 7.8 Hz, 2 H), 3.80 (s, 3 H), 3.83–3.84 (m, 2 H), 4.44 (s, 2 H), 6.86 (d, *J* = 8.7 Hz, 2 H), 7.26 (d, *J* = 8.7 Hz, 2 H); **^{13}C-NMR** (100 MHz, $CDCl_3$): δ = 25.9, 26.0, 26.4, 27.3, 28.3, 42.9, 55.3, 66.9, 72.3, 83.3, 113.7, 129.2, 130.9, 159.0; **HRMS** (ESI-Orbitrap) calcd. for $C_{24}H_{38}BO_4$ $[M+H]^+$: 401.2858, found: 401.2863.

(4*S*,5*S*)-4,5-Dicyclohexyl-2-((*R*)-1-((4-methoxybenzyl)oxy)hex-5-en-3-yl)-1,3,2-dioxaborolane (2g)

Preparation of the LDA-solution: 418 µl (2.93 mmol, 1.35 eq., ρ = 0.71 g/ml) DIPA were dissolved in 458 µl THF and 1.09 ml (2.72 mmol, 1.25 eq., 2.5 M in *n*-hexane) *n*-BuLi were added dropwise at – 40 °C under continuous stirring. After 10 min the reaction was warmed to room temperature and stirred for 20 min prior to use.

870 mg (2.17 mmol, 1.0 eq.) **2g-*3*** and 419 µl (6.52 mmol, 3.0 eq., ρ = 1.32 g/ml) DCM were dissolved in 3.08 ml THF and the LDA-solution was added dropwise at -40 °C under continuous stirring. After complete addition the reaction was stirred for 10 min at -40°C before a solution of 903 mg (6.63 mmol, 3.05 eq.) $ZnCl_2$ in 5.4 mL THF was added. The reaction was slowly warmed to room temperature (2-3 h) and stirred for 2 h at this temperature. Upon confirming complete conversion (^{1}H-NMR) the reaction was cooled to -78 °C and 5.43 ml (5.43 mmol, 2.5 eq., 1.0 M in Et_2O) allylmagnesium bromide were added dropwise. After 1.5 h at -78 °C and complete conversion (^{1}H-NMR) the reaction was worked up according to **GP3**. The obtained crude product was used in the following reaction without further purification. **Yield**: 957 mg (2.00 mmol, 92%, purity: 95%, impurity: *n*-pentane); yellow oil; $\mathbf{R_f}$ = 0.22 (*n*-pentane/Et_2O 9:1); $[\alpha]_D^{20}$ = -42.2 [$CHCl_3$, c = 1.00]; **^{1}H-NMR** (400 MHz, $CDCl_3$): δ = 0.90–1.00 (m, 4 H), 1.11–1.31 (m, 9 H), 1.54–1.59 (m, 2 H), 1.66–1.68 (m, 2 H), 1.73–1.79 (m, 8 H), 2.14 (dt, *J* =7.0, 14.0 Hz, 1 H), 2.14 (dt, *J* = 7.0, 14.0 Hz, 1 H), 3.46 (t, *J* = 6.8 Hz, 2 H), 3.77–3.78 (m, 2 H), 3.80 (s, 3 H), 4.41 (s, 2 H), 4.94 (dd, *J* = 1.6, 10.6 Hz, 1 H), 4.99 (dd, *J* = 1.6, 17.1 Hz, 1 H), 5.79 (ddt, *J* = 7.0, 10.6, 17.1 Hz, 1 H), 6.86 (d, *J* = 8.5 Hz, 2 H), 7.26 (d, *J* = 8.5 Hz, 2 H); **^{13}C-NMR** (100 MHz, $CDCl_3$): δ = 25.9, 26.0, 26.5, 27.6, 28.5, 30.6, 35.5, 43.0, 55.2, 69.6, 72.5, 83.4, 113.7, 115.1, 129.2, 130.8, 138.5, 159.0; **HRMS** (CI) calcd. for $C_{28}H_{44}BO_4$ $[M]^+$: 454.3254, found: 454.3264.

(4*S*,5*S*)-4,5-Dicyclohexyl-2-((*S*)-2-methyl-3-(trityloxy)propyl)-1,3,2-dioxaborolane (2h)[6]

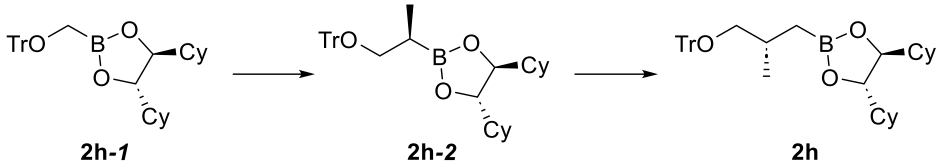

(4*S*,5*S*)-4,5-Dicyclohexyl-2-((trityloxy)methyl)-1,3,2-dioxaborolane (**2h-*1***)

16.0 g (61.5 mmol, 1.0 eq.) Triphenylmethanol were dissolved in 123 ml DMSO and 2.70 g (67.6 mmol, 1.1 eq., 60% in mineral oil) NaH were added at room temperature. After 24 h 13.6 g (61.5 mmol, 1.0 eq.) commercially available 2-(bromomethyl)-4,4,5,5-tetramethyl-1,3,2-dioxaborolane were added dropwise at 0°C and upon complete addition the cooling bath removed. The reaction was stirred for 2 d at room temperature until complete conversion (monitored by ^{1}H-NMR). The reaction mixture was quenched (cautiously!) with sat. NH_4Cl-solution. The aqueous phase was extracted with Et_2O (4x) and the organic phase was dried over $MgSO_4$. After evaporation of the solvent the remaining solid was broken up with mortar and pestle and washed with *n*-pentane. Subsequently the solid was dried in vacuo and dissolved in 215 ml Et_2O. 13.4 g (60.6 mmol, 1.0 eq.) (1*S*,2*S*)-1,2-dicyclohexylethan-1,2-diol and 5.3 ml H_2O were added and the reaction stirred for 3 d at room temperature. After confirming full conversion, the reaction was filtered over $MgSO_4$ and the solvent evaporated. The residue was purified (SiO_2, *60M*, *n*-pentane/Et_2O 95:5 → 9:1). **Yield**: 19.5 g (38.3 mmol, 63%); white solid; **melting point**: 109-110 °C; **R$_f$** = 0.28 (*n*-pentane/Et_2O 95:5); **$[\alpha]_D^{20}$** = -53.5 [$CHCl_3$, c = 1.00]; **^{1}H-NMR** (400 MHz, $CDCl_3$): δ = 0.96–1.12 (m, 4 H), 1.14–1.28 (m, 6 H), 1.35–1.40 (m, 2 H), 1.61–1.64 (m, 2 H), 1.66–1.68 (m, 2 H), 1.76–1.83 (m, 6 H), 2.85 (d, *J* = 15.8 Hz, 1 H), 2.94 (d, *J* = 15.8 Hz, 1 H), 3.92–3.94 (m, 2 H), 7.19–7.22 (m, 3 H), 7.26–7.30 (m, 6 H), 7.46–7.49 (m, 6 H); **^{13}C-NMR** (100 MHz, $CDCl_3$): δ = 25.9, 26.0, 26.5, 27.4, 28.4, 42.9, 83.8, 87.7, 126.7, 127.7, 128.9, 144.2; **HRMS** (ESI-Orbitrap) calcd. for $C_{34}H_{42}BO_3$ $[M+H]^+$: 508.3149, found: 508.3150.

(4*S*,5*S*)-4,5-Dicyclohexyl-2-((*R*)-1-(trityloxy)propan-2-yl)-1,3,2-dioxaborolane (**2h-*2***)[6]

According to **GP2** 9.00 g (17.7 mmol, 1.0 eq.) **2h-*1*** were reacted with 2.85 ml (44.3 mmol, 2.5 eq., ρ = 1.32 g/ml) DCM, 7.44 ml (18.6 mmol, 1.05 eq., 2.5 M in *n*-hexane) *n*-BuLi and 4.95 g (36.3 mmol, 2.05 eq.) $ZnCl_2$. (*sidenote:* this α-chloro boronic ester does tend to precipitate strongly! Continuous, vigorous stirring [>750 U/min] or use of 2.0 ml/mmol instead of 1.1 ml/mmol THF for boronic ester addition is advisable). After slowly warming up to room temperature (4 h) and confirming full conversion the reaction mixture was cooled to 0 °C and 15.3 ml (46.0 mmol, 2.6 eq., 3.0 M in THF) methyl magnesium chloride was added dropwise. The reaction was warmed up to room temperature over 16 h. After confirming full conversion (^{1}H-NMR) and workup according to **GP3**, the crude product was purified by flash chromatography (SiO_2, *60*, *n*-pentane/Et_2O 95:5). **Yield**: 7.39 g (13.8 mmol, 78%), colorless oil; R$_f$ = 0.5 (*n*-pentane /Et_2O 9:1); **$[\alpha]_D^{20}$** = -37.0 [$CHCl_3$, c = 1.00]; **^{1}H-NMR** (400 MHz, $CDCl_3$): δ = 0.90–1.08 (m, 4 H), 1.00 (d, *J* = 7.5 Hz, 3 H), 1.11–1.21 (m, 6 H), 1.28–1.34 (m, 2 H), 1.42–1.51 (m, 1 H), 1.57–1.65 (m, 4 H), 1.69–1.76 (m, 6 H), 3.03 (t, *J* = 7.6, 8.2 Hz, 1 H), 3.17 (dd, *J* = 7.0, 8.0 Hz, 1 H), 3.83–3.86 (m, 2 H), 7.19–7.22 (m, 3 H), 7.26–7.29 (m, 6 H), 7.45–7.47 (m, 6 H); **^{13}C-NMR** (100 MHz, $CDCl_3$): δ = 13.0, 25.9, 26.0, 26.5, 27.4, 28.4, 43.0, 66.6, 83.3, 86.1, 126.7, 127.5, 128.8, 144.6; **HRMS** (ESI-Orbitrap) calcd. for $C_{36}H_{46}BO_3$ $[M+H]^+$: 537.3535, found: 537.3552; analytical data are in accordance with literature[6]

(4*S*,5*S*)-4,5-Dicyclohexyl-2-((*S*)-2-methyl-3-(trityloxy)propyl)-1,3,2-dioxaborolane (2h)[6]

According to **GP2** 5.94 g (10.9 mmol, 1.0 eq.) **2h-*2*** were reacted with 1.75 ml (27.1 mmol, 2.5 eq., ρ = 1.32 g/ml) DCM, 4.56 ml (11.4 mmol, 1.05 eq., 2.5 M in *n*-hexane) *n*-BuLi and 3.03 g (22.2 mmol, 2.05 eq.) $ZnCl_2$. After 16 h, confirming full conversion (^{1}H-NMR) the reaction was worked up according to **GP3**. The α-chloro boronic ester was dissolved in 27.2 ml THF and cooled to 0 °C before 13.0 ml (13.0 mmol, 1.2 eq., 1.0 M in THF) Super-hydride® were added dropwise. The reaction was warmed up to room temperature over 16 h. After confirming full conversion (^{1}H-NMR) and workup according to **GP3**, the crude product was purified by flash chromatography (SiO_2, *60*, *n*-pentane/Et_2O 95:5→ 9:1). Yield: 5.92 g (10.1 mmol, 93%), white solid; **melting point**: 80–81 °C; **R$_f$** = 0.49 (*n*-pentane /Et_2O 9:1); **$[\alpha]_D^{20}$** = -15.6 [$CHCl_3$, c = 1.00]; **^{1}H-NMR** (400 MHz, $CDCl_3$): δ = 0.68 (dd, *J* = 8.8, 15.7 Hz, 1 H), 0.98–1.04 (m, 5 H), 0.99 (d, *J* = 6.5 Hz, 3 H), 1.11–1.28 (m, 8 H),1.54–1.57 (m, 2 H), 1.65–1.67 (m, 2 H), 1.72–1.76 (m, 6 H), 2.08 (sext, *J* = 6.5 Hz, 1 H), 2.81 (dd, *J* = 7.2, 8.4 Hz, 1 H), 2.90 (dd, *J* = 6.2, 8.4 Hz, 1 H), 3.77–3.78 (m, 2 H), 7.19–7.23 (m, 3 H), 7.26–7.29 (m, 6 H), 7.43–7.45 (m, 6 H); **^{13}C-NMR** (100 MHz, $CDCl_3$): δ = 19.9, 25.9, 26.0, 26.5, 27.4, 28.4, 42.9, 65.9, 70.3, 83.3, 86.0, 126.7, 127.6, 128.8, 144.6; **HRMS** (ESI-Orbitrap) calcd. for $C_{37}H_{48}BO_3$ $[M+H]^+$: 550.3691, found: 550.3688. analytical data are in accordance with literature[6]

Synthesis of tertiary alcohols 3

(2*R*,3*S*)-3-Methyl-2-phenylhexan-2-ol (3aa)

According to **GP5** 251 mg (1.01 mmol, 1.0 eq.) **1a** were deprotonated using 739 µl (1.04 mmol, 1.03 eq., 1.4 M in cyclohexane) *s*-BuLi for 20 min and reacted with 323 mg (1.06 mmol, 1.05 eq.) **2a** for 16 h. Then 185 mg (1.01 mmol, 1.0 eq.) $MgBr_2$ (dry) in 1.01 ml methanol were added. The reaction was stirred at room temperature for 1 h. The crude boronic ester was briefly purified by flash chromatography (**R$_f$** = 0.16–0.18, *n*-pentane/Et_2O 99:1) and directly oxidized according to **GP5.1** using 471 µl (5.03 mmol, 5.0 eq. 33% in H_2O) H_2O_2 and 201 mg (5.03 mmol, 5.0 eq., 0.5 M in H_2O) NaOH. The crude product was purified by flash chromatography (SiO_2, *60M*, *n*-pentane/Et_2O 9:1); **Yield**: 164 mg (853 µmol, 85%, *dr* > 97:3); colorless oil; **R$_f$** = 0.11 (*n*-pentane/Et_2O 9:1); **$[\alpha]_D^{20}$** = -9.5 [$CHCl_3$, c = 1.00]; **^{1}H-NMR** (400 MHz, $CDCl_3$): δ = 0.82 (d, *J* = 6.5 Hz, 3 H), 0.88 (t, *J* = 7.1 Hz, 3 H), 0.93–0.99 (m, 1 H), 1.09–1.17 (m, 1 H), 1.37–1.46 (m, 2 H), 1.53 (s, 3 H), 1.64 (br. s, 1 H), 1.77–1.83 (m, 1 H), 7.21–7.25 (m, 1 H), 7.31–7.35 (m, 2 H), 7.41–7.43 (m, 2 H); **^{13}C-NMR** (100 MHz, $CDCl_3$): δ = 14.1, 14.3, 21.0, 26.5, 33.2, 43.6, 76.9*, 125.4, 126.4, 127.8, 147.9; *determined with HSQC/HMBC; **HRMS** (CI) calcd. for $C_{13}H_{20}O$ $[M]^+$: 192.1514, found: 192.1510.

(2*S*,3*S*)-3-Methyl-2-phenylhexan-2-ol (3a'a)

According to **GP5** 250 mg (1.00 mmol, 1.0 eq.) **1a'** were deprotonated using 737 µl (1.03 mmol, 1.03 eq., 1.4 M in cyclohexane) *s*-BuLi for 20 min and reacted with 322 mg (1.05 mmol, 1.05 eq.) **2a** for 14 h. Then 184 mg (1.00 mmol, 1.0 eq.) $MgBr_2$ (dry) in 1.00 ml methanol were added. The reaction was stirred at room temperature for 1 h. The crude boronic ester was briefly purified by flash chromatography (**R$_f$** = 0.17–0.19, *n*-pentane/Et_2O 99:1) and directly oxidized according to **GP5.1** using 469 µl (5.01 mmol, 5.0 eq. 33% in H_2O) H_2O_2 and 200 mg (5.01 mmol, 5.0 eq., 0.5 M in H_2O) NaOH. The crude product was purified by flash chromatography (SiO_2, *60M*, *n*-pentane/Et_2O 9:1); **Yield**: 167 mg (868 µmol, 87%, *dr* > 97:3); colorless oil; **R$_f$** = 0.21 (*n*-pentane/Et_2O 85:15); **$[\alpha]_D^{20}$** = -49.5 [$CHCl_3$, c = 1.00]; **^{1}H-NMR** (400 MHz, $CDCl_3$): δ = 0.82 (t, *J* = 7.1 Hz, 3 H), 0.86 (d, *J* = 6.8 Hz, 3 H), 0.92–0.99 (m, 1

H), 1.10–1.17 (m, 1 H), 1.35–1.42 (m, 2 H), 1.51 (s, 3 H), 1.63 (br. s, 1 H, OH), 1.76–1.82 (m, 1 H), 7.21–7.36 (m, 1 H), 7.31–7.35 (m, 2 H), 7.41–7.43 (m, 2 H); **^{13}C-NMR** (100 MHz, $CDCl_3$): δ = 13.9, 14.2, 21.1, 26.6, 33.4, 43.5, 77.2, 125.2, 126.4, 127.9, 148.1; **HRMS** (ESI-Orbitrap) calcd. for $C_{13}H_{18}$ $[M-H_2O+H]^+$: 175.1481, found: 175.1478.

(2*S*,3*R*)-3-Methyl-2-phenylhexan-2-ol (3a'a')

3a'a' was synthesized analogous to **3aa**. **Yield**: 166 mg (863 µmol, 86 %, *dr* > 97:3); colorless oil, **R_f** = 0.11 (*n*-pentane/Et_2O 9:1); **$[\alpha]_D^{20}$** = +10.2 [$CHCl_3$, c = 1.00]; spectroscopic data identical to enantiomer **3aa**.

Chiral HPLC of **3aa**, **3a'a** and co-injection of **3aa**/**3a'a** and **3aa**/**3a'a'**.
Method for all measurements: *OD-H*, Hex:iPrOH, 99:1, 1 ml/min.

3aa:

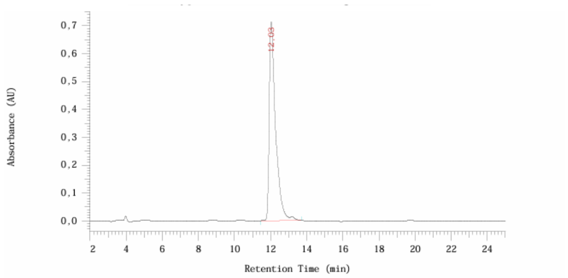

Peak Quantitation: AREA
Calculation Method: AREA%
Peak Quantitation: AREA

No.	RT	Area	Area %
1	12,03	8785091	100,000
		8785091	100,000

3a'a:

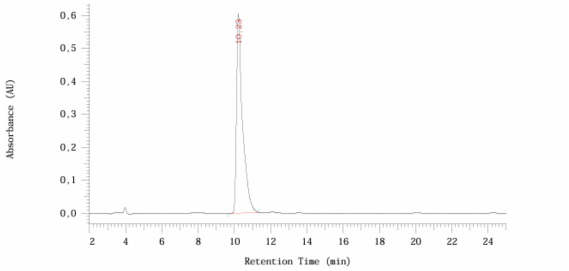

Peak Quantitation: AREA
Calculation Method: AREA%
Peak Quantitation: AREA

No.	RT	Area	Area %
1	10,23	6956246	100,000
		6956246	100,000

3aa/3a'a:

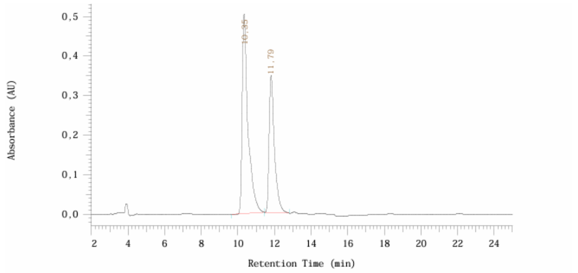

Peak Quantitation: AREA
Calculation Method: AREA%
Peak Quantitation: AREA

No.	RT	Area	Area %
1	10,35	5547303	61,141
2	11,79	3525679	38,859
3	41,09	0	0,000
4	47,35	0	0,000
		9072982	100,000

3aa/3a'a':

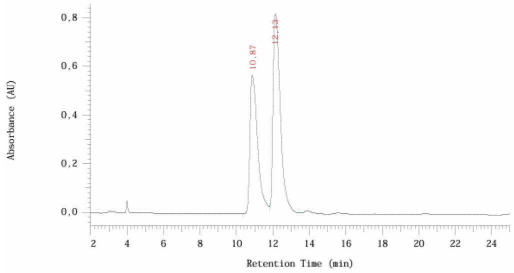

Peak Quantitation: AREA
Calculation Method: AREA%
Peak Quantitation: AREA

No.	RT	Area	Area %
1	10,87	0	0,000
2	12,13	0	0,000
		0	0,000

(2*S*,4*S*)-4-Methyl-2-phenylheptan-2-ol (3a'b)

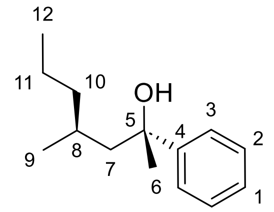

According to **GP5** 97.0 mg (389 µmol, 1.0 eq.) **1a'** were deprotonated using 308 µl (401 µmol, 1.03 eq.,1.3 M in cyclohexane) *s*-BuLi for 20 min and reacted with 131 mg (408 µmol, 1.05 eq.) **2b** for 0.5 h. Then 71.6 mg (389 µmol, 1.0 eq.) $MgBr_2$ (dry) in 389 µl methanol were added. The reaction was stirred at room temperature for 1 h. The crude boronic ester was briefly purified by flash chromatography (**R_f** = 0.25–0.30, *n*-pentane/Et_2O 99:1) and directly oxidized according to **GP5.1** using 182 µl (1.95 mmol, 5.0 eq. 33% in H_2O) H_2O_2 and 78.0 mg (1.95 mmol, 5.0 eq., 0.5 M in H_2O) NaOH. The crude product was purified by flash chromatography (SiO_2, *60M*, *n*-pentane/Et_2O 9:1); **Yield**: 52.0 mg (250 µmol, 64 %, *dr* >97:3); colorless oil; **R_f** = 0.24 (*n*-pentane/Et_2O 8:2); $[\alpha]_D^{20}$ = - 19.1 [$CHCl_3$, c = 1.00]; **^{1}H-NMR** (400 MHz, $CDCl_3$): δ = 0.70 (d, $^3J_{9,8}$ = 6.6 Hz,3 H, 9-H), 0.82 (t, $^3J_{12,11}$ = 6.9 Hz, 3 H, 12-H), 1.04–1.11 (m, 1 H, 10-H_a), 1.17–1.30 (m, 3 H, 10-H_b, 11-H), 1.49–1.53 (m, 1 H, 8-H), 1.56 (s, 3 H, 6-H), 1.64–1.69 (m, 2 H, 7-H_a, O-H), 1.81 (dd, $^3J_{7b,8}$ = 4.4 Hz, $^2J_{7b,7a}$ = 14.5 Hz, 1 H, 7-H_b), 7.21–7.24 (m, 1 H, 1-H), 7.31–7.34 (m, 2 H, 3-H), 7.43–7.45 (m, 2 H, 2-H); **^{13}C-NMR** (100 MHz, $CDCl_3$): δ = 14.3 (C-12), 19.9 (C-11), 21.4 (C-9), 28.7 (C-8), 30.8 (C-6), 40.9 (C-10), 51.2 (C-7), 75.1 (C-5), 124.8 (C-2), 126.4 (C-1), 128.0 (C-3), 148.5 (C-4); **HRMS** (ESI-Orbitrap) calcd. for $C_{14}H_{23}O$ $[M+H]^+$: 207.1743, found: 207.1746.

(2*R*,3*R*)-3-Methoxy-2-phenylbutan-2-ol (3a'd)

According to **GP5** 94.7 mg (380 µmol, 1.0 eq.) **1a'** were deprotonated using 307 µl (399 µmol, 1.05 eq., 1.3 M in cyclohexane) *s*-BuLi for 20 min and reacted with 117 mg (399 µmol, 1.05 eq.) **2d** for 1 h. The reaction was stirred at room temperature for 1 h. The crude boronic ester was briefly purified by flash chromatography (R_f = 0.27–0.32, *n*-pentane/Et_2O 8:2) and directly oxidized according to **GP5.1** using 178 µl (1.90 mmol, 5.0 eq. 33% in H_2O) H_2O_2 and 76.0 mg (1.90 mmol, 5.0 eq., 0.5 M in H_2O) NaOH. The crude product was purified by flash chromatography (SiO_2, *60M*, *n*-pentane/Et_2O 8:2); Yield: 44.4 mg (246 µmol, 65%, *dr* 97:3); colorless oil, **R_f** = 0.13 (*n*-pentane/Et_2O 8:2); **^{1}H-NMR** (400 MHz, $CDCl_3$): δ = 1.05 (d, *J* = 6.3 Hz, 3 H), 1.48 (s, 3 H), 3.30 (s, 3 H), 3.44 (q, *J* = 6.3 Hz, 1 H), 7.23–7.27 (m, 1 H), 7.32–7.36 (m, 2 H), 7.47–7.50 (m, 2 H); **^{13}C-NMR** (100 MHz, $CDCl_3$): δ = 13.2, 22.9, 57.4, 76.4, 83.8, 125.8, 126.9, 128.0, 145.7; **HRMS** (ESI-Orbitrap) calcd. for $C_{11}H_{15}O$ $[M-H_2O+H]^+$: 163.1117, found: 163.1108.

(2*R*,3*S*)-3-Methoxy-2-phenylbutan-2-ol (3a'd')

According to **GP5** 251 mg (1.01 mmol, 1.0 eq.) **1a'** were deprotonated using 739 µl (1.04 mmol, 1.05 eq., 1.3 M in cyclohexane) *s*-BuLi for 20 min and reacted with 310 mg (1.06 mmol, 1.05 eq.) **2d'** for 14 h. 185 mg (1.01 mmol, 1.0 eq.) $MgBr_2$ (dry) in 1.00 ml methanol was added. The reaction was stirred at room temperature for 45 min. The crude boronic ester was briefly purified by flash chromatography (R_f = 0.27–0.32, *n*-pentane/Et_2O 8:2) and directly oxidized according to **GP5.1** using 471 µl (5.03 mmol, 5.0 eq. 33% in H_2O) H_2O_2 and 201 mg (5.03 mmol, 5.0 eq., 0.5 M in H_2O) NaOH. The crude product was purified by flash chromatography (SiO_2, *60M*, *n*-pentane/Et_2O 9:1); **Yield**: 132 mg (732 µmol, 72%, *dr* > 97:3); colorless oil, **R_f** = 0.10 (*n*-pentane/Et_2O 9:1); $[\alpha]_D^{20}$ = +70.7 [$CHCl_3$, c = 1.00]; **^{1}H-NMR** (400 MHz, $CDCl_3$): δ = 0.89 (d, *J* = 6.3 Hz, 3 H), 1.59 (s, 3 H), 3.41 (s, 3 H), 3.43 (q, *J* = 6.3 Hz, 1

H), 7.21–7.25 (m, 1 H), 7.31–7.35 (m, 2 H), 7.40–7.42 (m, 2 H); **^{13}C-NMR** (100 MHz, $CDCl_3$): δ = 13.0. 27.4. 57.4, 76.1, 83.3, 125.2, 126.5, 127.9, 144.9; **HRMS** (CI) calcd. for $C_{11}H_{17}O_2$ $[M+H]^+$: 181.1223, found: 181.1221.

Chiral HPLC measurement of **3a'd'**.
Method: *OD-H*, Hex:iPrOH, 9:1, 1 ml/min.

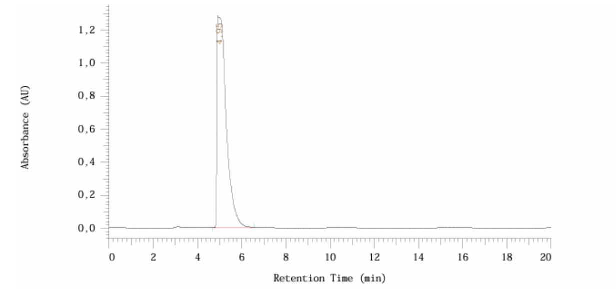

Peak Quantitation: AREA
Calculation Method: AREA%
Peak Quantitation: AREA

No.	RT	Area	Area %
1	4.95	18757704	100.000
		18757704	100.000

(2*S*,4*S*)-4-((4-Methoxybenzyl)oxy)-2-phenylpentan-2-ol (3a'e')

According to **GP4** 79.1 mg (153 µmol, 1.0 eq.) **4a'e'** were dissolved in THF and reacted with 71.5 µl (763 µmol, 5.0 eq. 33% in H_2O) H_2O_2 and 30.5 mg (763 µmol, 5.0 eq., 0.5 M in H_2O) NaOH at 0 °C for 20 min. The crude product was purified by flash chromatography (SiO_2, *60M*, *n*-pentane/Et_2O 8:2). **Yield**: 44.8 mg (149 µmol, 97%, *dr* > 97:3); colorless oil; **R_f** = 0.17 (*n*-pentane/Et_2O 8:2); $[\alpha]_D^{20}$ = - 3.2 [$CHCl_3$, c = 1.00]; **^{1}H-NMR** (500 MHz, $CDCl_3$):δ = 1.23 (d, *J* = 6.0 Hz, 3 H), 1.53 (s, 3 H), 1.82 (dd, *J* = 2.7, 14.9 Hz, 1 H), 1.97 (dd, *J* =10.4, 14.9 Hz, 1 H), 3.81 (s, 3 H), 4.06 (dqd, *J* = 2.7, 6.0, 10.4 Hz, 1 H), 4.38 (d, *J* = 10.8 Hz, 1 H), 4.63 (d, *J* = 10.8 Hz, 1 H), 6.88–6.91 (m, 2 H), 7.19–7.22 (m, 1 H), 7.27–7.33 (m, 4 H), 7.44–7.47 (m, 2 H); **^{13}C-NMR** (125 MHz, $CDCl_3$): δ = 19.7, 28.3, 50.1, 55.3, 70.0, 73.1, 73.8, 114.0, 124.5, 126.3, 128.0, 129.7, 129.8, 149.4, 159.4; slow decomposition in $CDCl_3$ was observed; **HRMS** (CI) calcd. for $C_{19}H_{23}O_2$ $[M+H-H_2O]^+$: 283.1693, found: 283.1675.

Chiral HPLC measurement of **3a'e'**.
Method: *OD-H*, Hex:iPrOH, 95:5, 1 ml/min.

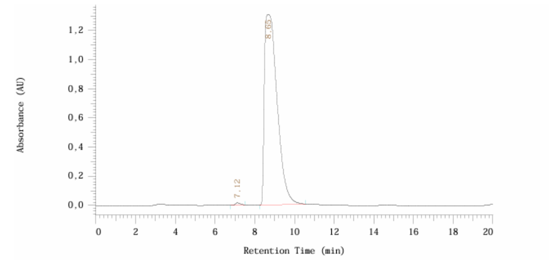

Peak Quantitation: AREA
Calculation Method: AREA%
Peak Quantitation: AREA

No.	RT	Area	Area %
1	7.12	135996	0.464
2	8.65	29190883	99.536
		29326879	100.000

(2*S*,3*R*,4*R*)-4-((4-Methoxybenzyl)oxy)-3-methyl-2-phenylpentan-2-ol (3a'f)

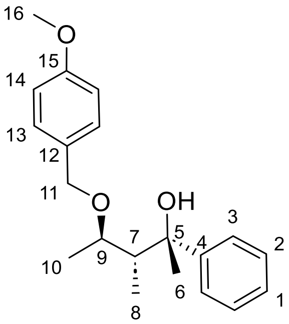

According to **GP5** 81.2 mg (326 µmol, 1.0 eq.) **1a'** were deprotonated using 258 µl (355 µmol, 1.05 eq., 1.3 M in cyclohexane) *s*-BuLi for 20 min and reacted with 151 mg (342 µmol, 1.05 eq.) **2f** for 3 h. 60.0 mg (326 µmol, 1.0 eq.) $MgBr_2$ (dry) in 326 µl methanol were added. The reaction was stirred at room temperature for 1 h. The crude boronic ester was briefly purified by flash chromatography (R_f = 0.30–0.36, *n*-pentane/Et_2O 9:1) and directly oxidized according to **GP5.1** using 153 µl (1.63 mmol, 5.0 eq. 33% in H_2O) H_2O_2 and 65.2 mg (1.63 mmol, 5.0 eq., 0.5 M in H_2O) NaOH. The crude product was purified by flash chromatography (SiO_2, *60M*, *n*-pentane/Et_2O 8:2); **Yield**: 64.0 mg (204 µmol, 62 %, *dr* > 97:3); colorless oil; $\mathbf{R_f}$ = 0.13 (*n*-pentane/Et_2O 8:2); $[\alpha]_D^{20}$ = - 76.1 [$CHCl_3$, c = 1.00]; **^{1}H-NMR** (400 MHz, $CDCl_3$): 0.96 (d, $^3J_{8,7}$ = 7.2 Hz, 3 H, 8-H), 1.16 (d, $^3J_{10,9}$ = 6.1 Hz, 3 H, 10-H), 1.54 (s, 3 H, 6-H), 2.05 (qd, $^3J_{7,8}$ = 7.2 Hz, $^3J_{7,9}$ = 8.8 Hz, 1 H, 7-H), 3.12 (qd, $^3J_{9,10}$ = 6.1 Hz, $^3J_{9,7}$ = 8.8 Hz, 1 H, 9-H), 3.82 (s, 3 H, 16-H), 4.06 (d, $^2J_{11a,11b}$ = 10.6 Hz, 1 H, 11-H_a), 4.46 (d, $^2J_{11b,11a}$ = 10.6 Hz, 1 H, 11-H_b), 5.58 (br.s, 1 H, OH), 6.89–6.91 (m, 2 H, 14-H), 7.22–7.29 (m,3 H, 1-H, 13-H), 7.30–7.34 (m, 2 H, 2-H), 7.37–7.39 (m, 2 H, 3-H);**^{13}C-NMR** (100 MHz, $CDCl_3$): δ = 13.7 (C-8), 17.5 (C-10), 29.5 (C-6), 50.1 (C-7), 55.3 (C-16), 70.0 (C-11), 77.6 (C-5), 79.4 (C-9), 114.0 (C-14), 126.0 (C-2), 126.3 (C-1), 127.6 (C-3), 129.4 (C-13), 129.9 (C-12), 146.3 (C-4), 159.3 (C-15);; **HRMS** (ESI-Orbitrap) calcd. for $C_{20}H_{27}O_3$ $[M+H]^+$: 315.1955, found: 315.1940.

(2*S*,4*S*)-4-Methyl-2-phenyl-5-(trityloxy)pentan-2-ol (3a'h)

According to **GP5** 88.7 mg (356 µmol, 1.0 eq.) **1a** were deprotonated using 282 µl (366 µmol, 1.1 eq., 1.4 M in cyclohexane) *s*-BuLi for 20 min and reacted with 206 mg (374 µmol, 1.05 eq.) **2h** for 1 h. Then 65.5 mg (356 µmol, 1.0 eq.) $MgBr_2$ (dry) in 356 µl methanol were added. The reaction was stirred at room temperature for 0.5 h. The crude boronic ester was briefly purified by flash chromatography ($\mathbf{R_f}$ = 0.11–0.12, *n*-pentane/Et_2O 99:1) and directly oxidized according to **GP5.1** using 167 µl (1.78 mmol, 5.0 eq. 33% in H_2O) H_2O_2 and 71.2 mg (1.78 mmol, 5.0 eq., 0.5 M in H_2O) NaOH. The crude product was purified by flash chromatography (SiO_2, *60M*, *n*-pentane/Et_2O 85:15); **Yield**: 69.2 mg (158 mmol, 44 %, *dr* 97:3); colorless oil; $\mathbf{R_f}$ = 0.16 (*n*-pentane/Et_2O 85:15); $[\alpha]_D^{20}$ = -14.7 [$CHCl_3$, c = 1.00]; **^{1}H-NMR** (500 MHz, $CDCl_3$): 0.88 (d, *J* = 6.9 Hz, 3 H), 1.55 (s, 3 H), 1.65 (dd, *J* = 5.4, 14.5 Hz, 1 H), 1.88 (dd, *J* = 5.7, 14.5 Hz, 1 H) 1.96 (ddqd, *J* = 5.4, 5.7, 6.9, 12.6 Hz, 1 H), 2.78 (br. s, 1 H), 2.80 (dd, *J* = 7.6, 9.1 Hz, 1 H), 2.94 (dd, *J* = 5.4, 9.1 Hz, 1 H), 7.19–7.24 (m, 4 H), 7.26–7.30 (m, 8 H), 7.27 (m, 8 H); **^{13}C-NMR** (125 MHz, $CDCl_3$): δ = 19.6, 30.1, 30.4, 49.1, 69.8, 74.4, 87.1, 124.7, 126.3, 126.9, 127.7, 128.0, 128.7, 144.0, 148.9; **HRMS** (ESI-Orbitrap) calcd. for $C_{12}H_{19}O_2$ $[M+H\text{-Trityl}]^+$: 195.1380, found: 195.1378.

Chiral HPLC measurement of **3a'h**.
Method: *OD-H*, Hex:iPrOH, 95:5, 1 ml/min.

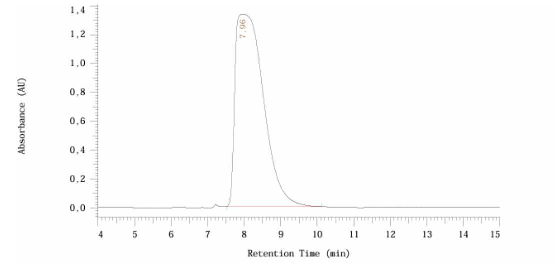

Peak Quantitation: AREA
Calculation Method: AREA%
Peak Quantitation: AREA

No.	RT	Area	Area %
1	7.96	35931100	100.000
		35931100	100.000

(2*R*,3*R*)-2-(4-Chlorophenyl)-3-((4-methoxybenzyl)oxy)butan-2-ol (3b'c)

According to **GP5** 91.5 mg (322 µmol, 1.0 eq.) **1b'** were deprotonated using 255 µl (332 µmol, 1.03 eq., 1.4 M in cyclohexane) *s*-BuLi for 20 min and reacted with 136 mg (339 µmol, 1.05 eq.) **2c** for 0.5 h. Then 59.4 mg (322 µmol, 1.0 eq.) $MgBr_2$ (dry) in 322 µl methanol were added. The reaction was stirred at room temperature for 1.5 h. The crude boronic ester was briefly purified by flash chromatography (**R_f** = 0.26–0.27, *n*-pentane/Et_2O 95:5) and directly oxidized according to **GP5.1** using 151 µl (1.61 mmol, 5.0 eq. 33% in H_2O) H_2O_2 and 64.4 mg (1.61 mmol, 5.0 eq., 0.5 M in H_2O) NaOH. The crude product was purified by flash chromatography (SiO_2, *60M*, *n*-pentane/Et_2O 8:2); **Yield**: 61.9 mg (193 µmol, 60%, *dr* > 97:3); colorless oil; **R_f** = 0.13 (*n*-pentane/Et_2O 8:2); $[\alpha]_D^{20}$ = - 88.2 [$CHCl_3$, c = 1.00]; **^{1}H-NMR** (400 MHz, $CDCl_3$): δ = 1.13 (d, *J* = 6.2 Hz, 3 H), 1.44 (s, 3 H), 2.98 (br. s, 1 H), 3.35 (dqd, *J* = 6.2 Hz, 1 H), 3.80 (s, 3 H), 4.21 (d, *J* = 11.3 Hz, 1 H), 4.47 (d, *J* = 11.3 Hz, 1 H), 6.83–6.86 (m, 2 H), 7.03–7.05 (m, 2 H), 7.27–7.29 (m, 2 H), 7.35–7.37 (m, 2 H); **^{13}C-NMR** (100 MHz, $CDCl_3$): δ = 13.7, 23.7, 55.3, 71.0, 76.2*, 80.8, 113.7, 127.2, 128.0, 129.3, 130.0, 132.5, 144.8, 159.2; *determined with HMBC; **HRMS (**CI**)** calcd. for $C_{18}H_{20}ClO_3$ $[M\text{-}H+]^-$: 319.1106, found: 319.1109.

Chiral HPLC measurement of **3b'c**.
Method: *OD-H*, Hex:iPrOH, 95:5, 1 ml/min.

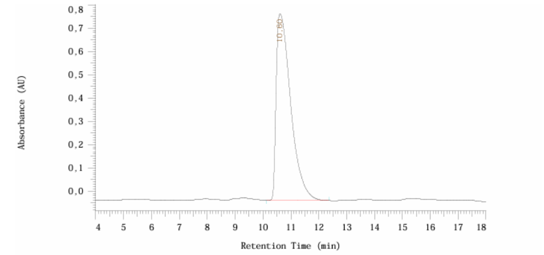

Peak Quantitation: AREA
Calculation Method: AREA%
Peak Quantitation: AREA

No.	RT	Area	Area %
1	10.60	14509456	100.000
		14509456	100.000

(2*R*,4*R*)-2-(4-Methoxyphenyl)-4-methylheptan-2-ol (3cb')

According to **GP5** 288 mg (1.03 mmol, 1.0 eq.) **1c** were deprotonated using 171 µl (1.13 mmol, 1.1 eq., ρ = 0.77 g/ml) TMEDA and 758 µl (1.06 mmol, 1.1 eq., 1.4 M in cyclohexane) *s*-BuLi for 10 min and reacted with 136 mg (339 µmol, 1.05 eq.) **2b'** for 14 h. Then 190 mg (1.03 mmol, 1.0 eq.) $MgBr_2$ (dry) in 1.03 ml methanol were added. The reaction was stirred at room temperature for 0.5 h. The crude boronic ester was briefly purified by flash chromatography (**R_f** = 0.40–0.41, *n*-pentane/Et_2O 99:1) and directly oxidized according to **GP5.1** using 483 µl (5.16 mmol, 5.0 eq. 33% in H_2O) H_2O_2 and 206 mg

(5.16 mmol, 5.0 eq., 0.5 M in H_2O) NaOH. The crude product was purified by flash chromatography (SiO_2, *60M*, *n*-pentane/Et_2O 85:15); **Yield**: 174 mg (736 µmol, 71 %, *dr* > 97:3); colorless oil; $\mathbf{R_f}$ = 0.2 (*n*-pentane/Et_2O 8:2); $[\alpha]_D^{20}$ = +19.5 [$CHCl_3$, c = 1.00]; **^{1}H-NMR** (500 MHz, $CDCl_3$): δ = 0.72 (d, *J* = 6.6 Hz, 3 H), 0.82 (t, *J* = 7.0 Hz, 1 H), 1.04–1.11 (m, 1 H), 1.15–1.28 (m, 3 H), 1.45–1.55 (m, 1 H), 1.54 (s, 3 H), 1.61–1.66 (m, 2 H), 1.78 (dd, *J* = 4.4, 14.3 Hz, 1 H), 6.85–6.87 (m, 2 H), 7.34–7.37 (m, 2 H); **^{13}C-NMR** (125 MHz, $CDCl_3$): δ = 14.3, 19.9, 21.4, 28.7, 30.8, 40.9, 51.3, 55.2, 74.8, 113.1, 126.0, 140.8, 158.1; **HRMS** (CI) calcd. for $C_{15}H_{24}O_2$ $[M]^+$: 236.1776, found: 236.1746.

Chiral HPLC measurement of **3cb'**.
Method: *OD-H*, Hex:iPrOH, 95:5, 1 ml/min.

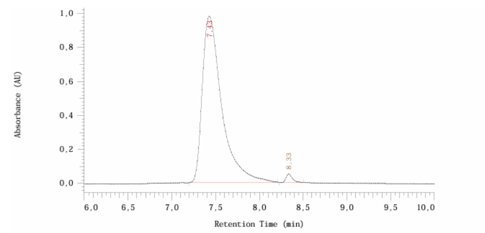

Peak Quantitation: AREA
Calculation Method: AREA%
Peak Quantitation: AREA

No.	RT	Area	Area %
1	7.43	7564608	98.426
2	8.33	120991	1.574
		7685599	100.000

(2*R*,3*R*)-2-(4-Chlorophenyl)-3-((4-methoxybenzyl)oxy)butan-2-ol (3cc')

Accroding to **GP4** 145 mg (272 µmol, 1.0 eq.) **4cc'** were dissolved in THF and reacted with 127 µl (1.36 mmol, 5.0 eq. 33% in H_2O) H_2O_2 and 140 mg (1.36 mmol, 5.0 eq., 0.5 M in H_2O) NaOH at 0 °C for 0.5 h. The crude product was purified by flash chromatography (SiO_2, *60M*, *n*-pentane/Et_2O 8:2). **Yield**: 80.0 mg (253 µmol, 93%, *dr* > 97:3); colorless oil; $\mathbf{R_f}$ = 0.04 (*n*-pentane/Et_2O 8:2); $[\alpha]_D^{20}$ = +92.5 [$CHCl_3$, c = 1.00]; **^{1}H-NMR** (500 MHz, $CDCl_3$): δ = 1.09 (d, *J* = 6.4 Hz, 1 H), 1.47 (s, 3 H), 3.00 (br. s, 1 H), 3.62 (q, *J* = 6.4 Hz, 1 H), 3.80 (s, 3 H), 3.81 (s, 3 H), 4.26 (dd, *J* = 11.1 Hz, 1 H), 4.48 (dd, *J* = 11.1 Hz, 1 H), 6.83–6.88 (m, 4 H), 7.11–7.13 (m, 2 H), 7.35–7.38 (m, 2 H); **^{13}C-NMR** (125 MHz, $CDCl_3$): δ = 14.0, 23.2, 55.2, 55.2, 71.2, 76.1, 81.5, 113.3, 113.7, 126.9, 129.3, 130.4, 138.0, 158.4, 159.1; **HRMS** (CI) calcd. for $C_{19}H_{23}O_3$ $[M+H-H_2O]^+$: 299.1642, found: 299.1637.

Chiral HPLC measurement of **3cc'**.
Method: *OD-H*, Hex:iPrOH, 9:1, 1 ml/min.

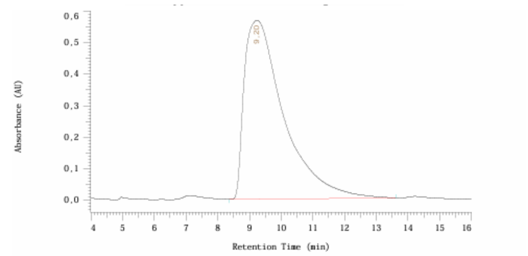

Peak Quantitation: AREA
Calculation Method: AREA%
Peak Quantitation: AREA

No.	RT	Area	Area %
1	9.20	24693916	100.000
		24693916	100.000

(2*R*,4*S*)-4-((4-Methoxybenzyl)oxy)-2-(3-methoxyphenyl)pentan-2-ol (3de')

According to **GP5** 280 mg (1.00 mmol, 1.0 eq.) **1d** were deprotonated using 166 µl (1.10 mmol, 1.1 eq., ρ = 0.77 g/ml) TMEDA and 737 µl (1.04 mmol, 1.1 eq., 1.4 M in cyclohexane) *s*-BuLi for 10 min and

reacted with 436 mg (1.05 mmol, 1.05 eq.) **2e’** for 2 h. Then 185 mg (1.00 mmol, 1.0 eq.) $MgBr_2$ (dry) in 1.00 ml methanol were added. The reaction was stirred at room temperature for 0.5 h. The crude boronic ester was briefly purified by flash chromatography (**R_f** = 0.42–0.43, *n*-pentane/Et_2O 8:2) and directly oxidized according to **GP5.1** using 469 µl (5.00 mmol, 5.0 eq. 33% in H_2O) H_2O_2 and 200 mg (5.00 mmol, 5.0 eq., 0.5 M in H_2O) NaOH. The crude product was purified first by flash chromatography (SiO_2, *60M*, *n*-pentane/Et_2O 9:1) then by reversed-phase flash chromatography (H_2O/ACN 99:1–ACN); **Yield**: 204 mg (617 µmol, 62 %,*dr* > 97:3); colorless oil; **R_f** = 0.07 (*n*-pentane/Et_2O 8:2); $[\alpha]_D^{20}$ = -12.9 [$CHCl_3$, c = 1.00]; **^{1}H-NMR** (400 MHz, $CDCl_3$):δ = 1.23 (d, *J* = 6.0 Hz, 3 H), 1.52 (s, 3 H), 1.82 (dd, *J* = 2.8, 14.9 Hz, 1 H), 1.97 (dd, *J* =10.4, 14.9 Hz, 1 H), 3.80 (s, 3 H), 3.81 (s, 3 H), 4.06 (m, 1 H), 4.38 (d, *J* = 10.8 Hz, 1 H), 4.63 (d, *J* = 10.8 Hz, 1 H), 6.74–6.77 (m, 1 H), 6.88–6.91 (m, 2 H), 6.97–6.99 (m, 1 H), 7.08–7.09 (m, 1 H), 7.20–7.24 (m, 1 H), 7.27–7.31 (m, 2 H); **^{13}C-NMR** (100 MHz, $CDCl_3$): δ = 19.7, 28.3, 50.0, 55.2, 55.3, 70.0, 73.1, 73.8, 110.3, 111.8, 114.0, 116.9, 128.9, 129.6, 129.8, 151.3, 159.4, 159.5; **HRMS** (CI) calcd. for $C_{20}H_{26}O_4$ $[M]^+$: 330.1831, found: 330.1852.

Chiral HPLC measurement of **3de’**.
Method: *OD-H*, Hex:iPrOH, 9:1, 1 ml/min.

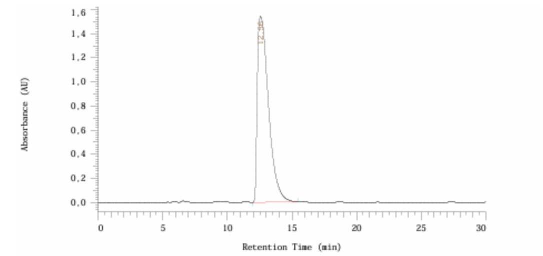

Peak Quantitation: AREA
Calculation Method: AREA%
Peak Quantitation: AREA

No.	RT	Area	Area %
1	12.56	46492217	100.000
		46492217	100.000

(2*R*,3*S*,4*S*)-4-((4-Methoxybenzyl)oxy)-2-(2-methoxyphenyl)-3-methylpentan-2-ol (3ef’)

According to **GP5** 281 mg (1.00 mmol, 1.0 eq.) **1e** were deprotonated using 167 µl (1.11 mmol, 1.1 eq., ρ = 0.77 g/ml) TMEDA and 739 µl (1.04 mmol, 1.1 eq., 1.4 M in cyclohexane) *s*-BuLi for 10 min and reacted with 452 mg (1.06 mmol, 1.05 eq.) **2f’** for 3 h. Then 185 mg (1.00 mmol, 1.0 eq.) $MgBr_2$ (dry) in 1.00 ml methanol were added. The reaction was stirred at room temperature for 1 h. The crude boronic ester was briefly purified by flash chromatography (**R_f** = 0.11–0.12, *n*-pentane/Et_2O 9:1) and directly oxidized according to **GP5.1** using 941 µl (10.0 mmol, 10 eq.,33% in H_2O) H_2O_2 and 402 mg (10.0 mmol, 10.0 eq., 0.5 M in H_2O) NaOH at 50 °C for 4. The crude product was purified by flash chromatography (SiO_2, *60M*, *n*-pentane/Et_2O 8:2); **Yield**: 137 mg (398 µmol, 40%, *dr* > 97:3); colorless oil; **R_f** = 0.14 (*n*-pentane/Et_2O 8:2); $[\alpha]_D^{20}$ = +8.4 [$CHCl_3$, c = 1.00]; **^{1}H-NMR** (400 MHz, $CDCl_3$):δ = 0.93 (d, *J* = 7.2 Hz, 3 H), 1.16 (d, *J* = 6.4 Hz, 3 H), 1.58 (s, 3 H), 2.55 (dq, *J* = 4.2, 7.2 Hz, 1 H), 3.43 (dq, *J* = 4.2, 6.4 Hz, 1 H), 3.80 (s, 3 H), 3.85 (s, 3 H), 4.16 (d, *J* = 11.0 Hz, 1 H), 4.26 (d, *J* = 11.0 Hz, 1 H), 6.82–6.84 (m, 2 H), 6.89–6.91 (m, 1 H), 6.93–6.96 (m, 1 H), 7.12–7.14 (m, 2 H), 7.21–7.25 (m, 1 H), 7.34–7.36 (m, 2 H); **^{13}C-NMR** (100 MHz, $CDCl_3$): δ = 10.8, 17.1, 25.3, 45.8, 55.3, 55.3, 69.8, 76.5, 77.1*, 111.3, 113.7, 120.7, 127.5, 128.0, 129.1, 130.9, 135.2, 156.5, 159.0; *determined with HMBC/HSQC; **HRMS** (ESI-Orbitrap) calcd. for $C_{21}H_{29}O_4$ $[M+H]^+$: 345.2060, found: 345.2041.

Chiral HPLC measurement of **3ef’**.
Method: *OD-H*, Hex:iPrOH, 95:5, 1 ml/min.

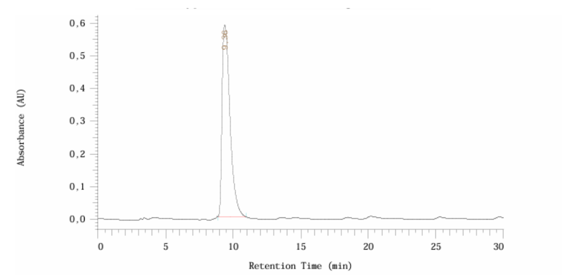

Peak Quantitation: AREA
Calculation Method: AREA%
Peak Quantitation: AREA

No.	RT	Area	Area %
1	9,36	11930692	100,000
		11930692	100,000

(2*R*,3*R*)-3-(2-((4-Methoxybenzyl)oxy)ethyl)-2-(3-methoxyphenyl)hex-5-en-2-ol (3dg)

According to **GP4** 81.7 mg (139 µmol, 1.0 eq.) **4dg** were dissolved in THF and reacted with 65 µl (694 µmol, 5.0 eq. 33% in H_2O) H_2O_2 and 27.8 mg (694 µmol, 5.0 eq., 0.5 M in H_2O) NaOH at 0 °C for 0.5 h. The crude product was purified by flash chromatography (SiO_2, *60M*, *n*-pentane/Et_2O 9:1). **Yield**: 43.6 mg (118 µmol, 85%, *dr* > 97:3); colorless oil; $\mathbf{R_f}$ = 0.30 (n-pentane/Et_2O 7:3); $[\alpha]_D^{20}$ = - 24.8 [$CHCl_3$, c = 1.00]; **^{1}H-NMR** (400 MHz, $CDCl_3$):δ = 1.53 (s, 3 H), 1.74–1.81 (m, 1 H), 1.84–1.91 (m, 1 H), 1.93–2.03 (m, 2 H), 2.03–2.11 (m, 1 H), 3.40 (ddd, *J* = 4.8, 6.2, 9.6 Hz, 1 H), 3.57 (ddd, *J* = 4.5, 8.0, 9.6 Hz, 1 H), 3.80 (s, 3 H), 3.81 (s, 3 H), 4.43–4.50 (m, 1 H), 4.85–4.92 (m, 2 H), 5.71–5.56 (m, 1 H), 6.74–6.77 (m, 1 H), 6.88–6.91 (m, 2 H), 6.97–6.99 (m, 1 H), 7.03–7.04 (m, 1 H), 7.21–7.24 (m, 1 H), 7.27–7.28 (m, 2 H); **^{13}C-NMR** (100 MHz, $CDCl_3$): δ = 27.7, 29.1, 33.3, 46.8, 55.2, 55.3, 67.5, 72.8, 76.1*, 111.3, 111.4, 113.8, 115.9, 117.7, 128.8, 129.4, 129.7, 138.1, 149.9, 159.3, 159.3;*determined with HMBC/HSQC; **HRMS** (ESI-Orbitrap) calcd. for $C_{23}H_{31}O_4$ $[M+H]^+$: 371.2217, found: 371.2200.

Chiral HPLC measurement of **3dg**.
Method: *OD-H*, Hex:iPrOH, 9:1, 1 ml/min.

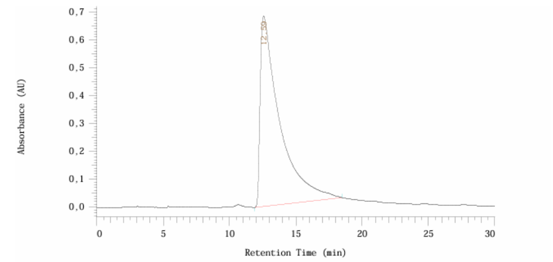

Peak Quantitation: AREA
Calculation Method: AREA%
Peak Quantitation: AREA

No.	RT	Area	Area %
1	12,59	34896809	100,000
		34896809	100,000

(4*R*,5*S*,*E*)-4,5-Dimethyloct-2-en-4-ol (3fa)

According to **GP5** 213 mg (1.00 mmol, 1.0 eq.) **1f** were deprotonated using 166 µl (1.10 mmol, 1.1 eq., ρ = 0.77 g/ml) TMEDA and 786 µl (1.10 mmol, 1.1 eq., 1.4 M in cyclohexane) *s*-BuLi for 10 min and reacted with 368 mg (1.20 mmol, 1.2 eq.) **2f'** for 1 h. Then 184 mg (1.00 mmol, 1.0 eq.) $MgBr_2$ (dry) in 1.00 ml methanol were added. The reaction was stirred at room temperature for 1 h. The crude boronic ester was briefly purified by flash chromatography ($\mathbf{R_f}$ = 0.28–0.29, *n*-pentane/Et_2O 99:1) and directly oxidized according to **GP5.1** using 436 µl (4.66 mmol, 5.0 eq.,33% in H_2O) H_2O_2 and 186 mg (5.03 mmol, 5.0 eq., 0.5 M in H_2O) NaOH at 0 °C for 0.5. The crude product was purified by flash chromatography (SiO_2, *60M*, *n*-pentane/Et_2O 8:2); Yield: 98.6 mg (631 mmol, 63 %, *dr* > 97:3); colorless, volatile liquid;

R$_f$ = 0.26 (*n*-pentane/Et_2O 8:2); **[α]$_D^{20}$** = -24.3 [$CHCl_3$, c = 1.00]; **^{1}H-NMR** (400 MHz, $CDCl_3$): δ = 0.88–0.96 (m, 1 H), 0.88 (d, *J* = 6.6 Hz, 3 H), 0.89 (t, *J* = 6.8 Hz, 3 H), 1.13–1.19 (m, 1 H), 1.21 (s, 3 H), 1.34 (br. s, 1 H), 1.39–1.52 (m, 3 H), 1.71 (dd, *J* = 1.2, 6.1 Hz, 3 H), 5.53 (dd, *J* = 1.2, 15.6 Hz, 1 H), 5.62 (qd, *J* = 6.1, 15.6 Hz, 1 H); **^{13}C-NMR** (100 MHz, $CDCl_3$): δ = 14.1, 14.4, 17.8, 21.2, 24.9, 33.5, 43.2, 75.2*, 123.2, 137.0; *determined with HMBC/HSQC; **HRMS** (ESI-Orbitrap) calcd. for $C_{10}H_{19}$ [M+H-H_2O]$^+$: 139.1481, found: 139.1478.

Chiral HPLC measurement of **3fa** failed due to decomposition.

(4*R*,6*S*,*E*)-4-Ethyl-6-((4-methoxybenzyl)oxy)hept-2-en-4-ol (3ge')

According to **GP4** 125 mg (252 µmol, 1.0 eq.) **4ge'** were dissolved in THF and reacted with 118 µl (1.26 mmol, 5.0 eq. 33% in H_2O) H_2O_2 and 50.3 mg (1.26 mmol, 5.0 eq., 0.5 M in H_2O) NaOH at 0 °C for 0.5 h. The crude product was purified by flash chromatography (SiO_2, *60M*, *n*-pentane/Et_2O 9:1). **Yield**: 58.0 mg (204 µmol, 81%, *dr* >97:3, purity 99%, impurity: 4-methoxybenzaldehyd); colorless liquid; **R**$_f$ = 0.16 (*n*-pentane/Et_2O 85:15); **[α]$_D^{20}$** = +137.3 [$CHCl_3$, c = 1.00]; **^{1}H-NMR** (400 MHz, $CDCl_3$):δ = 0.83 (t, *J* = 7.5 Hz, 3 H), 1.20 (d, *J* = 6.0 Hz, 1 H), 1.37 (qd, *J* = 7.5, 13.8 Hz, 1 H), 1.45–1.50 (m, 2 H), 1.70 (dd, *J* = 1.5, 6.5 Hz, 3 H), 1.79 (dd, *J* = 10.9, 14.6 Hz, 1 H), 3.80 (s, 1 H), 3.81–3.89 (m, 1 H), 4.22 (d, *J* = 10.8 Hz, 1 H), 4.41 (br. s, 1 H), 4.53 (d, *J* = 10.8 Hz, 1 H), 5.34 (dd, *J* = 1.5, 15.3 Hz, 1 H), 5.65 (dd, *J* = 6.5, 15.3 Hz, 1 H), 6.86–6.89 (m, 2 H), 7.22–7.24 (m, 2 H); **^{13}C-NMR** (100 MHz, $CDCl_3$): δ = 7.57, 17.7, 19.6, 35.3, 46.2, 55.3, 69.9, 73.6, 75.1, 113.9, 123.8, 129.7, 129.9, 136.2, 159.3; **HRMS** (ESI-Orbitrap) calcd. for $C_{17}H_{25}O_2$ [M+H-H_2O]$^+$: 261.1849, found: 261.1849.

Chiral HPLC measurement of **3ge'**.
Method: *OD-H*, Hex:iPrOH, 95:5, 1 ml/min.

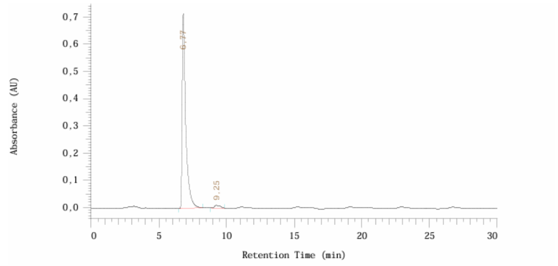

Peak Quantitation: AREA
Calculation Method: AREA%
Peak Quantitation: AREA

No.	RT	Area	Area %
1	6.77	6580799	97.824
2	9.25	146351	2.176
		6727150	100.000

(4*S*,5*R*,7*S*)-4,7-Dimethyl-5-phenyldecan-5-ol (3ha)

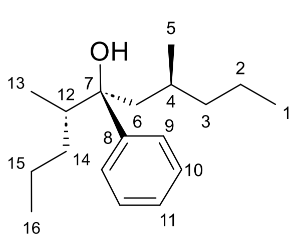

According to **GP5** 319 mg (1.00 mmol, 1.0 eq.) **1h** were deprotonated using 743 µl (1.04 mmol, 1.03 eq., 1.4 M in cyclohexane) *s*-BuLi for 3.5 h and reacted with 324 mg (1.06 mmol, 1.05 eq.) **2a** for 14 h. Then 184 mg (1.01 mmol, 1.0 eq.) $MgBr_2$ (dry) in 1.01ml methanol were added. The reaction was stirred at room temperature for 14 h. The crude boronic ester was briefly purified by flash chromatography (**R**$_f$

= 0.30–0.35, *n*-pentane/Et_2O 99:1) and directly oxidized according to **GP5.1** using 471 µl (5.03 mmol, 5.0 eq.,33% in H_2O) H_2O_2 and 201 mg (5.03 mmol, 5.0 eq., 0.5 M in H_2O) NaOH at 50 °C for 2. The crude product was purified by flash chromatography (SiO_2, *60M*, *n*-pentane/Et_2O 9:1); *Yield*: 142 mg (541 µmol, 54 %, *dr* 91:9); colorless oil; R_f = 0.23 (*n*-pentane/Et_2O 95:5);

^{1}H-NMR (400 MHz, $CDCl_3$): δ = 0.57 (d, $^3J_{5,4}$ = 6.6 Hz, 3 H, 5-H), 0.68 (d, $^3J_{13,12}$ = 6.7 Hz, 3 H, 13-H), 0.81 (t, $^3J_{1,2}$ = 7.0 Hz, 3 H, 1-H), 0.85–0.98 (m, 2 H, 14-H_a, 15-H_b), 0.90 (t, $^3J_{16,15}$ = 7.5 Hz, 3 H, 16-H), 1.05–1.48 (m, 7 H, 2-H, 3-H, 4-H, 15-H), 1.60 (br.s, 1 H, OH), 1.65–1.81 (m, 3 H, 6-H_a, 12-H, 14-H_b), 1.92 (dd, $^3J_{6b,4}$ = 5.3 Hz, $^2J_{6b,6a}$ = 14.4 Hz, 1 H, 6-H_b), 7.19–7.23 (m, 1 H, 11-H), 7.29–7.34 (m, 2 H, 9-H), 7.36–7.38 (m, 2 H, 10-H); **^{13}C-NMR** (100 MHz, $CDCl_3$): δ = 14.2 (C-1), 14.3 (C-13), 14.4 (C-16), 20.0 (C-2), 21.1 (C-15), 21.4 (C-5), 28.5 (C-4), 32.7 (C-14), 40.8 (C-3), 43.9 (C-12), 46.9 (C-6) 79.8 (C-7), 125.9 (C-10), 126.0 (C-11), 127.6 (C-9), 146.0 (C-8); **HRMS** (ESI-Orbitrap) calcd. for $C_{18}H_{31}O$ $[M+H]^+$: 263.2369, found: 263.2361.

Selected NMR-signals of diastereomer:
^{1}H-NMR (400 MHz, $CDCl_3$): δ = 0.70 (t, *J* = 7.0 Hz, 3 H, 1-H), 0.73 (t, *J* = 7.3 Hz, 3 H, 16-H), 0.86 (d, *J* = 6.5 Hz, 3 H, 5-H), 0.98 (d, *J* = 6.7 Hz, 3 H, 13-H), 1.87 (dd, *J* = 3.2, 14.4 Hz, 1 H, 6-H_a); **^{13}C-NMR** (100 MHz, $CDCl_3$): δ = 13.2, 14.1, 19.7, 28.7, 29.7, 41.2, 44.0, 46.8, 125.8, 126.0, 127.7.

Chiral HPLC measurement of **3ha**.
Method: *OD-H*, Hex:iPrOH, 100:0, 1 ml/min.

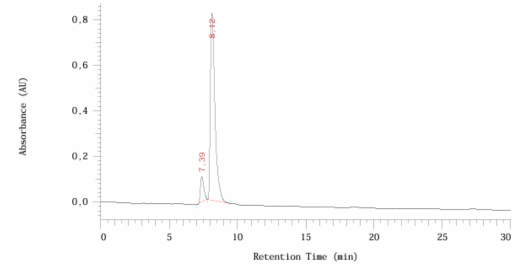

Peak Quantitation: AREA
Calculation Method: AREA%
Peak Quantitation: AREA

No.	RT	Area	Area %
1	7.39	949609	8.901
2	8.12	9718922	91.099
		10668531	100.000

(2*S*,3S,5*S*)-2-((4-Methoxybenzyl)oxy)-5-methyl-3-phenyloctan-3-ol (3hc')

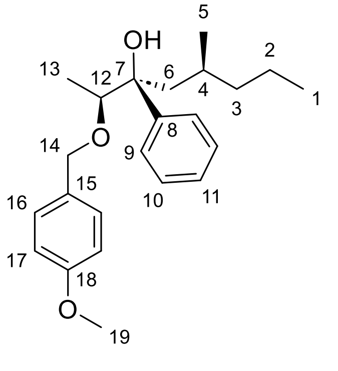

According to **GP4** 81.2 mg (141 µmol, 1.0 eq.) **4hc'** were dissolved in THF and reacted with 132 µl (1.41 mmol, 5.0 eq. 33% in H_2O) H_2O_2 and 50.3 mg (1.26 mmol, 5.0 eq., 0.5 M in H_2O) NaOH at 50 °C

for 2.5 h. The crude product was purified by flash chromatography (SiO_2, *60M*, *n*-pentane/Et_2O 8:2). Yield: 42.7 mg (120 µmol, 85%, *dr* > 97:3); colorless oil; **R$_f$** = 0.10 (*n*-pentane/Et_2O 9:1); **$[\alpha]_D^{20}$** = +33.2 [$CHCl_3$, c = 1.00]; **^{1}H-NMR** (400 MHz, $CDCl_3$):δ = 0.57 (d, $^3J_{5,4}$ = 6.6 Hz, 3 H, 5-H), 0.78 (t, $^3J_{1,2}$ = 7.0 Hz, 3 H, 1-H), 1.02–1.11 (m, 2 H, 2-H$_a$, 3-H$_a$), 1.19 (d, $^3J_{13,12}$ = 6.2 Hz, 3 H, 13-H), 1.23–1.29 (m, 1 H, 2-H$_b$), 1.38–1.46 (m, 2 H, 3-H$_b$, 4-H), 1.64 (dd, $^3J_{6a,4}$ = 5.2 Hz, $^2J_{6a,6b}$ = 14.3 Hz, 1 H, 6-H$_a$), 1.75 (dd, $^3J_{6b,4}$ = 6.1 Hz, $^2J_{6b,6a}$ = 14.3 Hz, 1 H, 6-H$_b$), 2.76 (br.s, 1 H, OH), 3.62 (q, $^3J_{12,13}$ = 6.2 Hz, 1 H, 12-H), 3.78 (s, 3 H, 19-H), 4.08 (d, $^3J_{14a,14b}$ = 11.2 Hz, 1 H, 14-H$_a$), 4.33 (d, $^3J_{15b,15a}$ = 11.2 Hz, 1 H, 14-H$_b$), 6.78 (d, $^3J_{17,16}$ = 8.5 Hz, 2 H, 17-H), 6.93 (d, $^3J_{16,17}$ = 8.5 Hz, 2 H, 16-H), 7.22–7.24 (m, 1 H, 11-H), 7.30–7.34 (m, 2 H, 10-H), 7.41–7.44 (m, 2 H, 9-H).**^{13}C-NMR** (100 MHz, $CDCl_3$): δ = 13.6 (C-13), 14.3 (C-1), 19.9 (C-2), 21.2 (C-5), 28.2 (C-4), 40.2 (C-3), 43.4 (C-6), 55.3 (C-19), 71.2 (C-14), 79.2* (C-7), 81.7 (C-12), 113.6 (C-17), 126.2 (C-9), 126.3 (C-11), 127.7 (C-10), 129.2 (C-16), 130.3 (C-15), 145.4 (C-8), 159.0 (C-18); *determined with HMBC/HSQC; **HRMS** (CI) calcd. for $C_{23}H_{31}O_2$ $[M+H-H_2O]^+$: 339.2319, found: 339.2316.
And in a second fraction: 1.80 mg (3.53 µmol, 2.5%, purity: 70%, impurity: (4*S*,5*S*)-4,5-Dicyclohexyl-2-methyl-1,3,2-dioxaborolan)(2*S*,3R,5S)-2-((4-Methoxybenzyl)oxy)-5-methyl-3-phenyloctan-3-ol; **R$_f$** = 0.11 (n-pentane/Et_2O 9:1); Selected ^{1}H-NMR-signals of diastereomer: **^{1}H-NMR** (400 MHz, $CDCl_3$):δ = 0.81 (t, *J* = 7.3 Hz, 3 H), 1.19 (d, *J* = 6.3 Hz, 3 H), 3.66 (q, *J* = 6.3 Hz, 1 H), 3.79 (s, 3 H), 4.13 (d, *J* = 11.2 Hz, 1 H), 4.38 (d, *J* = 11.2 Hz, 1 H), 6.79 (d, *J* = 8.5 Hz, 1 H), 6.98 (d, *J* = 8.5 Hz, 1 H), 7.23–7.26 (m, 1 H), 7.32–7.35 (m, 2 H), 7.40–7.42 (m, 2 H).

Chiral HPLC measurement of **3hc'** (first fraction).
Method: *OD-H*, Hex:iPrOH, 99:1, 1 ml/min.

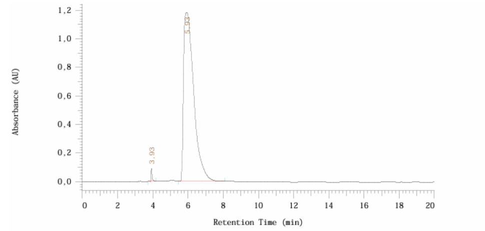

Peak Quantitation: AREA
Calculation Method: AREA%
Peak Quantitation: AREA

No.	RT	Area	Area %
1	3.93	188396	0.788
2	5.93	23711028	99.212
		23899424	100.000

(2*S*,4*R*,6*S*)-2-((4-Methoxybenzyl)oxy)-6-methyl-4-phenylnonan-4-ol (3he')

According to **GP4** 81.4 mg (138 µmol, 1.0 eq.) **4hc'** were dissolved in THF and reacted with 130 µl (1.38 mmol, 5.0 eq. 33% in H_2O) H_2O_2 and 55.3 mg (1.38 mmol, 5.0 eq., 0.5 M in H_2O) NaOH at 0 °C for 20 min. The crude product was purified by flash chromatography (SiO_2, *60M*, *n*-pentane/Et_2O 96:4). **Yield**: 45.0 mg (121 µmol, 88%, *dr* > 97:3); colorless oil; **R$_f$** = 0.19 (*n*-pentane/Et_2O 95:5); **$[\alpha]_D^{20}$** = +140.3 [$CHCl_3$, c = 1.00]; **^{1}H-NMR** (400 MHz, $CDCl_3$):δ = 0.53 (d, *J* = 6.5 Hz, 3 H), 0.82 (d, *J* = 6.9 Hz, 3 H), 1.02–1.10 (m, 1 H), 1.12 (d, *J* = 6.0 Hz, 3 H), 1.14–1.31 (m, 3 H), 1.53 (dd, *J* = 7.1,13.6 Hz, 1 H), 1.55–1.63 (m, 1 H), 1.75 (dd, *J* = 3.8, 13.6 Hz, 1 H), 1.95 (dd, *J* = 2.4,14.7 Hz, 1 H), 2.07 (dd, *J* = 10.8, 14.7 Hz, 1 H), 3.31 (ddd, *J* = 2.4, 6.0, 10.8 Hz, 1 H), 3.81 (s, 3 H), 3.86 (d, *J* = 10.5 Hz, 1 H), 4.33 (d, *J* = 10.5 Hz, 1 H), 4.85 (br. s, 1 H), 6.84 (d, *J* = 8.6 Hz, 1 H), 7.15 (d, *J* = 8.6 Hz, 1 H), 7.17–7.23 (m, 1 H), 7.30–7.34 (m, 1 H), 7.34–7.38 (m, 1 H); **^{13}C-NMR** (100 MHz, $CDCl_3$): δ = 14.3, 19.6, 20.0, 21.1, 27.9, 41.1, 48.9, 51.7, 55.3, 70.1, 73.8, 77.2*, 113.9, 125.6, 125.9, 127.9, 129.7, 129.8, 147.6, 159.3; *determined with HMBC/HSQC; $C_{24}H_{33}O_2$ $[M+H-H_2O]^+$: 353.2475, found: 353.2460.

Chiral HPLC measurement of **3he'**.

Method: *OD-H*, Hex:*i*PrOH, 98:2, 1 ml/min.

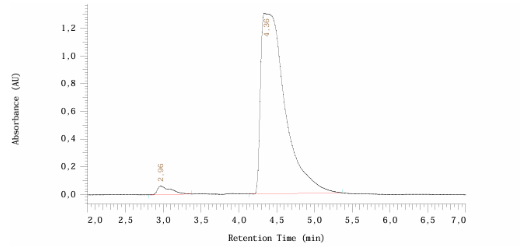

Peak Quantitation: AREA
Calculation Method: AREA%
Peak Quantitation: AREA

No.	RT	Area	Area %
1	2,96	371227	2,479
2	4,36	14604614	97,521
		14975841	100,000

Synthesis and isolation of tertiary boronic ester 4

(4*R*,5*R*)-4,5-Dicyclohexyl-2-((2*S*,3*S*)-3-((4-methoxybenzyl)oxy)-2-phenylbutan-2-yl)-1,3,2-dioxaborolane (4a'c')

According to **GP5** 248 mg (993 µmol, 1.0 eq.) **1a'** were deprotonated using 731 µl (1.02 mmol, 1.03 eq., 1.3 M in cyclohexane) *s*-BuLi for 20 min and reacted with 418 mg (1.04 mmol, 1.05 eq.) **2c'** for 14 h. 183 mg (993 µmol, 1.0 eq.) $MgBr_2$ (dry) in 1.00 ml methanol were added. The reaction was stirred at room temperature for 1 h. The crude product was purified by flash chromatography (SiO_2, *60*, *n*-pentane/Et_2O 95:5); **Yield**: 427 mg (801 µmol, 81%, purity 95%, impurity: *n*-pentane); colorless oil; $\mathbf{R_f}$ = 0.22 (*n*-pentane/Et_2O 95:5); $[\alpha]_D^{20}$ = + 35.5 [$CHCl_3$, c = 1.00]; **^{1}H-NMR** (400 MHz, $CDCl_3$): δ = 0.87–0.96 (m, 4 H), 1.01–1.09 (m, 9 H), 1.24–1.33 (m, 2 H), 1.44 (s, 3 H), 1.52–1.55 (m, 2 H), 1.57–1.77 (m, 8 H), 3.77 (s, 3 H), 4.00 (q, *J* = 6.2 Hz,1 H), 4.07 (d, *J* = 11.4 Hz, 1 H), 4.31 (d, *J* = 11.4 Hz, 1 H), 6.74–6.76 (m, 2 H), 6.93–6.95 (m, 2 H), 7.14–7.18 (m, 1 H), 7.25–7.29 (m, 2 H), 7.25–7.29 (m, 2 H); **^{13}C-NMR** (100 MHz, $CDCl_3$): δ = 16.0, 16.8, 25.9, 25.9, 26.4, 27.7, 28.3, 42.9, 55.2, 71.1, 80.8, 83.5, 113.4, 125.3, 126.3, 127.7, 127.9, 131.5, 144.9, 158.6; **HRMS** (ESI-Orbitrap) calcd. for $C_{32}H_{46}BO_4$ $[M+H]^+$: 505.3484, found: 505.3470.

(4*R*,5*R*)-4,5-Dicyclohexyl-2-((2*R*,4*S*)-4-((4-methoxybenzyl)oxy)-2-phenylpentan-2-yl)-1,3,2-dioxaborolane (4a'e')

According to **GP5** 354 mg (1.42 mmol, 1.0 eq.) **1a'** were deprotonated using 731 µl (1.16 ml (1.46 mmol, 1.03 eq., 1.3 M in cyclohexane) *s*-BuLi for 20 min and reacted with 621 mg (1.50 mmol, 1.05 eq.) **2c'** for 2 h. 261 mg (1.42 mmol, 1.0 eq.) $MgBr_2$ (dry) in 1.42 ml methanol were added. The reaction was stirred at room temperature for 0.5 h. The crude product was purified by flash chromatography (SiO_2, *60*, *n*-pentane/Et_2O 96:4); **Yield**: 609 mg (1.14 mmol, 80%); colorless oil; $\mathbf{R_f}$ = 0.17 (*n*-pentane/Et_2O 95:5); $[\alpha]_D^{20}$ = +50.4 [$CHCl_3$, c = 1.00]; **^{1}H-NMR** (500 MHz, $CDCl_3$): δ = 0.77–0.94 (m, 4 H), 1.05–1.09 (m, 6 H), 1.09 (d, *J* = 6.0 Hz, 3 H), 1.12–1.18 (m, 2 H), 1.40 (s, 3 H), 1.42–1.45 (m, 2 H), 1.57–1.60 (m, 2 H), 1.65–1.75 (m, 6 H), 1.78 (dd, *J* = 3.6, 14.1 Hz, 1 H), 2.33 (dd, *J* = 8.7, 14.1 Hz, 1 H), 3.46 (dqd, *J* = 3.6, 6.0, 8.7 Hz, 1 H), 3.72–3.74 (m, 2 H), 3.80 (s, 3 H), 4.35 (d, *J* = 11.9 Hz, 1 H), 4.44 (d, *J* = 11.9 Hz, 1 H), 6.85–6.88 (m, 2 H), 7.10–7.13 (m, 1 H), 7.24–7.28 (m, 4 H, 5-H), 7.37–7.40 (m, 2 H); **^{13}C-NMR** (125 MHz, $CDCl_3$): δ = 20.4, 20.8, 25.8, 25.9, 26.4, 27.9, 28.4, 42.9, 46.4, 55.3, 69.2, 71.6,

83.7, 113.6, 125.0, 126.9, 127.9, 129.1, 131.1, 147.4, 158.9; **HRMS** (CI) calcd. for $C_{33}H_{47}BO_4$ $[M]^+$: 518.3567, found: 518.3534.

(4*R*,5*R*)-4,5-Dicyclohexyl-2-((2*R*,3*S*)-3-((4-methoxybenzyl)oxy)-2-(4-methoxyphenyl)-butan-2-yl)-1,3,2-dioxaborolane (4cc')

According to **GP5** 288 mg (1.03 mmol, 1.0 eq.) **1c** were deprotonated using 171 µl (1.14 mmol, 1.1 eq., ρ = 0.77 g/ml) TMEDA and 759 µl (1.06 mmol, 1.03 eq., 1.3 M in cyclohexane) *s*-BuLi for 10 min and reacted with 434 mg (1.08 mmol, 1.05 eq.) **2c'** for 14 h. 190 mg (1.03 mmol, 1.0 eq.) $MgBr_2$ (dry) in 1.03 ml methanol were added. The reaction was stirred at room temperature for 1 h. The crude product was purified by flash chromatography (SiO_2, *60*, *n*-pentane/Et_2O 95:5); **Yield**: 491.7 mg (872 µmol, 85%, purity: 95%, impurity: *n*-pentane); colorless oil; $\mathbf{R_f}$ = 0.27 (*n*-pentane/Et_2O 9:1); $[\alpha]_D^{20}$ = +30.0 [$CHCl_3$, c = 1.00]; **^{1}H-NMR** (400 MHz, $CDCl_3$): δ = 0.74–0.84 (m, 2 H), 0.87–0.96 (m, 2 H), 0.88 (d, *J* = 6.1 Hz, 3 H), 0.98–1.09 (m, 6 H), 1.17–1.24 (m, 2 H), 1.42 (s, 3 H), 1.42–1.46 (m, 2 H), 1.57–1.67 (m, 8 H), 3.78 (s, 3 H), 3.78–3.80 (m, 2 H), 3.80 (s, 3 H), 4.15 (q, *J* = 6.1 Hz, 1 H), 4.49 (d, *J* = 11.8 Hz, 1 H), 4.59 (d, *J* = 11.8 Hz, 1 H), 6.81–6.86 (m, 4 H, 6-H), 7.25–7.31 (m, 4 H, 5-H); **^{13}C-NMR** (100 MHz, $CDCl_3$): δ = 12.7, 14.0, 25.7, 25.9, 26.4, 27.3, 28.1, 42.9, 55.2, 55.3, 70.3, 80.2, 83.4, 113.3, 113.5, 128.0, 128.4, 131.9, 136.1, 157.3, 158.6; **HRMS** (ESI-Orbitrap) calcd. for $C_{33}H_{48}BO_5$ $[M+H]^+$: 535.3589, found: 535.3578.

(4*S*,5*S*)-4,5-Dicyclohexyl-2-((2*S*,3*R*)-3-(2-((4-methoxybenzyl)oxy)ethyl)-2-(3-methoxyphenyl)-hex-5-en-2-yl)-1,3,2-dioxaborolane (4dg)

According to **GP5** 257 mg (920 µmol, 1.0 eq.) **1d** were deprotonated using 153 µl (1.01 mmol, 1.1 eq., ρ = 0.77 g/ml) TMEDA and 667 µl (947 µmol, 1.03 eq., 1.3 M in cyclohexane) *s*-BuLi for 10 min and reacted with 439 mg (966 µmol, 1.05 eq.) **2g** for 14 h. 169 mg (920 µmol, 1.0 eq.) $MgBr_2$ (dry) in 920 µl methanol were added. The reaction was stirred at room temperature for 1 h. The crude product was purified by flash chromatography (SiO_2, *60*, *n*-pentane/Et_2O 95:5); **Yield**: 307.9 mg (523 µmol, 57%); colorless oil; $\mathbf{R_f}$ = 0.14 (*n*-pentane/Et_2O 9:1); $[\alpha]_D^{20}$ = -13.4 [$CHCl_3$, c = 1.00]; **^{1}H-NMR** (400 MHz, $CDCl_3$): δ = 0.77–0.96 (m, 4 H), 1.05–1.12 (m, 6 H), 1.15–1.21 (m, 2 H), 1.25–1.38 (m, 1 H), 1.29 (s, 3 H), 1.43–1.46 (m, 2 H), 1.48–1.63 (m, 3 H), 1.65–1.73 (m, 6 H), 2.06 (td, *J* = 8.4,13.4 Hz, 1 H), 2.18–2.27 (m, 2 H), 3.00 (dt, *J* = 6.8, 9.3 Hz, 1 H), 3.23 (dt, *J* = 5.3, 9.3 Hz, 1 H), 3.76 (s, 3 H), 3.76–3.80 (m, 2 H), 3.79 (s, 3 H), 4.19 (s, 2 H), 4.93 (d, *J* = 9.8 Hz, 1 H), 5.03 (d, *J* = 16.3 Hz, 1 H), 5.86 (dddd, *J* = 6.0, 8.4, 9.8,16.3 Hz, 1 H), 6.65–6.68 (m, 2 H), 6.82–6.84 (m, 2 H), 6.94–6.95 (m, 1 H), 6.96–6.98 (m, 1 H), 7.12–7.15 (m, 3 H); **^{13}C-NMR** (100 MHz, $CDCl_3$): δ = 14.9, 25.8, 25.9, 26.4, 27.6, 28.4, 30.2, 40.1, 41.1, 43.0, 55.1, 55.2, 69.8, 71.9, 83.6, 110.3, 113.6, 113.7, 115.2, 120.2, 128.6, 129.1, 130.9, 139.0, 147.6, 158.9, 159.3; **HRMS** (ESI-Orbitrap) calcd. for $C_{37}H_{54}BO_5$ $[M+H]^+$: 589.4059, found: 589.4044.

(4*R*,5*R*)-4,5-Dicyclohexyl-2-((4*S*,6*S*,*E*)-4-ethyl-6-((4-methoxybenzyl)oxy)hept-2-en-4-yl)-1,3,2-dioxaborolane (4ge')

According to **GP5** 227 mg (1.00 mmol, 1.0 eq.) **1d** were deprotonated using 166 µl (1.10 mmol, 1.1 eq., ρ = 0.77 g/ml) TMEDA and 840 µl (1.10 mmol, 1.1 eq., 1.3 M in cyclohexane) *s*-BuLi for 30 min and reacted with 467 mg (1.07 mmol, 1.07 eq.) **2e'** for 14 h. 184 mg (1.00 mmol, 1.0 eq.) $MgBr_2$ (dry) in 1.00 ml methanol were added. The reaction was stirred at room temperature for 1 h. The crude product was purified by flash chromatography (SiO_2, *60*, *n*-pentane/Et_2O 9:1); **Yield**: 298 mg (600 µmol, 60%); colorless oil; $\mathbf{R_f}$ = 0.2 (*n*-pentane/Et_2O 9:1); $[\alpha]_D^{20}$ = +36.3 [$CHCl_3$, c = 1.00]; **^{1}H-NMR** (400 MHz, $CDCl_3$): δ = 0.81 (t, *J* = 7.4 Hz, 3 H), 0.90–1.00 (m, 4 H), 1.11–1.26 (m, 12 H), 1.35–1.47 (m, 2 H), 1.42–1.45 (m, 2

H), 1.52–1.60 (m, 3 H), 1.65–1.73 (m, 9 H), 1.83–1.87 (m, 2 H), 1.92 (dd, *J* = 8.0, 13.9 Hz, 1 H), 3.46–3.51 (m, 1 H), 4.38–4.46 (m, 2 H), 5.33 (qd, *J* = 1.5, 15.8 Hz, 1 H), 5.51 (qd, *J* = 6.3, 15.8 Hz, 1 H), 6.84–6.87 (m, 2 H), 7.25–7.27 (m, 2 H); **^{13}C-NMR** (100 MHz, $CDCl_3$): δ = 10.0, 18.7, 20.4, 25.9, 26.0, 26.5, 28.2, 28.7, 29.7, 43.1, 44.3, 55.2, 69.3, 72.4, 83.6, 113.5, 123.4, 129.0, 131.4, 136.6, 158.8; **HRMS** (CI) calcd. for $C_{31}H_{49}BO_4$ $[M]^+$: 496.3704, found: 496.3724.

(4*R*,5*R*)-4,5-Dicyclohexyl-2-((2*S*,3*R*,5*S*)-2-((4-methoxybenzyl)oxy)-5-methyl-3-phenyloctan-3-yl)-1,3,2-dioxaborolane (4hc')

According to **GP5** 266 mg (831 µmol, 1.0 eq.) **1h** were deprotonated using 653 µl (856 µmol, 1.03 eq., 1.3 M in cyclohexane) *s*-BuLi for 3.5 h and reacted with 466 mg (1.16 mmol,, 1.4 eq.) **2c'** for 3 h. 153 mg (831 µmol, 1.0 eq.) $MgBr_2$ (dry) in 831 µl methanol were added. The reaction was stirred at room temperature for 14 h. The crude product was purified by flash chromatography (SiO_2, *60*, *n*-pentane/Et_2O 99:1); **Yield**: 379 mg (660 µmol, 79%); colorless oil; **R_f** = 0.47 (*n*-pentane/Et_2O 9:1); **$[\alpha]_D^{20}$** = +12.9 [$CHCl_3$, c = 1.00]; **^{1}H-NMR** (400 MHz, $CDCl_3$): δ = 0.64 (d, *J* = 6.7 Hz, 3 H), 0.80 (t, *J* = 7.1 Hz, 3 H), 0.90–1.05 (m, 6 H), 1.02 (d, *J* = 6.2 Hz, 3 H), 1.08–1.20 (m, 8 H), 1.24–1.37 (m, 5 H), 1.48–1.53 (m, 1 H), 1.55–1.78 (m, 10 H), 1.82–1.85 (m, 2 H), 1.91–1.94 (m, 2 H), 3.80 (s, 3 H), 3.81–3.85 (m, 2 H), 3.93 (q, *J* = 6.2 Hz, 1 H), 4.33 (d, *J* = 11.4 Hz, 1 H), 4.50 (d, *J* = 11.4 Hz, 1 H), 6.83–6.85 (d, $J_{21,20}$ = 8.6 Hz, 2 H, 21-H), 7.11–7.15 (m, 1 H), 7.20–7.25 (m, 4 H), 7.52 (d, $^3J_{14,15}$ = 7.6 Hz, 2 H); **^{13}C-NMR** (100 MHz, $CDCl_3$): δ = 14.5, 16.3, 20.3, 20.9, 25.9, 26.0, 26.5, 28.1, 28.9, 31.1, 41.3, 42.2, 43.1, 55.3, 70.8, 80.5, 83.9, 113.4, 125.2, 127.2, 128.5, 129.9, 131.9, 143.5, 158.6; **HRMS** (CI) calcd. for $C_{37}H_{56}BO_4$ $[M+H]^+$: 575.4266, found: 575.4220.

(4*R*,5*R*)-4,5-Dicyclohexyl-2-((2*S*,4*S*,6*S*)-2-((4-methoxybenzyl)oxy)-6-methyl-4-phenylnonan-4-yl)-1,3,2-dioxaborolane (4he')

According to **GP5** 198 mg (620 µmol, 1.0 eq.) **1h** were deprotonated using 456 µl (638 µmol, 1.03 eq., 1.3 M in cyclohexane) *s*-BuLi for 3.5 h and reacted with 270 mg (654 µmol, 1.05 eq.) **2e'** for 3 h. 114 mg (620 µmol, 1.0 eq.) $MgBr_2$ (dry) in 620 µl methanol were added. The reaction was stirred at room temperature for 14 h. The crude product was purified by flash chromatography (SiO_2, *60*, *n*-pentane/Et_2O 99:1); **Yield**: 242 mg (412 µmol, 67%); colorless oil; **R_f** = 0.43 (*n*-pentane/Et_2O 96:4); **$[\alpha]_D^{20}$** = +43.3 [$CHCl_3$, c = 1.00]; **^{1}H-NMR** (400 MHz, $CDCl_3$): δ = 0.53 (d, *J* = 6.6 Hz, 3 H), 0.80 (t, *J* = 7.0 Hz, 3 H), 0.86–1.01 (m, 7 H), 0.95 (d, *J* = 6.1 Hz, 3 H), 1.10–1.28 (m, 13 H), 1.31–1.35 (m, 1 H), 1.48–1.52 (m, 2 H), 1.60–1.66 (m, 2 H), 1.68–1.71 (m, 6 H), 1.77 (dd, *J* = 4.4, 14.2 Hz, 1 H), 1.86–1.90 (m, 3 H), 2.36 (dd, *J* = 8.2, 14.2 Hz, 1 H), 3.16 (dqd, *J* = 4.4, 6.1, 8.2 Hz, 1 H), 3.70–3.73 (m, 2 H), 4.20–4.27 (m, 2 H), 6.77–6.79 (d, *J* = 8.7 Hz, 2 H), 7.07–7.09 (d, *J* = 8.7 Hz, 2 H), 7.10–7.13 (t, *J* = 7.6 Hz, 2 H), 7.22–7.25 (m, 2 H), 7.53 (d, *J* = 7.6 Hz, 2 H); **^{13}C-NMR** (100 MHz, $CDCl_3$): δ = 14.5, 20.1, 20.4, 20.4, 25.8, 25.9, 25.9, 26.5, 28.6, 29.0, 30.9, 41.1, 42.9, 46.4, 55.2, 69.6, 72.8, 84.1, 113.5, 124.9, 127.6, 128.3, 128.8, 131.6, 146.2, 158.7; **HRMS** (CI) calcd. for $C_{38}H_{58}BO_4$ $[M+H]^+$: 589.4423, found: 589.4450.

Further transformations of 4a'e'

(4*R*,5*R*)-4,5-Dicyclohexyl-2-((2*S*,4*S*)-4-((4-methoxybenzyl)oxy)-2-methyl-2-phenylpentyl)-1,3,2-dioxaborolane (5 a'e')

Preparation of the LDA-stock solution: 182 µl (1.28 mmol, 1.35 eq., ρ = 0.71 g/ml) DIPA were dissolved in 347 µL THF and 470 µl (1.18 mmol, 1.25 eq., 2.5 M in *n*-hexane) *n*-BuLi were added dropwise at -40 °C under continuous stirring. After 10 min the reaction was warmed to room temperature and stirred for 20 min prior to use.

98 mg (189 µmol, 1.0 eq.) **4a'e'** and 36.2 µl (567 µmol, 3.0 eq., ρ = 1.32 g/ml) DCM were dissolved in 347 µl THF and 200 µl of the LDA-stock solution were added dropwise at -40 °C under continuous stirring. After complete addition the reaction was stirred for 15 min at -40 °C before a solution of 79.0 mg (576 µmol, 3.05 eq.) $ZnCl_2$ in 340 µL THF was added. The reaction was slowly warmed to -10 °C (over 2 h) and then to 15 °C over night (16 h). Upon confirming quantitative conversion (^{1}H-NMR) the reaction was cooled to 0 °C and 387 µl (387 µmol, 2.05 eq., 1.0 M in THF) Super-hydride® ($LiBEt_3H$) were added dropwise and stirred for additional 10 min before warming to room temperature. After 5 h complete conversion was confirmed (^{1}H-NMR) the reaction was worked up according to **GP3**. The crude product was purified by flash chromatography (SiO_2, *60*, *n*-pentane/Et_2O 95:5). **Yield**: 84.2 mg (158 µmol, 84%, *dr* > 97:3); colorless oil; $\mathbf{R_f}$ = 0.13 (, *n*-pentane/Et_2O 95:5); $[\boldsymbol{\alpha}]_{\boldsymbol{D}}^{\mathbf{20}}$ = +56.4 [$CHCl_3$, c = 1.00]; **^{1}H-NMR** (500 MHz, $CDCl_3$):δ = 0.71–0.79 (m, 2 H), 0.85–0.90 (m, 2 H), 0.97 (d, *J* = 6.2 Hz, 3 H), 1.05–1.18 (m, 8 H), 1.24 (d, *J* = 14.7 Hz, 1 H), 1.38 (d, *J* = 14.7 Hz, 1 H), 1.42–1.48 (m, 2 H), 1.51 (s, 3 H), 1.56–1.59 (m, 2 H), 1.62–1.64 (m, 2 H), 1.67–1.71 (m, 4 H), 1.88 (dd, *J* = 3.8,14.5 Hz, 1 H), 2.07 (dd, *J* = 6.4,14.5 Hz, 1 H), 3.25 (dp, *J* = 3.8 Hz, 6.2 Hz, 1 H, 2-H), 3.65–3.67 (m, 2 H), 3.79 (s, 3 H), 4.11 (d, *J* = 11.0 Hz, 1 H), 4.27 (d, *J* = 11.0 Hz, 1 H), 6.81–6.84 (m, 2 H), 7.12–7.15 (m, 3 H), 7.24–7.27 (m, 2 H), 7.32–7.35 (m, 2 H); **^{13}C-NMR** (125 MHz, $CDCl_3$): δ = 21.0, 25.8, 26.0, 26.4, 26.7, 27.3, 28.3, 38.9*, 42.9, 52.7, 55.3, 69.7, 73.1, 83.1, 113.6, 125.2, 126.3, 127.8, 129.2, 131.1, 149.2, 158.9; *determined with HMBC/HSQC; **HRMS** (CI) calcd. for $C_{34}H_{50}BO_4$ [M+H]$^+$: 533.3797, found: 533.3806.

(2*S*,4*S*)-4-((4-Methoxybenzyl)oxy)-2-methyl-2-phenylpentanal (6a'e')

Preparation of the LDA-solution: 74.2 µl (521 µmol, 1.35 eq., ρ = 0.71 g/ml) DIPA were dissolved in 143 µL THF and 193 µl (483 µmol, 1.25 eq., 2.5 M in *n*-hexane) *n*-BuLi was added dropwise at – 40 °C under continuous stirring. After 10 min the reaction was warmed to room temperature and stirred for 20 min prior to use.

200 mg (386 µmol, 1.0 eq.) **4a'e'** and 73.9 µl (1.16 mmol, 3.0 eq., ρ = 1.32 g/ml) DCM were dissolved in 715 µl THF and the LDA-solution was added dropwise at -40 °C under continuous stirring. After complete addition the reaction was stirred for 15 min at -40 °C before a solution of 160 mg (1.18 mmol, 3.05 eq.) $ZnCl_2$ in 700 µL THF were added. The reaction was slowly warmed to -10 °C (over 2 h) and then directly warmed to room temperature and stirred for 0.5 h. Upon confirming quantitative conversion (^{1}H-NMR) the reaction was worked up according to **GP3**. (*sidenote*: the crude α-chloro boronic ester was only partially characterized due to its instability). The crude product was directly reacted according to **GP4** with 181 µl (1.93 mmol, 5.0 eq. 33% in H_2O) H_2O_2 and 77.0 mg (1.93 mmol, 5.0 eq., 0.5 M in H_2O) NaOH at 0 °C for 0.5 h. The crude product was purified by flash chromatography (SiO_2, *60M*, *n*-pentane/Et_2O 9:1). Yield: 95.0 mg (304 µmol, 79%); colorless oil; $\mathbf{R_f}$ = 0.11 (, *n*-pentane/Et_2O 9:1); $[\boldsymbol{\alpha}]_{\boldsymbol{D}}^{\mathbf{20}}$ = +29.5 [$CHCl_3$, c = 1.00]; **^{1}H-NMR** (500 MHz, $CDCl_3$): δ = 1.23(d, *J* = 6.0 Hz, 3 H), 1.44 (s, 3 H), 1.96 (dd, *J* = 2.2, 14.5 Hz, 1 H), 2.44 (dd, *J* = 10.4, 14.5 Hz, 1 H), 3.57 (dqd, *J* = 2.2, 6.0, 10.4 Hz, 1 H), 3.80 (s, 3 H), 4.20 (d, *J* = 10.7 Hz, 1 H), 4.38 (d, *J* = 10.7 Hz, 1 H), 6.86–6.89 (m, 2 H), 7.09–

7.24 (m, 2 H), 7.26–7.29 (m, 3 H), 7.33–7.37 (m, 2 H), 9.48 (s, 1 H); **^{13}C-NMR** (125 MHz, $CDCl_3$): δ = 19.7, 20.0, 44.5, 52.4, 55.3, 70.1, 71.1, 113.7, 126.9, 127.1, 128.8, 129.9, 130.1, 140.7, 159.2, 201.0; **HRMS** (CI) calcd. for $C_{20}H_2O_3$ $[M]^+$: 312.1725, found: 312.1733.

Crude-NMR analysis and rotation value of **4a'e'-Cl:** **^{1}H-NMR** (500 MHz, $CDCl_3$): δ = 0.57–0.64 (m, 2 H), 0.77–0.84 (m, 2 H), 0.94 (d, *J* = 6.0 Hz, 3 H), 1.00–1.12 (m, 8 H), 1.34–1.48 (m, 4 H), 1.60–1.67 (m, 6 H), 1.65 (s, 3 H), 1.87 (dd, *J* = 3.5, 14.8 Hz, 1 H), 2.51 (dd, *J* = 7.3, 14.5 Hz, 1 H), 3.19 (dqd, *J* = 3.5, 6.0, 7.3 Hz, 1 H), 3.64–3.66 (m, 2 H), 3.72 (s, 1 H), 3.80 (s, 3 H), 4.08 (d, *J* = 11.0 Hz, 1 H), 4.30 (d, *J* = 11.0 Hz, 1 H), 6.82–6.85 (m, 2 H), 7.17–7.22 (m, 3 H), 7.27–7.33 (m, 4 H); **^{13}C-NMR** (125 MHz, $CDCl_3$): δ = 19.7, 20.8, 25.8, 25.9, 26.3, 27.0, 27.8, 42.5, 43.5, 47.5, 55.3, 69.7, 72.6, 83.4, 113.6, 126.6, 127.2, 128.2, 129.3, 131.0, 144.1, 158.9; $[\alpha]_D^{20}$ = +46.3 [$CHCl_3$, c = 1.00].

1-Methoxy-4-((((2*S*,4*R*)-4-phenylpentan-2-yl)oxy)methyl)benzol (7a'e')

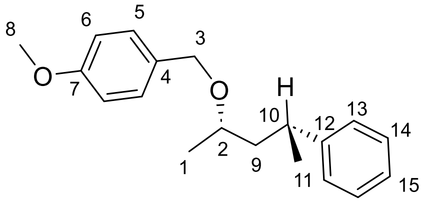

73.0 mg (141 µmol, 1.0 eq.) **4a'e'** were dissolved in 1.41 ml *n*-pentane and 353 µl (353 µmol, 2.5 eq., 1.0 M in THF, containing 5% H_2O) TBAF were added. The reaction mixture was stirred at 45 °C for 3 h. After confirming complete conversion (monitored by TLC; careful: product and **4a'e'** have similar R_f-values) the reaction was poured in a separatory funnel filled with H_2O. The organic phase was washed with sat. NaCl and dried over $MgSO_4$. After removing the solvent under reduced pressure the crude product was purified by flash chromatography (SiO_2, *60M*, *n*-pentane/Et_2O 95:5) in two fractions: **Yield**: 28.0 mg (98.4 µmol, 70 % d.Th., *dr* > 97:3) and 5.0 mg (17.5 µmol, 12%., *dr* 81:19); colorless liquid; **R_f** = 0.17 (n-pentane/Et_2O 95:5); $[\alpha]_D^{20}$ = +8.7 [$CHCl_3$, c = 1.00]; **^{1}H-NMR** (500 MHz, $CDCl_3$):δ = 1.18 (d, $^3J_{1,2}$ = 6.2 Hz, 3 H, 1-H), 1.20 (d, $^3J_{11,10}$ = 7.2 Hz, 3 H, 11-H), 1.59 (ddd, $^3J_{9a,2}$ = 6.3 Hz, $^3J_{9a,10}$ = 7.4 Hz, $^2J_{9a,9b}$ = 13.7 Hz, 1 H, 9-H_a), 1.98 (ddd, $^3J_{9b,2}$ = 6.9 Hz, $^3J_{9b,10}$ = 7.4 Hz, $^2J_{9b,9a}$ = 13.7 Hz, 1 H, 9-H_b), 2.88 (tq, $^3J_{10,9}$ = 6.9 Hz, $^3J_{10,11}$ = 7.2 Hz, 1 H, 10-H), 3.40 (qt, $^3J_{2,1}$ = 6.2 Hz, $^3J_{2,9}$ = 6.5 Hz, 1 H, 2-H), 3.80 (s, 3 H, 8-H), 4.31 (d, $^2J_{3a,3b}$ = 11.2 Hz, 1 H, 3-H_a), 4.43 (d, $^2J_{3b,3a}$ = 11.2 Hz, 1 H, 3-H_b), 6.83–6.86 (m, 2 H, 6-H), 7.16–7.22 (m, 5 H, 5-H, 13-H, 15-H), 7.26–7.29 (m, 2 H, 14-H); **^{13}C-NMR** (125 MHz, $CDCl_3$): δ = 19.7 (C-1), 22.2 (C-11), 36.2 (C-10), 45.3 (C-9), 55.3 (C-8), 69.8 (C-3), 72.4 (C-2), 113.7 (C-6), 125.9 (C-15), 126.9 (C-13), 128.4 (C-14), 129.2 (C-5), 131.1 (C-4), 147.5 (C-7), 159.0 (C-12).; **HRMS** (ESI-Orbitrap) calcd. for $C_{19}H_{25}O_2$ $[M+H]^+$: 285.1849, found: 285.1840.

Selected ^{1}H-NMR-signals of minor diastereomer: **^{1}H-NMR** (500 MHz, $CDCl_3$):δ = 1.11 (d, *J* = 6.3 Hz, 3 H), 1.21 (d, *J* = 6.9 Hz, 3 H), 2.97–3.04 (m, 1 H), 3.22 (dqd, *J* = 3.5, 6.0, 9.5 Hz, 1 H), 3.81 (s, 3 H), 4.18 (d, *J* = 11.0 Hz, 1 H).

1-Methoxy-4-((((2*S*,4*R*)-4-methyl-4-phenylhex-5-en-2-yl)oxy)methyl)benzol (8a'e')

92.6 mg (179 µmol, 1.0 eq.) **4a'e'** were dissolved in 893 µl THF and 625 µl (625 µmol, 3.5 eq., 1.0 M in THF) vinyl magnesium bromide was added at 0 °C, stirred for 15 min at 0 °C and then 15 min at room temperature. Afterwards 204 mg (804 µmol, 4.5 eq.) iodine in 1.0 ml THF were added dropwise at 0 °C and stirred for 0.5 h at this temperature. 67.5 mg (1.25 mmol, 7.0 eq.) NaOMe in 333 µl MeOH were added at 0 °C and stirred for 1 h at this temperature. The reaction was quenched with sat. $Na_2S_2O_3$ -

solution and the aqueous phase was extracted with *n*-pentane (3x). The organic phase was dried over $MgSO_4$ and the solvent evaporated under reduced pressure. For simplified isolation of the desired product the crude mixture was reacted further according **GP4** with 27.7 µl (895 µmol, 5.0 eq., 33% in H_2O) H_2O_2 and 35.8 mg (895 µmol, 5.0 eq., 0.5 M in H_2O) NaOH at 0 °C for 0.5 h. The crude product was purified by flash chromatography (SiO_2, *60M*, *n*-pentane/Et_2O 98:2). **Yield**: 22.0 mg (71.0 µmol, 40 %, *dr > 97:3*); colorless oil; $\mathbf{R_f}$ = 0.13 (*n*-pentane/Et_2O 98:2); $[\alpha]_D^{20}$ = +63.8 [$CHCl_3$, c = 1.00]; **^{1}H-NMR** (500 MHz, $CDCl_3$):δ = 1.07 (d, *J* = 6.1 Hz, 3 H), 1.45 (s, 3 H), 1.91 (dd, *J* = 3.6, 14.4 Hz, 1 H), 2.13 (dd, *J* =6.8, 14.4 Hz, 1 H), 3.36 (dp, *J* = 3.6, 6.1 Hz, 1 H), 3.80 (s, 3 H), 4.15 (d, *J*= 10.9 Hz, 1 H), 4.34 (d, *J* = 10.9 Hz, 1 H), 5.00 (d, 17.4 Hz, 1 H), 5.04 (d, *J* = 10.8 Hz, 1 H), 6.07 (dd, *J* = 10.8 H, 17.4 Hz, 1 H), 6.84–6.86 (m, 2 H), 7.17–7.21 (m, 3 H), 7.25–7.33 (m, 4 H); **^{13}C-NMR** (125 MHz, $CDCl_3$): δ = 21.1, 25.2, 43.8, 48.5*, 55.3, 69.9, 72.5, 110.9, 113.7, 125.8, 126.8, 128.0, 129.3, 131.0, 147.1, 147.8, 159.0; *determined with HMBC/HSQC; **HRMS** (ESI-Orbitrap) calcd. for $C_{21}H_{27}O_2$ $[M+H]^+$: 311.2006, found: 311.1992.

NMR-spectra

(*R*)-1-(4-Chlorophenyl)ethyldiisopropylcarbamat (1b')

^{1}H-NMR (400 MHz, $CDCl_3$):

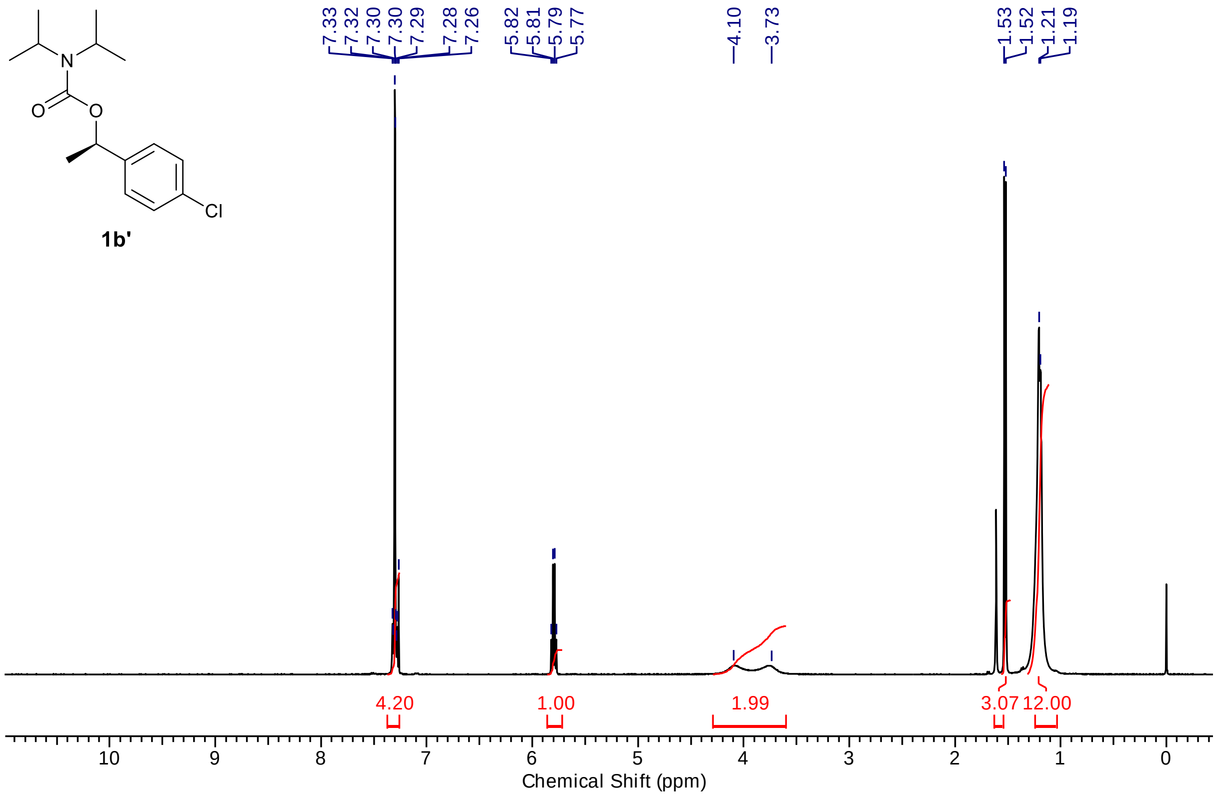

^{13}C-NMR (100 MHz, $CDCl_3$):

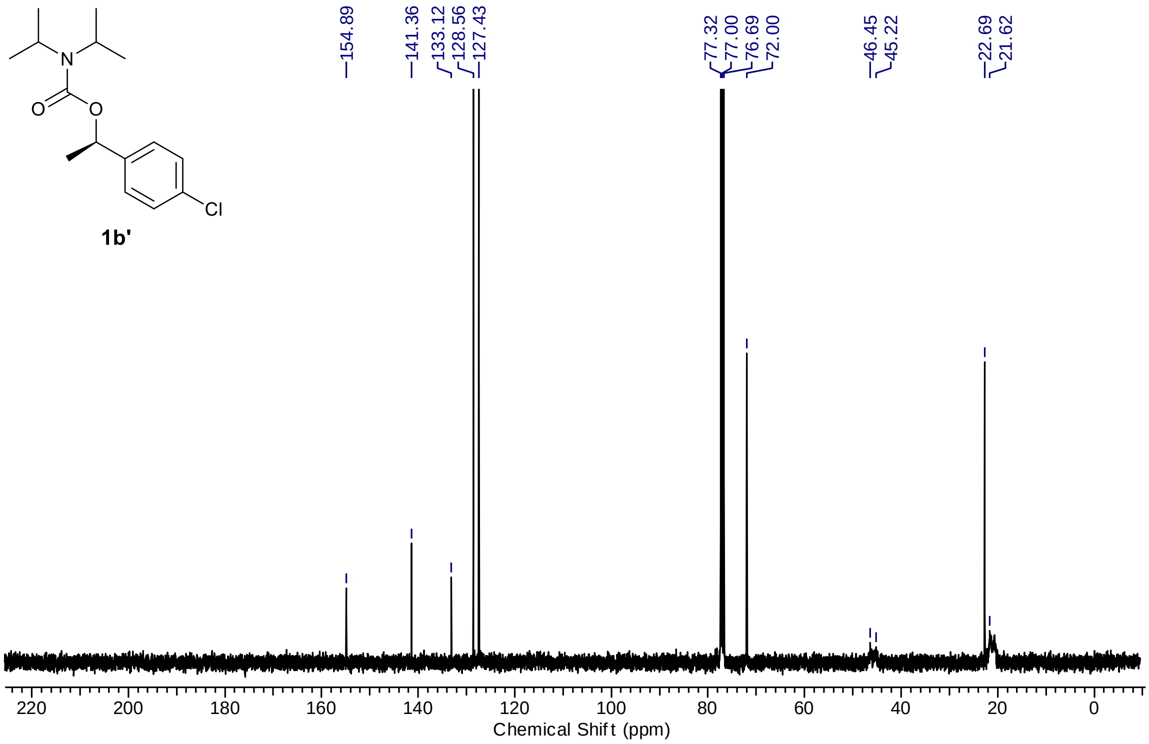

(*S*)-1-(3-Methoxyphenyl)ethyl diisopropylcarbamate (1d)

^{1}H-NMR (400 MHz, $CDCl_3$):

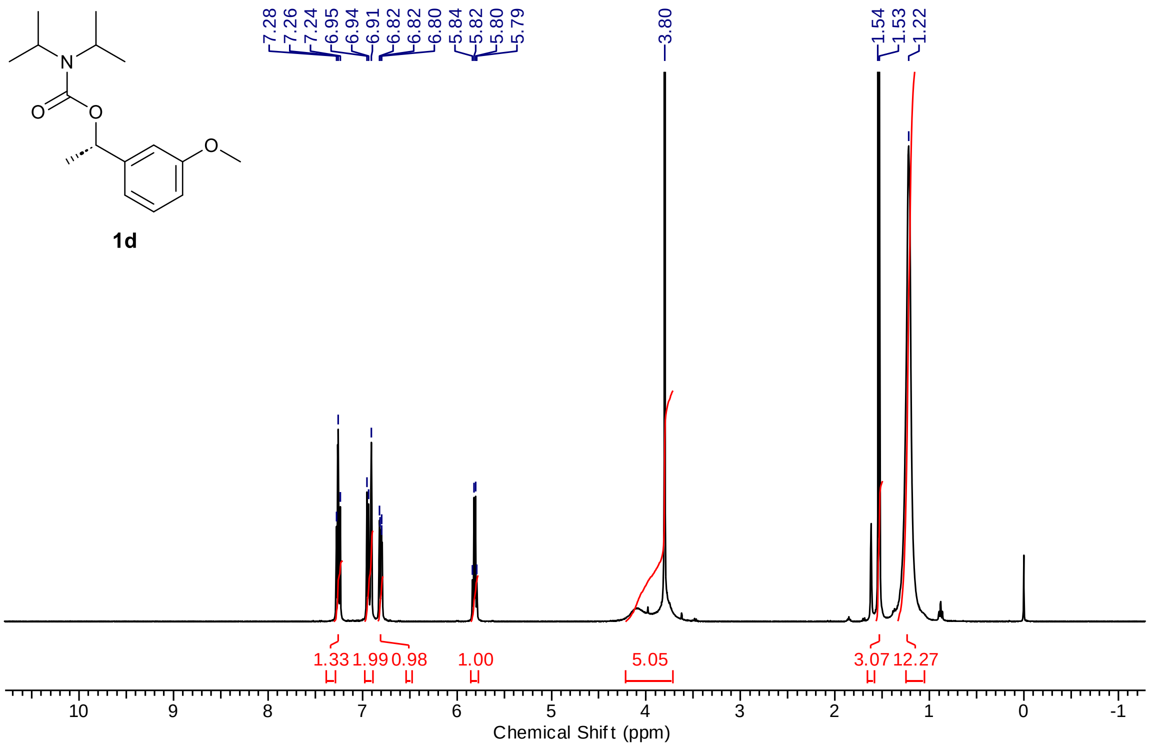

^{13}C-NMR (100 MHz, $CDCl_3$):

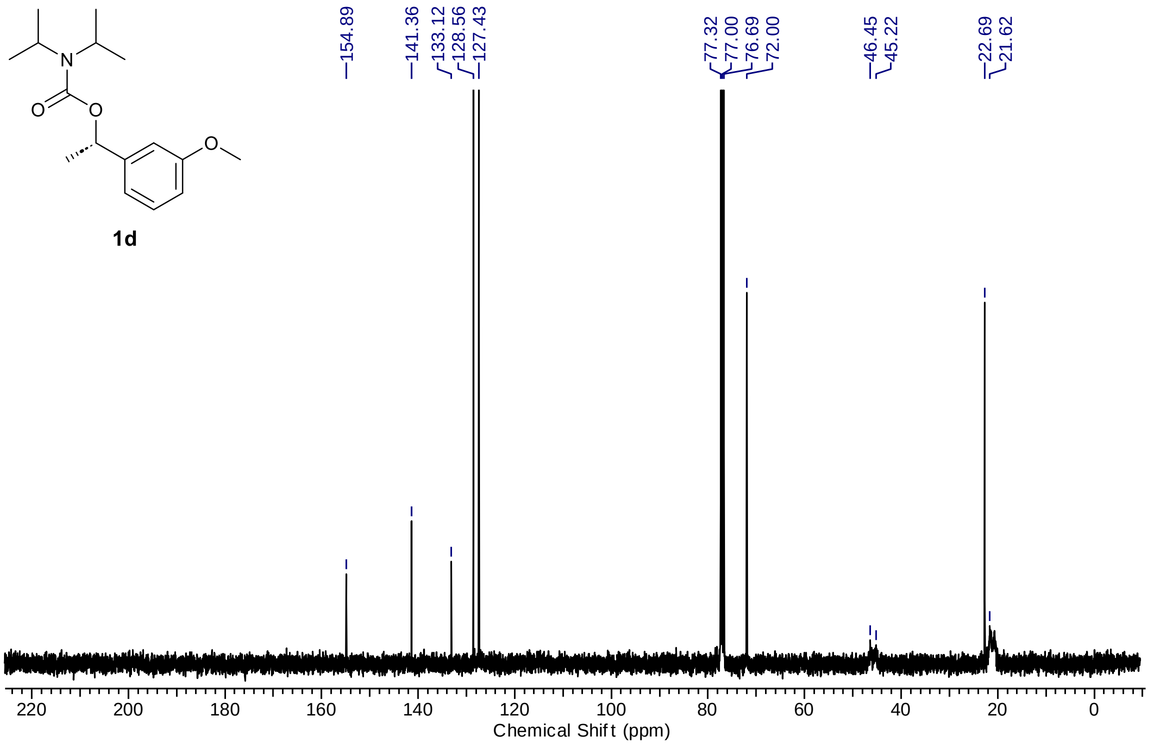

(4*S*,5*S*)-4,5-Dicyclohexyl-2-((1*S*,3*S*)-3-methyl-1-phenylhexyl)-1,3,2-dioxaborolane (1h-*1*)

^{1}H-NMR (400 MHz, $CDCl_3$):

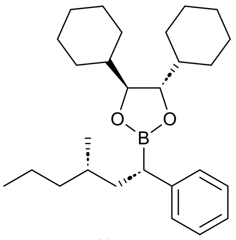

1h-*1*

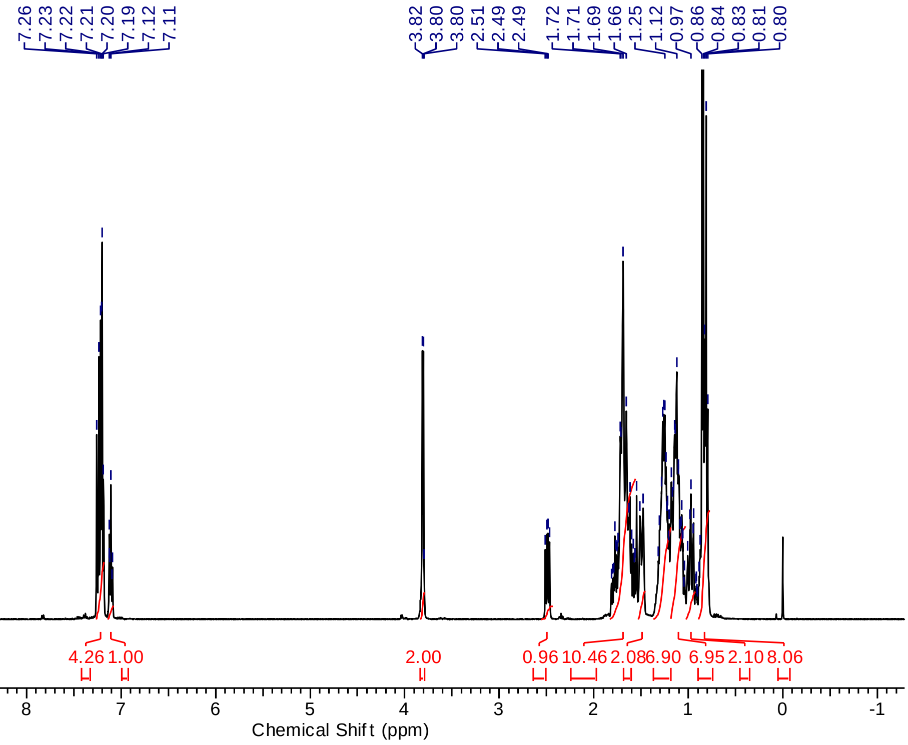

^{13}C-NMR (100 MHz, $CDCl_3$):

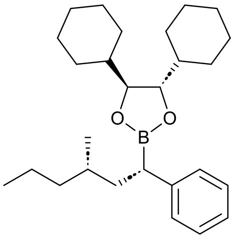

1h-*1*

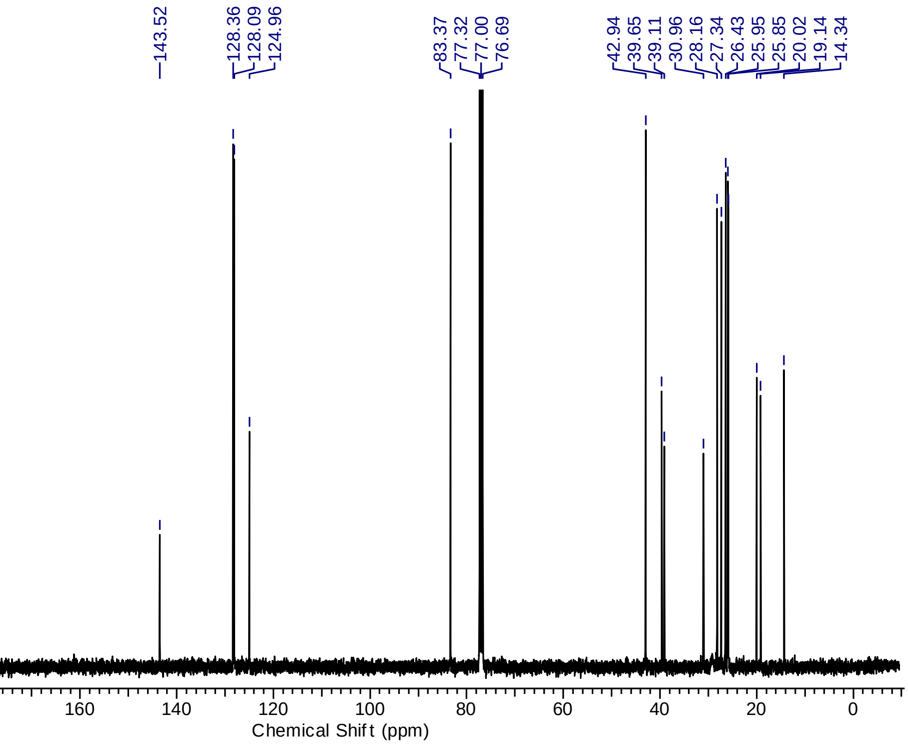

(1*S*,3*S*)-3-Methyl-1-phenylhexan-1-ol (1h-*2*)

^{1}H-NMR (400 MHz, $CDCl_3$):

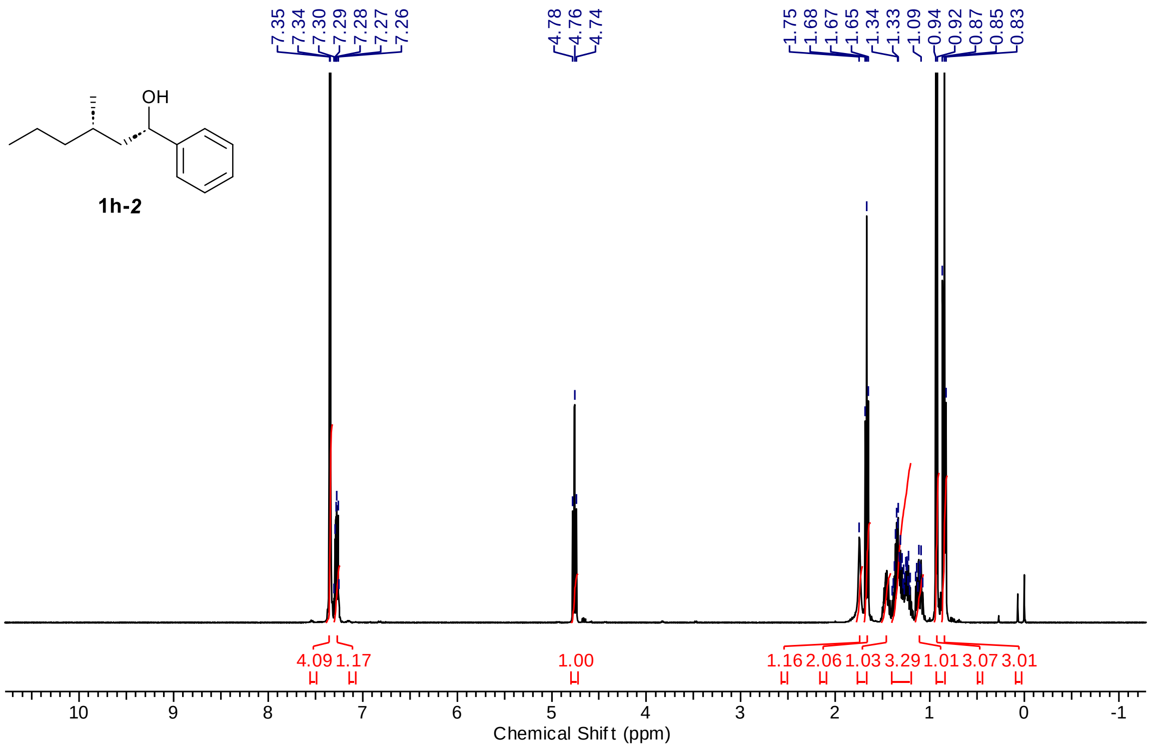

^{13}C-NMR (100 MHz, $CDCl_3$):

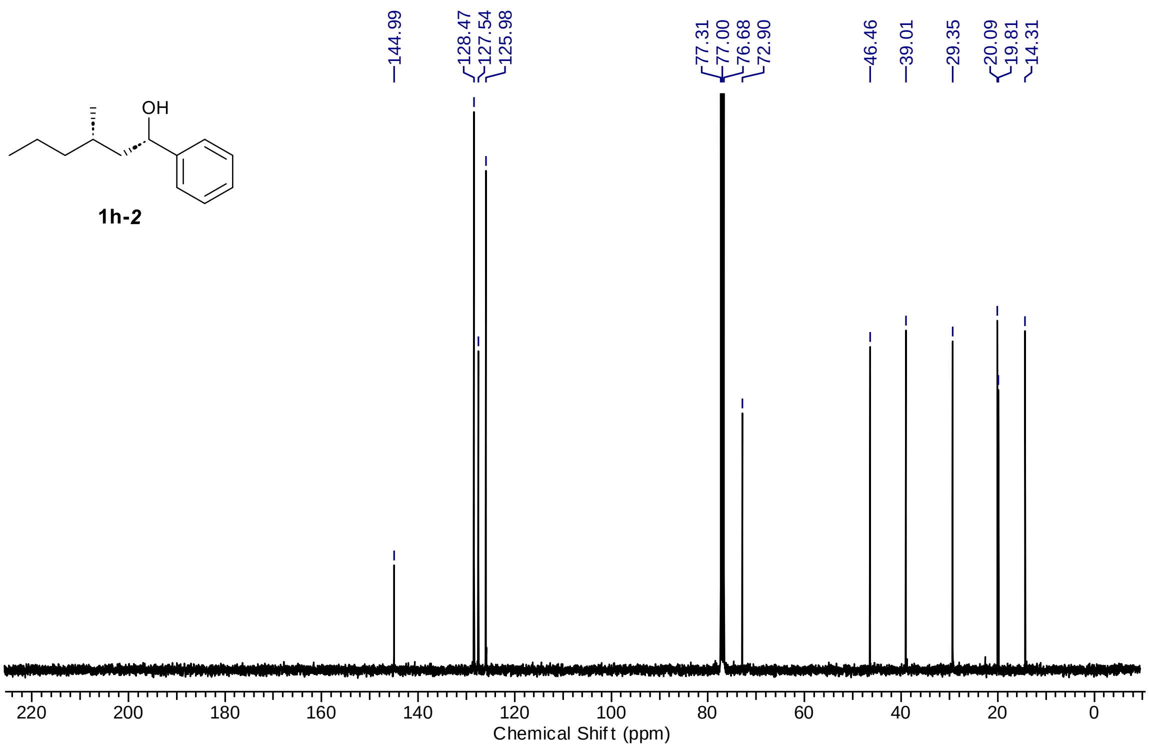

$^{1}H,^{1}H$-COSY

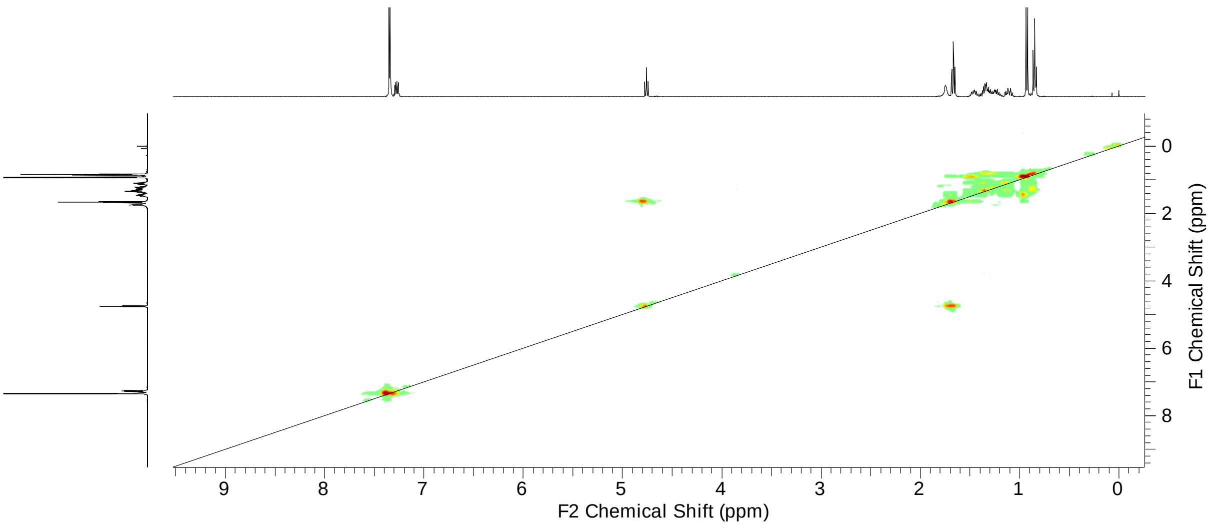

$^{1}H,^{13}C$-HSQC

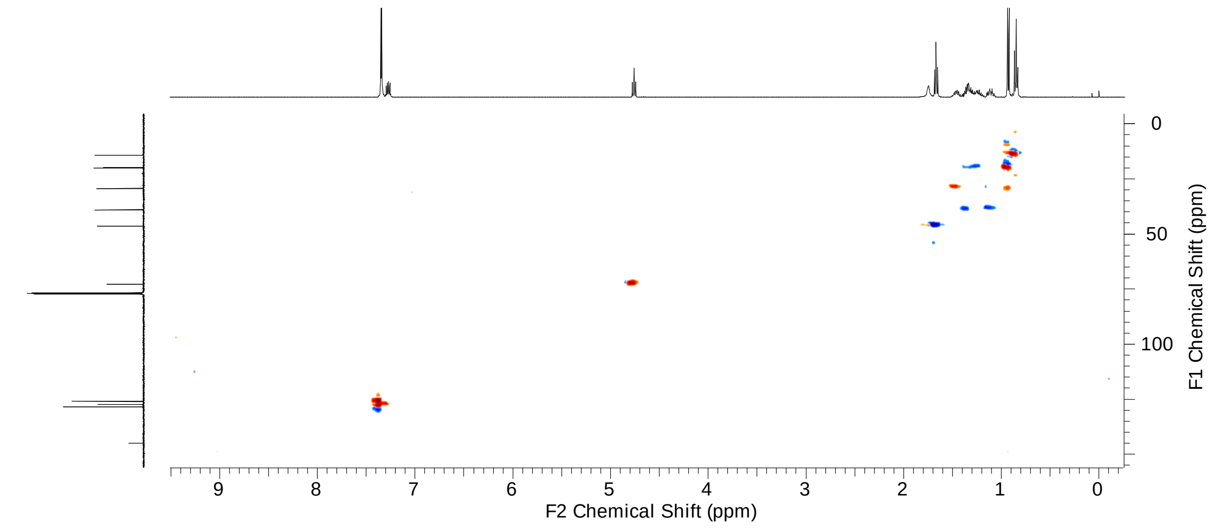

$^{1}H,^{13}C$-HMBC

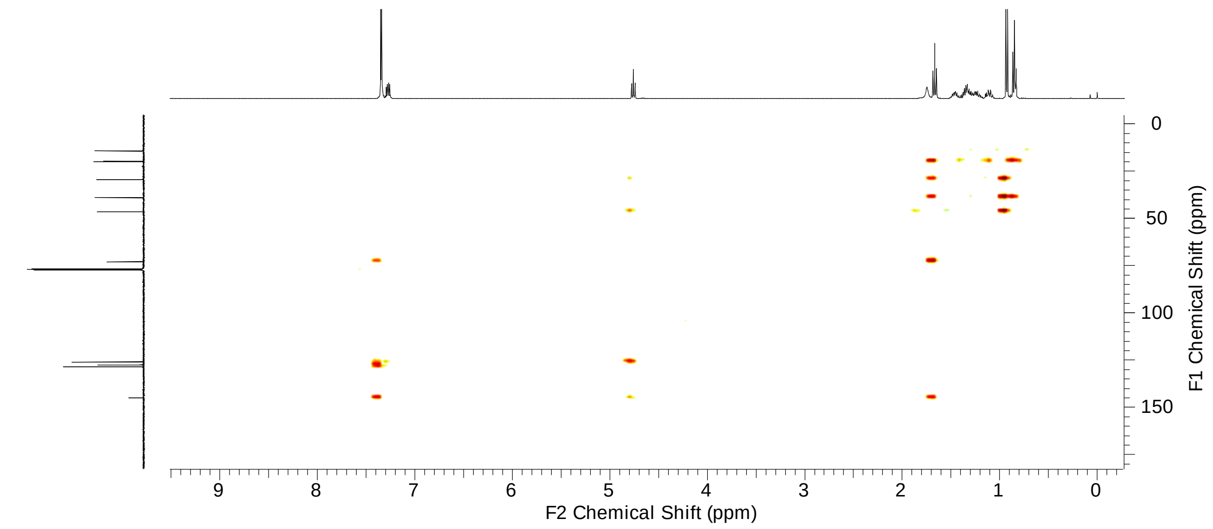

(1*S*,3*S*)-3-Methyl-1-phenylhexyl diisopropylcarbamate (1h)

^{1}H-NMR (500 MHz, $CDCl_3$):

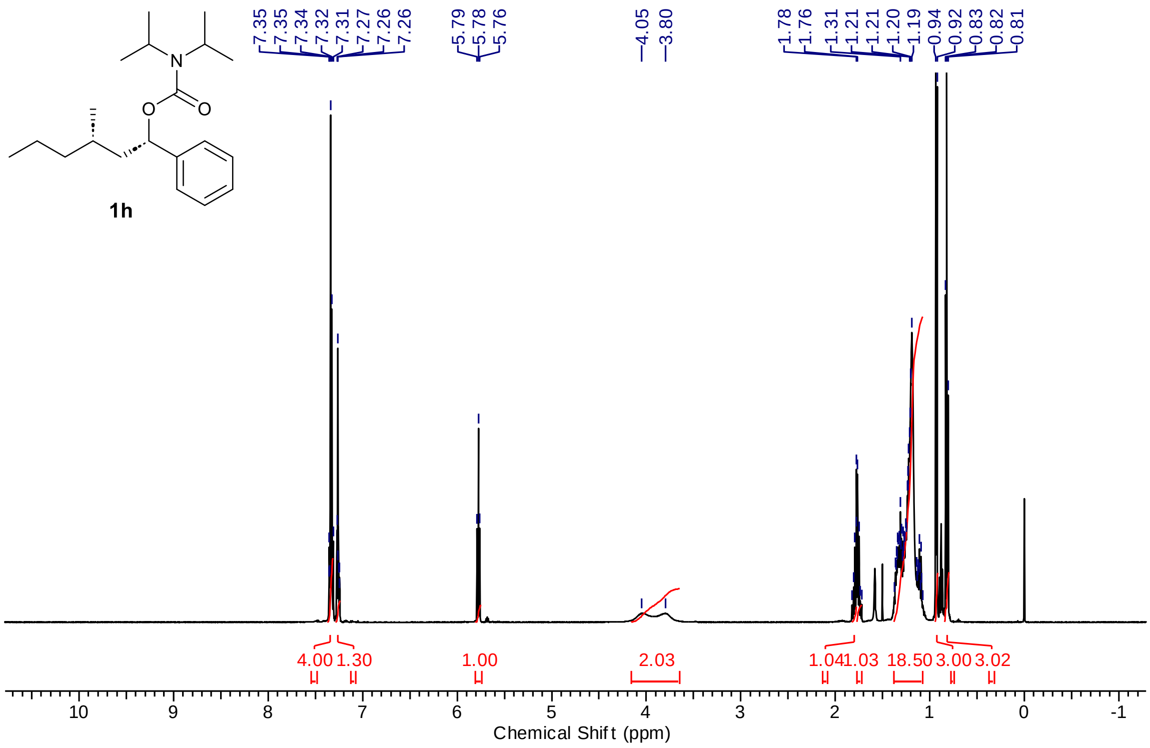

^{13}C-NMR (125 MHz, $CDCl_3$):

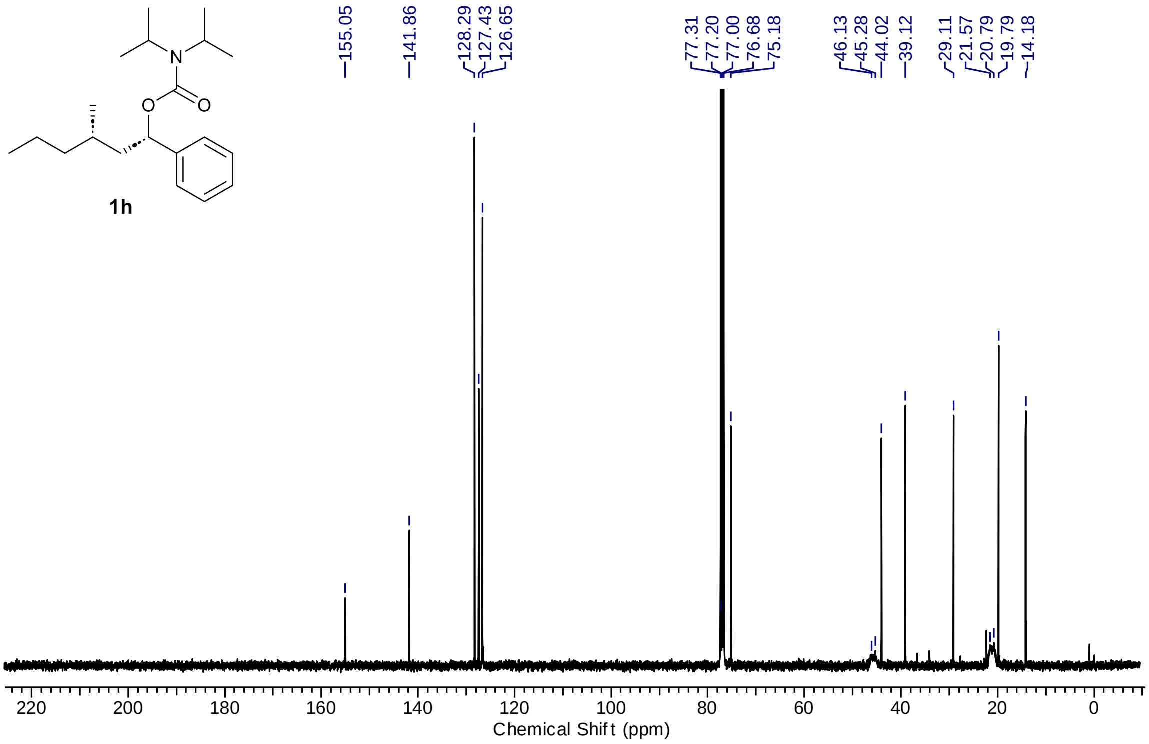

(4S,5S)-4,5-Dicyclohexyl-2-((S)-pentan-2-yl)-1,3,2-dioxaborolane (2a)

^{1}H-NMR (400 MHz, $CDCl_3$):

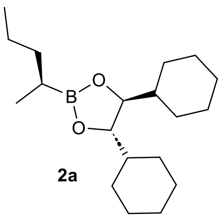

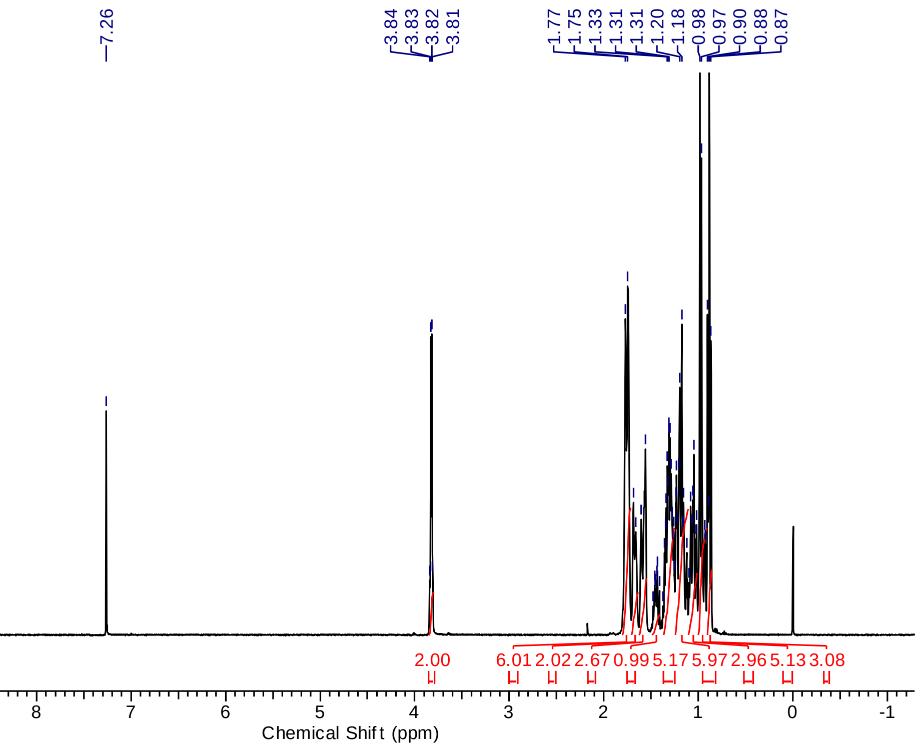

^{13}C-NMR (100 MHz, $CDCl_3$):

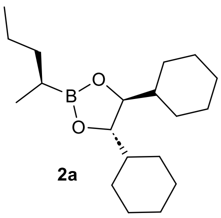

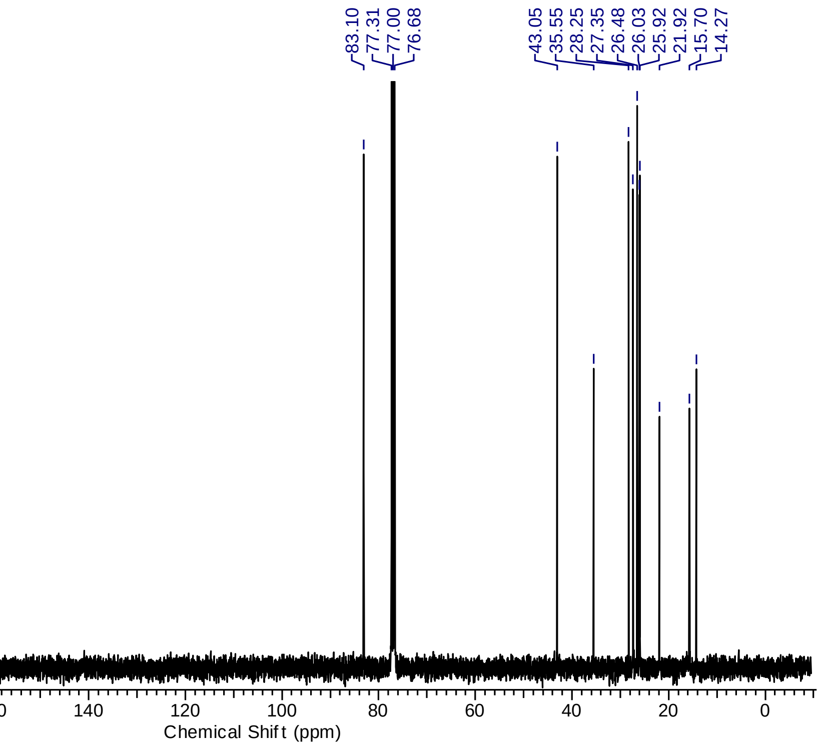

(4*S*,5*S*)-4,5-Dicyclohexyl-2-((*R*)-2-methylpentyl)-1,3,2-dioxaborolane (2b)

^{1}H-NMR (400 MHz, $CDCl_3$):

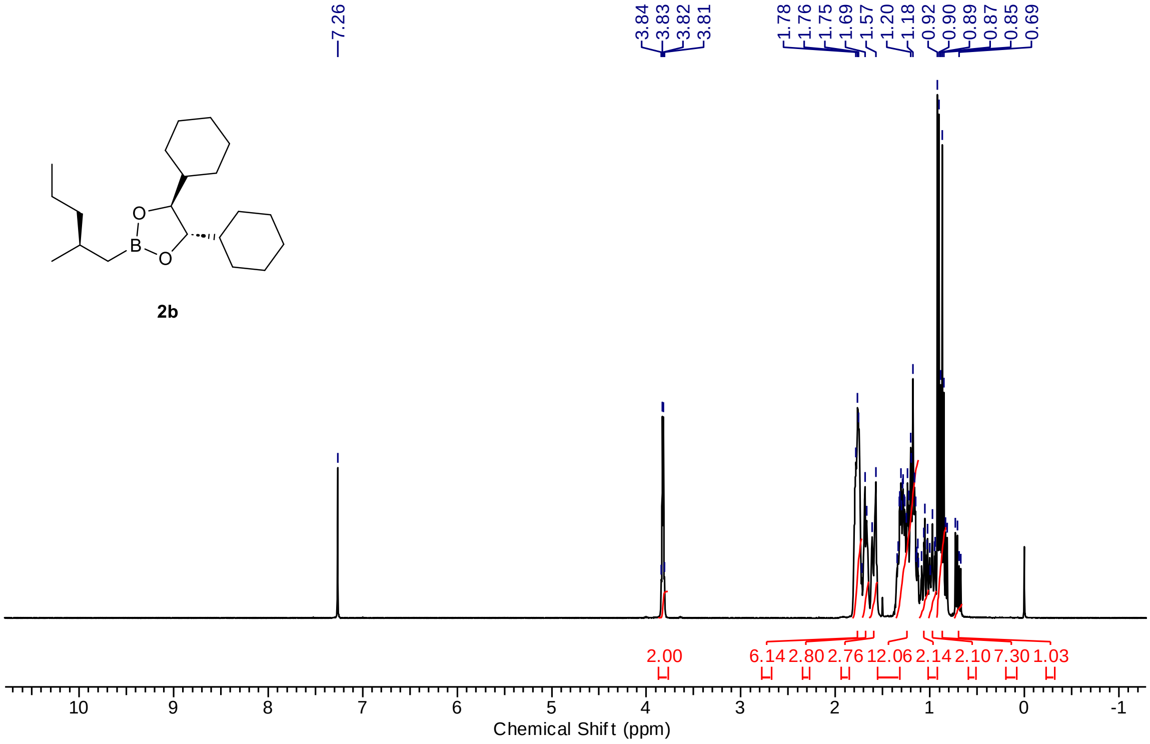

^{13}C-NMR (100 MHz, $CDCl_3$):

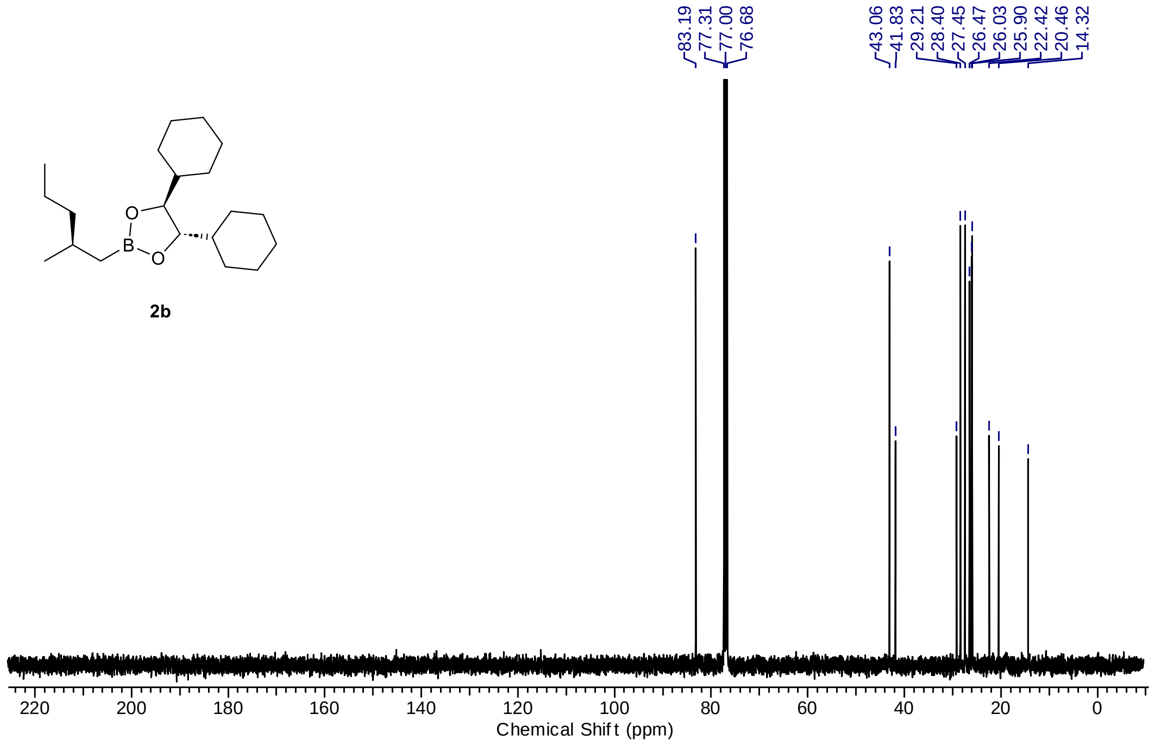

(4*S*,5*S*)-4,5-Dicyclohexyl-2-((*S*)-1-methoxyethyl)-1,3,2-dioxaborolane (2d)

^{1}H-NMR (400 MHz, $CDCl_3$):

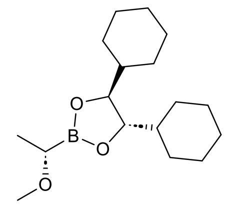

2d

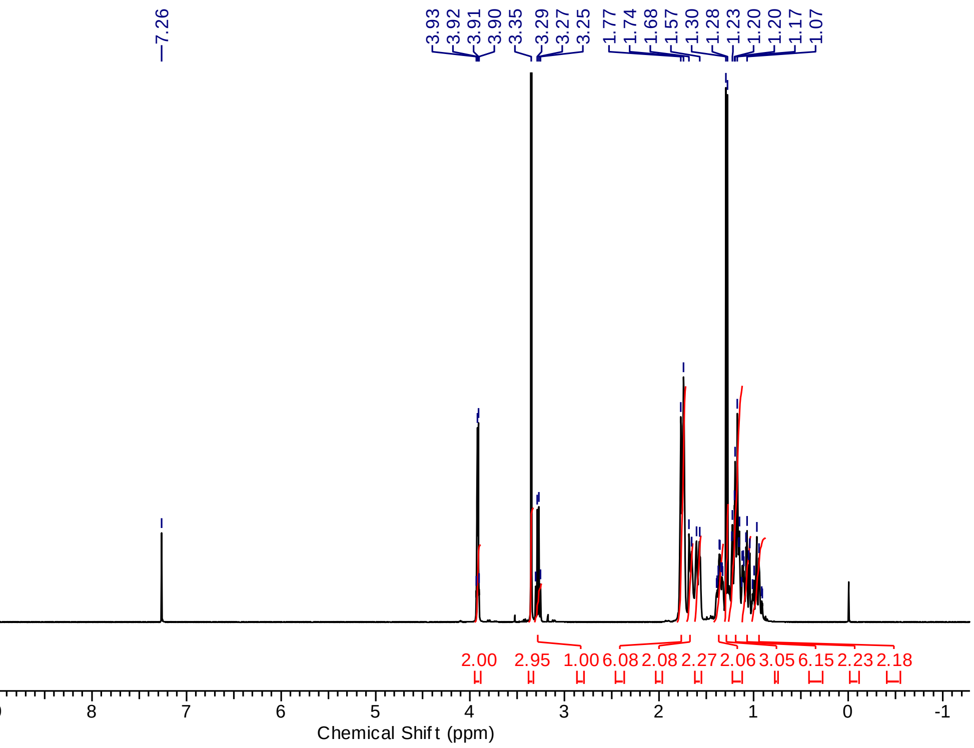

^{13}C-NMR (100 MHz, $CDCl_3$):

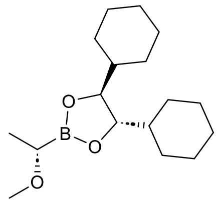

2d

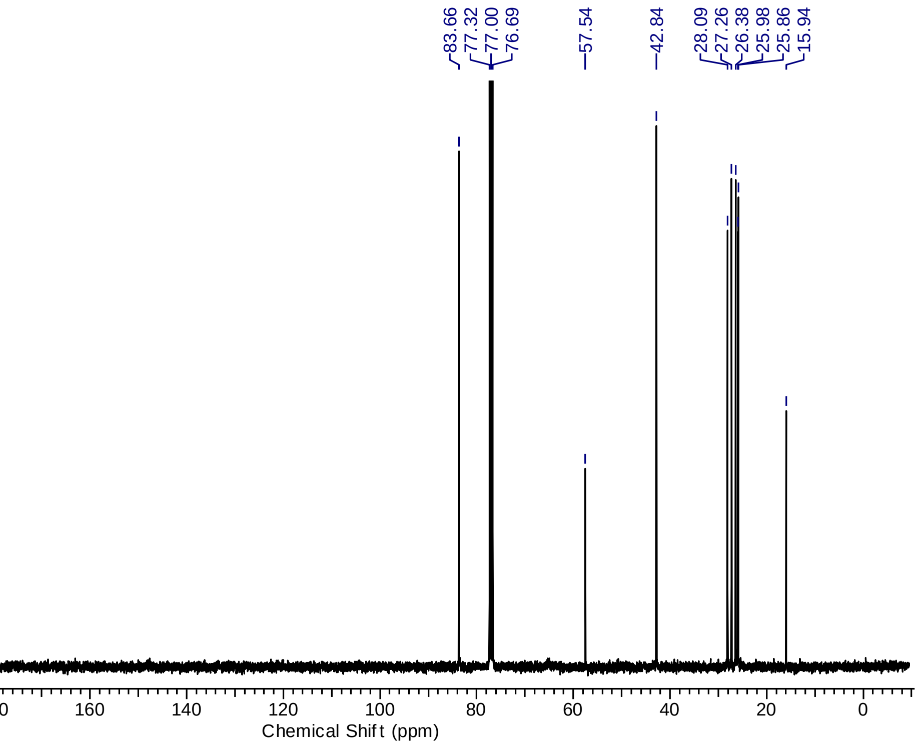

(4*R*,5*R*)-4,5-Dicyclohexyl-2-((*S*)-2-((4-methoxybenzyl)oxy)propyl)-1,3,2-dioxaborolane (2e')

^{1}H-NMR (400 MHz, $CDCl_3$):

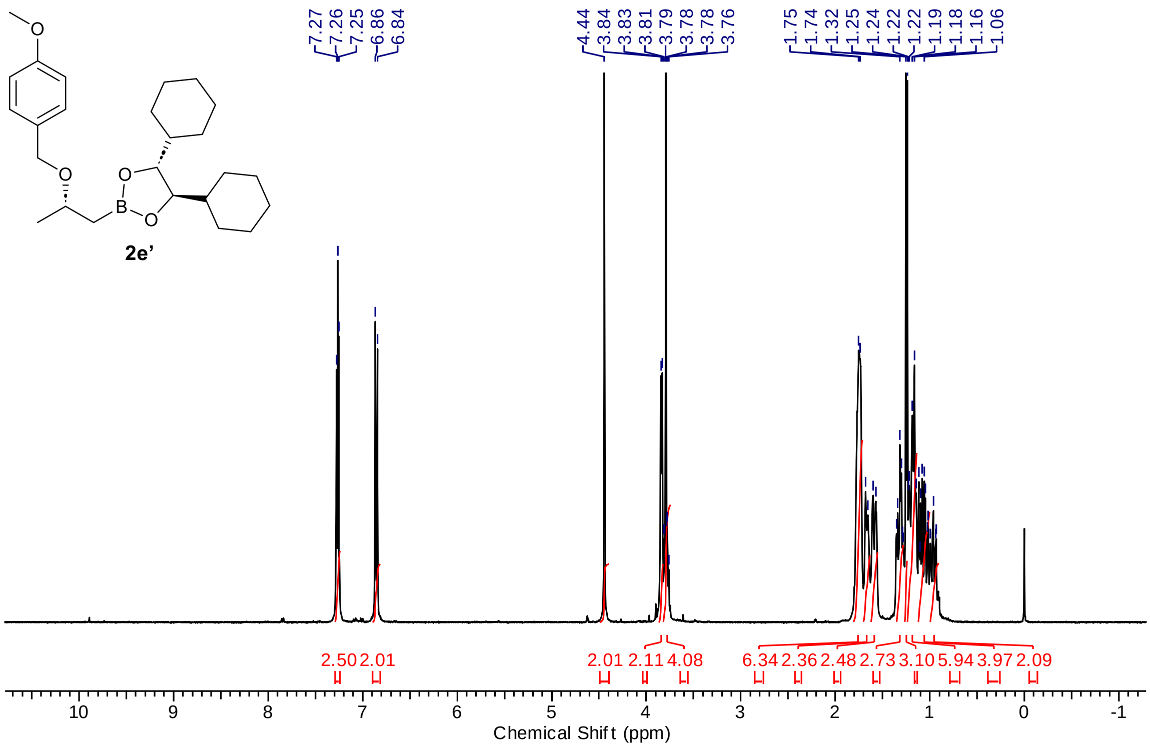

^{13}C-NMR (100 MHz, $CDCl_3$):

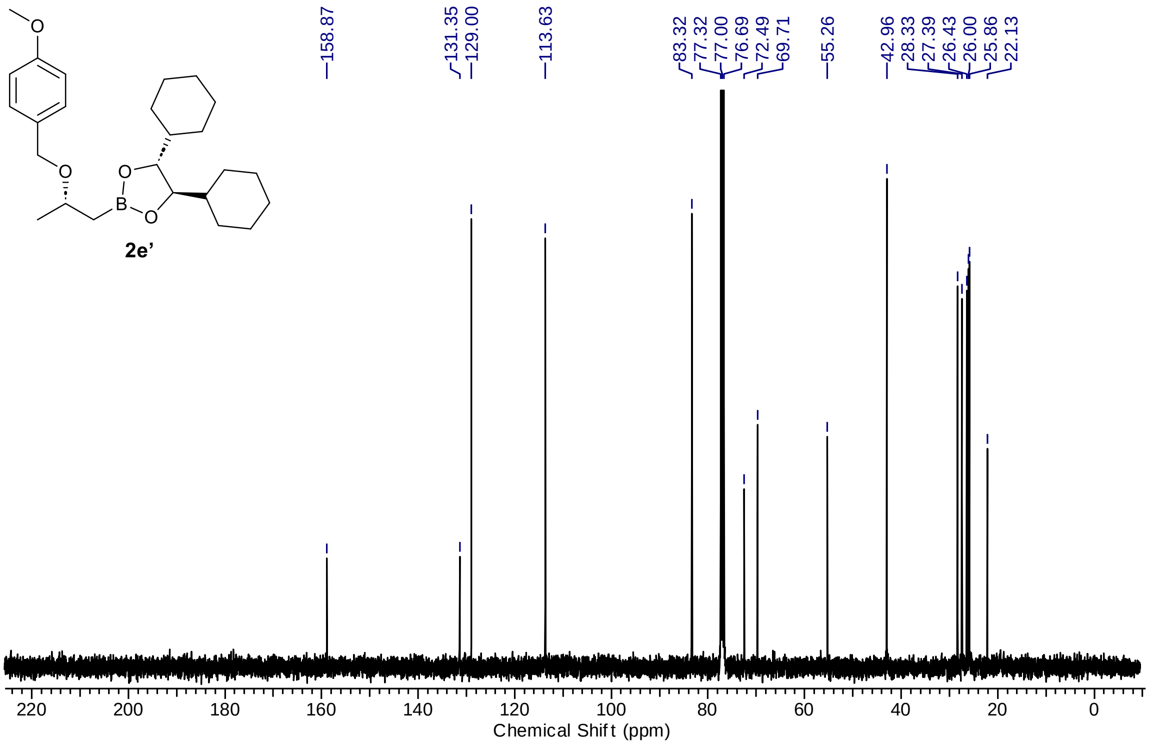

(4*R*,5*R*)-4,5-Dicyclohexyl-2-((2*S*,3*S*)-3-((4-methoxybenzyl)oxy)butan-2-yl)-1,3,2-dioxaborolane (2f')

^{1}H-NMR (400 MHz, $CDCl_3$):

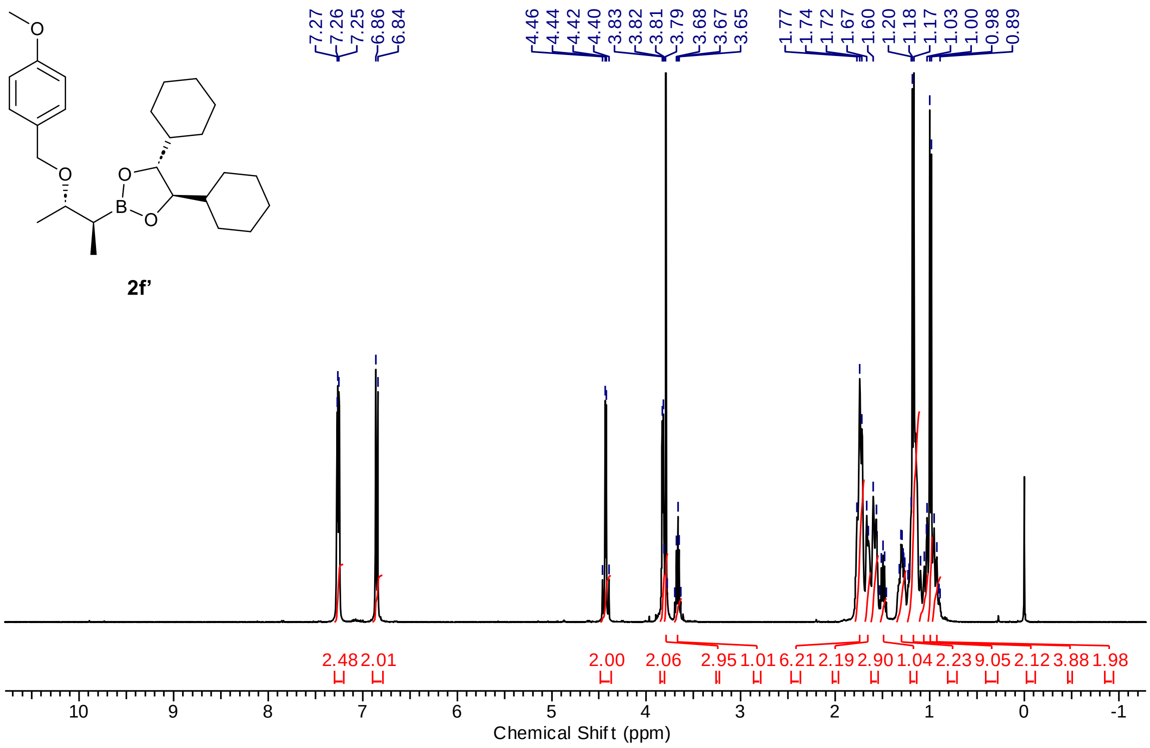

^{13}C-NMR (100 MHz, $CDCl_3$):

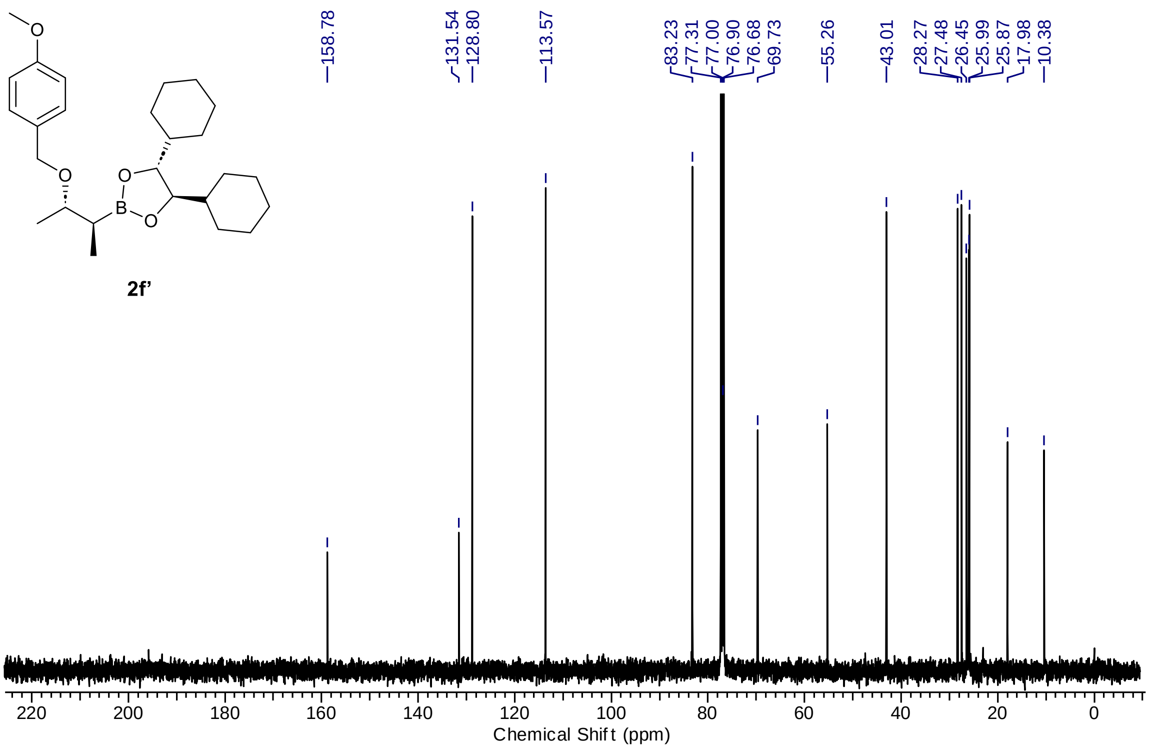

2-(2-((4-Methoxybenzyl)oxy)ethyl)-4,4,5,5-tetramethyl-1,3,2-dioxaborolane (2g-*2*)

[1]**H-NMR** (400 MHz, $CDCl_3$):

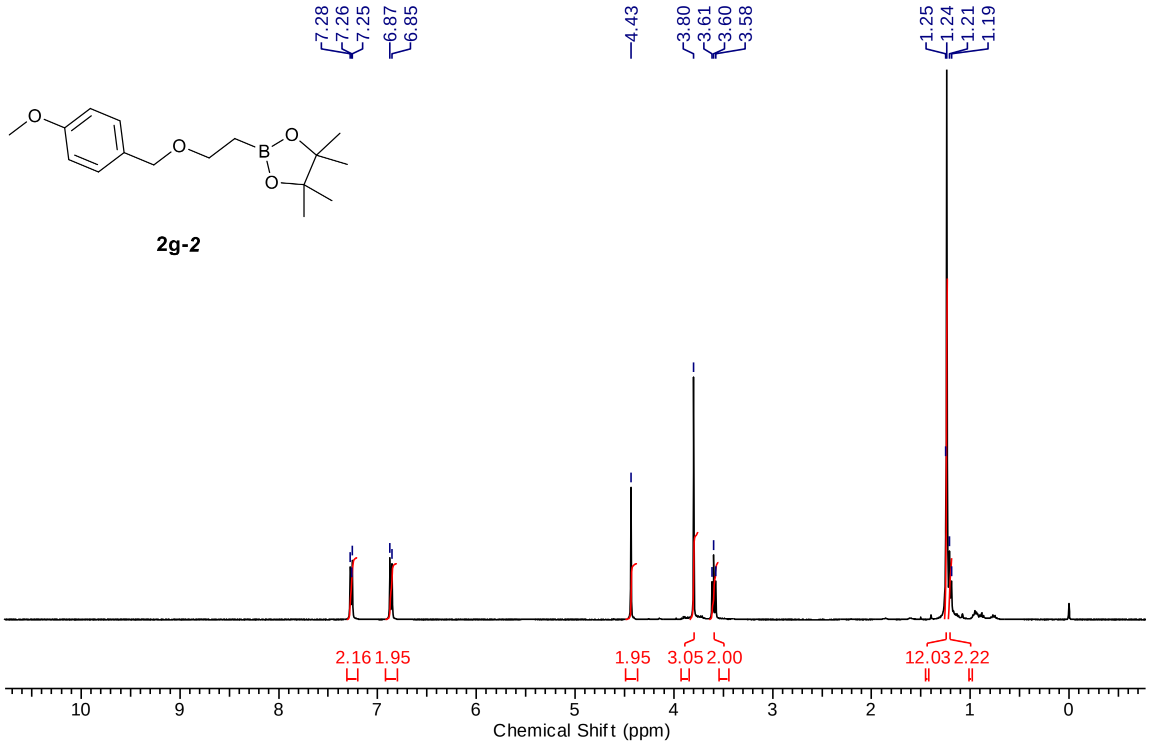

[13]**C-NMR** (100 MHz, $CDCl_3$):

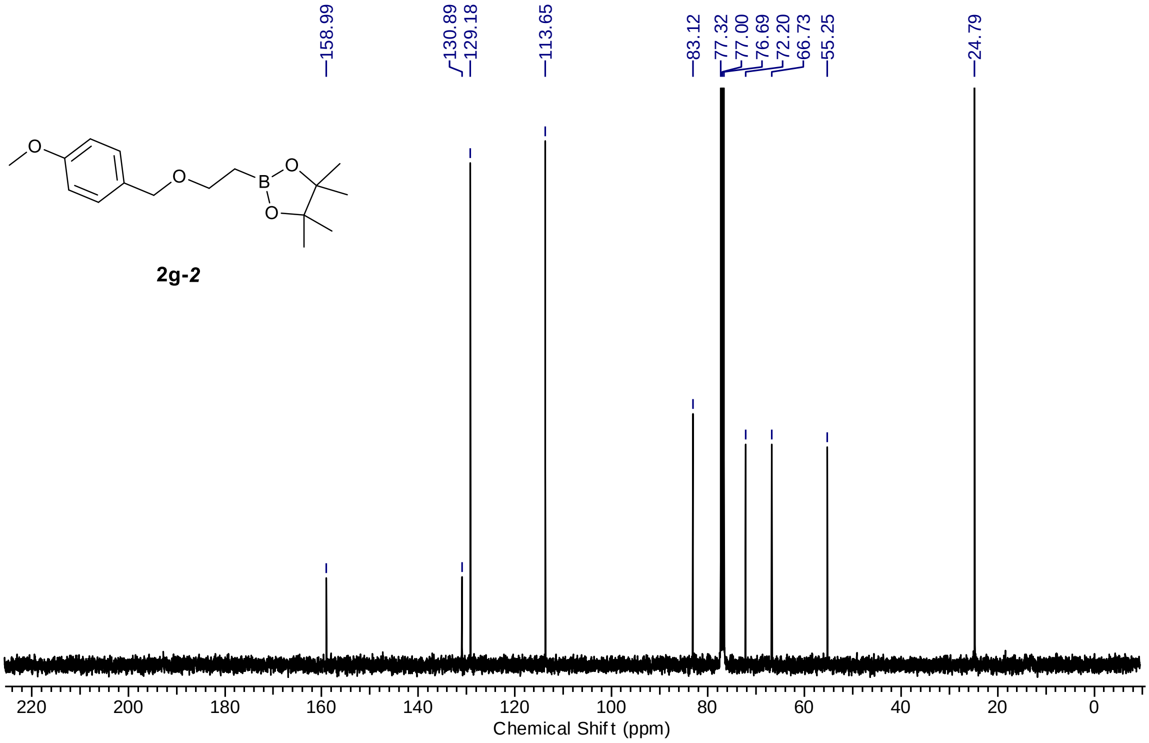

(4*S*,5*S*)-4,5-Dicyclohexyl-2-(2-((4-methoxybenzyl)oxy)ethyl)-1,3,2-dioxaborolane (2g-*3*)

^{1}H-NMR (400 MHz, $CDCl_3$):

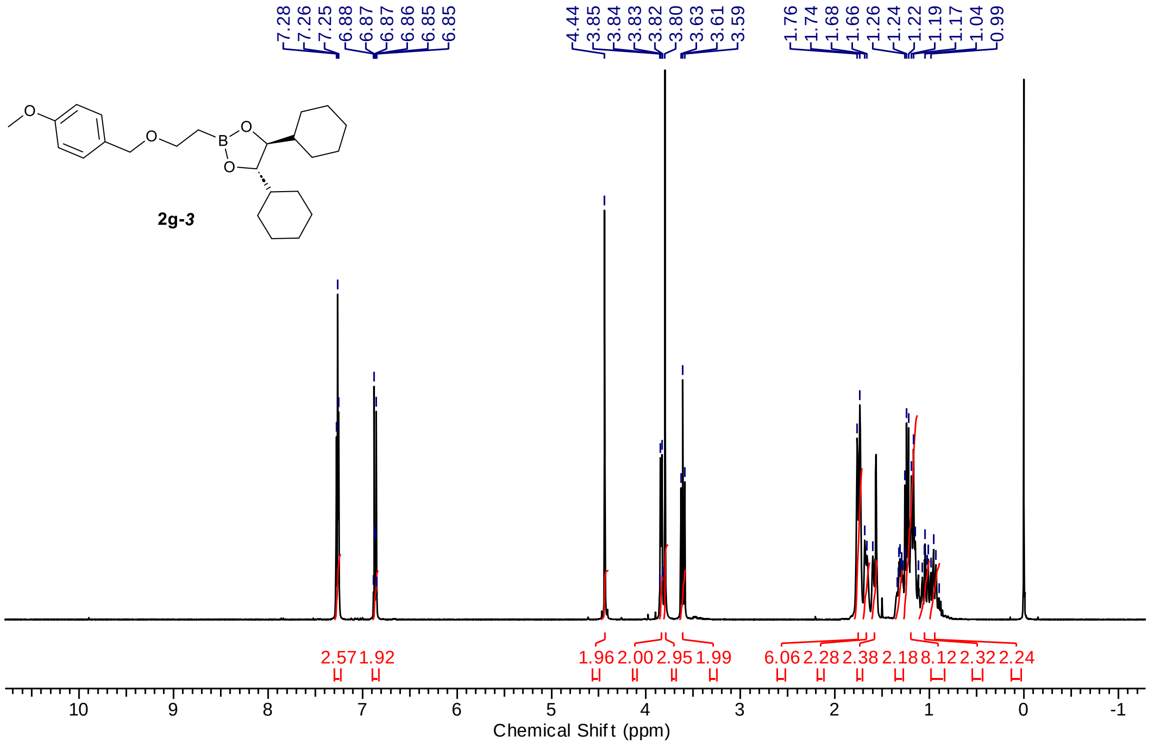

^{13}C-NMR (100 MHz, $CDCl_3$):

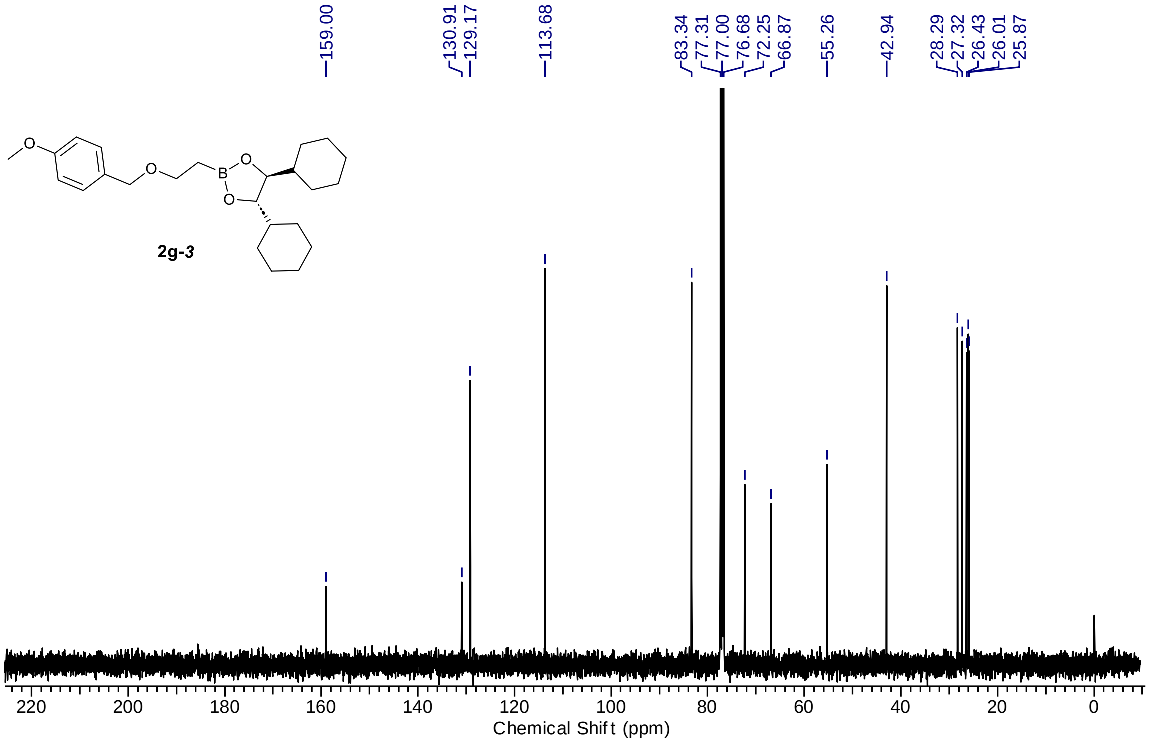

(4*S*,5*S*)-4,5-Dicyclohexyl-2-((*R*)-1-((4-methoxybenzyl)oxy)hex-5-en-3-yl)-1,3,2-dioxaborolane (2g)

^{1}H-NMR (400 MHz, $CDCl_3$):

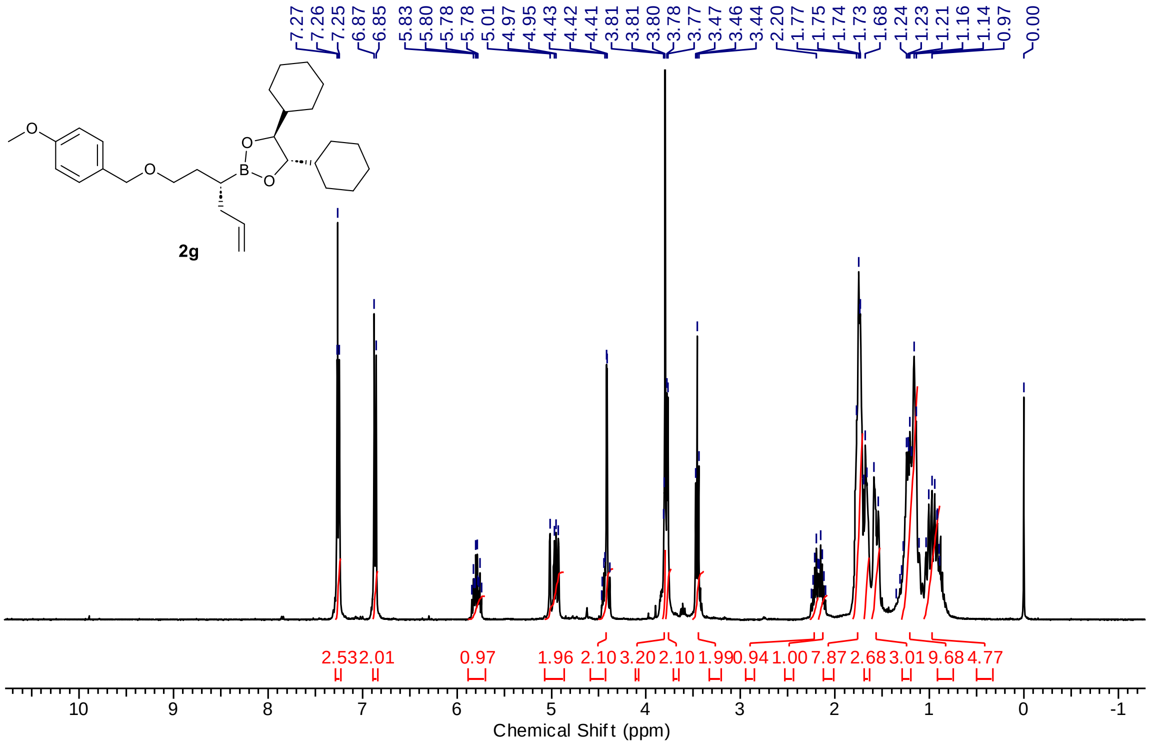

^{13}C-NMR (100 MHz, $CDCl_3$):

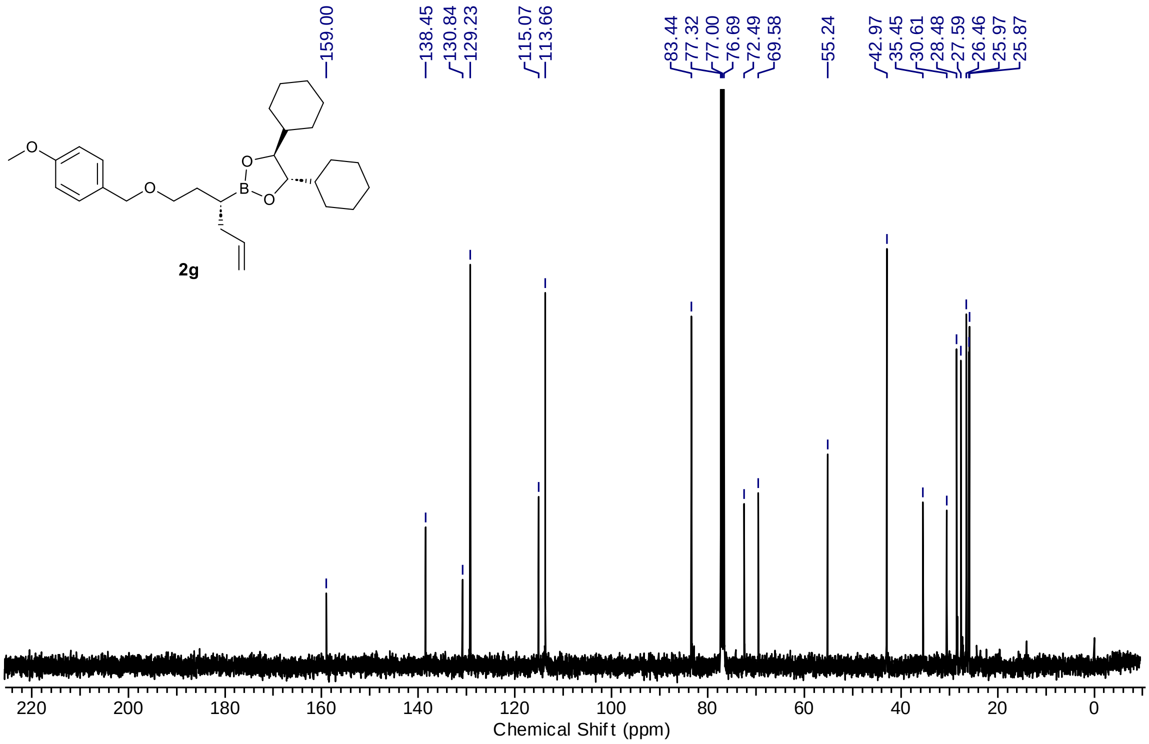

(2*R*,3*S*)-3-Methyl-2-phenylhexan-2-ol (3aa)

^{1}H-NMR (400 MHz, $CDCl_3$):

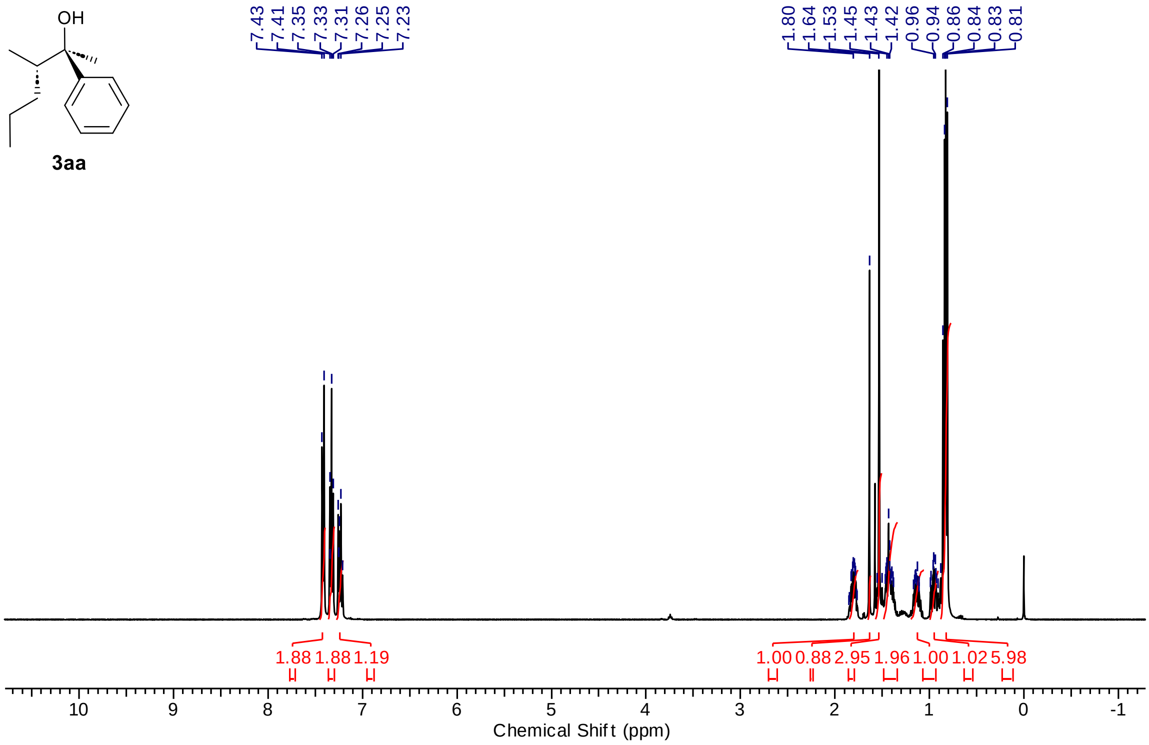

^{13}C-NMR (100 MHz, $CDCl_3$):

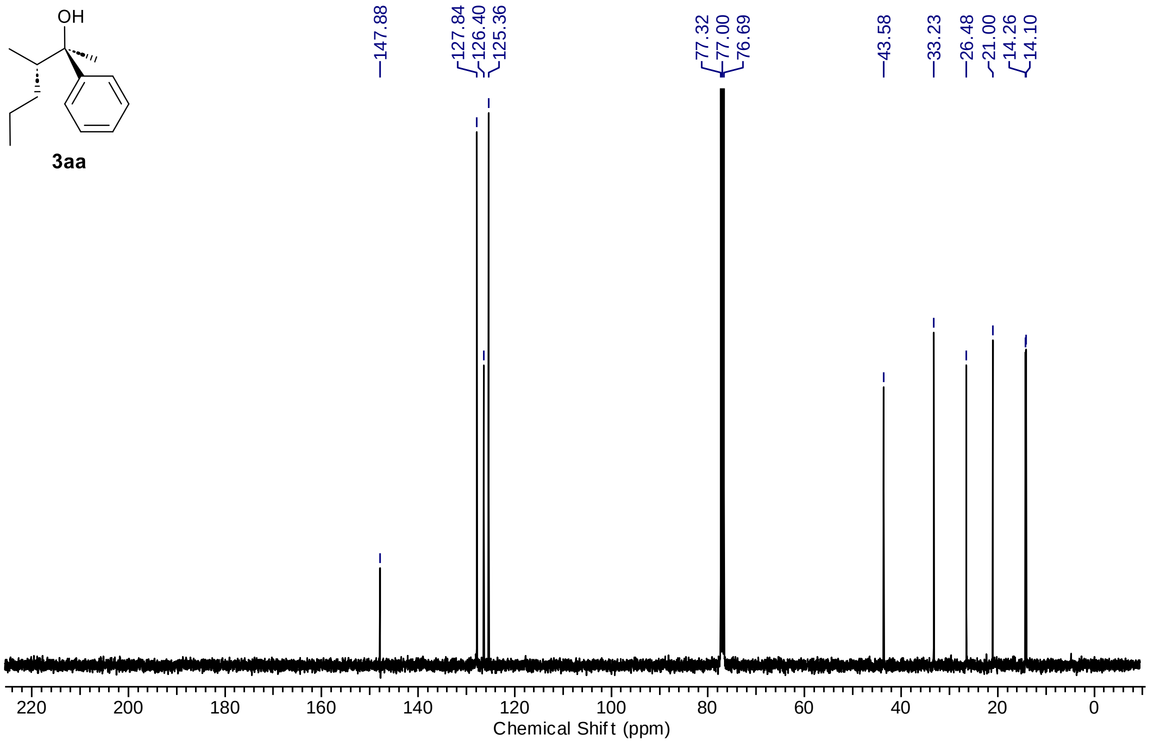

(2*S*,3*S*)-3-Methyl-2-phenylhexan-2-ol (3a’a):

^{1}H-NMR (400 MHz, $CDCl_3$):

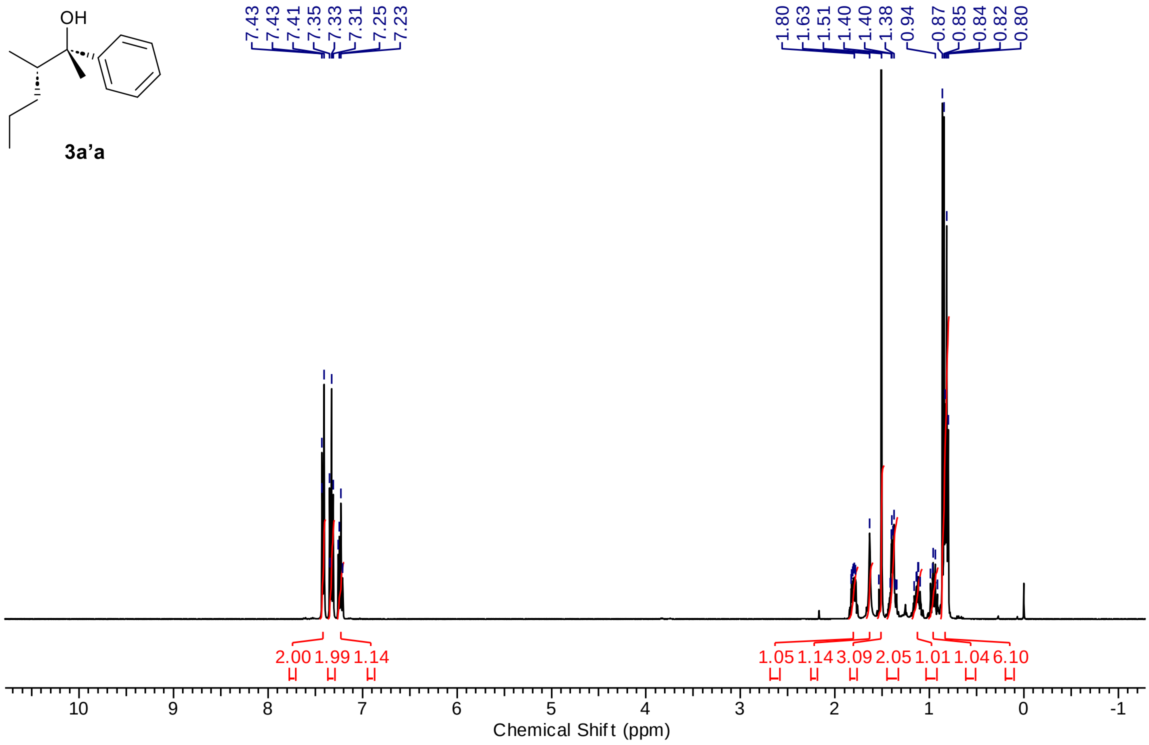

^{13}C-NMR (100 MHz, $CDCl_3$):

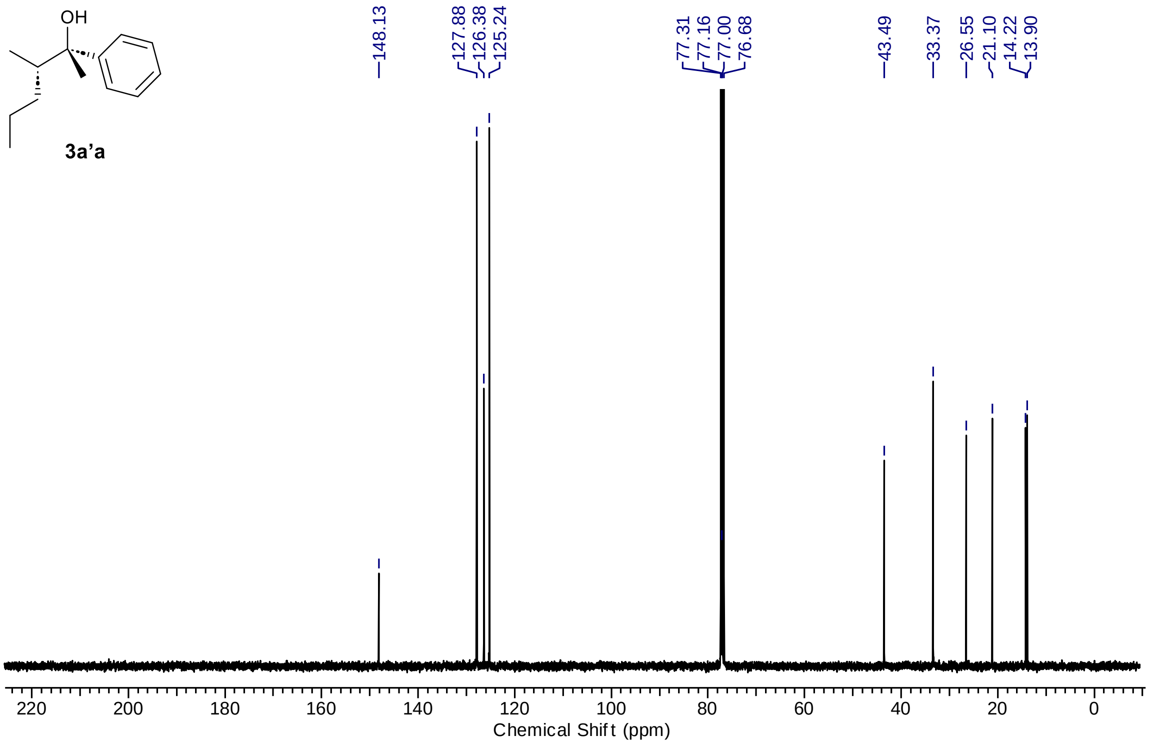

(2*S*,4*S*)-4-Methyl-2-phenylheptan-2-ol (3a'b):

^{1}**H-NMR** (400 MHz, $CDCl_3$):

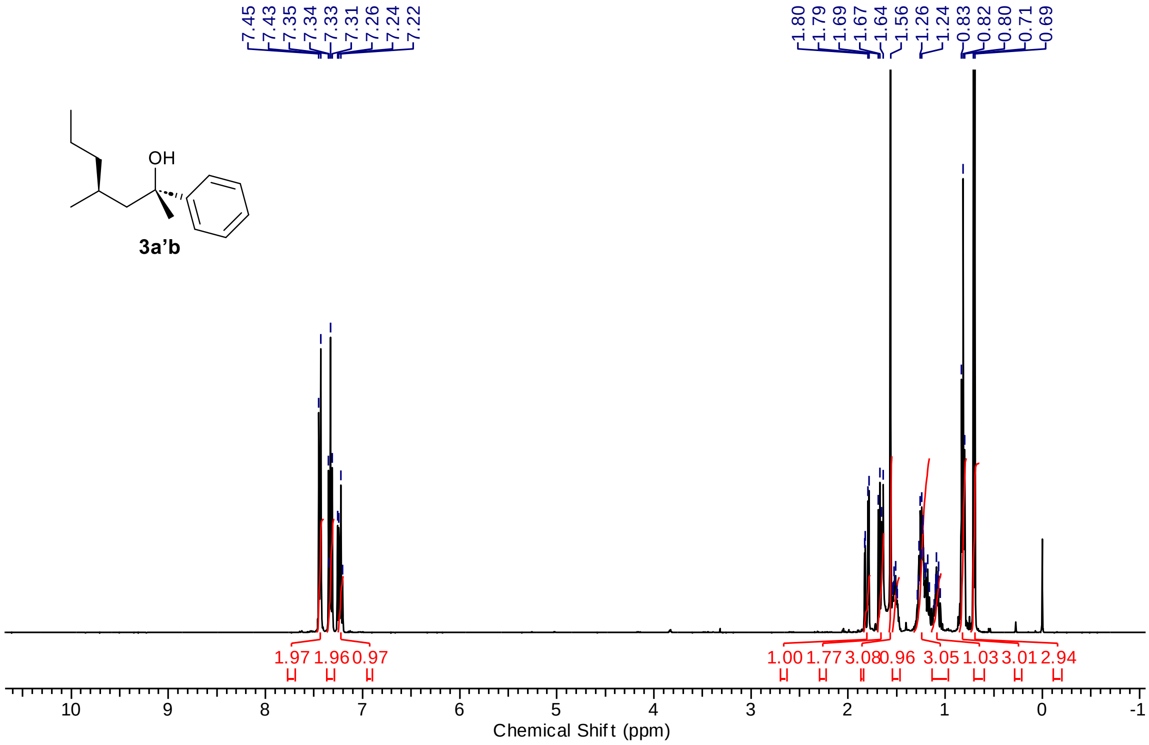

^{13}**C-NMR** (100 MHz, $CDCl_3$):

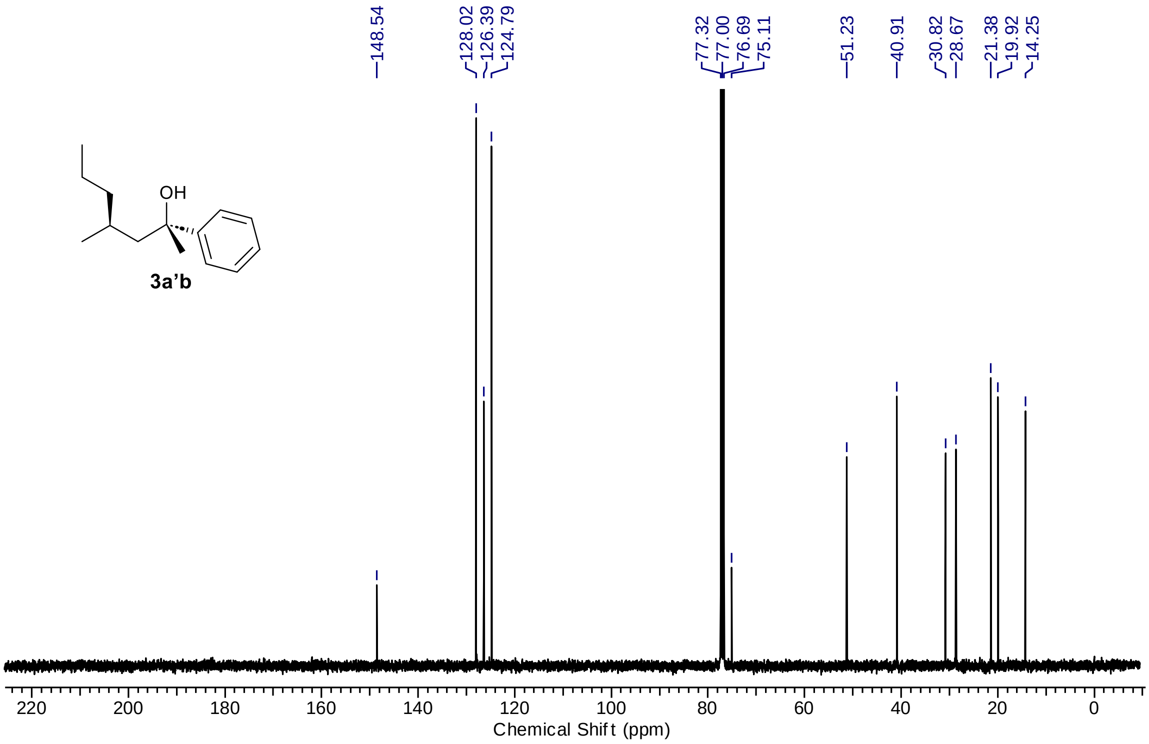

Diasteomers without use of $MgBr_2$: (2*S*,4*S*)-4-Methyl-2-phenylheptan-2-ol (3a'b) and (2*S*,4R)-4-Methyl-2-phenylheptan-2-ol:

^{1}H-NMR (400 MHz, $CDCl_3$):

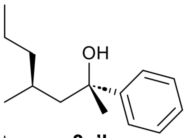

3a'b

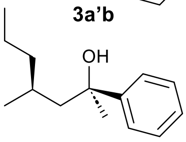

3a'b-dia

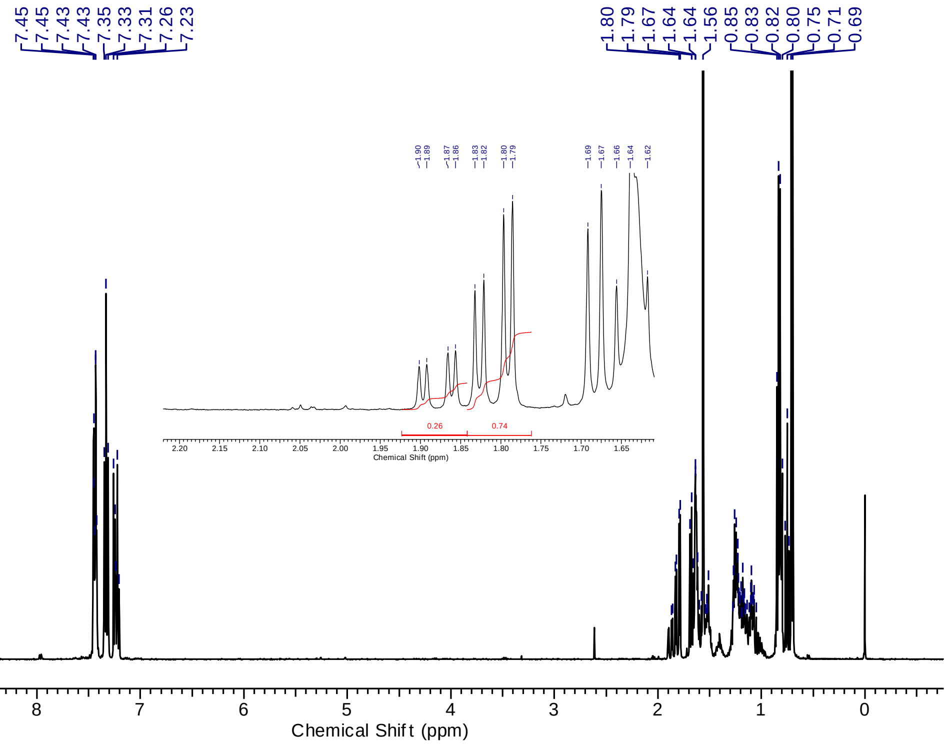

^{13}C-NMR (100 MHz, $CDCl_3$):

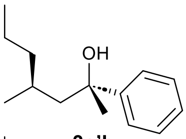

3a'b

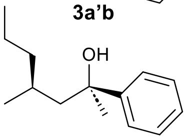

3a'b-dia

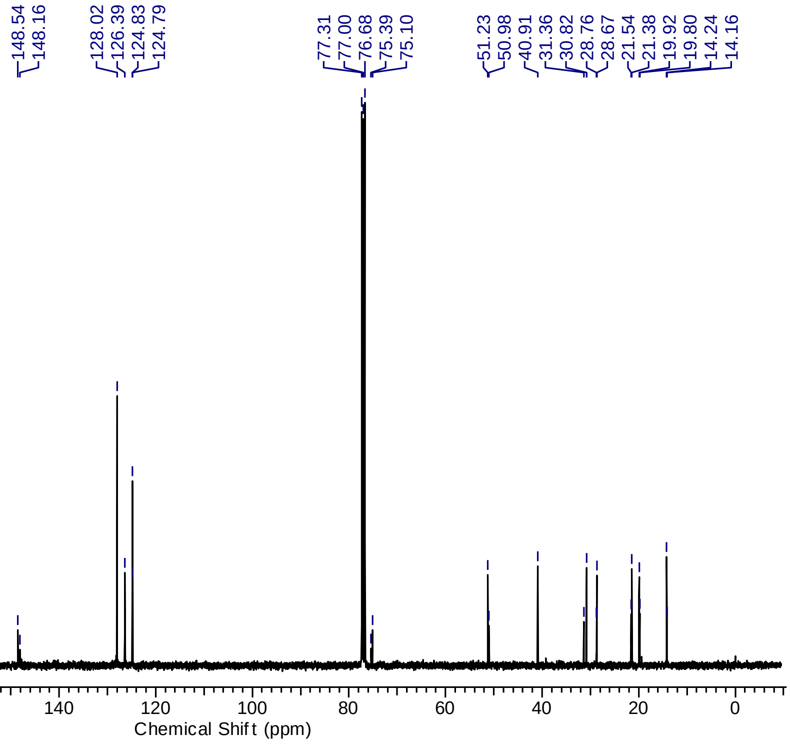

$^{1}H,^{1}H$-COSY

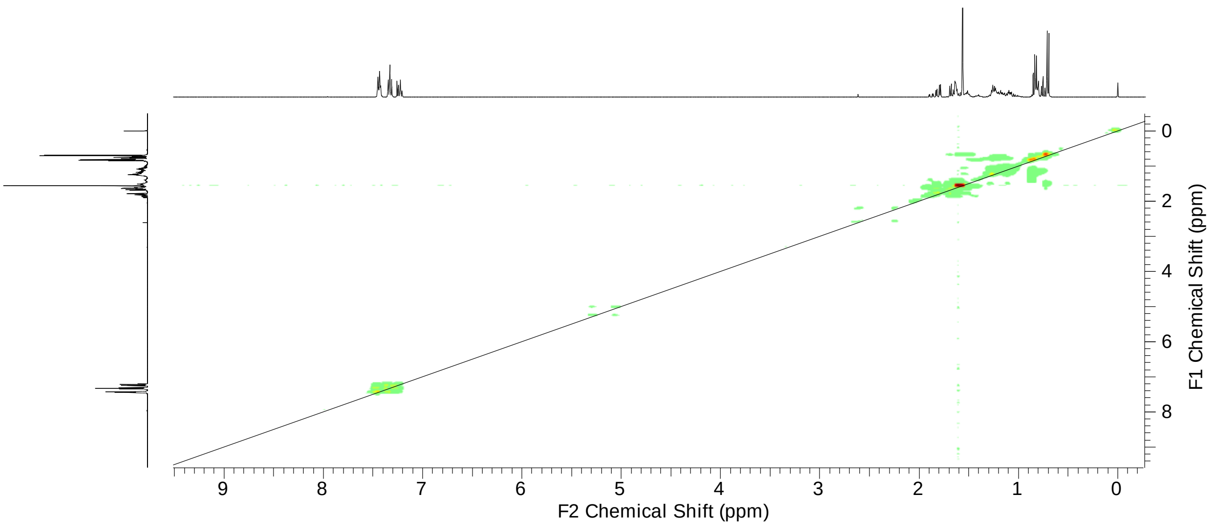

$^{1}H,^{13}C$-HSQC

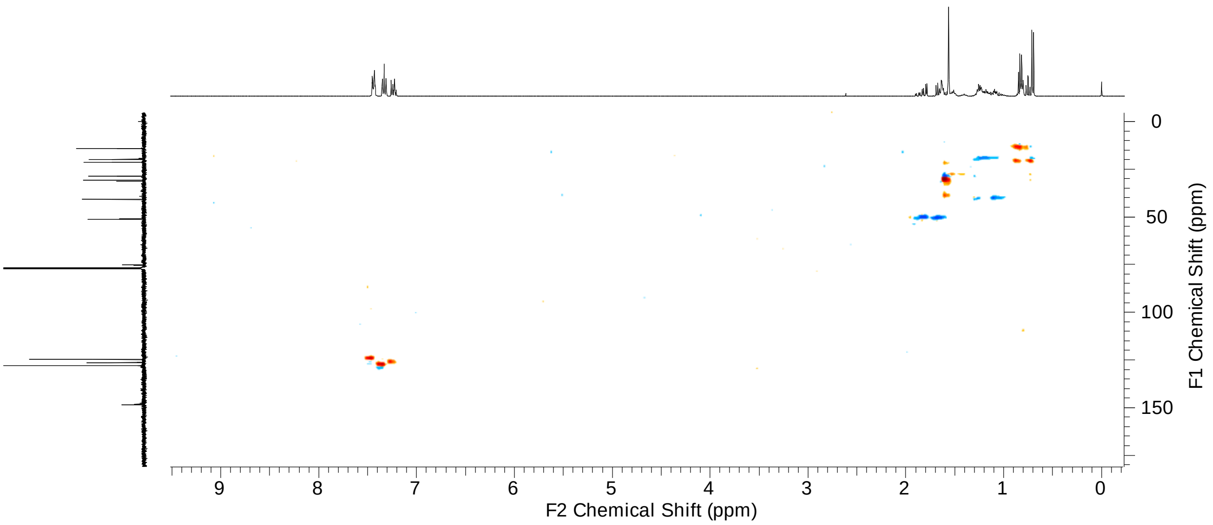

$^{1}H,^{13}C$-HMBC

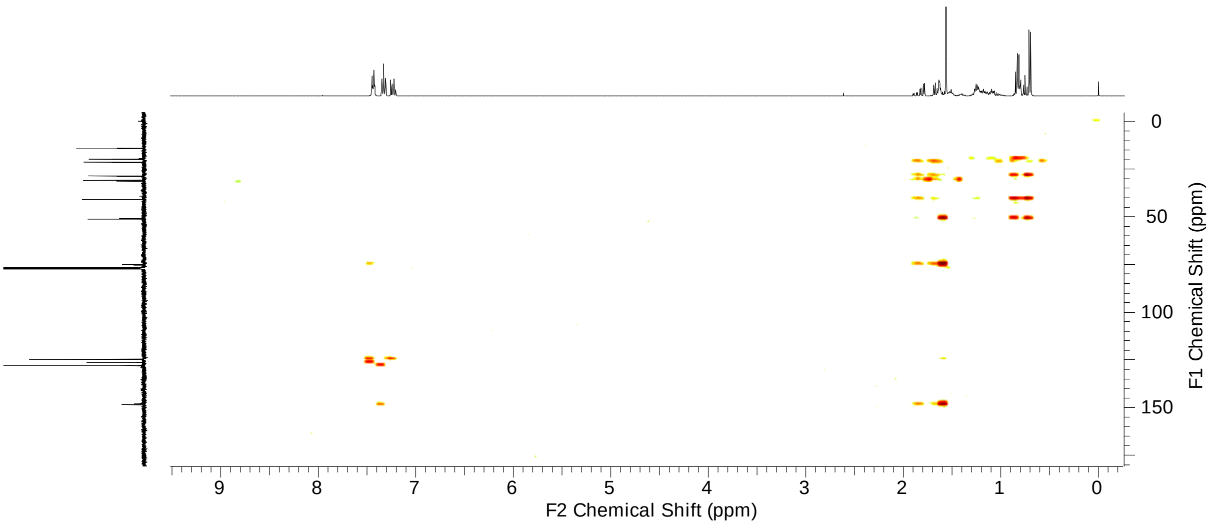

(2*R*,3*R*)-3-Methoxy-2-phenylbutan-2-ol (3a'd)

[1]**H-NMR** (400 MHz, $CDCl_3$):

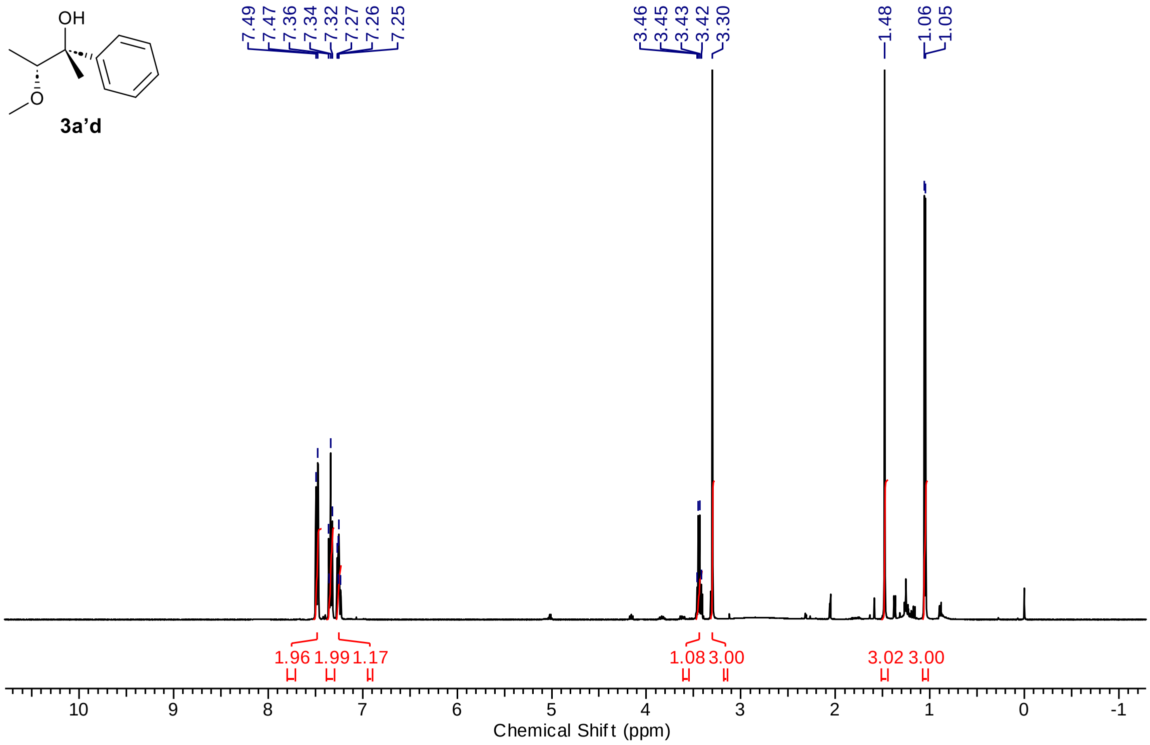

[13]**C-NMR** (100 MHz, $CDCl_3$):

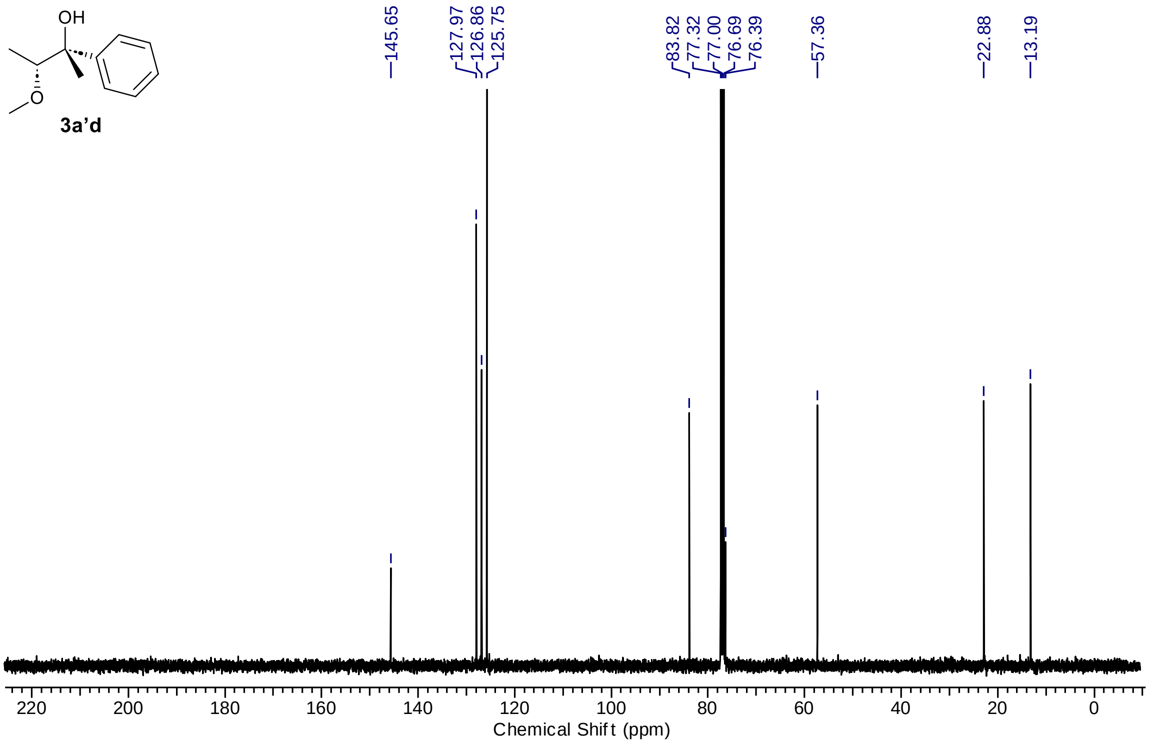

(2*R*,3*S*)-3-Methoxy-2-phenylbutan-2-ol (3a'd')

^{1}H-NMR (400 MHz, $CDCl_3$):

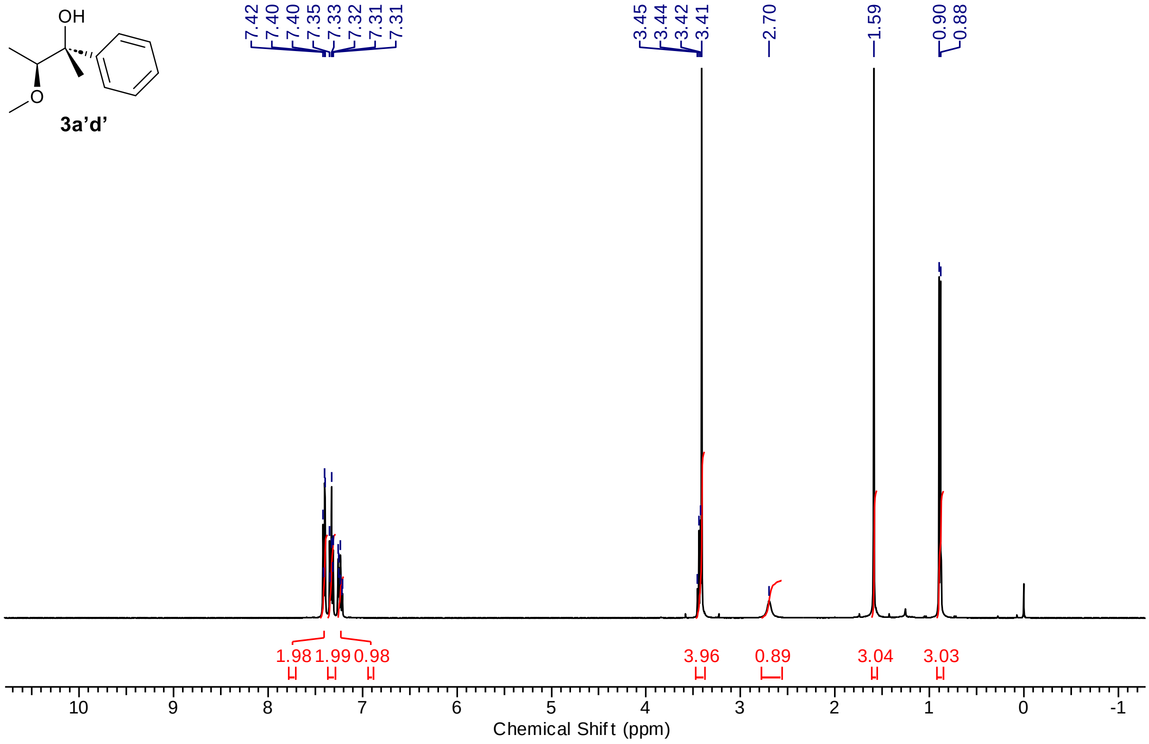

^{13}C-NMR (100 MHz, $CDCl_3$):

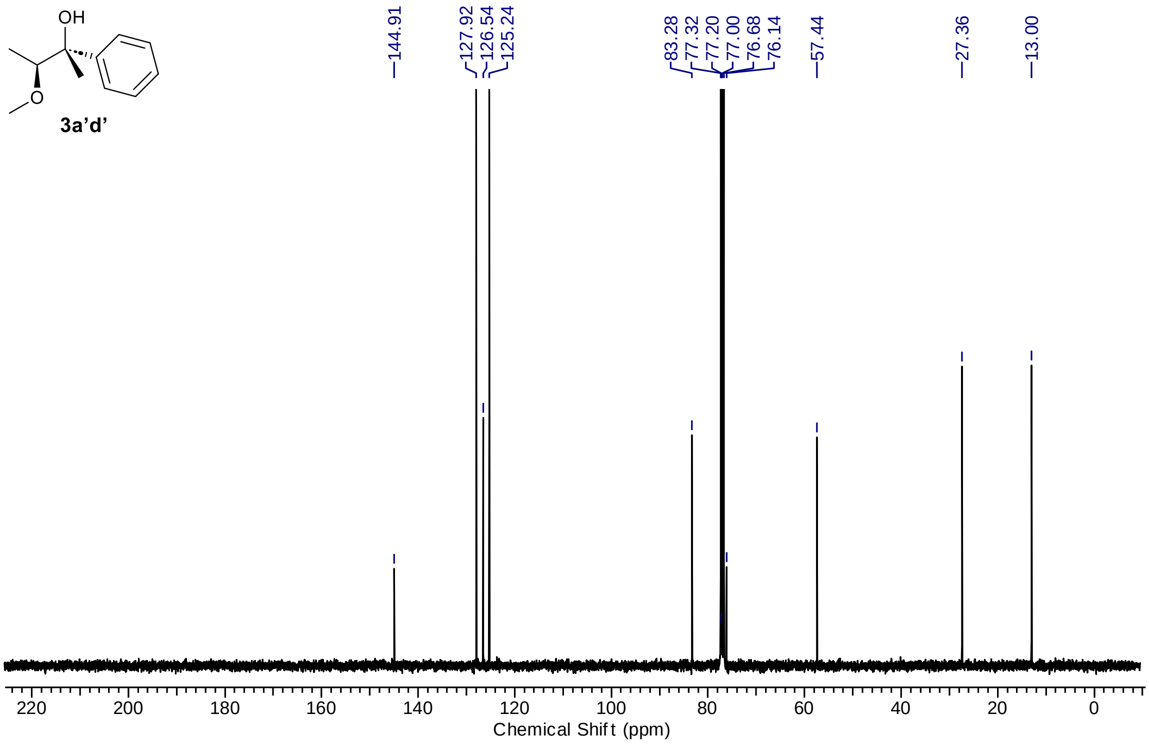

(2*S*,4*S*)-4-((4-Methoxybenzyl)oxy)-2-phenylpentan-2-ol (3a'e')

^{1}H-NMR (500 MHz, $CDCl_3$):

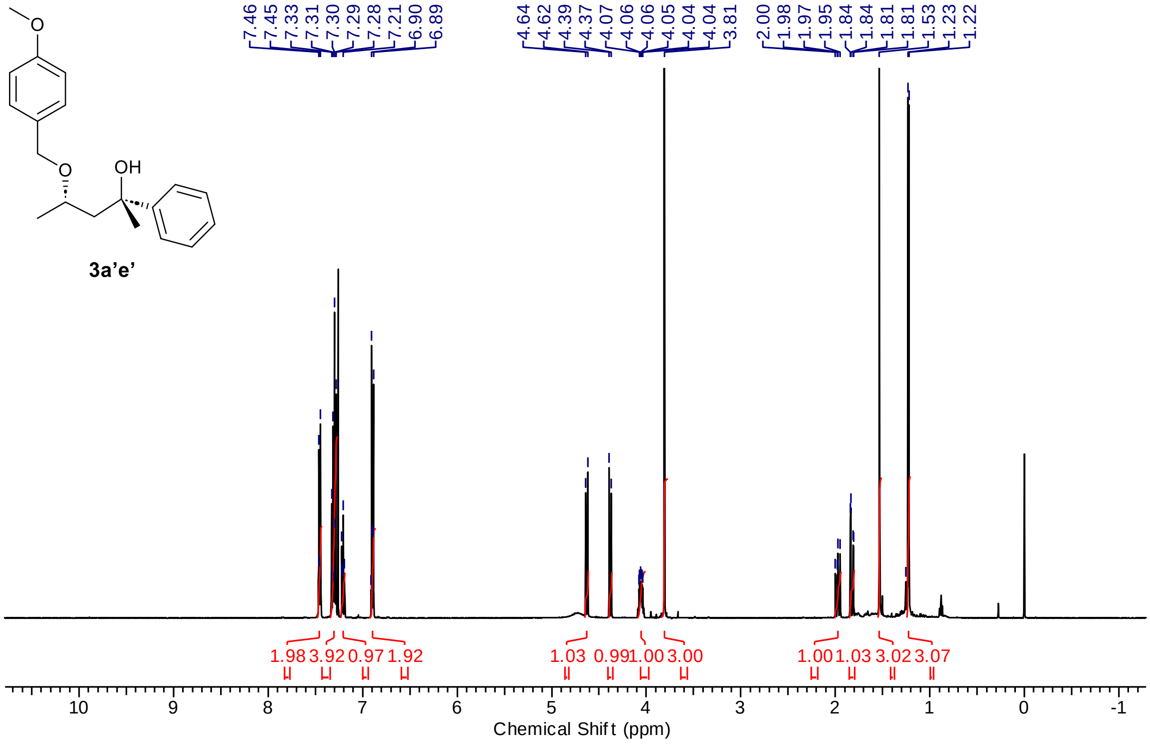

^{13}C-NMR (125 MHz, $CDCl_3$):

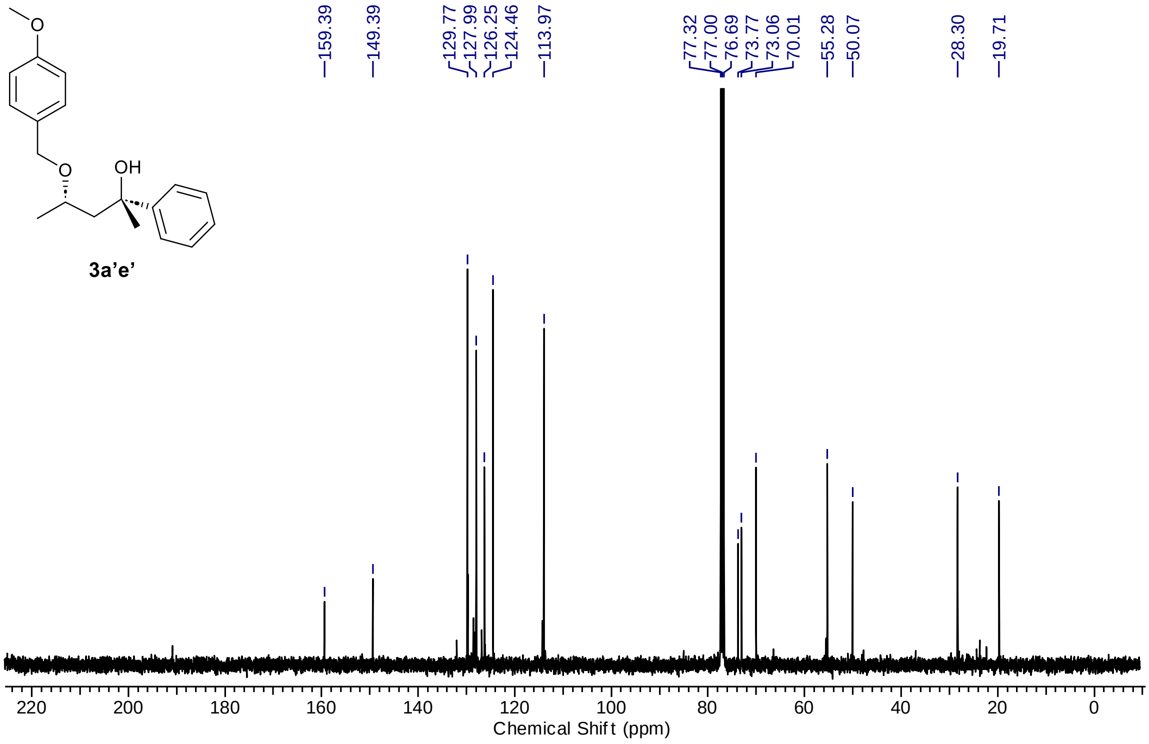

(2*S*,3*R*,4*R*)-4-((4-Methoxybenzyl)oxy)-3-methyl-2-phenylpentan-2-ol (3a’f)

^{1}H-NMR (400 MHz, $CDCl_3$):

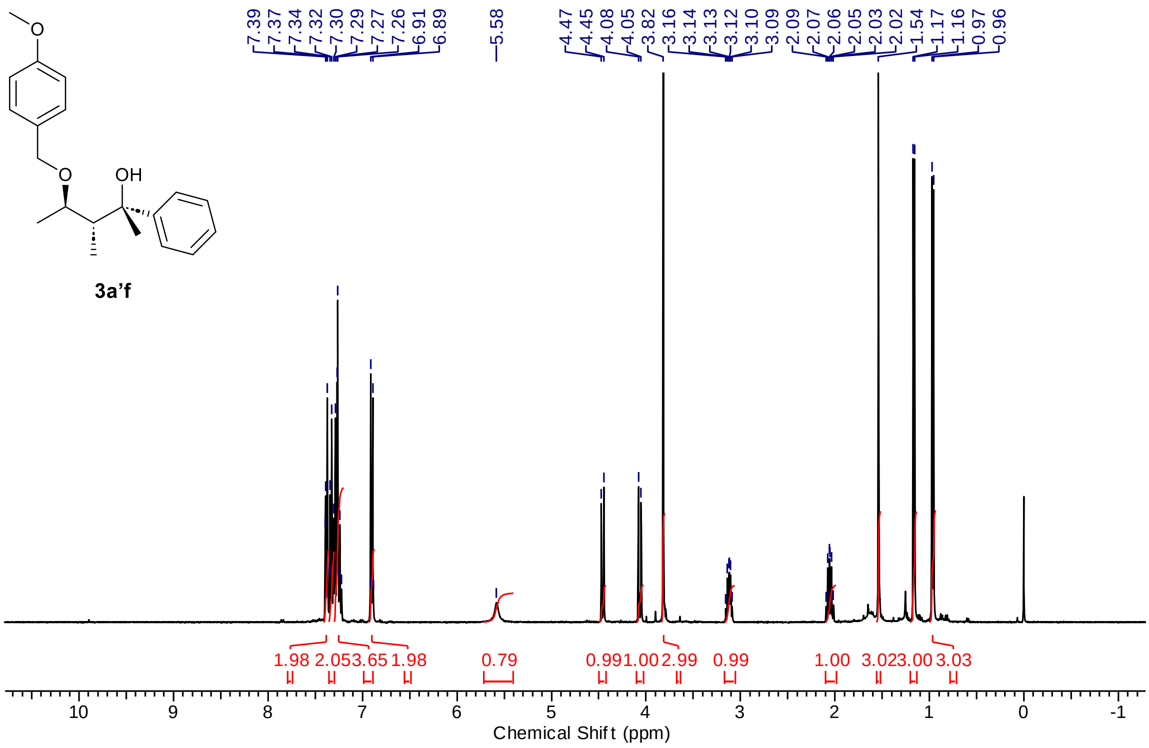

^{13}C-NMR (100 MHz, $CDCl_3$):

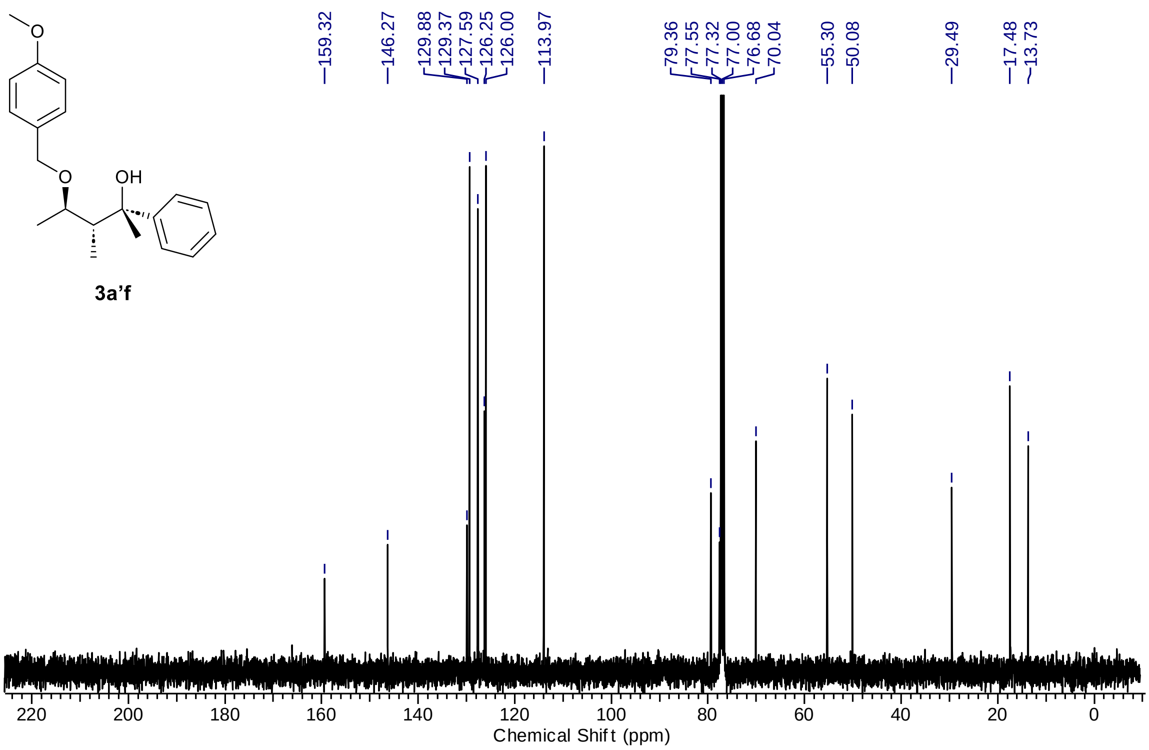

Diastereomers without use of $MgBr_2$: (2*S*,3*R*,4*R*)-4-((4-Methoxybenzyl)oxy)-3-methyl-2-phenylpentan-2-ol (3a'f) and (2*R*,3*R*,4*R*)-4-((4-Methoxybenzyl)oxy)-3-methyl-2-phenylpentan-2-ol

^{1}H-NMR (400 MHz, $CDCl_3$):

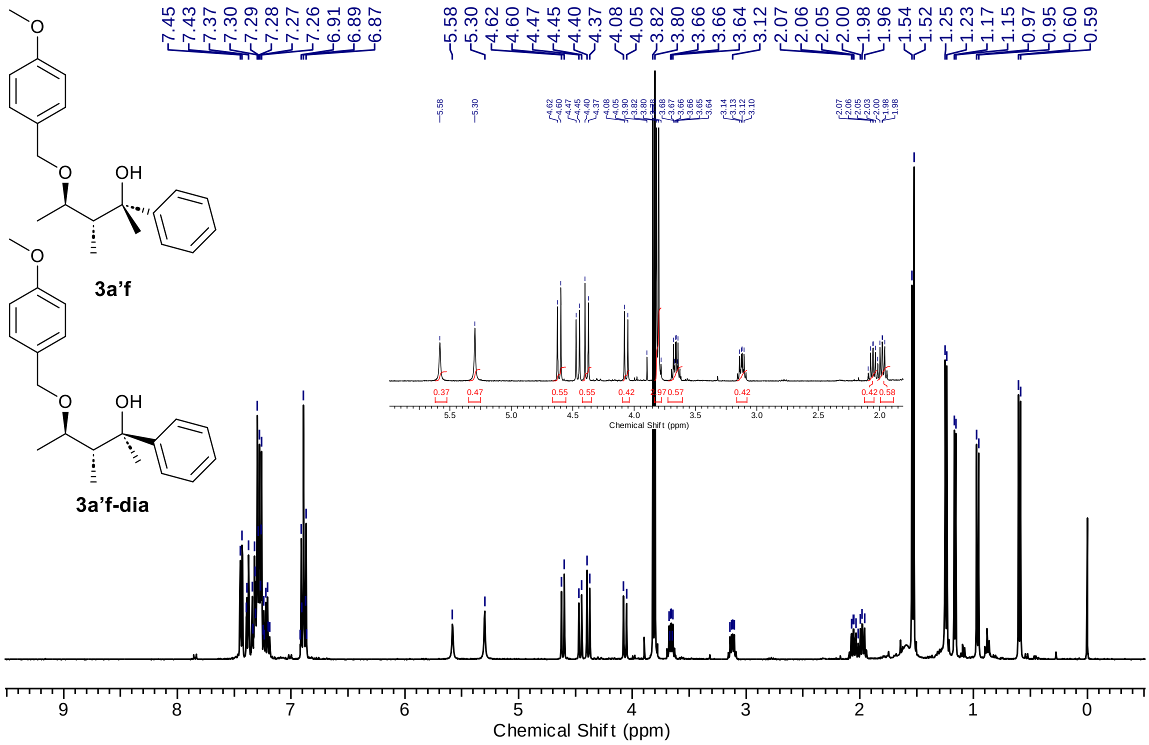

^{13}C-NMR (100 MHz, $CDCl_3$):

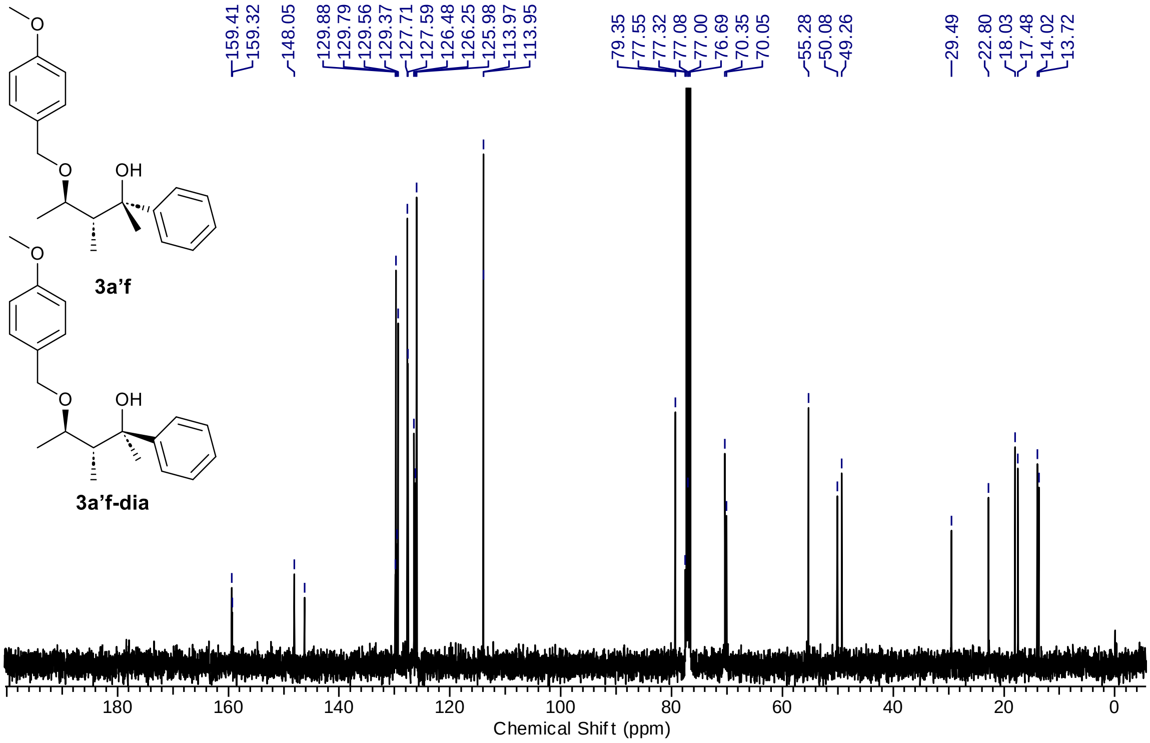

$^{1}H,^{1}H$-COSY

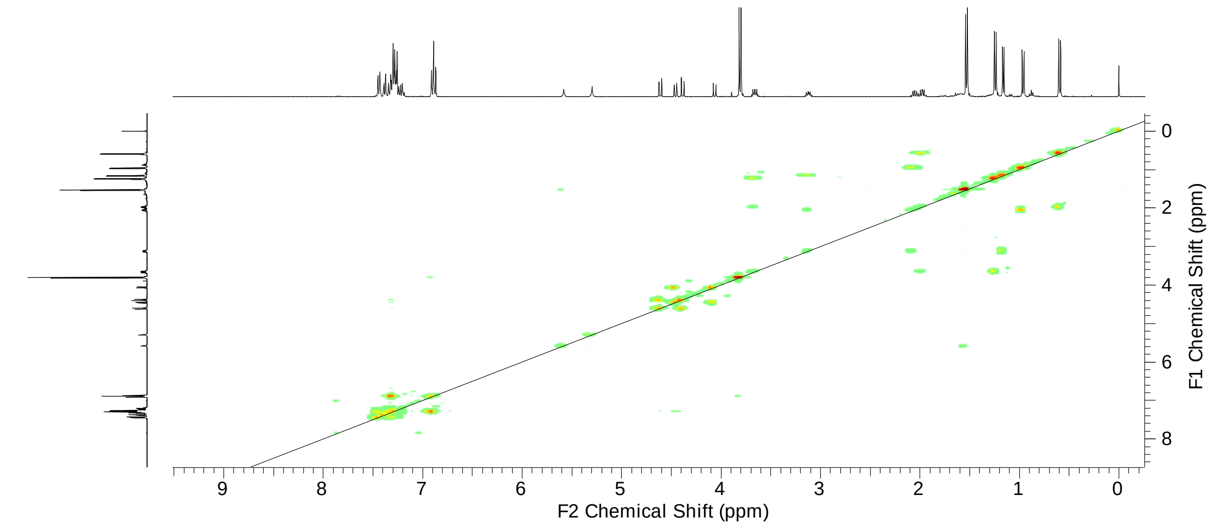

$^{1}H,^{13}C$-HSQC

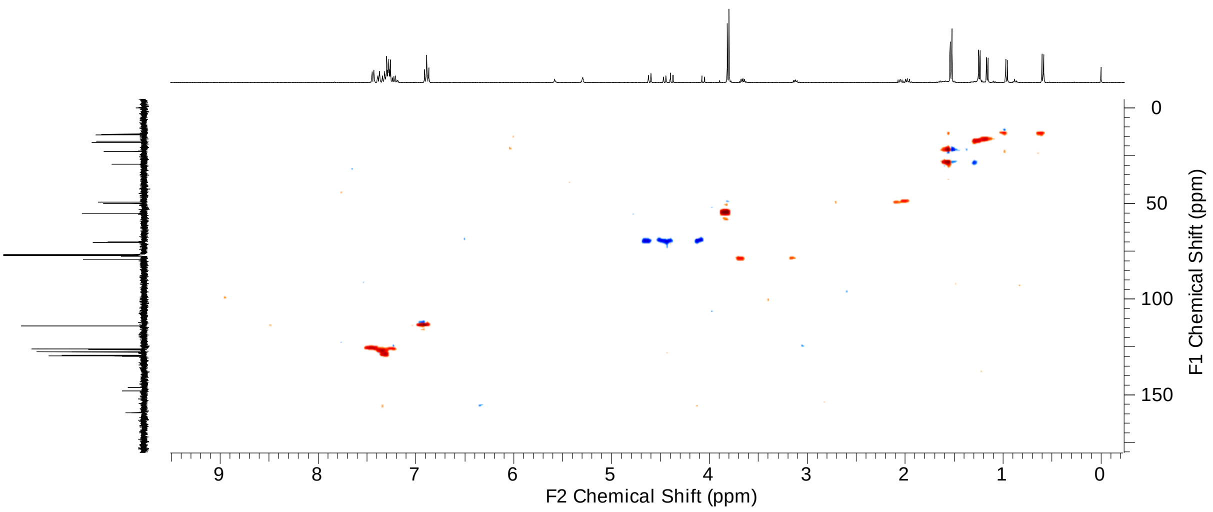

$^{1}H,^{13}C$-HMBC

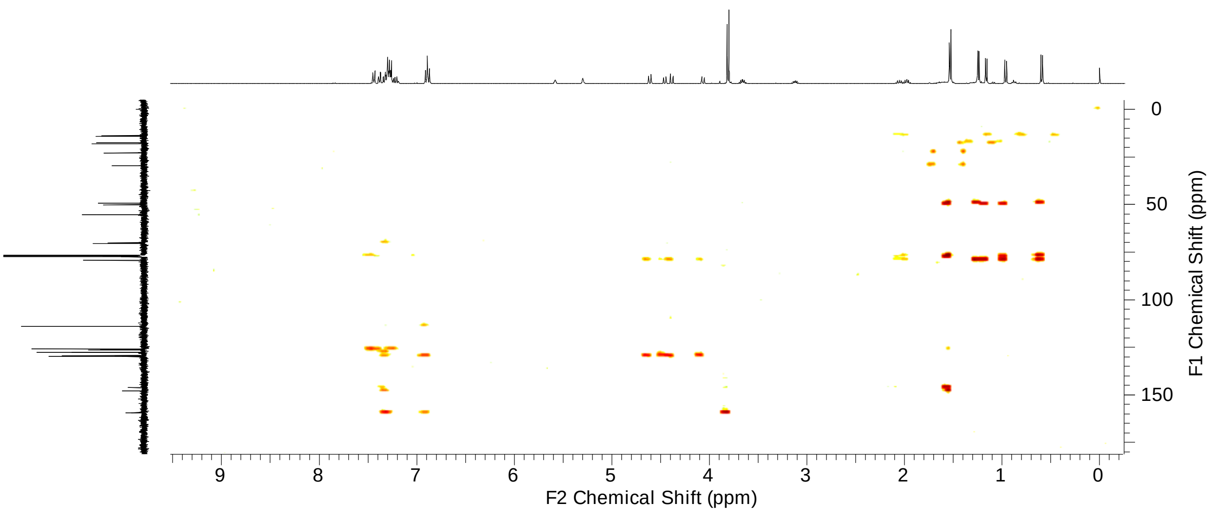

(2*S*,4*S*)-4-Methyl-2-phenyl-5-(trityloxy)pentan-2-ol (3a’h)

^{1}H-NMR (500 MHz, $CDCl_3$):

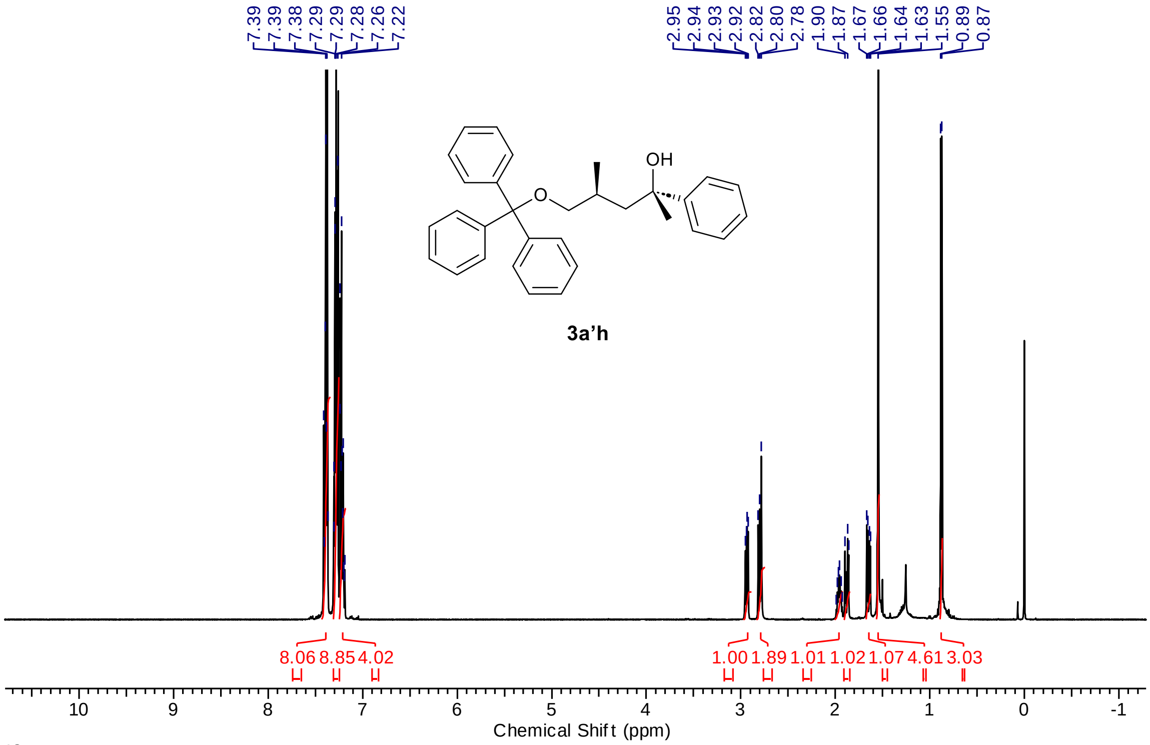

^{13}C-NMR (125 MHz, $CDCl_3$):

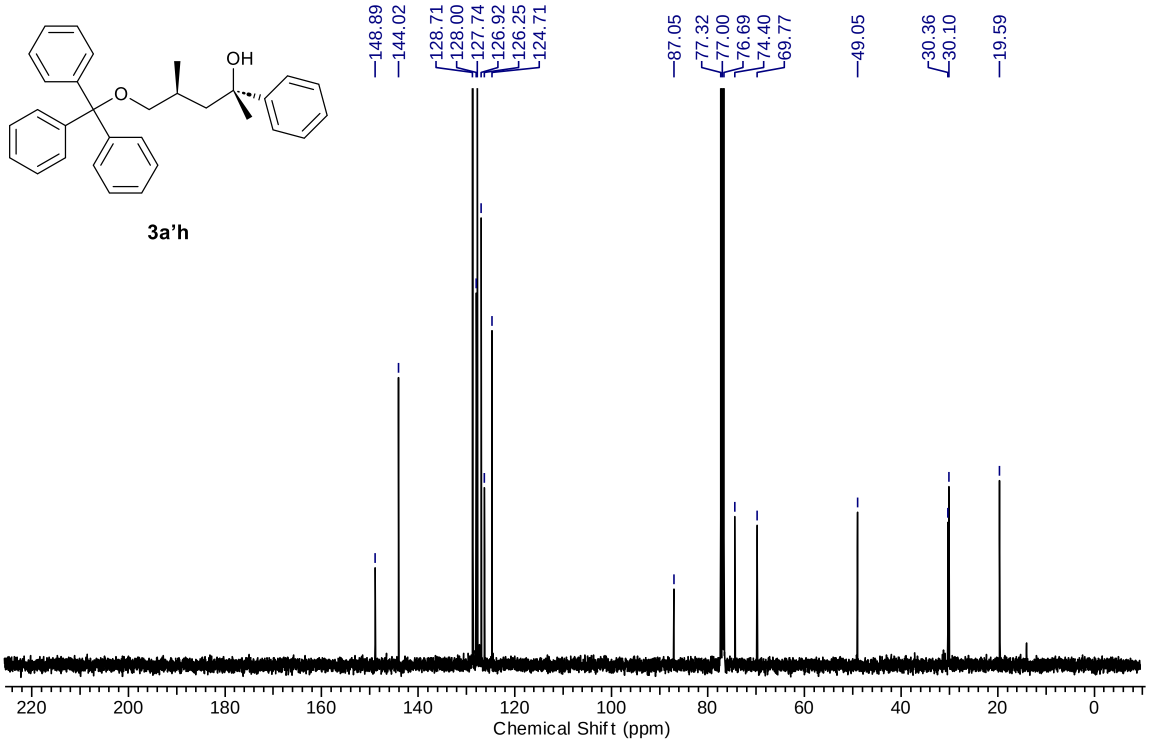

(2*R*,3*R*)-2-(4-Chlorophenyl)-3-((4-methoxybenzyl)oxy)butan-2-ol (3b'c)

^{1}H-NMR (400 MHz, $CDCl_3$):

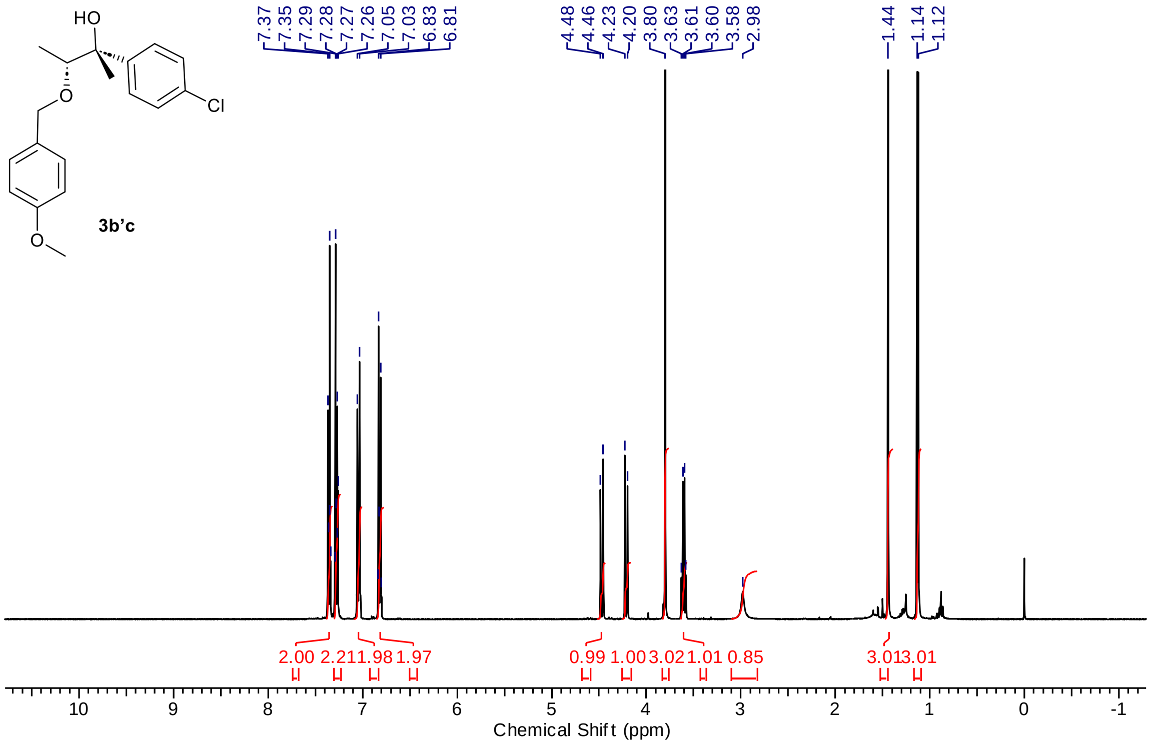

^{13}C-NMR (100 MHz, $CDCl_3$):

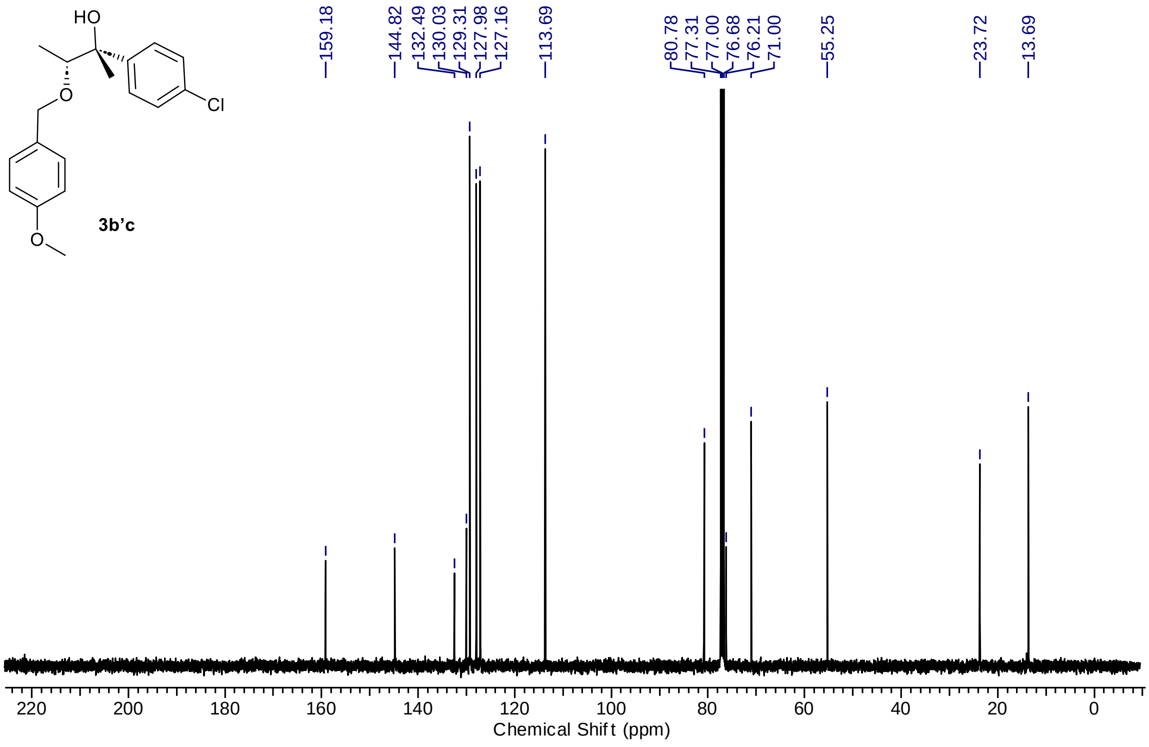

(2*R*,4*R*)-2-(4-Methoxyphenyl)-4-methylheptan-2-ol (3cb')

^{1}H-NMR (500 MHz, $CDCl_3$):

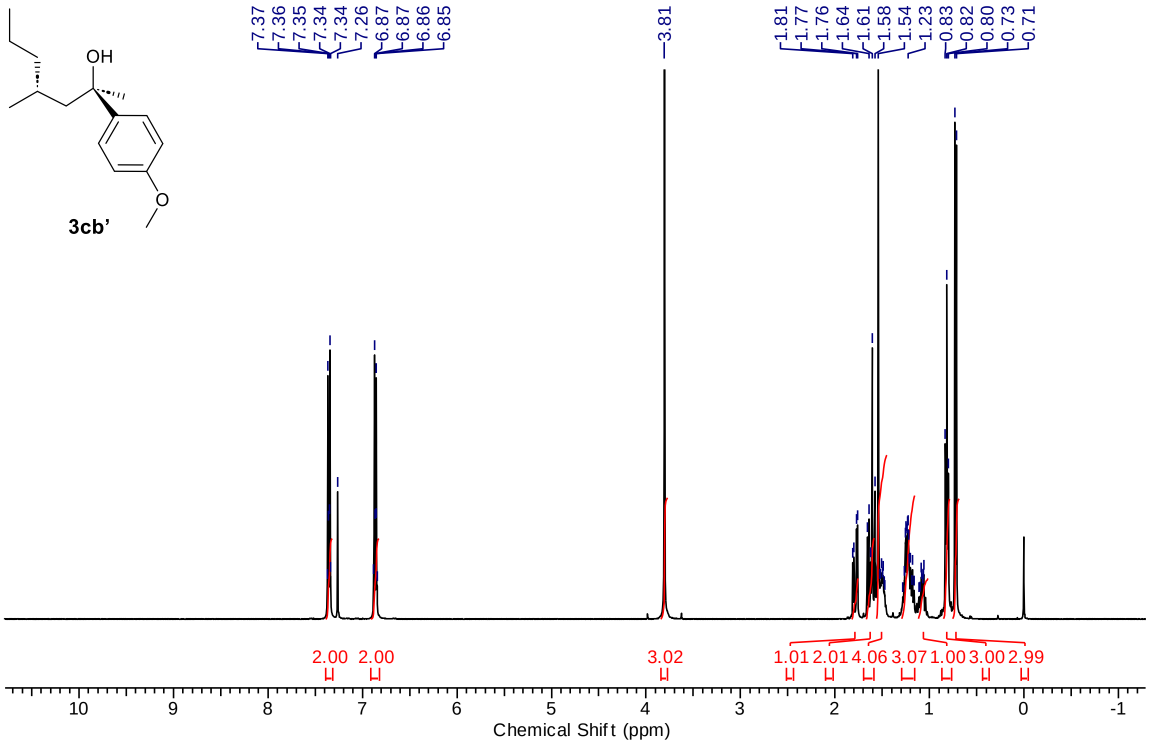

^{13}C-NMR (125 MHz, $CDCl_3$):

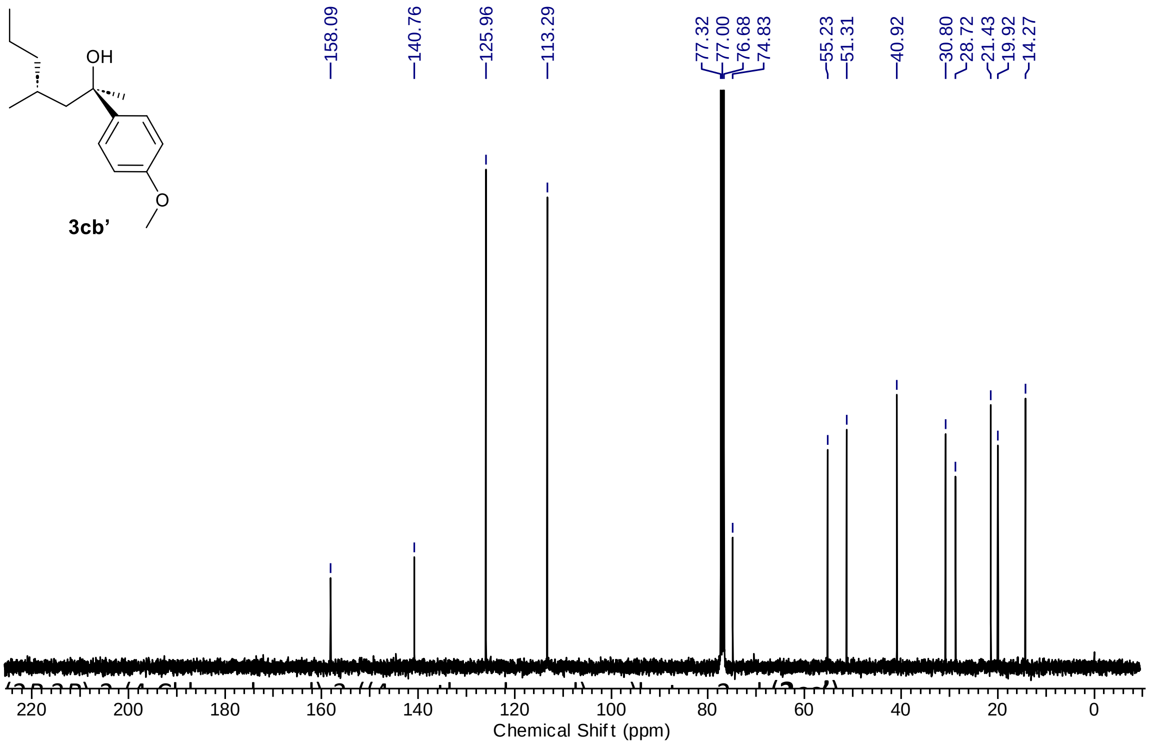

(2*R*,3*R*)-2-(4-Chlorophenyl)-3-((4-methoxybenzyl)oxy)butan-2-ol (3cc')

^{1}H-NMR (500 MHz, $CDCl_3$):

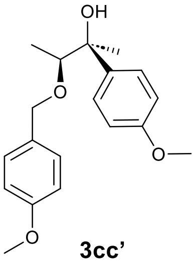

3cc'

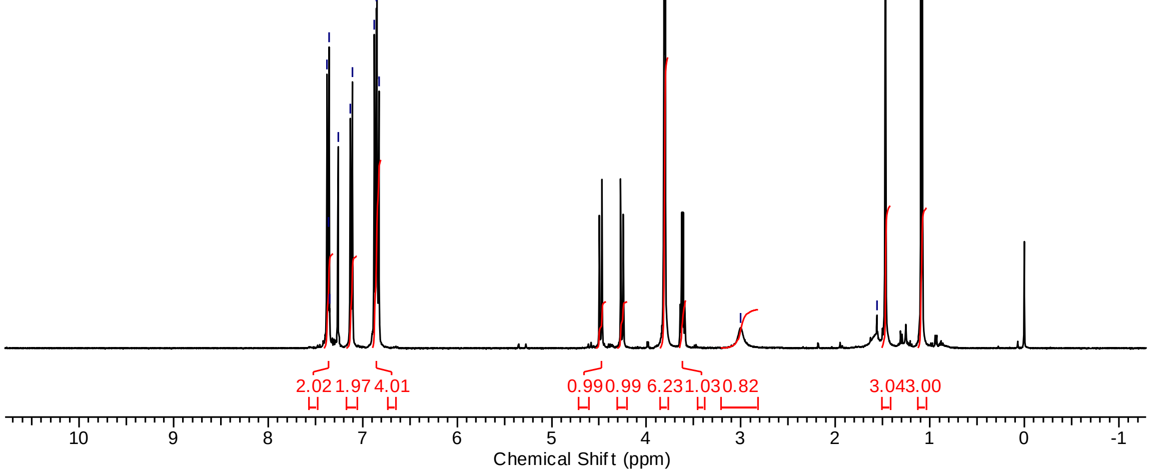

^{13}C-NMR (125 MHz, $CDCl_3$):

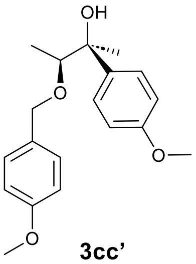

3cc'

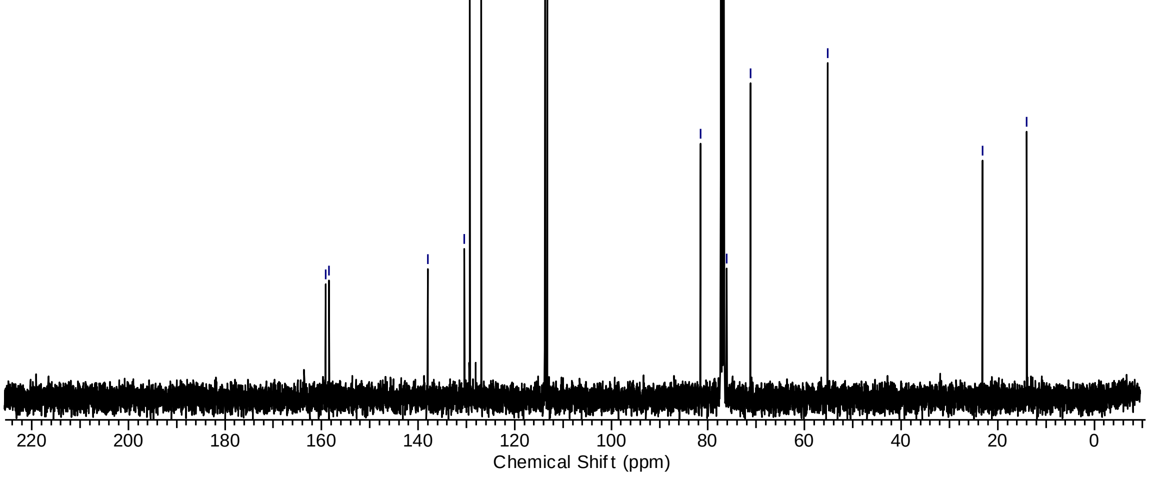

(2*R*,4*S*)-4-((4-Methoxybenzyl)oxy)-2-(3-methoxyphenyl)pentan-2-ol (3de')

^{1}H-NMR (400 MHz, $CDCl_3$):

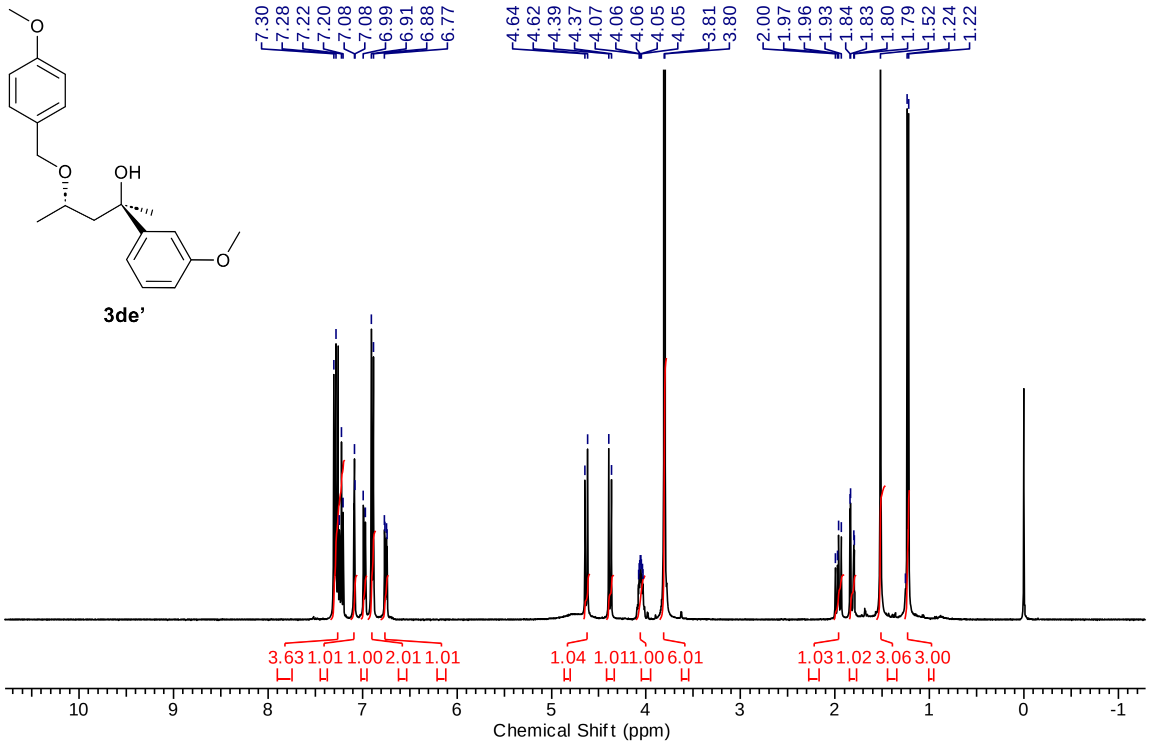

^{13}C-NMR (100 MHz, $CDCl_3$):

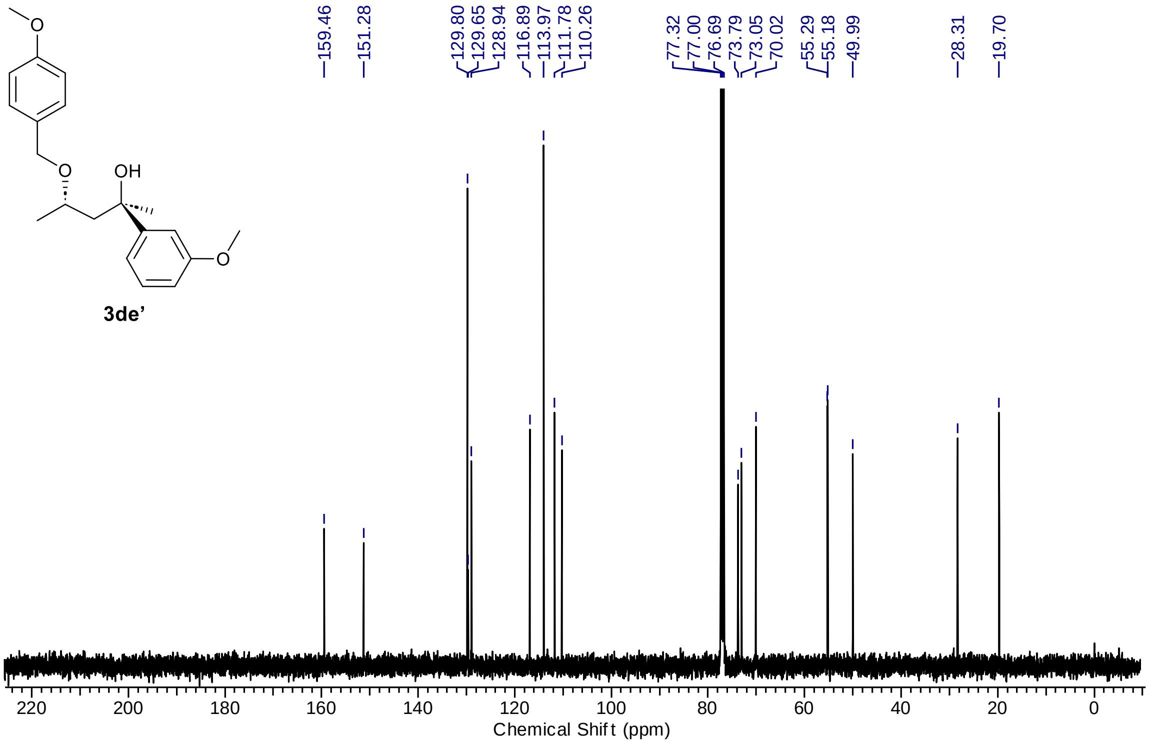

(2*R*,3*S*,4*S*)-4-((4-Methoxybenzyl)oxy)-2-(2-methoxyphenyl)-3-methylpentan-2-ol (3ef')

^{1}H-NMR (400 MHz, $CDCl_3$):

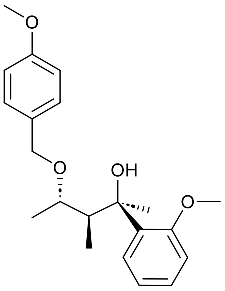

3ef'

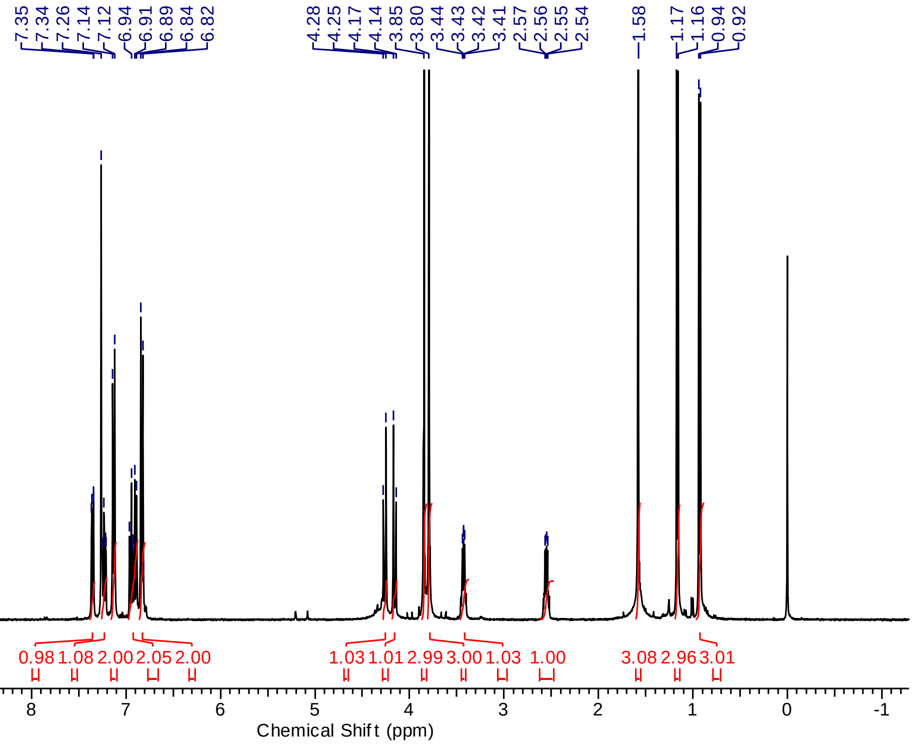

^{13}C-NMR (100 MHz, $CDCl_3$):

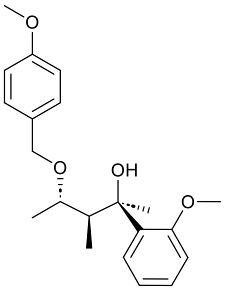

3ef'

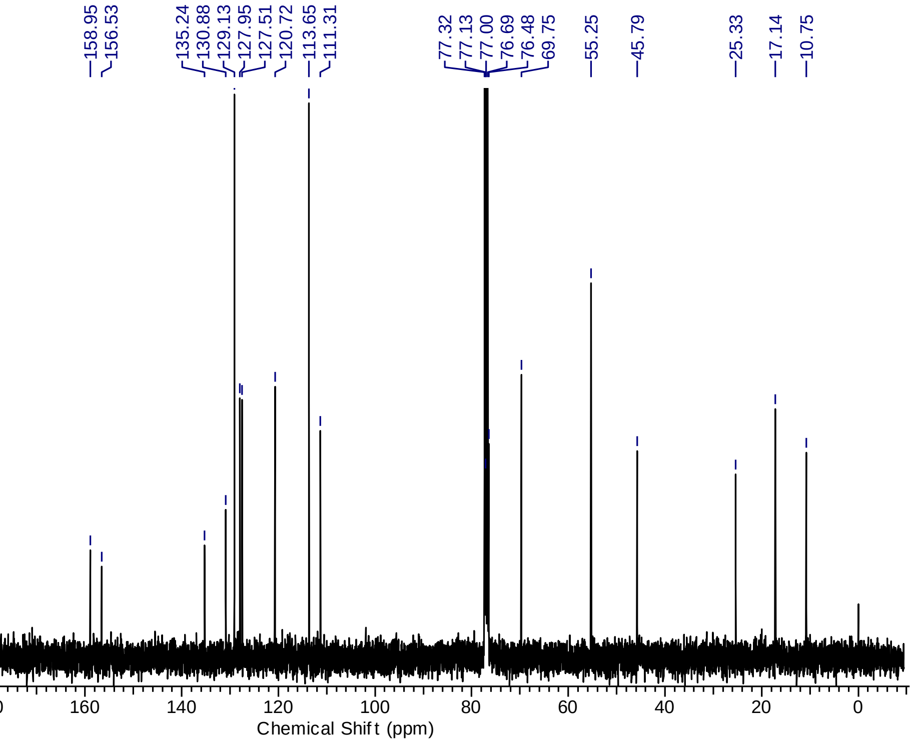

(2*R*,3*R*)-3-(2-((4-Methoxybenzyl)oxy)ethyl)-2-(3-methoxyphenyl)hex-5-en-2-ol (3dg)

^{1}H-NMR (400 MHz, $CDCl_3$):

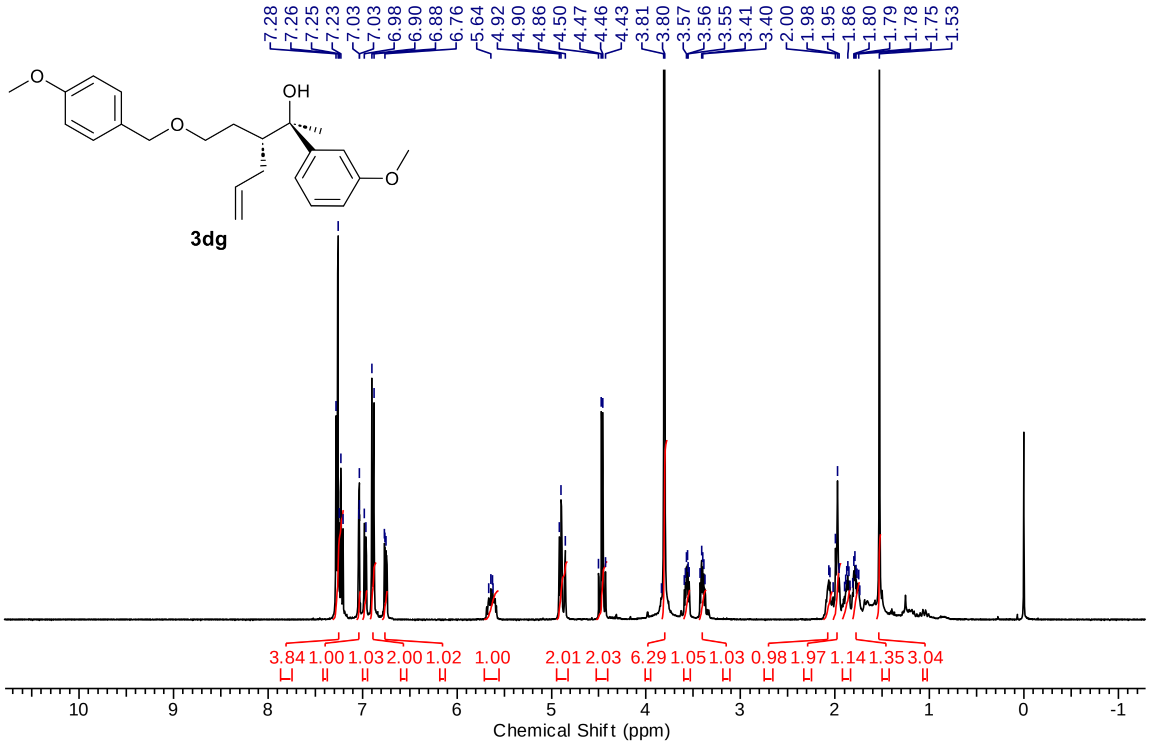

^{13}C-NMR (100 MHz, $CDCl_3$):

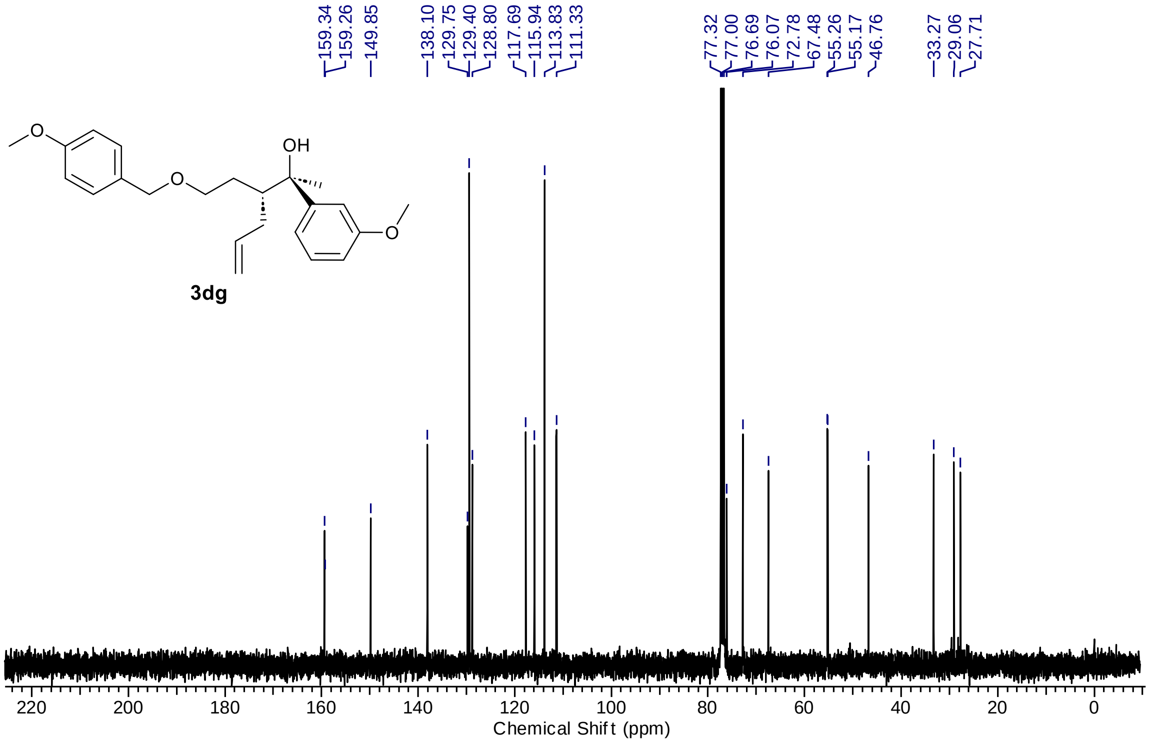

(4*R*,5*S*,*E*)-4,5-Dimethyloct-2-en-4-ol (3fa)

^{1}H-NMR (400 MHz, $CDCl_3$):

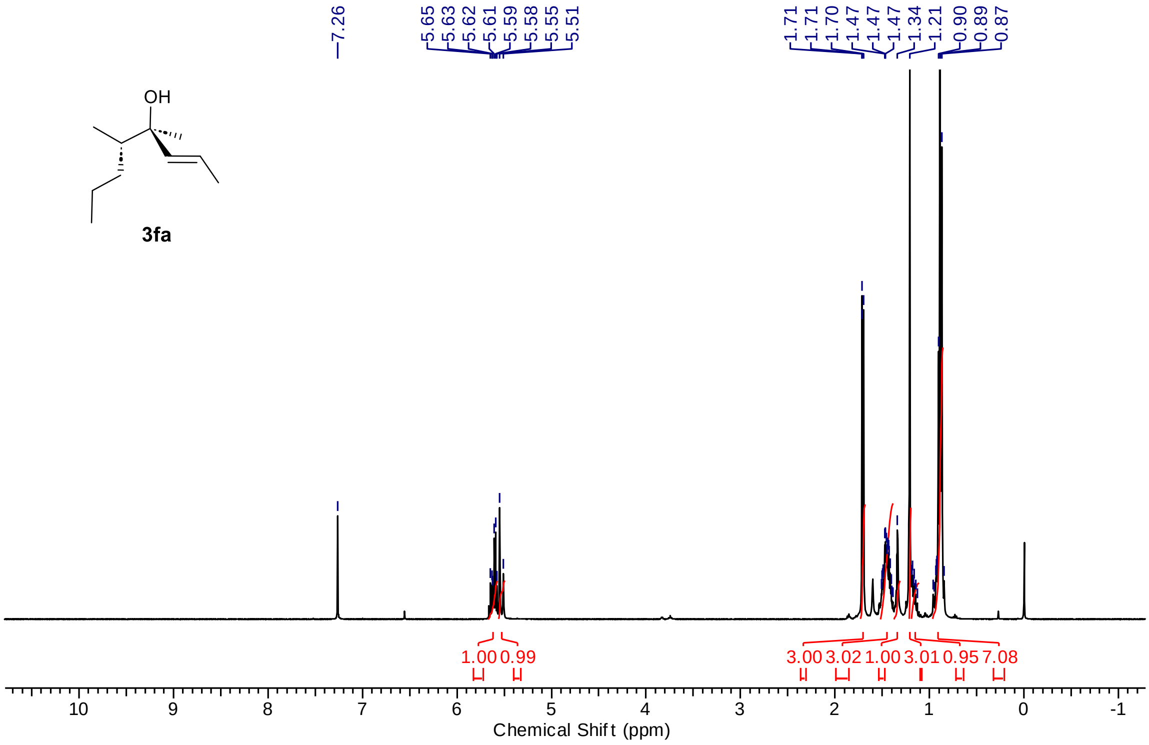

^{13}C-NMR (100 MHz, $CDCl_3$):

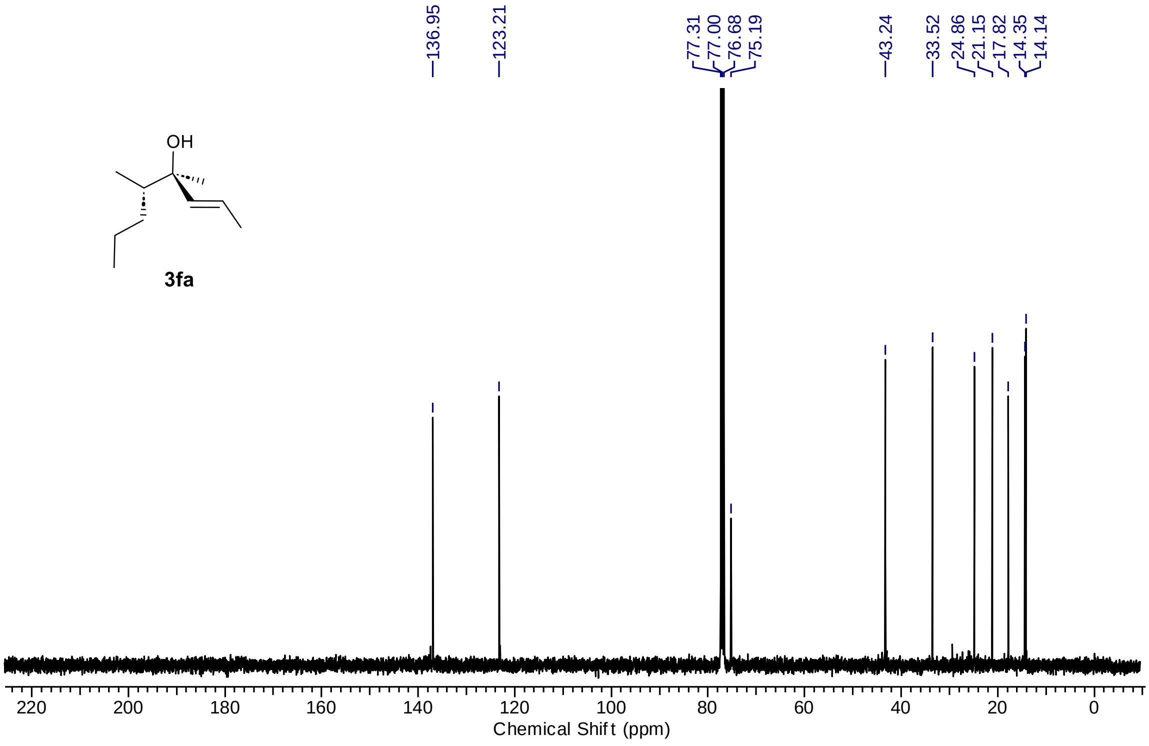

(4*R*,6*S*,*E*)-4-Ethyl-6-((4-methoxybenzyl)oxy)hept-2-en-4-ol (3ge')

^{1}H-NMR (400 MHz, $CDCl_3$):

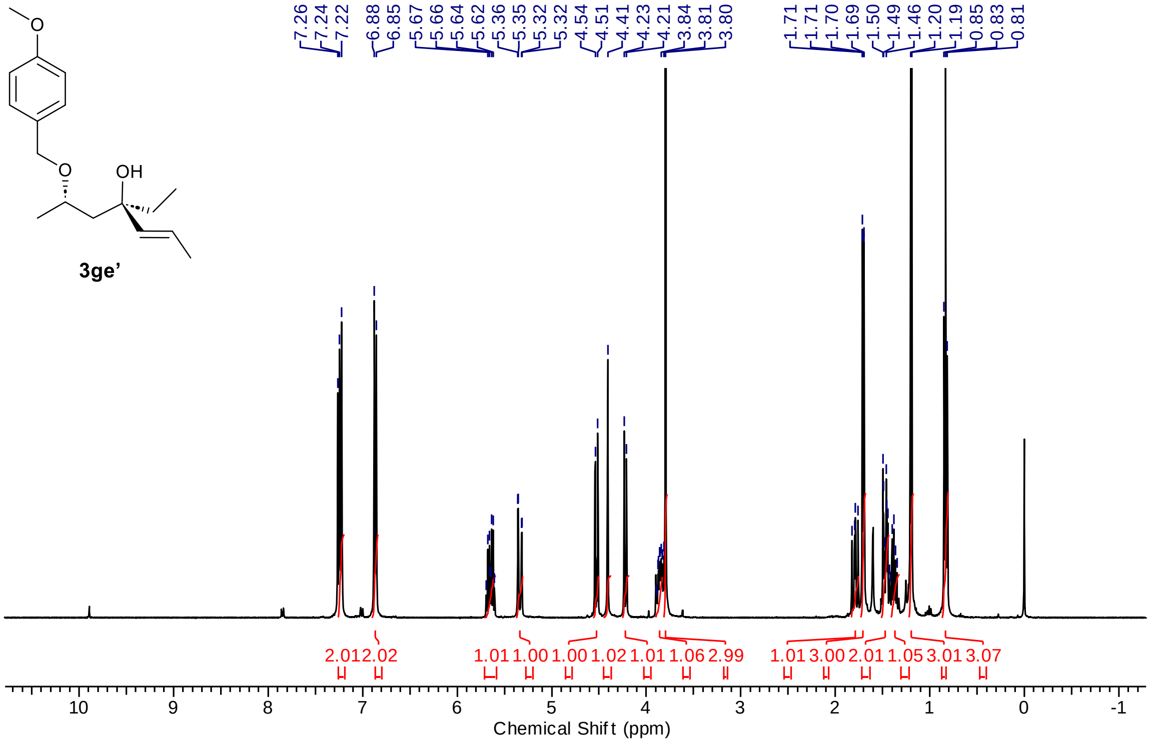

^{13}C-NMR (100 MHz, $CDCl_3$):

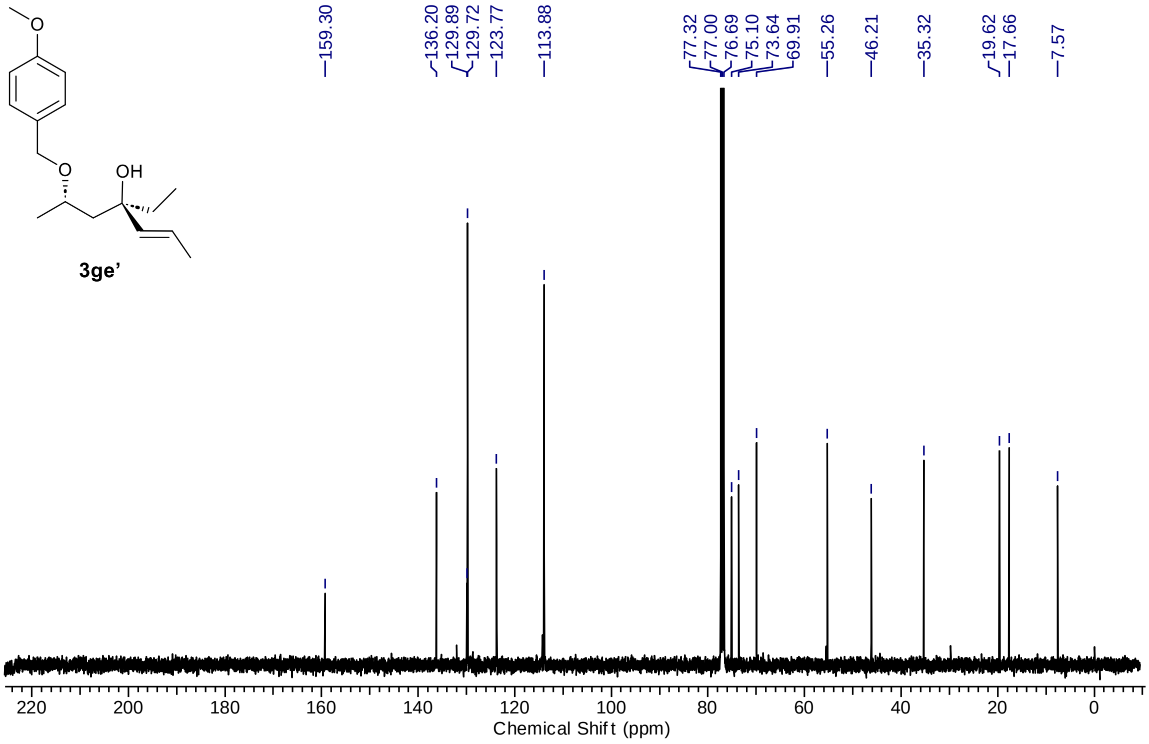

(4*S*,5*R*,7*S*)-4,7-Dimethyl-5-phenyldecan-5-ol (3ha):

^{1}**H-NMR** (400 MHz, $CDCl_3$):

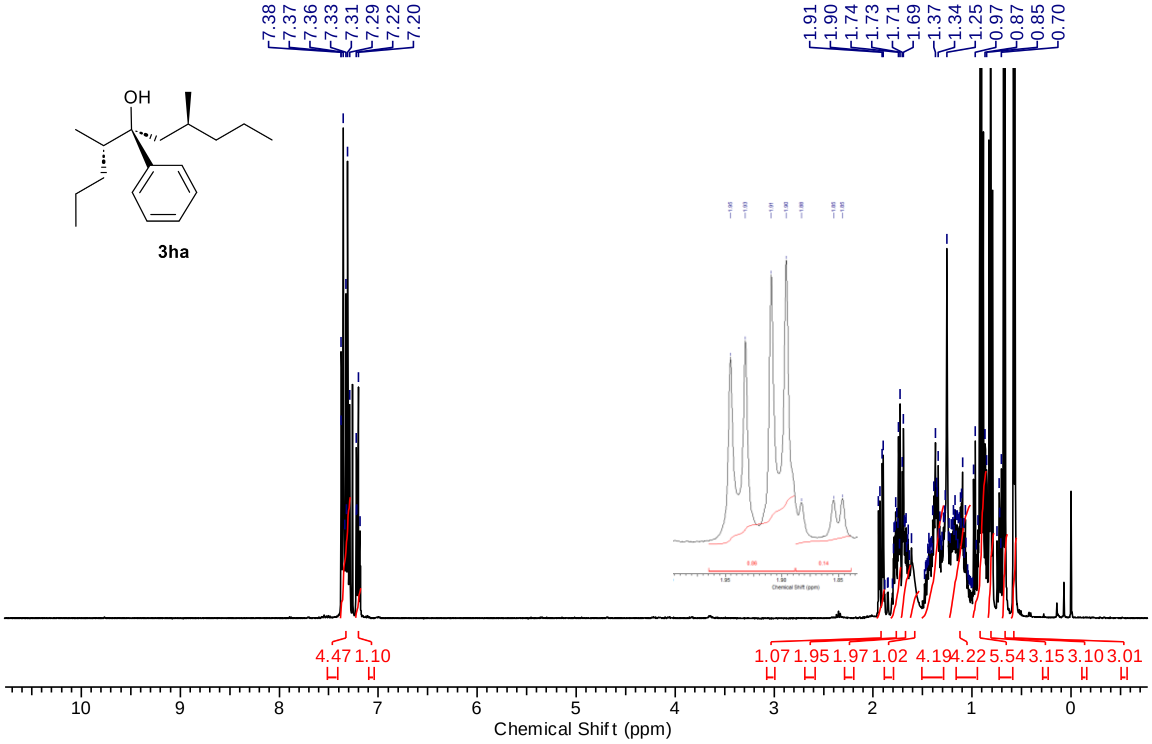

^{13}**C-NMR** (100 MHz, $CDCl_3$):

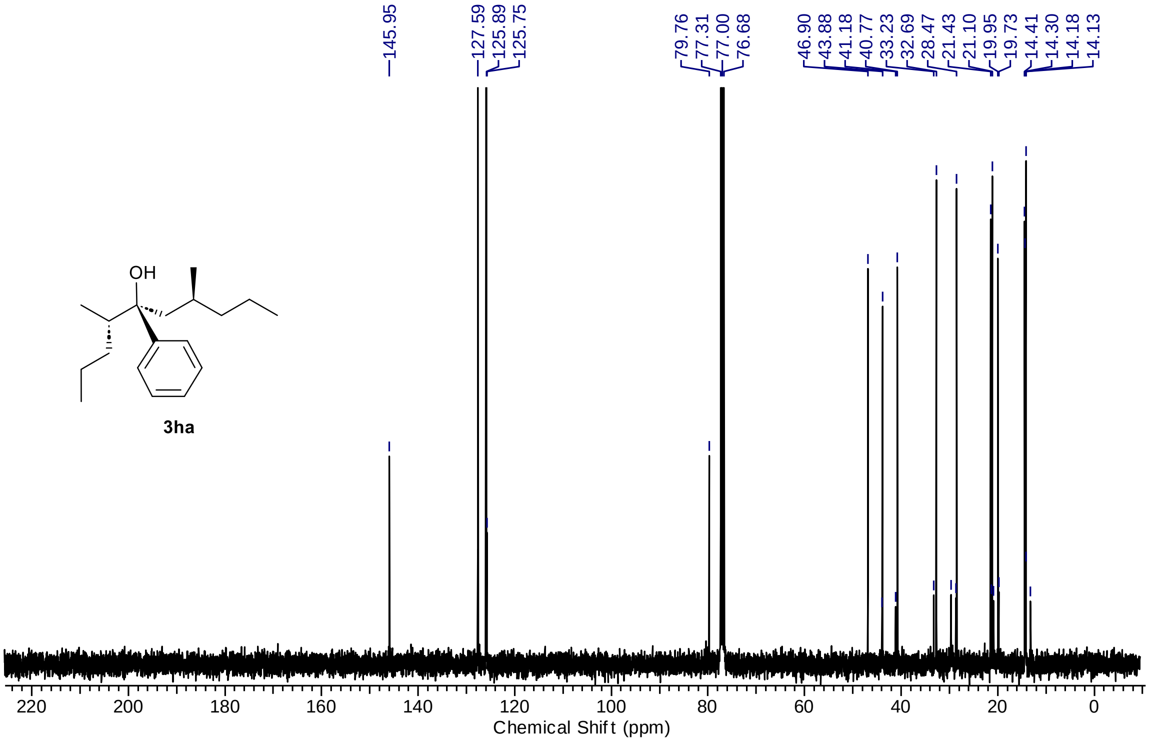

^{1}H,^{1}H-COSY

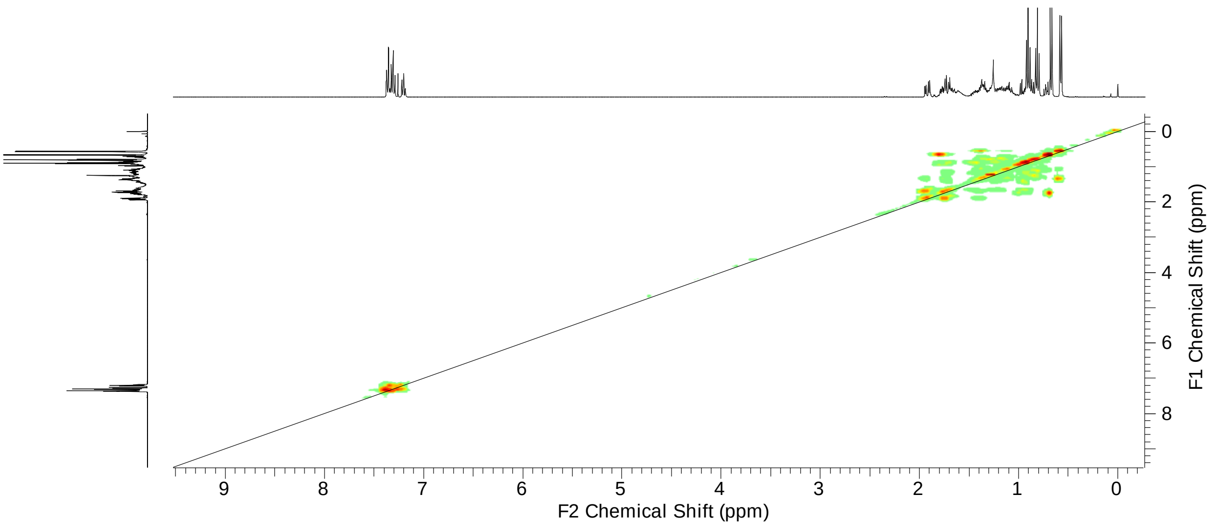

^{1}H,^{13}C-HSQC

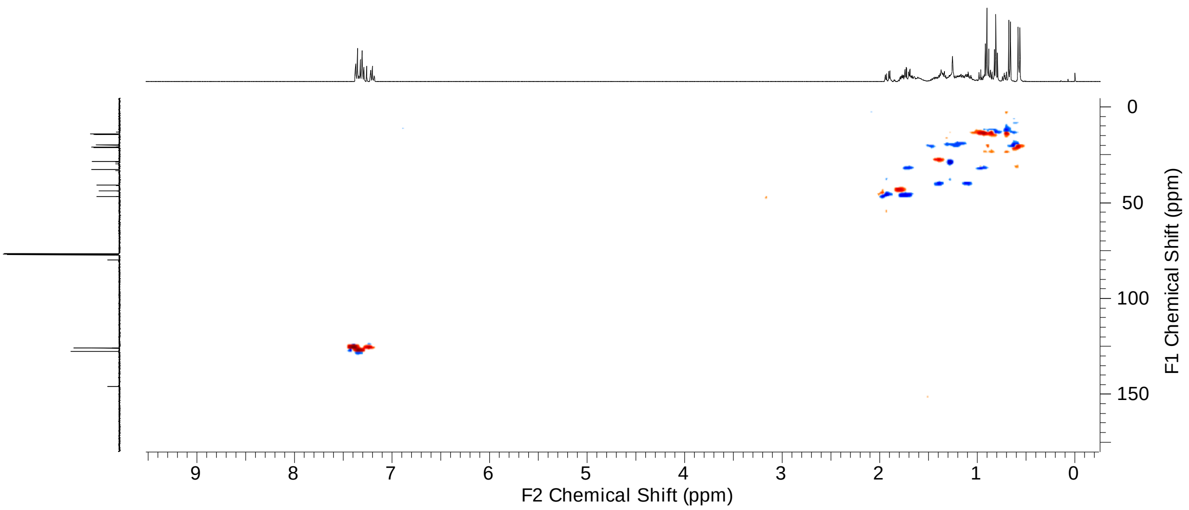

^{1}H,^{13}C-HMBC

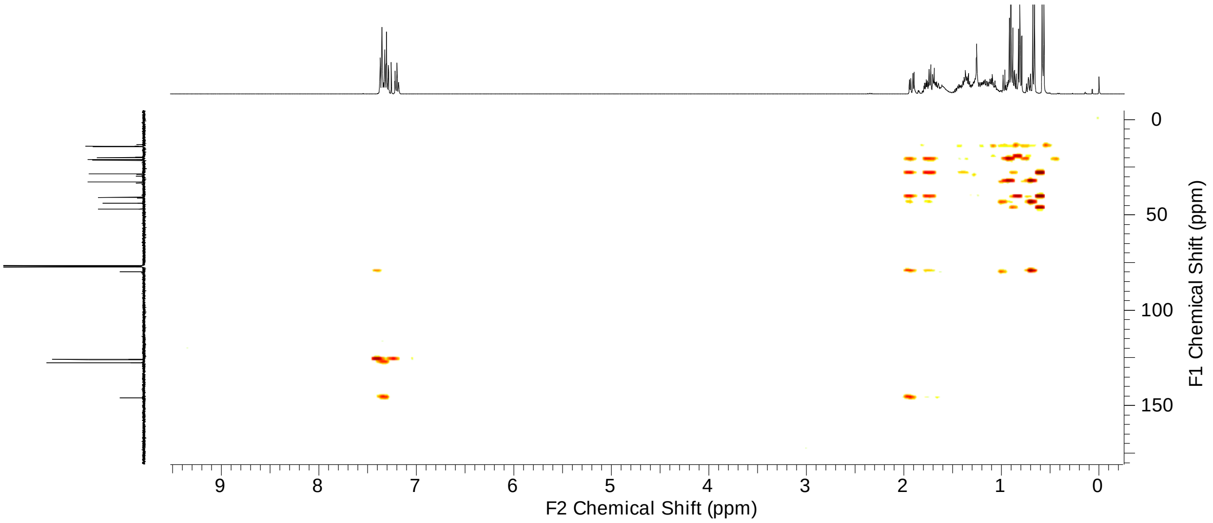

(2*S*,3S,5*S*)-2-((4-Methoxybenzyl)oxy)-5-methyl-3-phenyloctan-3-ol (3hc')

^{1}H-NMR (400 MHz, $CDCl_3$):

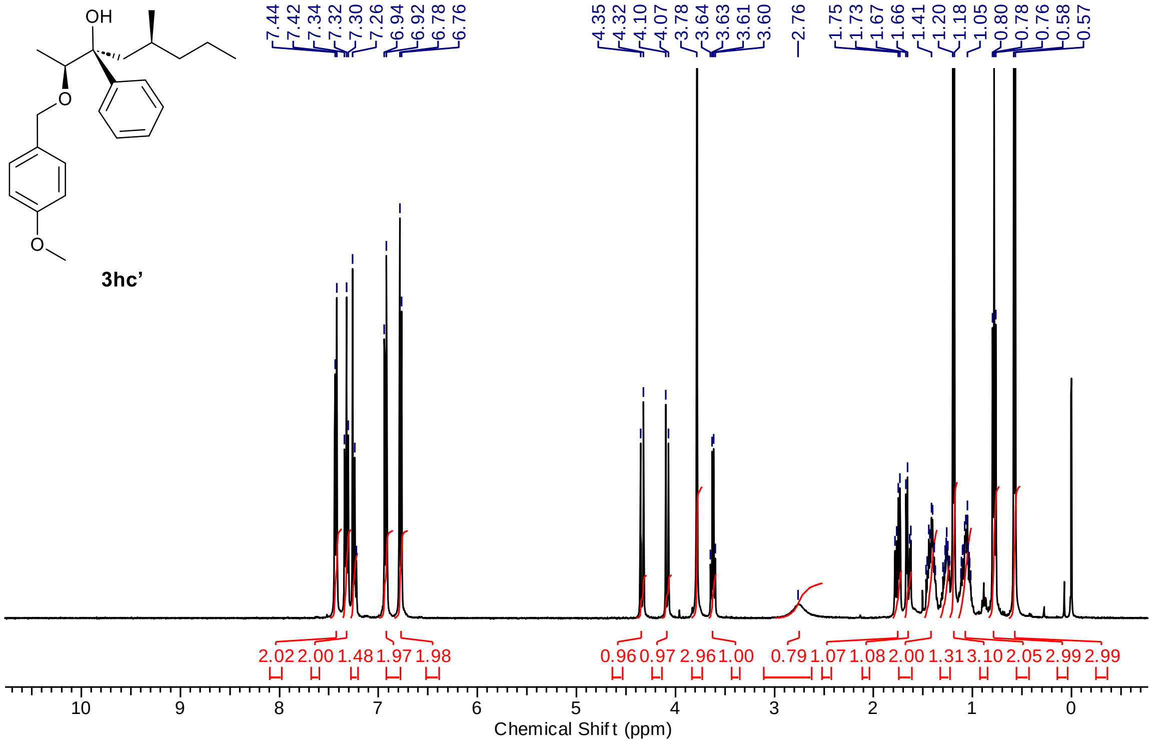

^{13}C-NMR (100 MHz, $CDCl_3$):

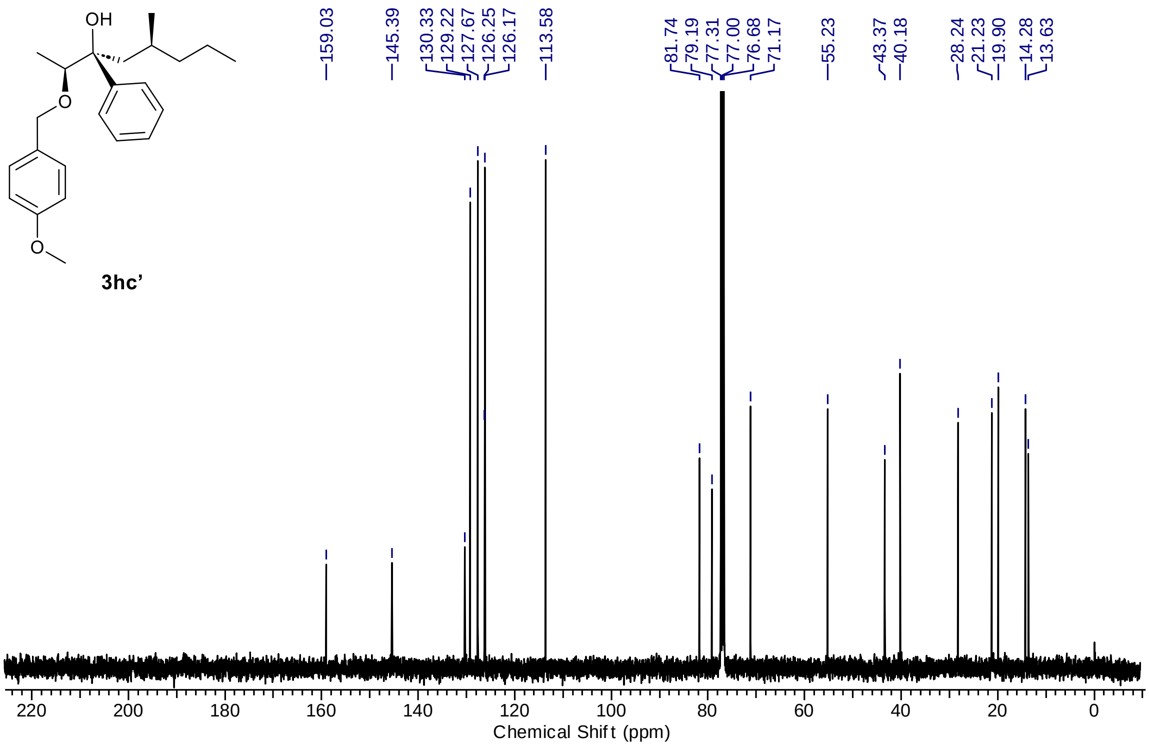

$^{1}H,^{1}H$-COSY

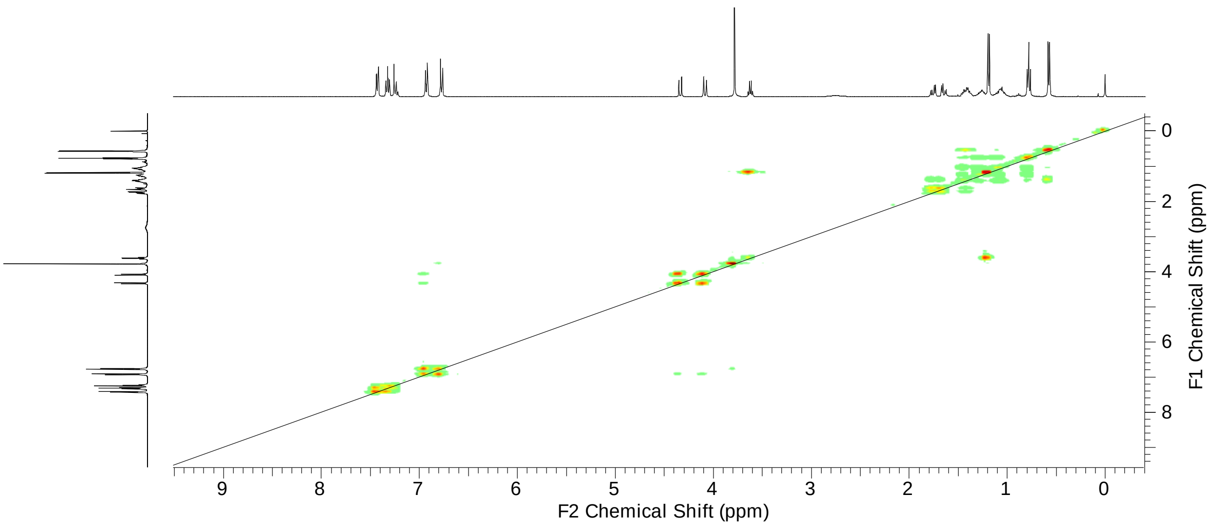

$^{1}H,^{13}C$-HSQC

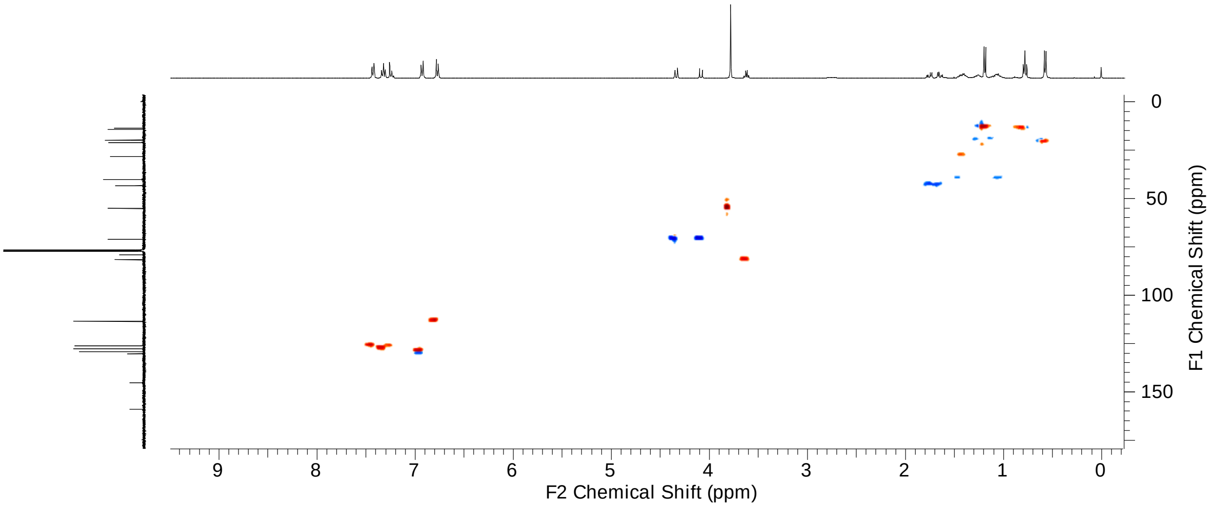

$^{1}H,^{13}C$-HMBC

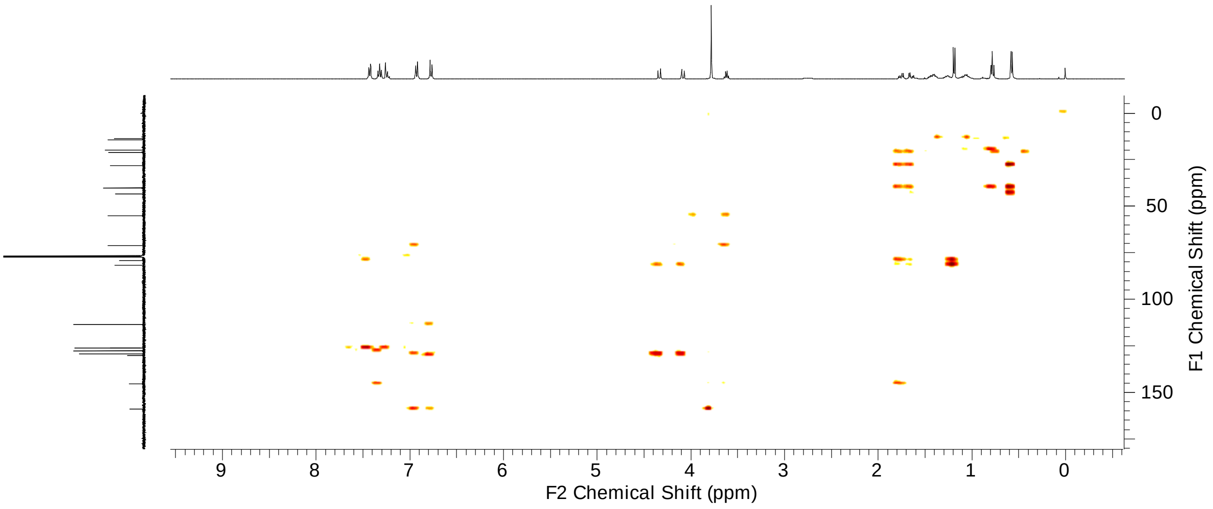

(2*S*,4*R*,6*S*)-2-((4-Methoxybenzyl)oxy)-6-methyl-4-phenylnonan-4-ol (3he')

^{1}H-NMR (400 MHz, $CDCl_3$):

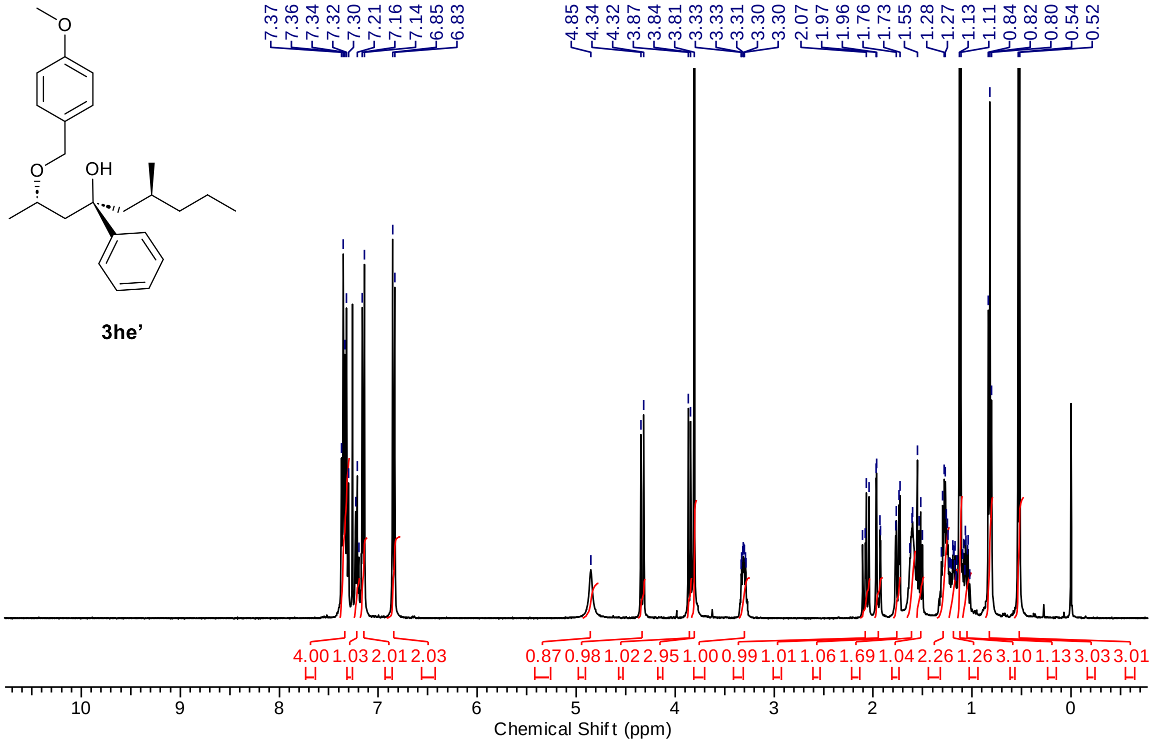

^{13}C-NMR (100 MHz, $CDCl_3$)

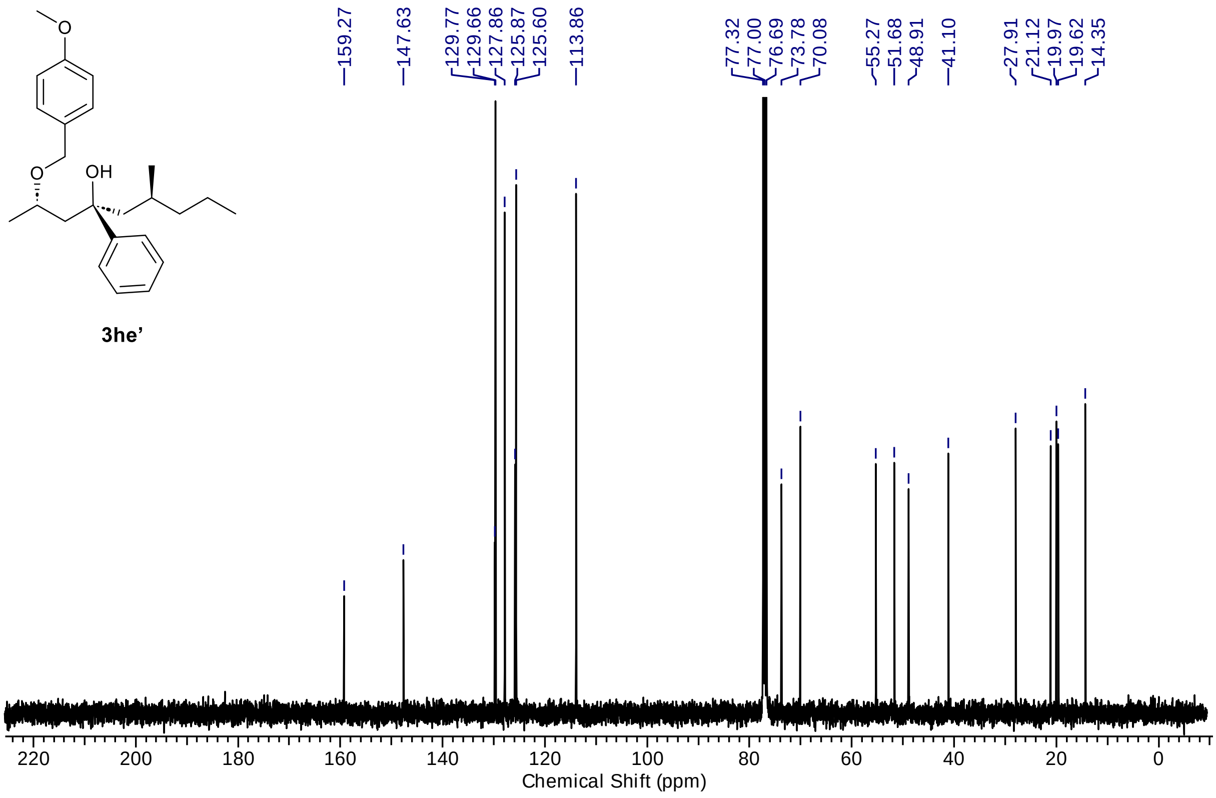

(4*R*,5*R*)-4,5-Dicyclohexyl-2-((2*S*,3*S*)-3-((4-methoxybenzyl)oxy)-2-phenylbutan-2-yl)-1,3,2-dioxaborolane (4a’c’)

^{1}H-NMR (400 MHz, $CDCl_3$):

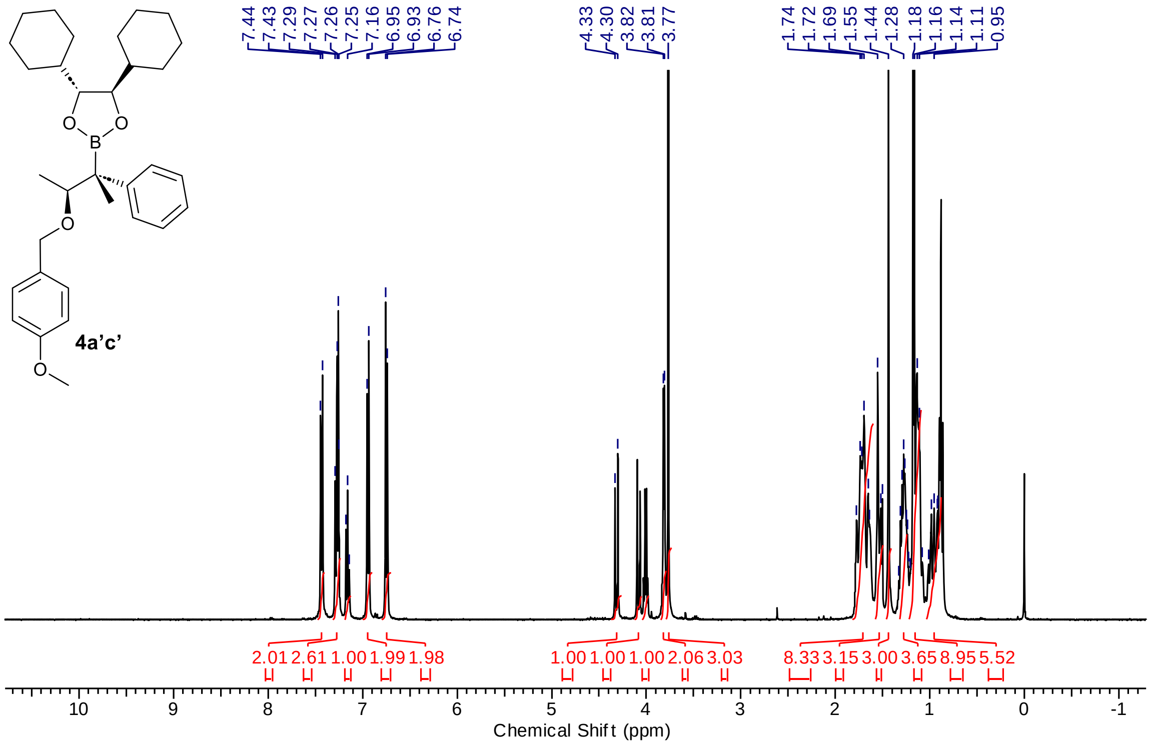

^{13}C-NMR (100 MHz, $CDCl_3$):

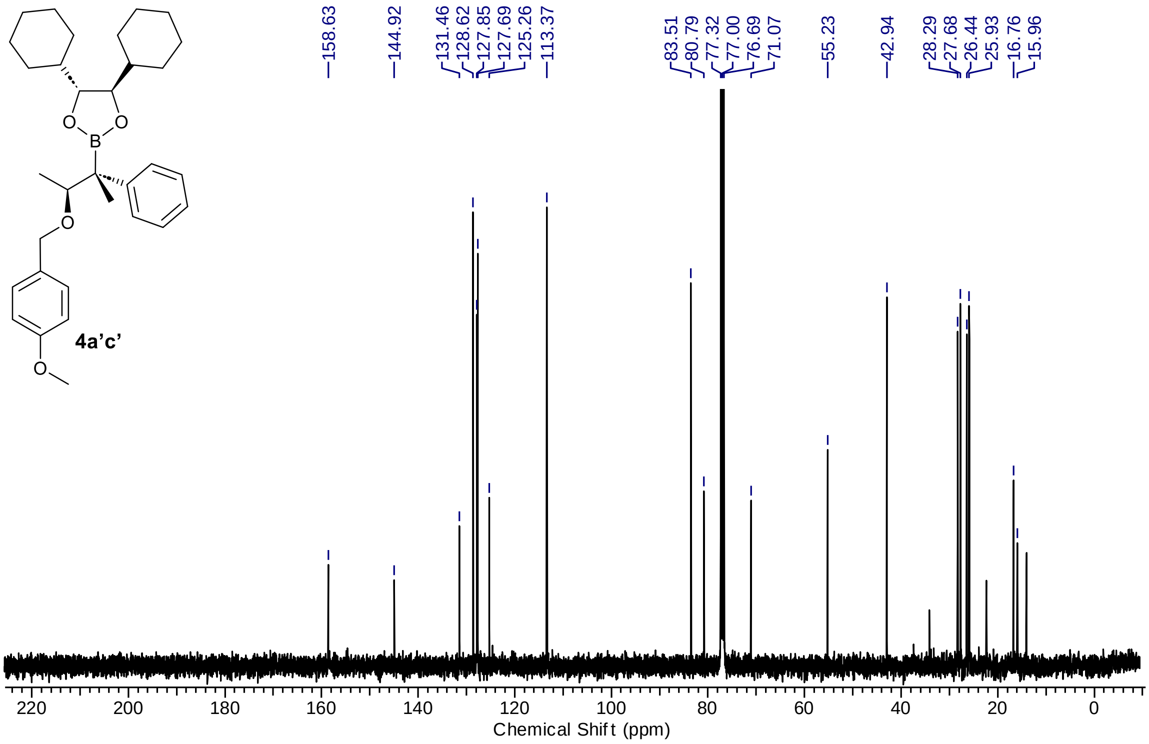

(4*R*,5*R*)-4,5-Dicyclohexyl-2-((2*R*,4*S*)-4-((4-methoxybenzyl)oxy)-2-phenylpentan-2-yl)-1,3,2-dioxaborolane (4a’e’)

^{1}H-NMR (500 MHz, $CDCl_3$):

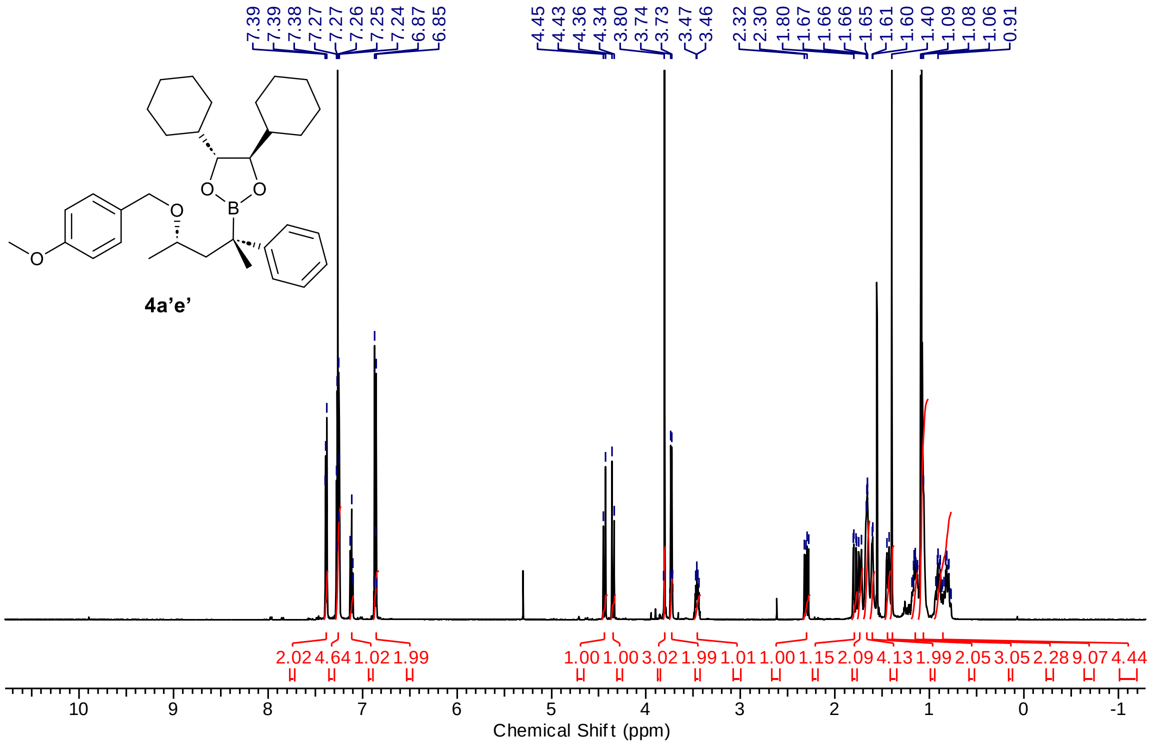

^{13}C-NMR (125 MHz, $CDCl_3$):

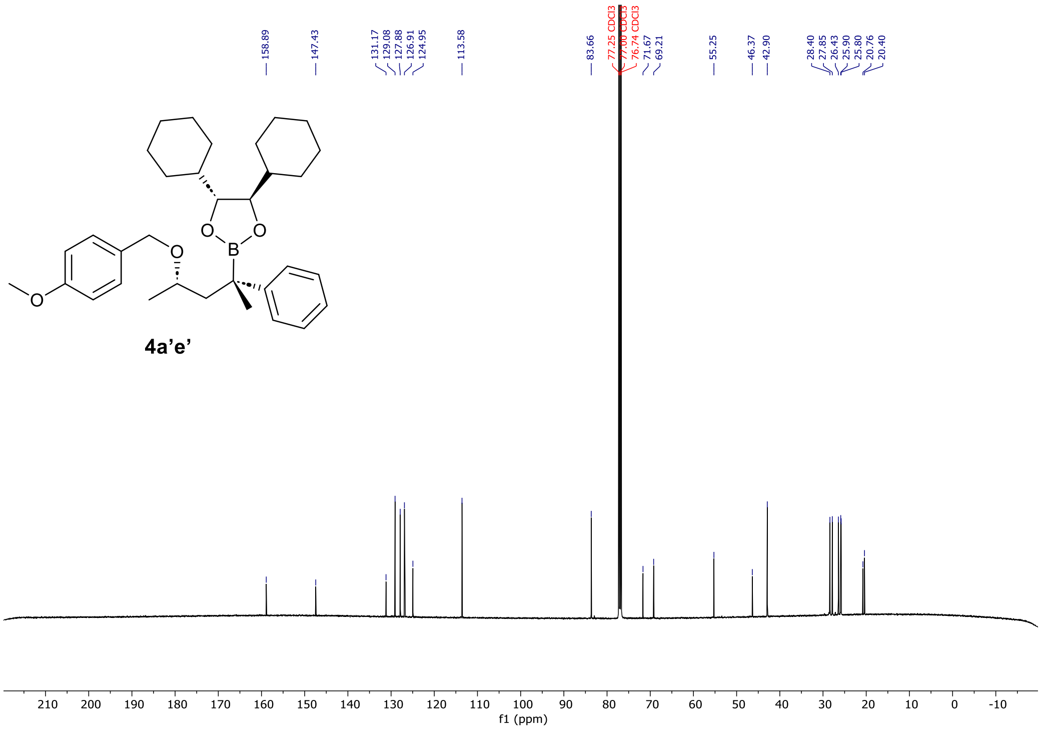

(4*R*,5*R*)-4,5-Dicyclohexyl-2-((2*R*,3*S*)-3-((4-methoxybenzyl)oxy)-2-(4-methoxyphenyl)-butan-2-yl)-1,3,2-dioxaborolane (4cc')

^{1}H-NMR (400 MHz, $CDCl_3$):

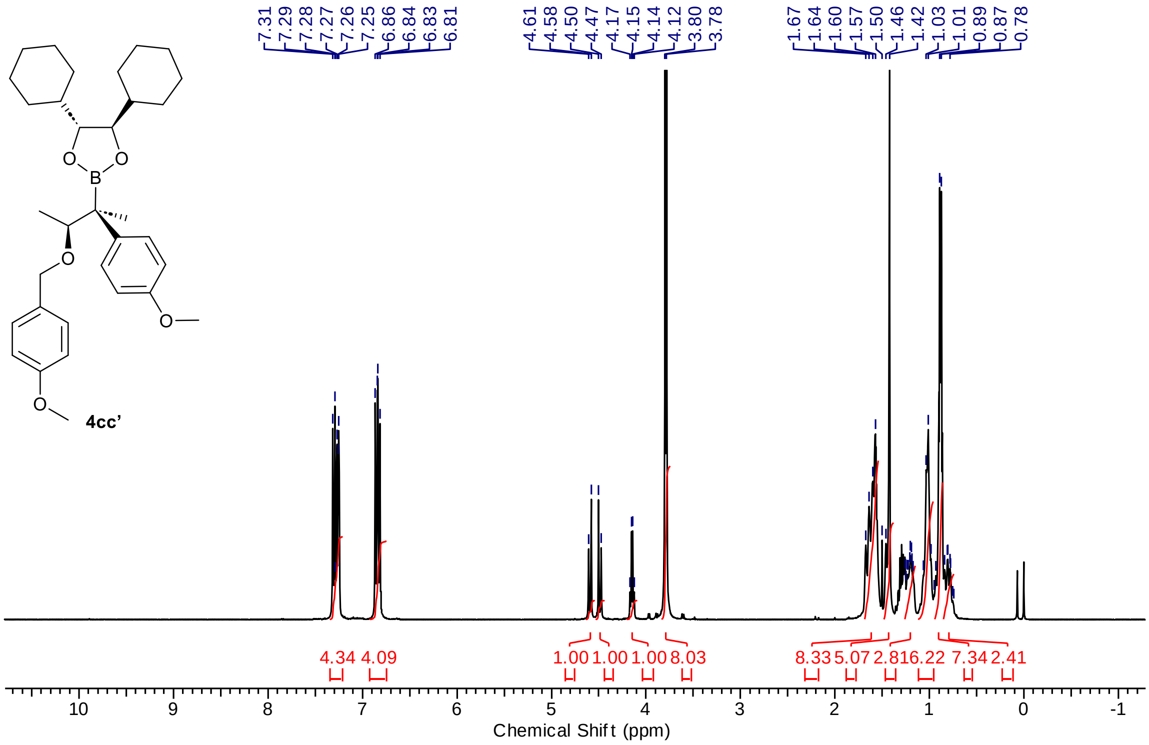

^{13}C-NMR (100 MHz, $CDCl_3$):

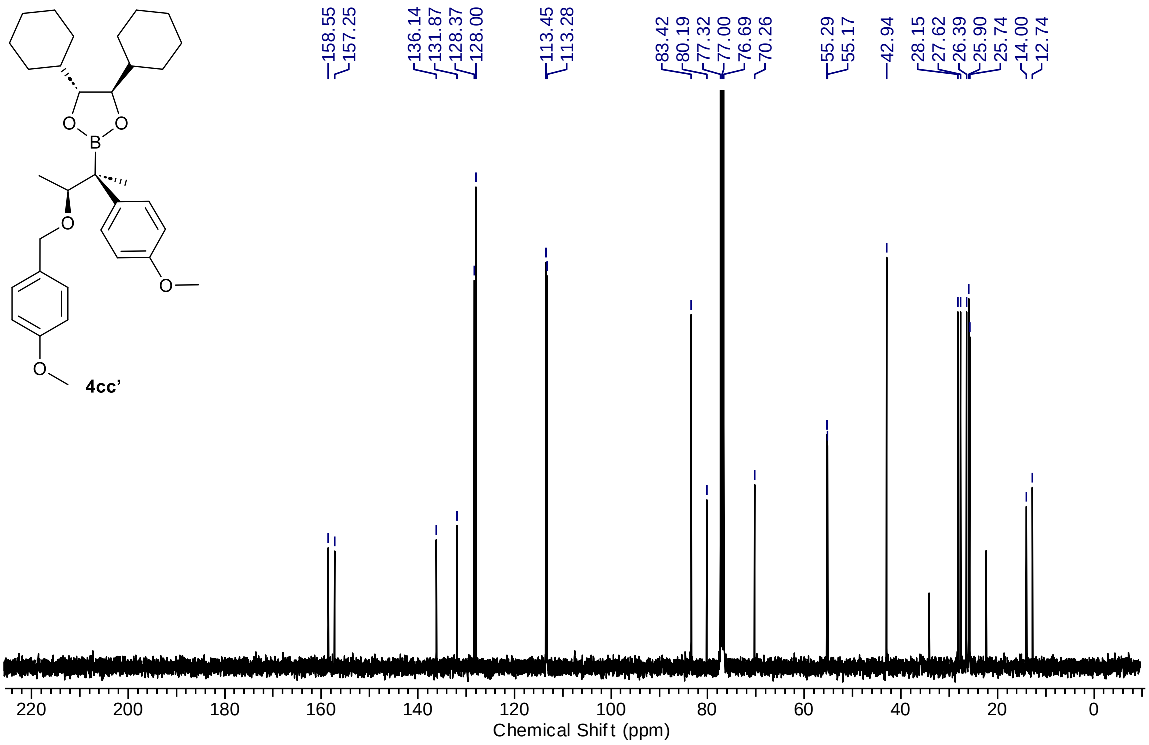

(4*S*,5*S*)-4,5-Dicyclohexyl-2-((2*S*,3*R*)-3-(2-((4-methoxybenzyl)oxy)ethyl)-2-(3-methoxyphenyl)hex-5-en-2-yl)-1,3,2-dioxaborolane (4dg)

^{1}H-NMR (400 MHz, $CDCl_3$):

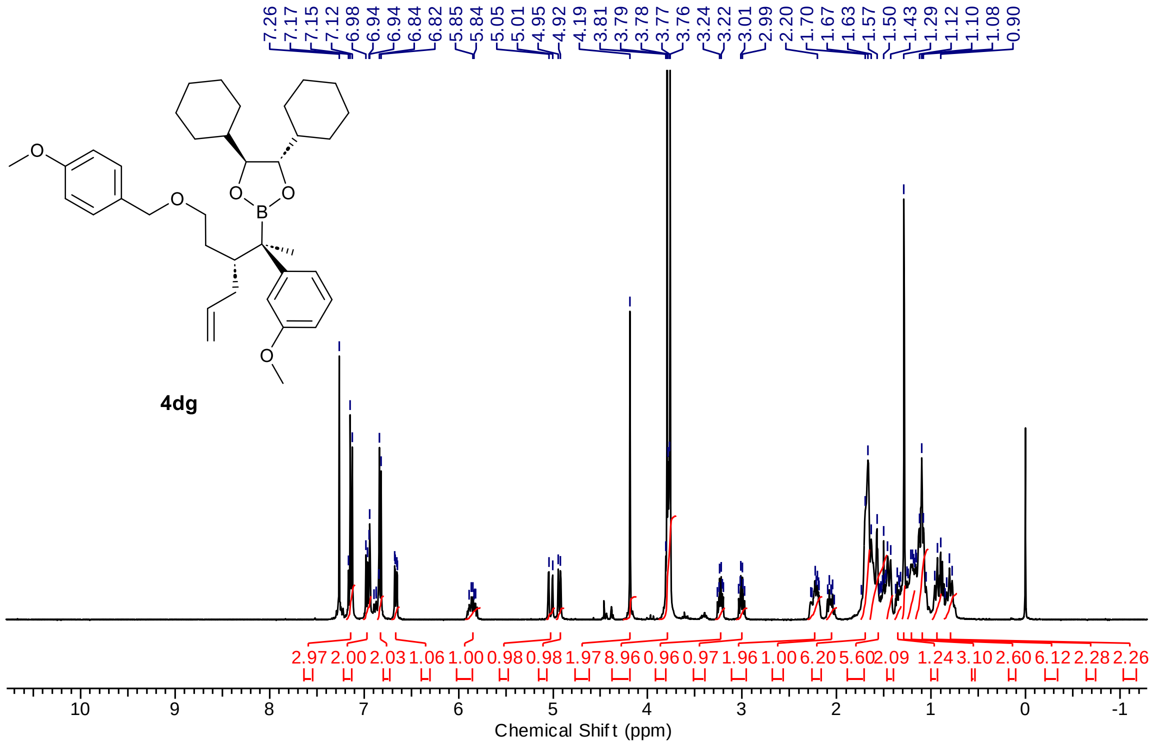

^{13}C-NMR (100 MHz, $CDCl_3$):

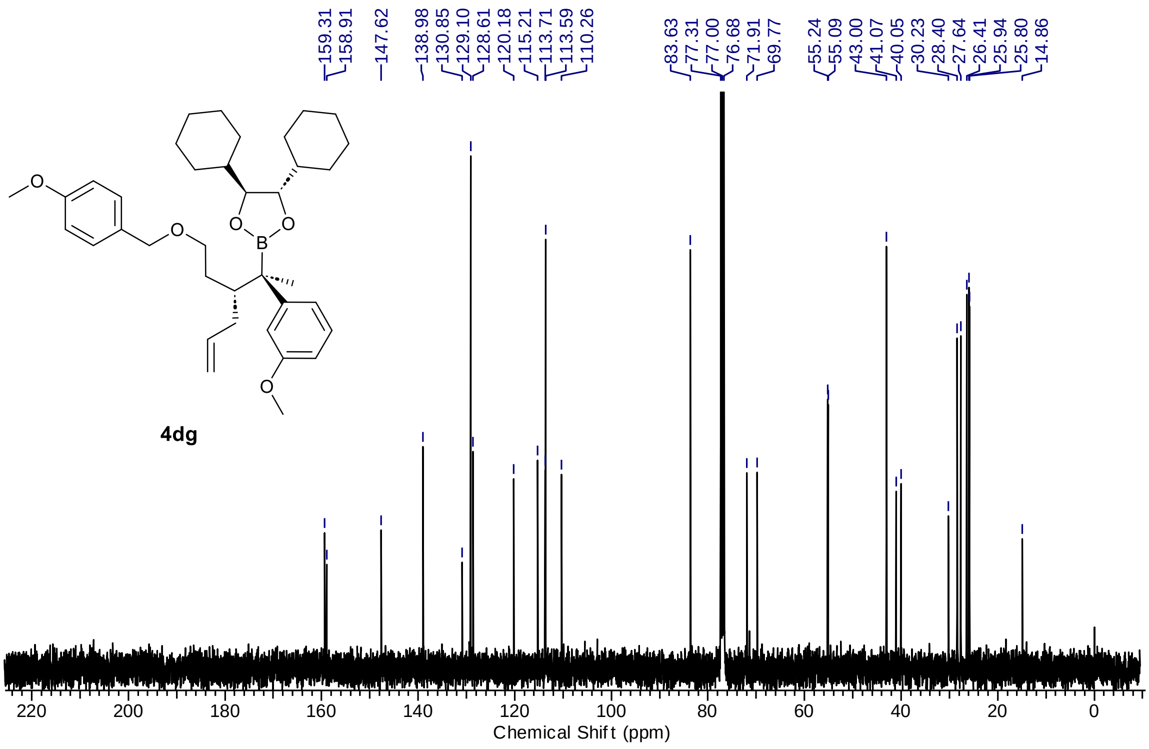

(4*R*,5*R*)-4,5-Dicyclohexyl-2-((4*S*,6*S*,*E*)-4-ethyl-6-((4-methoxybenzyl)oxy)hept-2-en-4-yl)-1,3,2-dioxaborolane (4ge')

^{1}H-NMR (400 MHz, CDCl$_3$):

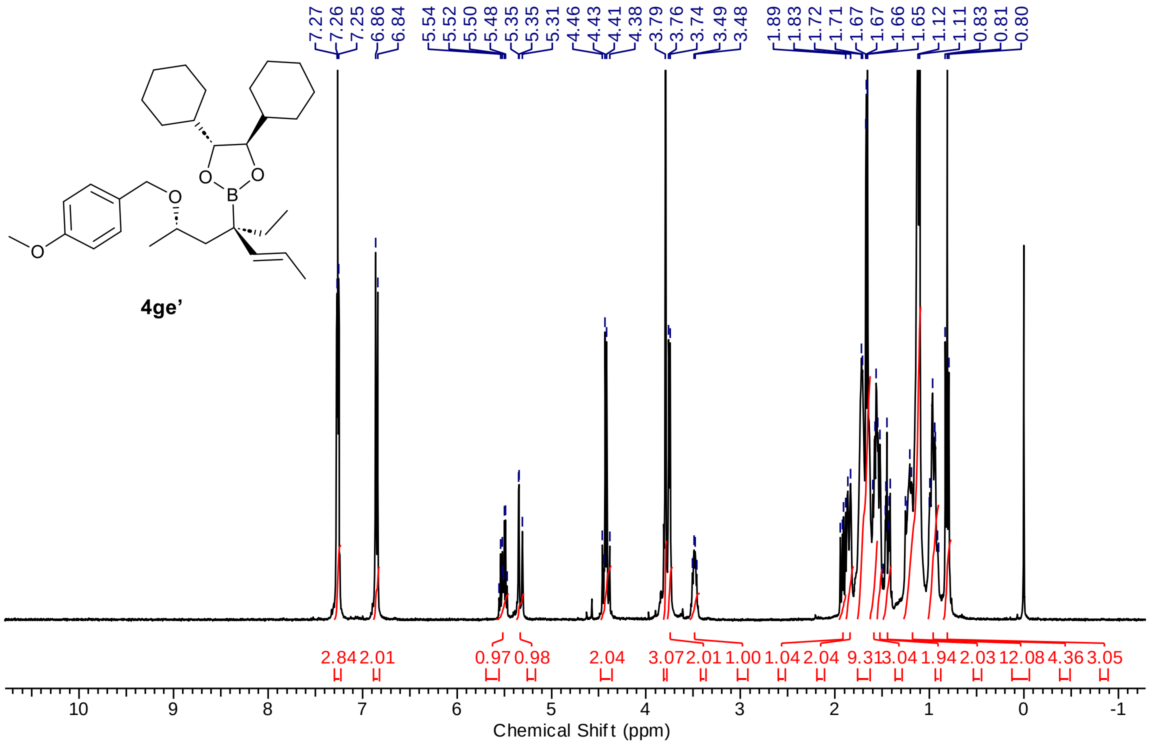

^{13}C-NMR (100 MHz, CDCl$_3$):

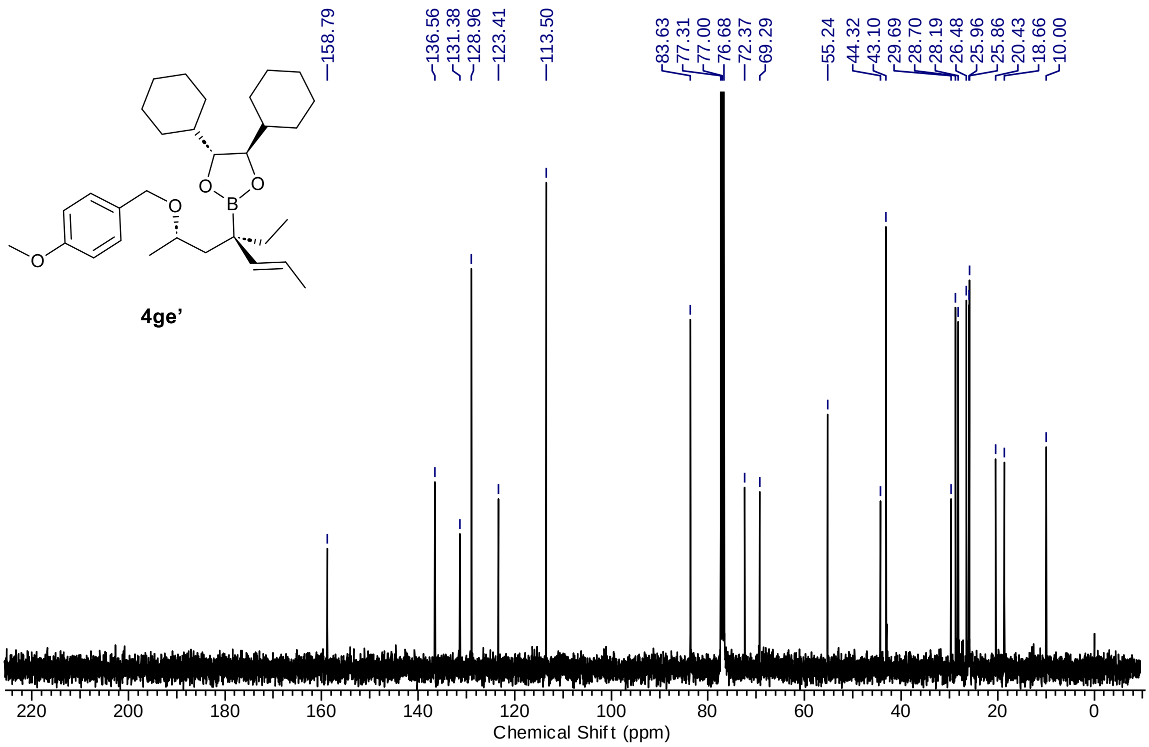

(4*R*,5*R*)-4,5-Dicyclohexyl-2-((2*S*,3*R*,5*S*)-2-((4-methoxybenzyl)oxy)-5-methyl-3-phenyloctan-3-yl)-1,3,2-dioxaborolane (4hc’)

^{1}H-NMR (400 MHz, $CDCl_3$):

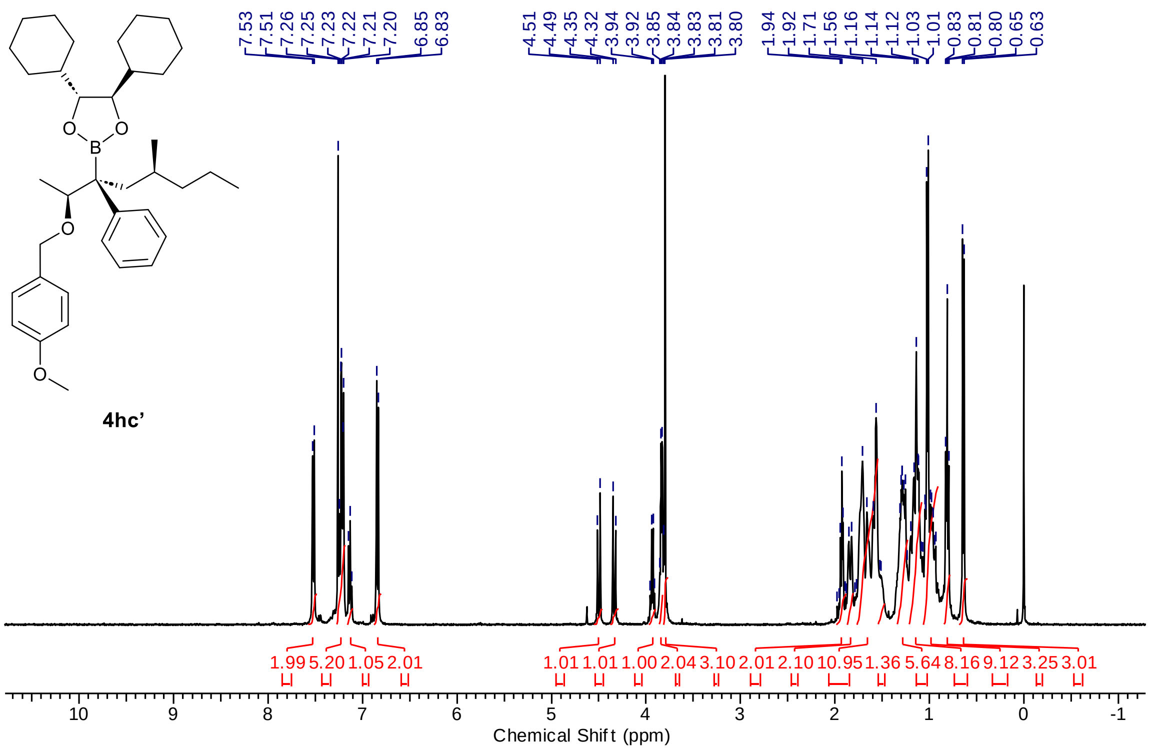

^{13}C-NMR (100 MHz, $CDCl_3$):

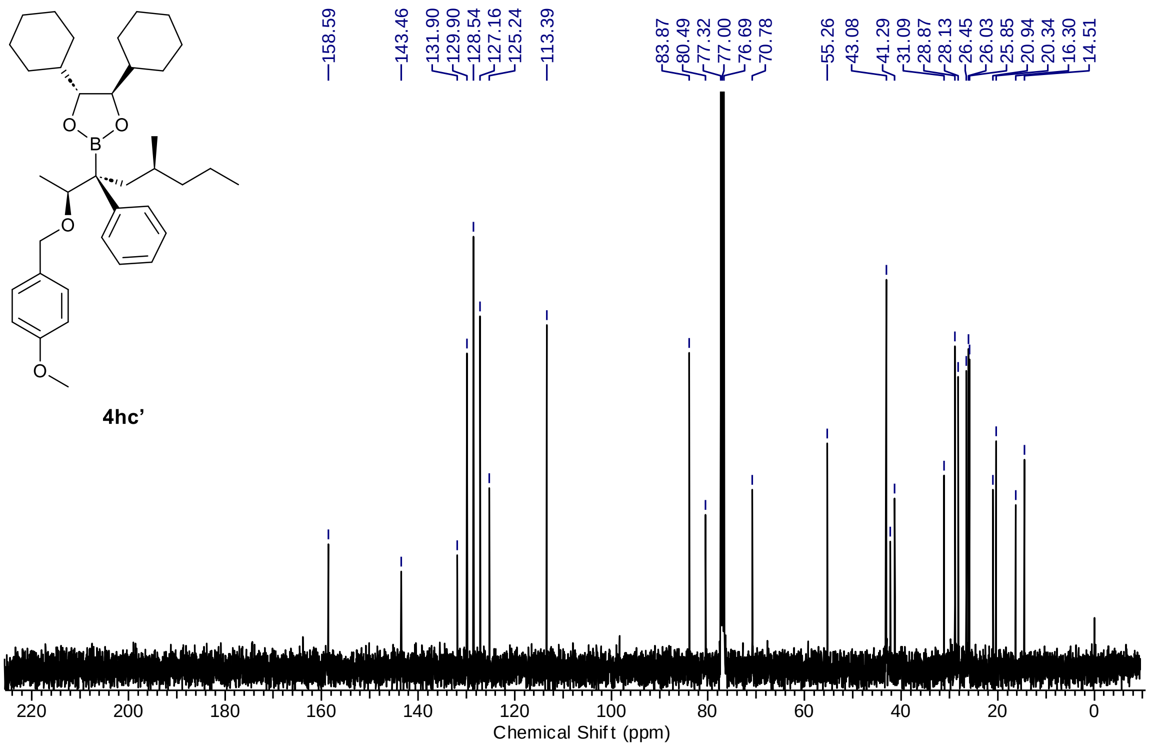

(4*R*,5*R*)-4,5-Dicyclohexyl-2-((2*S*,4*S*,6*S*)-2-((4-methoxybenzyl)oxy)-6-methyl-4-phenylnonan-4-yl)-1,3,2-dioxaborolane (4he')

^{1}H-NMR (400 MHz, $CDCl_3$):

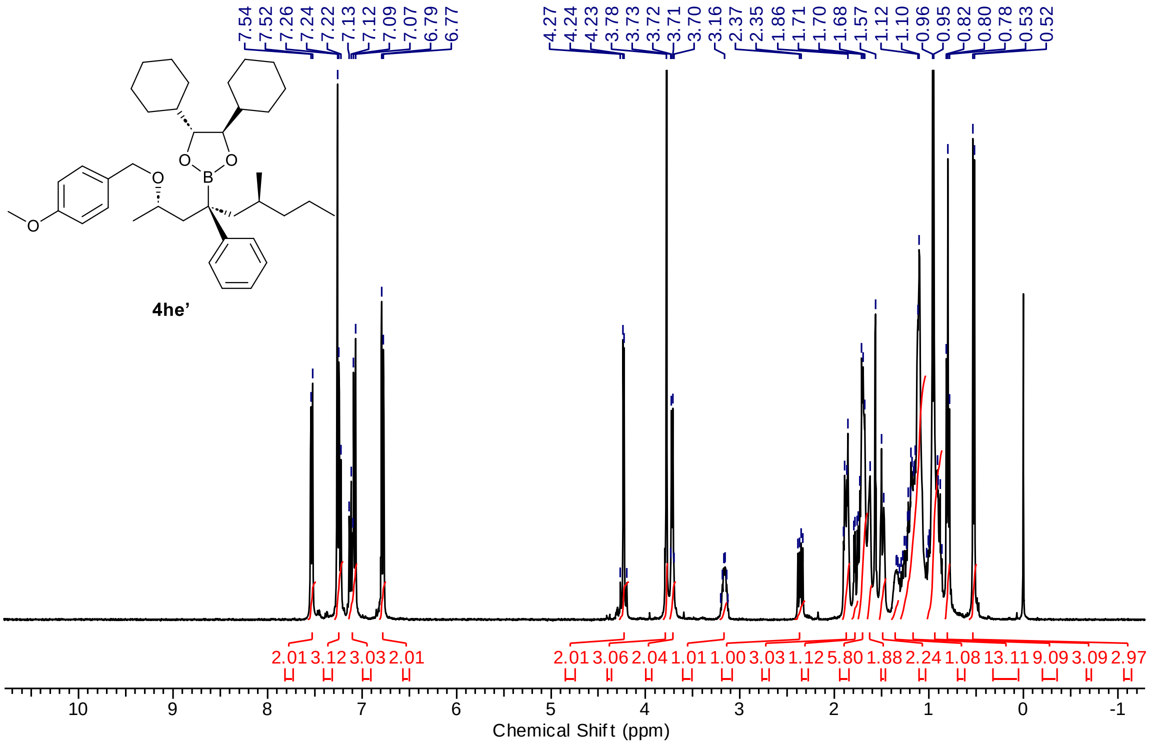

^{13}C-NMR (100 MHz, $CDCl_3$):

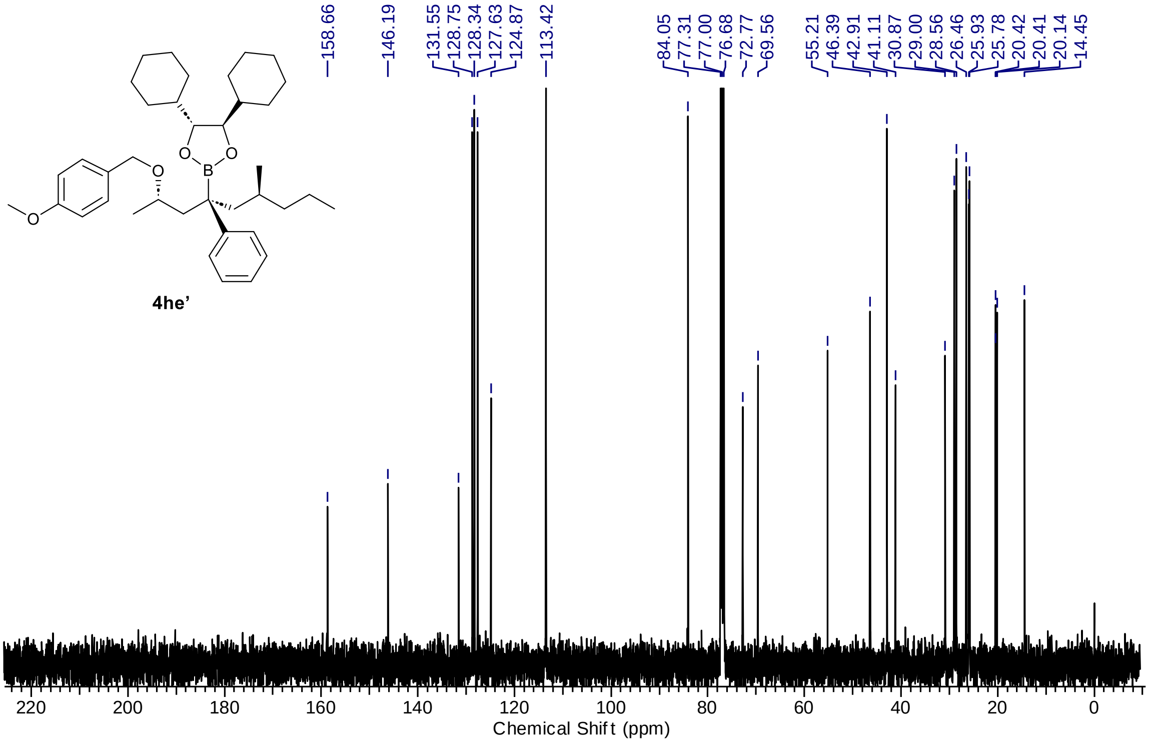

(4R,5R)-2-((1S,2S,4S)-1-chloro-4-((4-methoxybenzyl)oxy)-2-methyl-2-phenylpentyl)-4,5-dicyclohexyl-1,3,2-dioxaborolane (4a'e'-Cl)

^{1}**H-NMR** (500 MHz, $CDCl_3$):

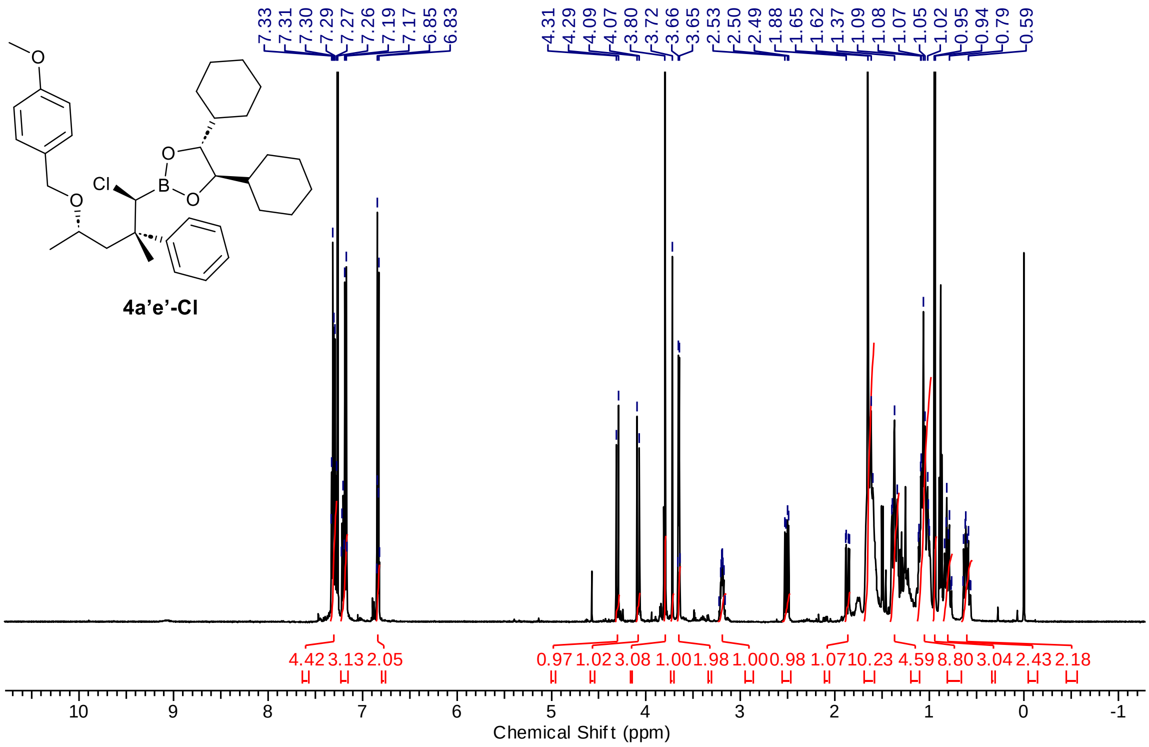

^{13}**C-NMR** (125 MHz, $CDCl_3$):

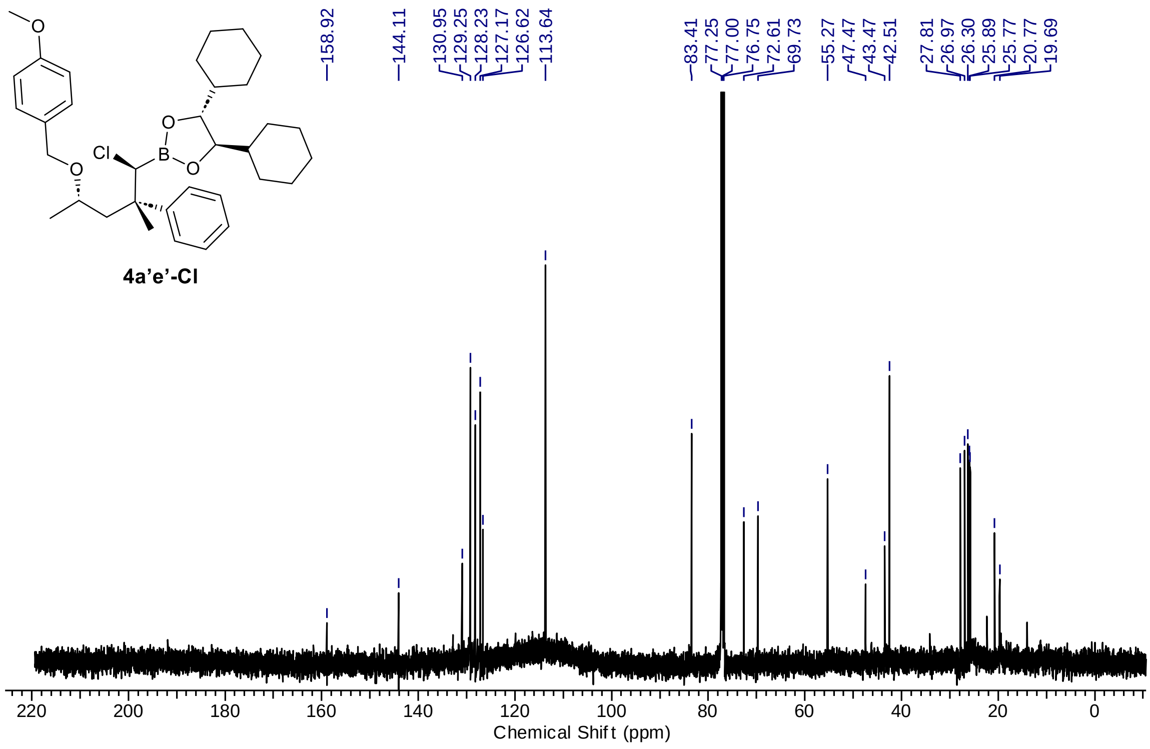

(4*R*,5*R*)-4,5-Dicyclohexyl-2-((2*S*,4*S*)-4-((4-methoxybenzyl)oxy)-2-methyl-2-phenylpentyl)-1,3,2-dioxaborolane (5 a'e'):

^{1}H-NMR (500 MHz, $CDCl_3$):

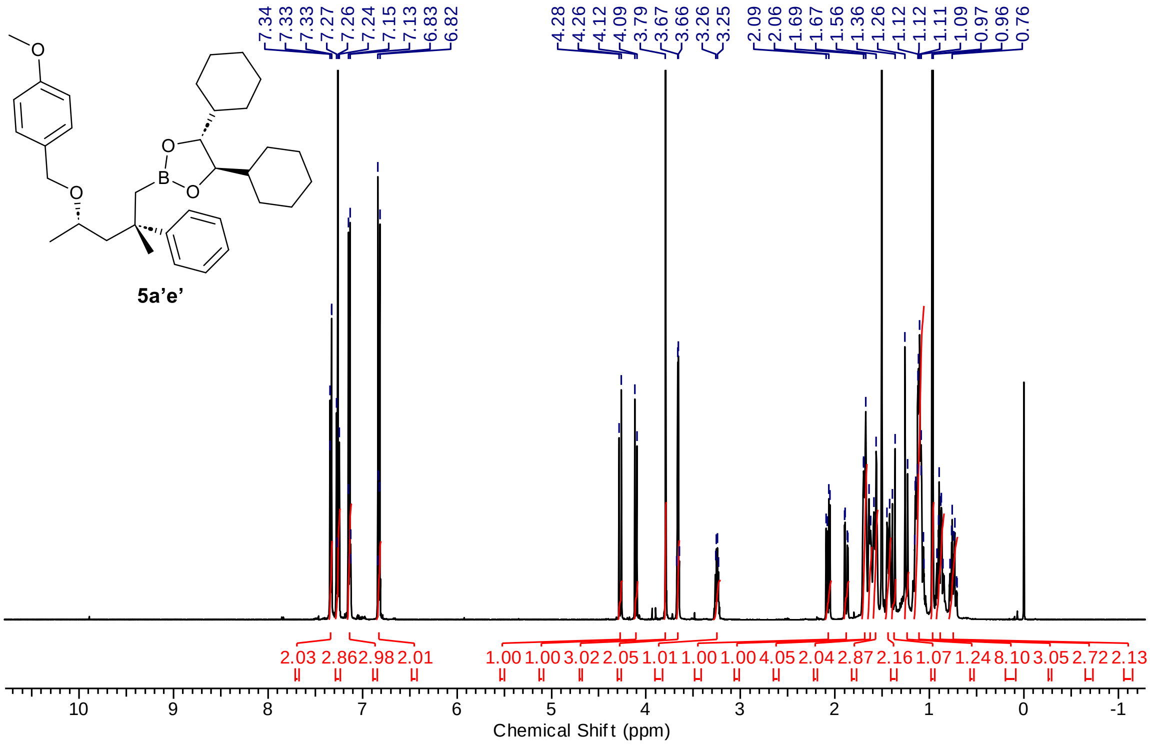

^{13}C-NMR (125 MHz, $CDCl_3$):

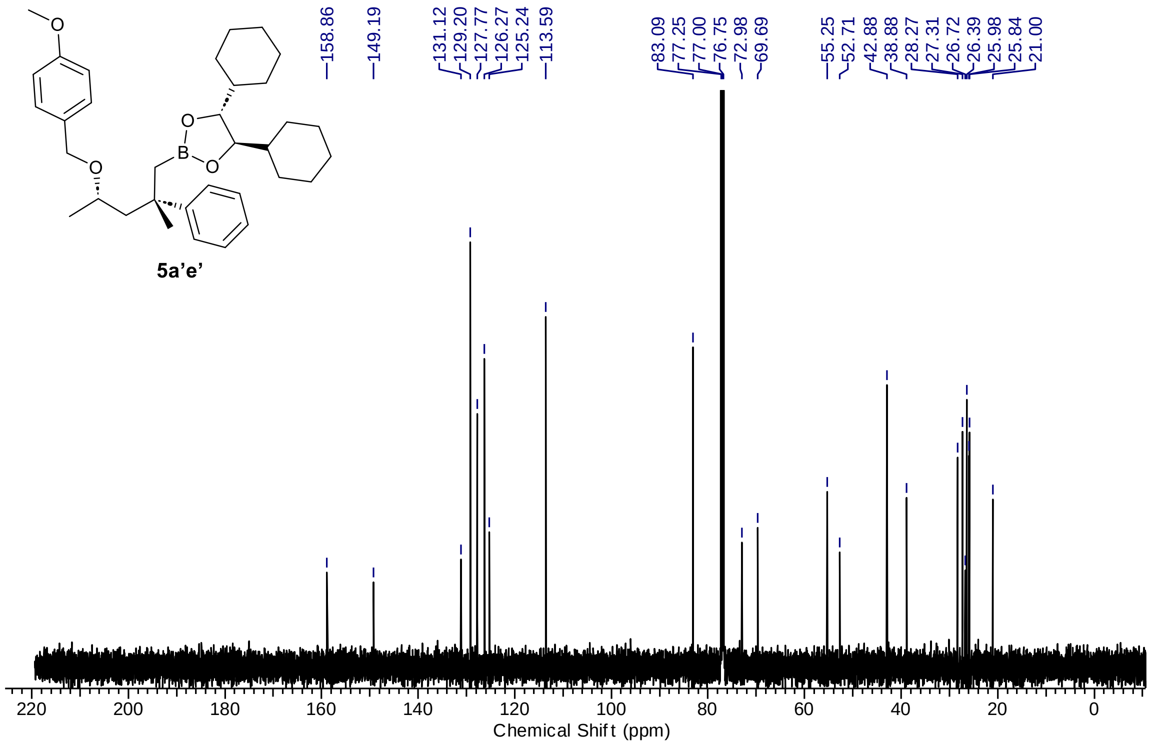

(2*S*,4*S*)-4-((4-Methoxybenzyl)oxy)-2-methyl-2-phenylpentanal (6a'e')

^{1}H-NMR (500 MHz, $CDCl_3$):

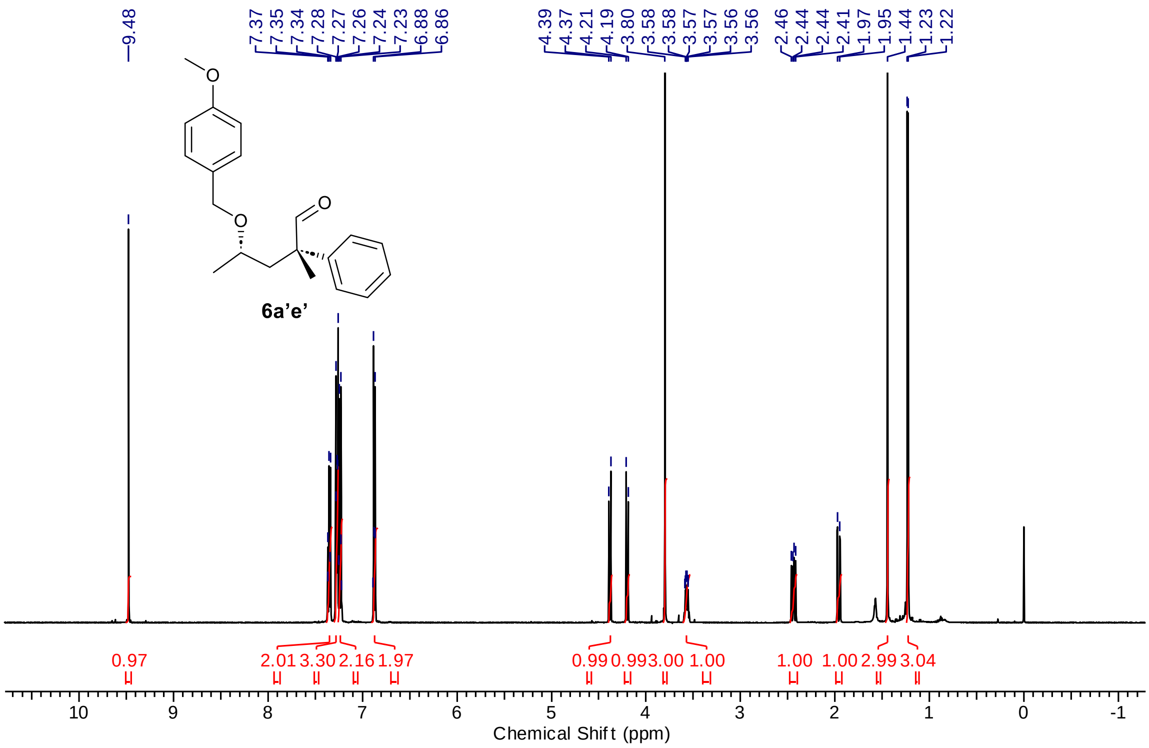

^{13}C-NMR (125 MHz, $CDCl_3$):

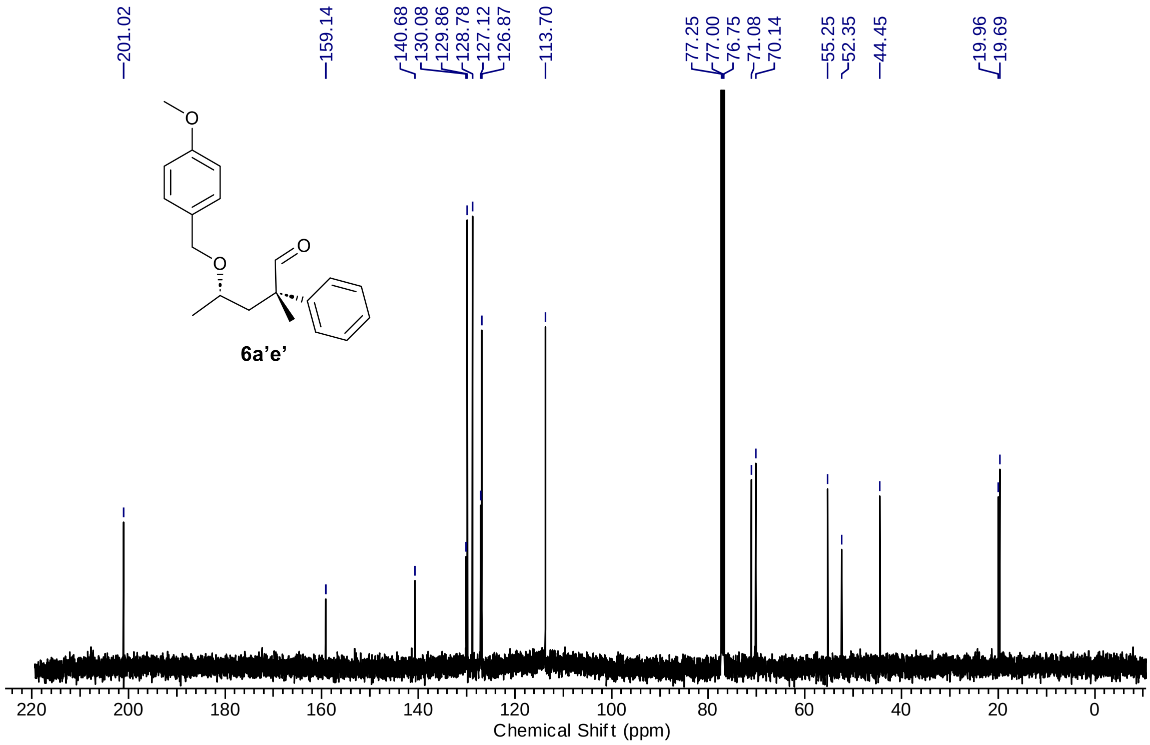

1-Methoxy-4-((((2*S*,4*R*)-4-phenylpentan-2-yl)oxy)methyl)benzol (7a'e')

^{1}H-NMR (500 MHz, $CDCl_3$):

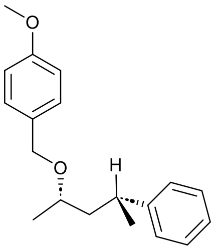

7a'e'

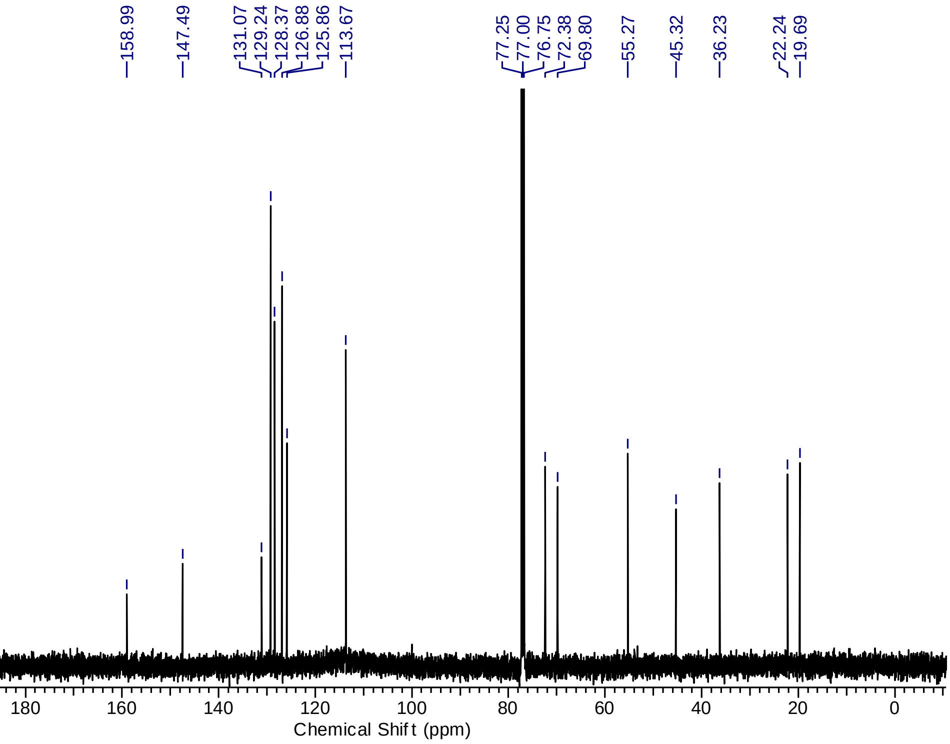

^{13}C-NMR (125 MHz, $CDCl_3$):

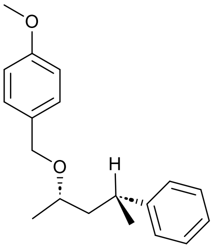

7a'e'

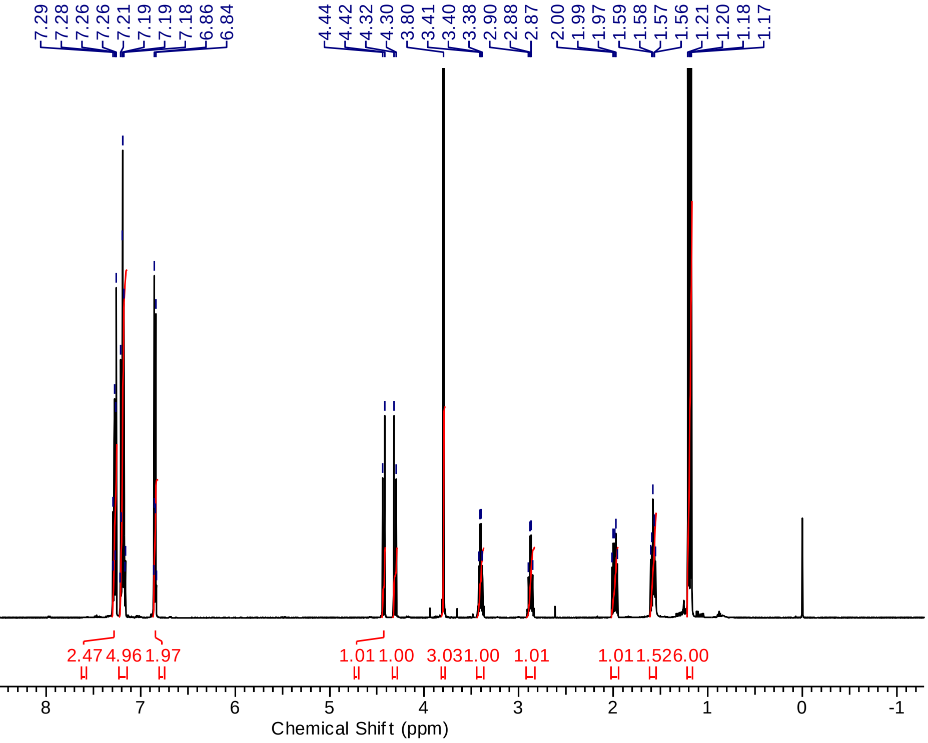

$^1H,^1H$-COSY

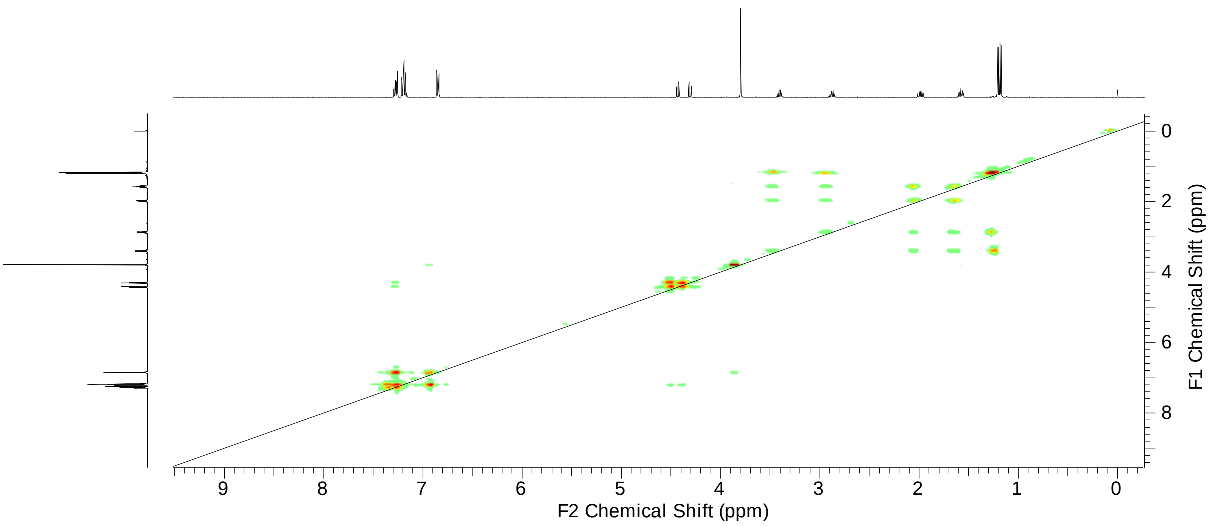

$^1H,^{13}C$-HSQC

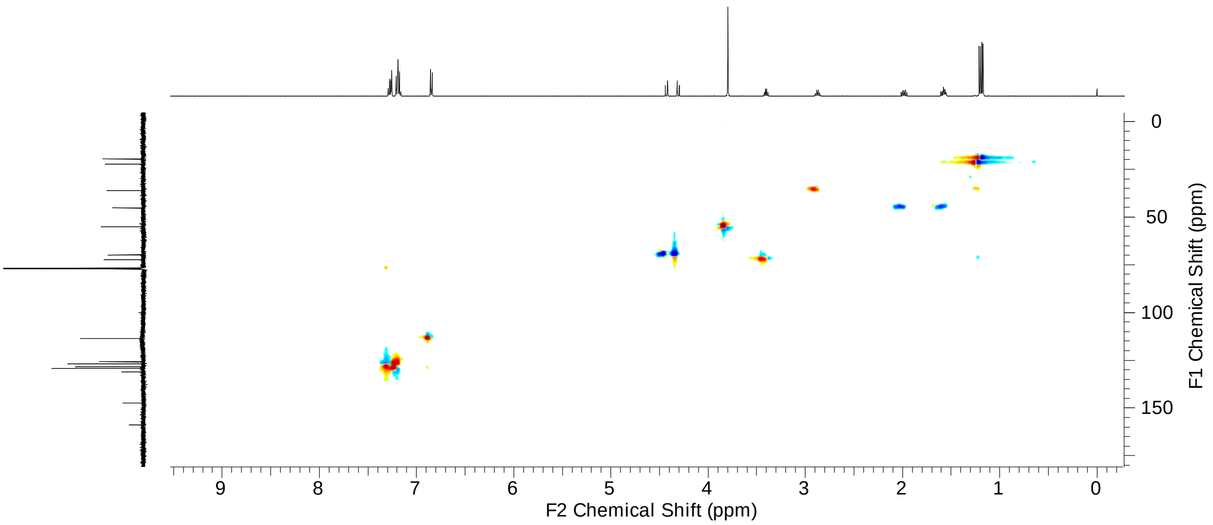

$^1H,^{13}C$-HMBC

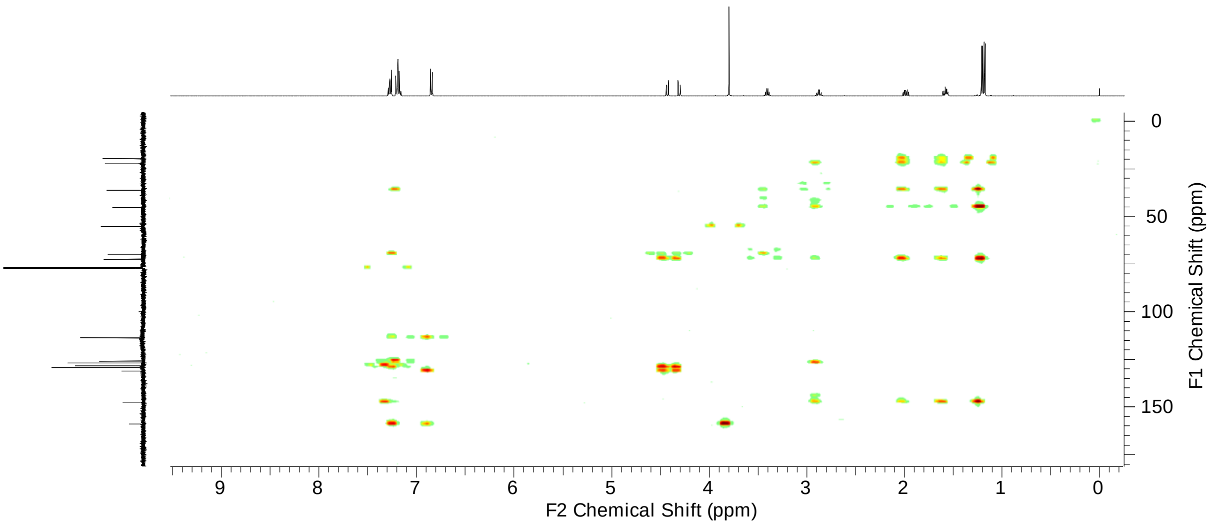

1-Methoxy-4-((((2*S*,4*R*)-4-methyl-4-phenylhex-5-en-2-yl)oxy)methyl)benzol (8a'e')

^{1}H-NMR (500 MHz, $CDCl_3$):

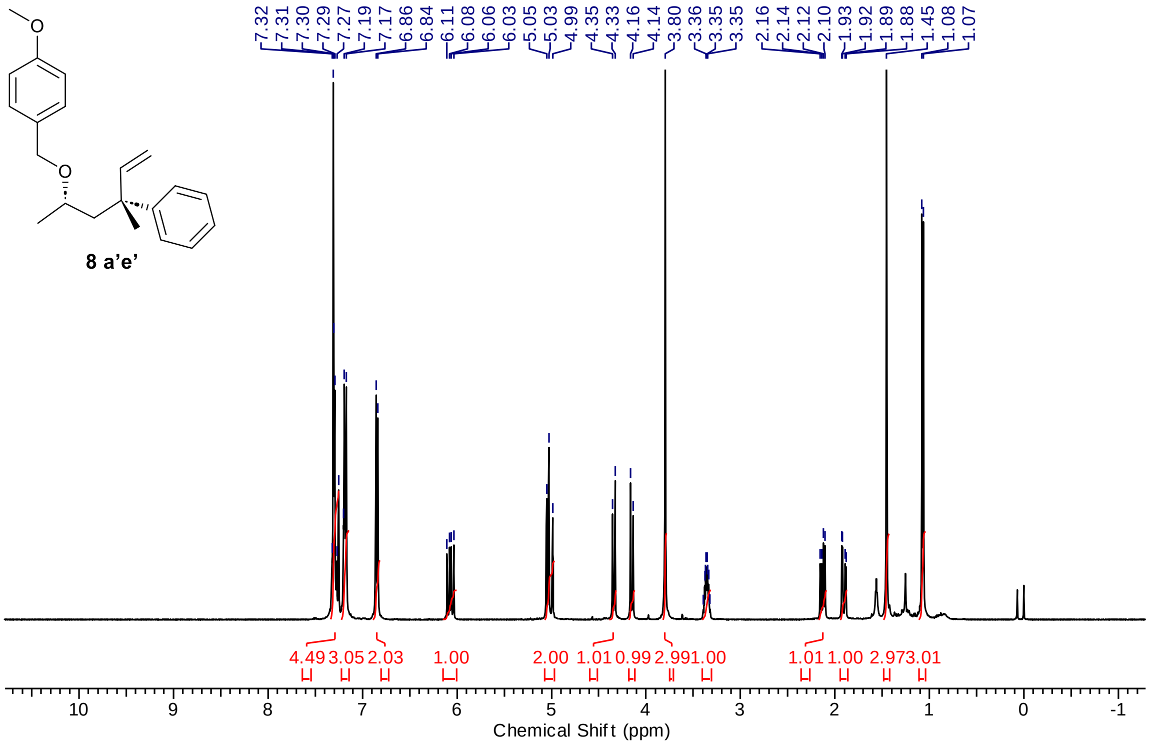

^{13}C-NMR (125 MHz, $CDCl_3$):

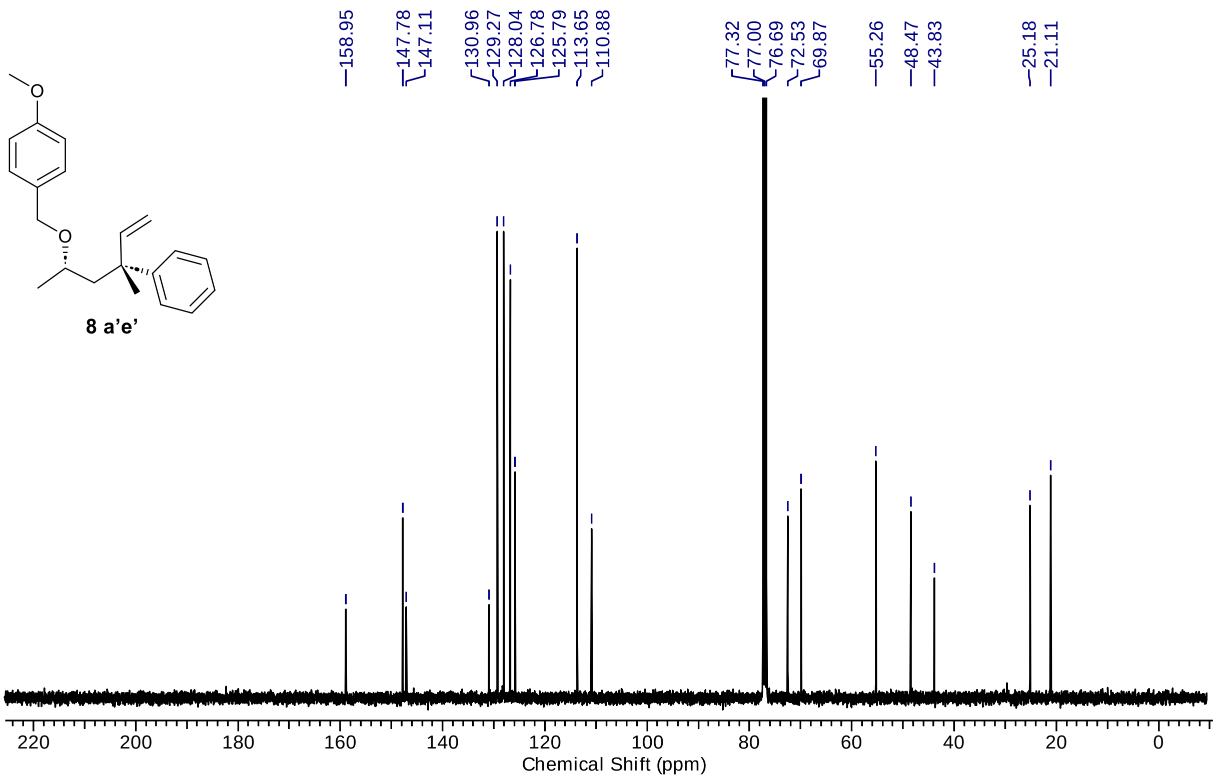

References

[1] S. Roesner, D. J. Blair, V. K. Aggarwa, Enantioselective installation of adjacent tertiary benzylic stereocentres using lithiation–borylation–protodeboronation methodology. Application to the synthesis of bifluranol and fluorohexestrol, *Chem. Sci*. 2015,**6**, 3718-3723.

[2] D. J. Blair, C. J. Fletcher, K. M. P. Wheelhouse, V. K. Aggarwal, Stereocontrolled Synthesis of Adjacent Acyclic Quaternary-Tertiary Motifs: Application to a Concise Total Synthesis of (−)-Filiformin, *Angew. Chem. Int. Ed*. 2014, **53**, 5552-5555.

[3] V. Bagutski, R.M. French, V. K. Aggarwal, Full Chirality Transfer in the Conversion of Secondary Alcohols into Tertiary Boronic Esters and Alcohols Using Lithiation–Borylation Reactions, *Angew. Chem. Int. Ed*. 2010, **49**, 5142-5145.

[4] A. P. Pulis, V. K. Aggarwal, Synthesis of Enantioenriched Tertiary Boronic Esters from Secondary Allylic Carbamates. Application to the Synthesis of C30 Botryococcene, *J. Am. Chem. Soc.* 2012, **134**, 7570−7574.

[5] T. Kinsinger, U. Kazmaier, Application of Vinyl Nucleophiles in Matteson Homologations, *Org. Lett.* 2022, **24**, 3599–3603.

[6] M. Tost, O. Andler, U. Kazmaier, A Matteson Homologation-Based Synthesis of Doliculide and Derivatives, *Eur. J. Org. Chem.* 2021, **2021**, 6459-6471.
